# Robust differential expression analysis by learning discriminant boundary in multi-dimensional space of statistical attributes

**DOI:** 10.1186/s12859-016-1386-x

**Published:** 2016-12-19

**Authors:** Yuanzhe Bei, Pengyu Hong

**Affiliations:** Computer Science Department, Brandeis University, Waltham, MA 02453 USA

**Keywords:** Differential expression analysis, Discriminant boundary learning, False discovery rate, Discriminant-Cut

## Abstract

**Background:**

Performing statistical tests is an important step in analyzing genome-wide datasets for detecting genomic features differentially expressed between conditions. Each type of statistical test has its own advantages in characterizing certain aspects of differences between population means and often assumes a relatively simple data distribution (e.g., Gaussian, Poisson, negative binomial, etc.), which may not be well met by the datasets of interest. Making insufficient distributional assumptions can lead to inferior results when dealing with complex differential expression patterns.

**Results:**

We propose to capture differential expression information more comprehensively by integrating multiple test statistics, each of which has relatively limited capacity to summarize the observed differential expression information. This work addresses a general application scenario, in which users want to detect as many as DEFs while requiring the false discovery rate (FDR) to be lower than a cut-off. We treat each test statistic as a basic attribute, and model the detection of differentially expressed genomic features as learning a discriminant boundary in a multi-dimensional space of basic attributes. We mathematically formulated our goal as a constrained optimization problem aiming to maximize discoveries satisfying a user-defined FDR. An effective algorithm, Discriminant-Cut, has been developed to solve an instantiation of this problem. Extensive comparisons of Discriminant-Cut with 13 existing methods were carried out to demonstrate its robustness and effectiveness.

**Conclusions:**

We have developed a novel machine learning methodology for robust differential expression analysis, which can be a new avenue to significantly advance research on large-scale differential expression analysis.

**Electronic supplementary material:**

The online version of this article (doi:10.1186/s12859-016-1386-x) contains supplementary material, which is available to authorized users.

## Background

High-throughput technologies, such as DNA microarray [1, 2] and RNA-seq (RNA sequencing) [3], have made it possible to perform genome-wide profiling of various genomic features, such as, genes, transcripts, exons, DNA modifications, and so on. These technologies have been widely adopted to detect genomic features (referred to as “features” from now on) that are differentially expressed between different conditions (e.g., phenotypes, treatments, etc.). When analyzing genome-wide datasets to detect differentially expressed features (DEFs), it is important to control the overall false positive rate because thousands of hypotheses are tested simultaneously. Controlling the false discovery rate (FDR – the expected proportion of false positives among all features called significant) was first introduced by Benjamini and Hochberg [4] to large-scale testing problems and has been broadly applied in detecting DEFs since then. The Benjamini-Hochberg (BH) approach takes the *p*-values of all hypothesis tests and uses a sequential method to estimate the rejection region (i.e., *p*-value threshold). More recently, researchers formulated FDR estimation in a Bayesian fashion [5–7], which assumes the distribution of the statistic as a density mixed of nulls and alternatives. The Bayesian approaches can be implemented non-parametrically using the test statistics directly rather than their *p*-values. The calculation of test statistics (and their *p*-values) can be deemed as a mapping from the original high-dimensional observations to a single index value per feature. The ordinary *t*-test [8] was one of the most popular mappings for detecting differential expressions measured by DNA microarrays. The *t*-test assumes normality in the target data and can be prone to outliers. In addition, its variance estimation is feature specific and is impacted by great variability when only few samples/replicates are available in DNA microarray experiments. To deal with this problem, various versions of moderated *t*-statistics [6, 9–16] were developed to utilize information across features for regularizing variance estimation.

Statistics based on the Poisson distribution [17, 18] or the negative binomial (NB) distribution [19–22] were later proposed specifically for detecting DEFs using RNA-seq data. Different from typical DNA microarray approaches that rely on hybridization to measure the expression levels of features as continuous values, RNA-seq approaches use deep sequencing to produce millions of short reads corresponding to those features. The reads are then mapped onto a reference genome, which makes Poisson a natural representation of read counts. It was shown that the Poisson distribution was able to effectively characterize technical replicates in RNA-seq experiments [17]. However, the Poisson distribution forced the mean and variance to be the same and predicts a smaller variance than what was observed in biological replicates [23]. To deal with this so-called over-dispersion problem, PoissonSeq [24] applied a power transformation to make the data distribution look more like Poisson. Auer [25] proposed a two-stage Poisson model (TSPM) to handle features with significant over-dispersion evidence by a quasi-likelihood approach [26]. In the meantime, the NB distribution was proposed as an alternative [23] and has been gaining momentum in analyzing RNA-seq data. Compared to the Poisson distribution, the NB distribution allows the modeling of a more general mean-variance relation by taking another dispersion parameter. Several NB-based approaches, such as DESeq [20], DESeq2 [27], edgeR [22], NBPSeq [28], EBSeq [29], baySeq [30], ShrinkSeq [31], and so on, have been developed, and they mainly differ in their ways of modeling and estimating the dispersion parameter.

Recently, it was demonstrated that the moderated *t*-statistic, when combined with appropriate data preprocessing methods, could be powerful for detecting DEFs using RNA-seq data. For example, voom [32] extended limma [11], which uses the moderated *t*-statistic in a pipeline well-established for analyzing DNA microarray data, for differential expression analysis using RNA-seq data. Voom applies a logarithmic transforms to read-counts normalized by the corresponding library size, estimates the mean-variance relationship non-parametrically from the transformed data, uses the estimated relationship to generate a precision weight for each normalized observation, and finally enters them into the limma empirical Bayes analysis pipeline for detecting DEFs. In another example, vst/limma [33] applied the variance-stabilizing transformation (vst) of DESeq to RNA-seq data before using limma to calculate the moderated *t*-statistic.

The above test statistics can be viewed as attributes extracted from data to characterize the observed differential expression patterns. Most existing attribute extraction methods make specific assumptions about data distributions (e.g., Gaussian, Poisson, or NB), and then calculate a statistic (i.e., an attribute in our words) for each feature. Although those test statistics are efficient in preserving differential expression information up to certain levels, they leave plenty of room for further improvements. In real applications, the profiles of individual features in the same dataset can be governed by complex distributions, and hence may not be well represented by the assumed distribution [34]. We made a similar observation that the distributions could indeed be far more complex than those often assumed (see Figs. 2 and 11 in the Results section for examples). Individual attributes based on relatively simple distribution assumptions will have limited capacity in characterizing complex differential expression patterns, and hence can greatly affect DEF detection results. In theory, we can explicitly make every differential expression test follow a common family of distributions by designing a complex distribution form (e.g., mixture of simple distributions) to approximate all complex distributions in data. Such a complex distribution will have unknown parameters that can be estimated from data by applying the same procedure to all features. However, it can be challenging to design not only a statistic for testing differential expressions based on such a complex distribution but also a parametric DEF detection approach that uses this test statistic.

There are non-parametric approaches that do not assume data distribution, such as, SAMSeq [34] and NOISeq [35]. SAMseq utilizes the ranksum test statistic [36] to characterize differential expressions and uses resampling to adjust for different library sizes. Although the ranksum test does not assume any data distribution and is less likely to be affected by outliers, it can sometimes be considerably less capable of preserving information. NOISeq uses two simple attributes (log fold-changes and absolute expression differences), and estimates the null as the joint distribution of these two attributes from replicates (or replicates simulated from an empirically determined multinomial distribution), which is then used to calculate the odds of an observed statistic pair indicating differential expression. Nevertheless, NOISeq does not directly estimate FDR. In addition, log fold-changes and absolute expression differences can be prone to outliers and are not powerful enough for characterizing complex differential expression patterns. However, NOISeq motivated us to investigate better ways for integrating multiple attributes to detect DEFs while controlling the FDR.

In this paper, we call the above attributes “basic” because of their relatively simple forms and limited capacity in preserving differential expression information. Most of the existing DEF detection methods rely on one single basic attribute in each analysis run, which can greatly restrict their detection power. Since different basic attributes may capture distinct aspects of differential expression patterns, we anticipate that DEFs can be better differentiated from non-DEFs using multiple basic attributes, which may be extracted from data using existing tools, such as, DESeq2, voom, limma, and so on. This work addresses a general application scenario, in which users set a target FDR and ask a method to detect as many DEFs as possible. This can be formulated as a constrained optimization problem that tries to learn an optimal decision boundary in a space of multiple basic attributes to differentiate DEFs from non-DEFs. An algorithm Discriminant-Cut has been developed to explore the linear decision boundary family. Extensive tests were conducted to test Discriminant-Cut and compare it with several popular DEF detection methods. The results demonstrate that it is significantly advantageous to combine multiple basic attributes in detecting DEFs.

## Methods

### DEF detection as learning multi-dimensional decision boundary

Let ***G*** = {*g*_*ij*_}_*i* = 1 … *M*,*j* = 1 … *N*_ contain the values of *M* features in *N* samples, in which *g*_*ij*_ is the value of the *i*-th feature in *j*-th sample. Without loss of generality, we assume that samples are randomly selected from a population with two different conditions. Let ***Y*** = {*y*_*j*_}_*j* = 1,…,*N*_, where *y*_*j*_ be the binary condition label of the *j*-th sample. The goal is to detect features that are differentially expressed between these two conditions. We propose to treat DEF detection as finding a discriminant function *h*(∙) that specifies the decision boundary between DEFs and non-DEFs. Let *d*_*i*_ = *h*(*g*_*i*1_, *g*_*i*2_, …, *g*_*iN*_; *y*_1_, *y*_2_, …, *y*_*N*_) be the discriminant value of the *i*-th feature. The *i*-th feature is called a DEF if *d*_*i*_ > 0. The unknown parameters of *h*(∙) should be learned from ***X*** = <***G***, ***Y***>. It can be challenging to design a proper *h*(∙) in a top-down way and learn such a function. To circumvent this problem, we can take advantage of previous research achievements in designing and calculating various statistics for testing differential expression (e.g., *t*-statistic, moderated *t*-statistic, ranksum statistic, Wald statistic for NB-based differential expression tests, etc.). We let *h*(*g*_*i*1_, *g*_*i*2_, …, *g*_*iN*_; *y*_1_, *y*_2_, …, *y*_*N*_) ≜ *f*(*s*_*i*_^(1)^, *s*_*i*_^(2)^, …, *s*_*i*_^(*K*)^) where *s*_*i*_^(1)^, *s*_*i*_^(2)^, …, *s*_*i*_^(*K*)^ are *K* different basic attributes (i.e., test statistics) of the *i*-th feature. This design can be considered as a two-layer data summarization mapping with calculating the basic attributes as the first layer and *f*(∙) as the second layer. The function *f*(∙) should be much less complex than *h*(∙), and its unknown parameters can be estimated from ***X*** more easily. Our approach can be geometrically interpreted as treating each feature as a point in the multi-dimensional space of those *K* basic attributes, and learning *f*(∙) from a given dataset to specify a decision boundary between DEFs and non-DEFs in that space. Each basic attribute provides a certain point-of-view about being differentially expressed, which is then integrated by *f*(∙) to produce a more comprehensive view. We leave the detailed specification of *f*(∙) to implementation and focus on explaining the idea for now. It will be shown later in our experiments that simple instantiations of *f*(∙), such as linear functions, can deliver superior performance.

As simple as it sounds, it is in fact quite significant and innovative to explicitly model DEF detection as learning a decision boundary in a multi-dimensional space. Conventional DEF detection approaches use top-down approaches to design single attributes to characterize differential expression information, and then find decision points in one-dimensional spaces. To accurately deal with complex differential expression patterns in the traditional way, we need to design a complex data distribution and a corresponding statistic for testing differential expression, which can be challenging and often requires performing less tractable computations. Our approach is much more simple and practical, and offers a straightforward geometrical interpretation. Our novel formulation of DEF detection opens up a new avenue to advance DEF detection research by incorporating decision boundary modeling and learning techniques developed in Machine Learning community. Learning *f*(∙) from ***X*** is an unsupervised task because no feature is labeled as DEF or non-DEF in ***X***. As far as we know, this kind of unsupervised learning problem (i.e., maximizing discoveries under a FDR constraint) has not captured major attentions in Machine Learning research.

### Maximizing DEF detection by constrained optimization

Let $\mathbb{D}\left(\boldsymbol{X},f\right)={\left\{{d}_i=f\left({s}_i^{(1)},{s}_i^{(2)},\dots, {s}_i^{(K)}\right)\ \right\}}_{i=1\dots M}$ be the discriminant value set including the discriminant values of all *M* features in ***X***. We want to learn *f*(∙) from data so that the number of the detected DEFs is maximized while the FDR is under controlled by a user-defined threshold Ψ. Given a dataset ***X*** and a fixed discriminant function *f*(∙), the DEF set is indicated as

1 $$ \Gamma \left(\boldsymbol{X},f\right)=\left\{i\left|{d}_i>0,\ {d}_i\in \mathbb{D}\left(\boldsymbol{X},f\right)\right.\right\} $$

Let FDR(***X***, *f*) denote the corresponding FDR of Γ(***X***, *f*). This problem of learning *f*(∙) from ***X*** to maximize the size of Γ(***X***, *f*) subject to the FDR constraint can be mathematically written as:

2 $$ \underset{\ f\left(\cdot \right)}{ \max}\left|\Gamma \left(\boldsymbol{X},f\right)\right|\  Satisfy\ \mathrm{F}\mathrm{D}\mathrm{R}\left(\boldsymbol{X},f\right)<\Psi $$

**Fig. 1:**
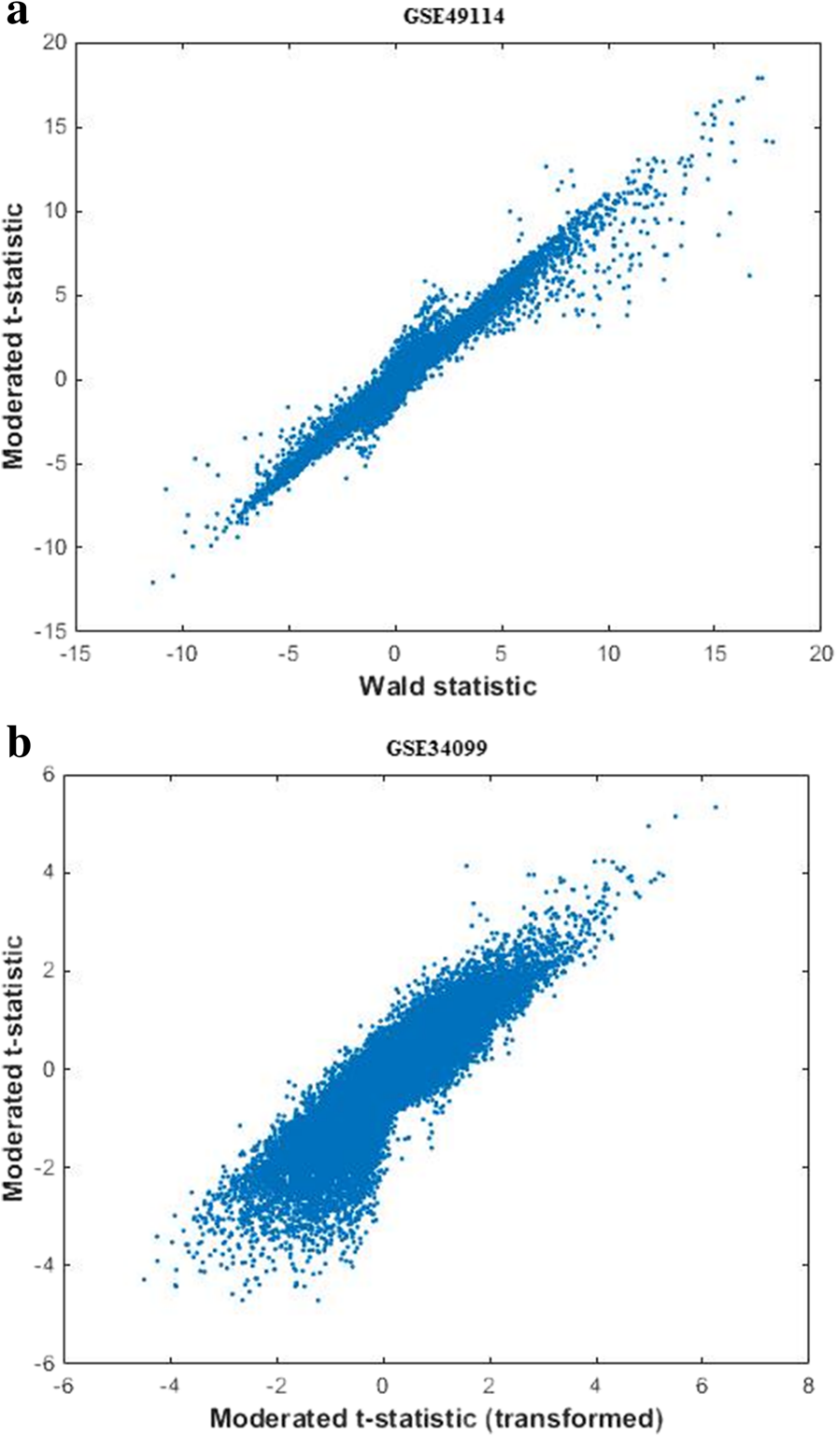
The up- and down-regulation tails may show different characteristics in the joint distribution of two basic attributes. Two typical examples are shown here. **a** A RNA-seq dataset GSE49114 (control vs. Schisto-PH): *Y*-axis – the moderated *t*-statistic from voom; *X*-axis – the Wald statistic of the NB-based differential expression test from DESeq2. **b** A DNA methylation dataset GSE34099 (Rett syndrome vs. control): *Y*-axis – the moderated *t*-statistic from limma; *X*-axis – the moderated *t*-statistic from voom. GSE49114 and GSE34099 were downloaded from the Gene Expression Omnibus database (http://www.ncbi.nlm.nih.gov/geo/)

Our approach is different from the optimal discovery procedure (ODP) [37] that tries to optimally capture common differential expression patterns shared among detected DEFs by rigorously exploring the relevant information across features to rank their significance of being differentially expressed. The current setup of ODP only allows one kind of hypothesis test for all features in each analysis run. Our approach tries to capture differential expression information of individual features as much as possible by scrutinizing their expression profiles from multiple “view angles” (i.e., using multiple basic attributes). We aim to maximize the number of detected DEFs at a given FDR level. It is possible that different numbers of DEFs can have the same FDR. It can be beneficial to treat up- and down-regulation asymmetrically (i.e., using different discriminant functions) because the induced and suppressed features may exhibit different up- and down-tail characteristics in the joint distribution of basic attributes (Fig. 1). Equation (2) and the following derivations are general and can be applied to detect both up- and down-regulated features. Before we introduce the algorithm to find the parameters of *f*(∙) by trying to solve Eq. (2), we explain how to estimate FDR(***X***, *f*) in the following.

### FDR estimation

In practice, FDR(***X***, *f*) in Eq. (2) is unknown. To estimate the FDR of an arbitrary *f*(∙), we implemented the Storey framework [5] in a non-parametric fashion [10], which we briefly explain below for completeness. Let the NULL hypothesis of a feature be that it is not a DEF. Assuming there are *M* independent features. Table 1 lists the possible results when simultaneously testing *M* features for calling DEFs using *f*(∙), among which *R*(*f*) is an observable variable indicating the number of DEFs detected by *f*(∙) and *V*(*f*) is a hidden variable indicating the number of false DEFs detected by *f*(∙). Let *D*_*f*_ be the variable representing discriminant value calculated by *f*(∙). We can write down the FDR according to [38] as a function of *f*(∙):

3 $$ \begin{array}{c}\hfill \mathrm{F}\mathrm{D}\mathrm{R}(f)=E\left[\frac{V(f)}{R(f)}\kern0.45em \Big|R(f)>0\right]\mathrm{P}\left(R(f)>0\right)\hfill \\ {}\hfill =\mathrm{P}\left( NULL\Big|{D}_f>0,R(f)>0\right)\cdot \mathrm{P}\left(R(f)>0\right)\hfill \end{array} $$

**Table 1:**
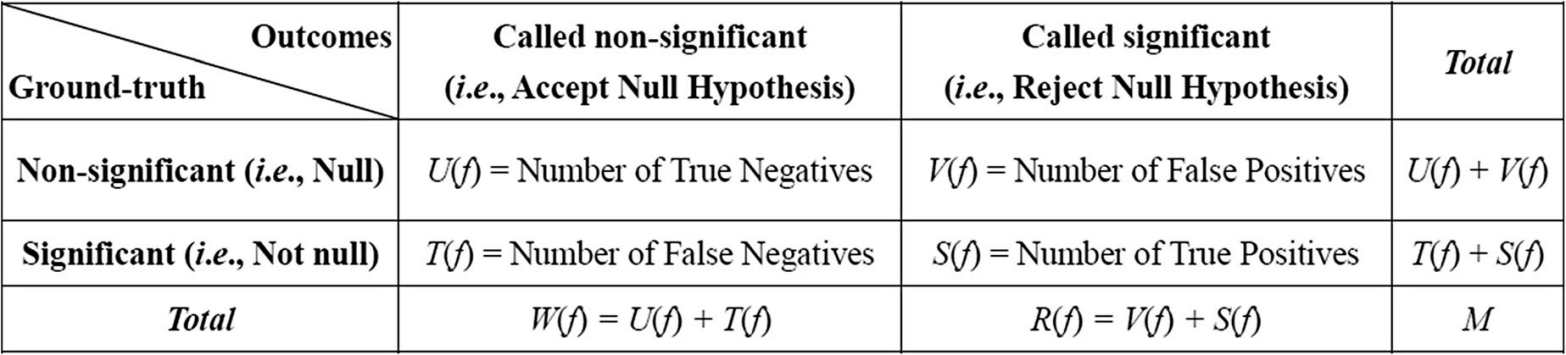
Outcomes when applying *f*(∙) to classifying *M* features into DEFs or non-DEFs

Equation (3) can be rewritten using the Bayes rule as the following:

4 $$ \begin{array}{c}\hfill \mathrm{F}\mathrm{D}\mathrm{R}(f)=\frac{\mathrm{P}(NULL)\cdot \mathrm{P}\left({D}_f>0\Big| NULL,R(f)>0\right)}{\mathrm{P}\left({D}_f>0\Big|R(f)>0\right)}\cdot \mathrm{P}\left(R(f)>0\right)\hfill \\ {}\hfill +\frac{\mathrm{P}(NULL)\cdot \mathrm{P}\left({D}_f>0\Big| NULL,R(f)=0\right)}{\mathrm{P}\left({D}_f>0\Big|R(f)>0\right)}\cdot \mathrm{P}\left(R(f)=0\right)\hfill \\ {}\hfill =\frac{\mathrm{P}(NULL)\cdot \mathrm{P}\left({D}_f>0\Big| NULL\right)}{\mathrm{P}\left({D}_f>0\Big|R(f)>0\right)}\hfill \end{array} $$

Equation (4) utilizes the fact that P(*D*_*f*_ > 0|*NULL*, *R*(*f*) = 0) = 0 because no hypothesis is rejected when *R*(*f*) = 0. Below we explain non-parametric methods for estimating P(*D*_*f*_ > 0|*NULL*), P(*NULL*), and P(*D*_*f*_ > 0|*R*(*f*) > 0) given a dataset ***X*** and a fixed *f*(∙).

**Estimate P**(***D***_***f***_ > 0|***NULL***). The term P(*D*_*f*_ > 0|*NULL*) is the probability of *D*_*f*_ > 0 when NULL is true. The distribution of *D*_*f*_ under NULL condition, depending on both the distributions of basic attributes and *f*(∙), can be extremely complex. Hence it may not feasible to determine this term in an analytical form. We therefore estimate P(*D*_*f*_ > 0|*NULL*) by adopting the non-parametric method developed in [10], which allows us to better explore the structure of data distribution in a data-dependent manner. This method randomly permutes the original dataset *B* times to generate the null control, and estimates P(*D*_*f*_ > 0|*NULL*) as:

5 $$ \widehat{\mathrm{P}}\left({D}_f>0\Big| NULL\right)=\frac{{\widehat{\mathbb{E}}}_b\left(\left|\left\{{d}_{i,b}^{*}>0\Big|{d}_{i,b}^{*}\in \mathbb{D}\left({\boldsymbol{X}}_b^{*},f\right)\right\}\right|\right)}{M} $$

where *d*_*i*,b_^*^ is the discriminant value of the *i*-th feature in the *b*-th (1 ≤ *b* ≤ *B*) permutation ***X***_b_^*^. The function ${\widehat{\mathbb{E}}}_b\left(\cdot \right)$ uses all *B* permutated datasets to estimate the expected number of non-DEFs that are incorrectly classified as DEFs. A reasonable choice of ${\widehat{\mathbb{E}}}_b\left(\cdot \right)$ is the median/mean function.

**Estimate P**(***NULL***). It is expected that P(*NULL*) ⋅ *M* features are non-DEFs (i.e., true NULL hypotheses). Below we use the *p*-value concept to explain how to estimate P(NULL) from data although we do not need to estimate *p*-values. Assuming that all features are independent, the *p*-values of the discriminant values of these P(*NULL*) ⋅ *M* features should be uniformly distributed between 0 and 1. Therefore, for some chosen *p*-value cutoff *λ* ∈ (0, 1), we should expect that there are (1 − *λ*) ⋅ P(*NULL*) ⋅ *M* non-DEFs whose *p*-values are greater than *λ*. Let *d*_*λ*_ denote the discriminant value whose *p*-value is *λ*. Since it is possible for some true DEFs to have *p*-values greater than *λ*, it is expected that $\left(1-\lambda \right)\cdot P(NULL)\cdot M\le \left|\left\{{d}_i\Big|{d}_i\le {d}_{\lambda },{d}_i\in \mathbb{D}\left(\boldsymbol{X},f\right)\right\}\right|$ when *M* is large enough and *λ* is well-chosen. In practice, *d*_*λ*_ can be estimated as the value smaller than *λ* percentile of elements in the permutation set ${\left\{\mathbb{D}\left({\boldsymbol{X}}_b^{*},f\right)\right\}}_{b=1\dots B}$. We can hence have a conservative estimation of P(*NULL*) as:

6 $$ \widehat{\mathrm{P}}(NULL)=\frac{\left|\left\{{d}_i\Big|{d}_i\le {d}_{\lambda },{d}_i\in \mathbb{D}\left(\boldsymbol{X},f\right)\right\}\right|}{\left(1-\lambda \right)\cdot M} $$

We conservatively set *λ* = 50% and truncate $\widehat{\mathrm{P}}(NULL)$ at 1 because a probability should never exceed 1.

**Estimate P**(***D***_***f***_ > 0|***R***(***f***) > 0). The probability P(*D*_*f*_ > 0|*R*(*f*) > 0) can be naturally estimated as:

7 $$ \widehat{\mathrm{P}}\left({D}_f>0\Big|R(f)>0\right)=\frac{\left|\left\{{d}_i\Big|{d}_i>0,{d}_i\in \mathbb{D}\left(\boldsymbol{X},f\right)\right\}\right|\vee 1}{M}=\frac{r\left(\boldsymbol{X},f\right)\vee 1}{M} $$

where $r\left(\boldsymbol{X},f\right)=\left|\left\{{d}_i\Big|{d}_i>0,{d}_i\in \mathbb{D}\left(\boldsymbol{X},f\right)\right\}\right|$ is an observed value of the variable *R*(*f*) given the dataset ***X***, and *r*(***X***, *f*) ∨ 1 = *r*(***X***, *f*) if *r*(***X***, *f*) > 0, otherwise 1. The term *r*(***X***, *f*) ∨ 1 prevents the estimated FDR from being undefined due to having 0 as the denominator. Plugging Eqs. (5–7) into Eq. (4), we have the estimated FDR as:

8 $$ {\widehat{\mathrm{FDR}}}_{\lambda}\left(\boldsymbol{X},f\right)=\frac{\left|\left\{{d}_i\Big|{d}_i\le {d}_{\lambda },{d}_i\in \mathbb{D}\left(\boldsymbol{X},f\right)\right\}\right|\cdot {\widehat{\mathbb{E}}}_b\left(\left|\left\{{d}_{i,b}^{*}>0\Big|{d}_{i,b}^{*}\in \mathbb{D}\left({\boldsymbol{X}}_b^{*},f\right)\right\}\right|\right)}{\left(1-\lambda \right)\cdot \left\{r\left(\boldsymbol{X},f\right)\vee 1\right\}\cdot M} $$

If the number of permutation is large enough, *r*(***X***, *f*) ∨ 1 will effectively set the estimated FDR as 0 when *r*(***X***, *f*) = 0 because, on expectation, the discriminant values of the permuted data are less significant than those of the original data. Thus we have ${\widehat{\mathbb{E}}}_b\left(\left|\left\{{d}_{i,b}^{*}>0\Big|{d}_{i,b}^{*}\in \mathbb{D}\left({\boldsymbol{X}}_b^{*},f\right)\right\}\right|\right)/M\le \left|\left\{\left.{d}_i\right|{d}_i>0,{d}_i\in \mathbb{D}\left(\boldsymbol{X},f\right)\right\}\right|/M=r\left(\boldsymbol{X},f\right)/M=0$, which makes ${\widehat{\mathrm{FDR}}}_{\lambda}\left(\boldsymbol{X},f\right)=0$.

### Discriminant-Cut algorithm

As a simple start to implement Eq. (2), we chose the discriminant function *f*(∙) from the linear function family $f\left({s}_1,\dots, {s}_K\right)={\displaystyle \sum_{i=1}^K}{w}_i{s}_i-\tau$, subject to $\left|{\displaystyle \sum_{i=1}^K}{w}_i\right|=1$, where {*w*_*i*_} and *τ* are the unknown parameters of *f*(∙) to be learned from ***X***. We further require *w*_*i*_ ≥ 0 when detecting up-regulated DEFs and *w*_*i*_ ≤ 0 when detecting down-regulated DEFs, which effectively make $\left|{\displaystyle \sum_{i=1}^K}{w}_i\right|={\displaystyle \sum_{i=1}^K}\left|{w}_i\right|=1$ a L_1_ regularization that tends to yield sparse models. A simple algorithm, Discriminant-Cut (DC), was designed and implemented to search for the “ideal” f*(⋅). DC performs an exhaustive search at an empirically decided resolution (Additional file 1: Algorithm S1). The algorithm first populates a set of {*w*_*i*_} candidates, and for each of them, tunes *τ* to detect as many DEFs as possible while keeping the estimated FDRs under controlled by a user-desired threshold Ψ. Since both finding f*(⋅) and estimating FDR using the same permutation set, it is possible that the final estimated FDR is biased. To address this, we referred the idea in [39]. After choosing f*(⋅), we calibrate its cutoff *τ* using another large independent permutation set, and then apply the recalibrated f*(⋅) to identify DEFs. The efficiency of the search was greatly improved by sorting intermediate results to facilitate quick search, binary search, and avoiding unnecessary exploration (details in Additional file 1: Section 1.1). The algorithm runs fast in practice. In our experiments, most of the runtime was spent on computing basic attributes, and the remaining computations took almost negligible time.

There are approaches for linearly combining multiple attributes (or statistics) from either dependent or independent datasets [39–41] (and the references therein). Some of them mainly explore the covariance between attributes. Some aim to minimize the *p*-values of individual features by allowing each feature to has its own combination setting. Our approach does not make any assumption about the joint distribution of the attributes. We try to maximally explore differential expression information in one dataset, and force all features to share the same {*w*_*i*_}. In addition, our objective function explicitly models the overall goal – maximize detections constrained by a target FDR. In the future, it may worth exploring how minimizing the *p*-values of individual features can benefit our goal.

## Results

### RNA-seq simulation test

We firstly carried out a series simulation tests, in which the ground truths were known to ensure proper comparison, to assess the advantages of combining multiple basic attributes by DC. We let DC use up to three representative basic attributes: (1) *s* ^*T*^ – the moderated *t*-statistic from voom, (2) *s* ^*R*^ – the corrected ranksum statistic from SAM (this is different from SAMseq’s ranksum statistic that is adjusted for different library sizes by resampling), and (3) *s* ^*NB*^ – the Wald statistic for NB-based differential expression test from DESeq2. This produced seven DC configurations: DC^*T*^ (DC using *s* ^*T*^), DC^*R*^ (DC using *s* ^*R*^), DC^*NB*^ (DC using *s* ^*NB*^), DC^*T*+*R*^ (DC using *s* ^*T*^ and *s* ^*R*^), DC^*R*+*NB*^ (DC using *s* ^*R*^ and *s* ^*NB*^), DC^*T*+*NB*^ (DC using *s* ^*T*^ and *s* ^*NB*^), and DC^*T*+*R*+*NB*^ (DC using *s* ^*T*^, *s* ^*R*^ and *s* ^*NB*^). We also compared DC with 13 other RNA-seq differential expression analysis methods including baySeq, DESeq, EBSeq, edgeR, NBPSeq, SAMseq, ShrinkSeq, TSPM, voom, vst/limma, PoissonSeq, DESeq2, and ODP.

#### Simulation design

**Fig. 2:**
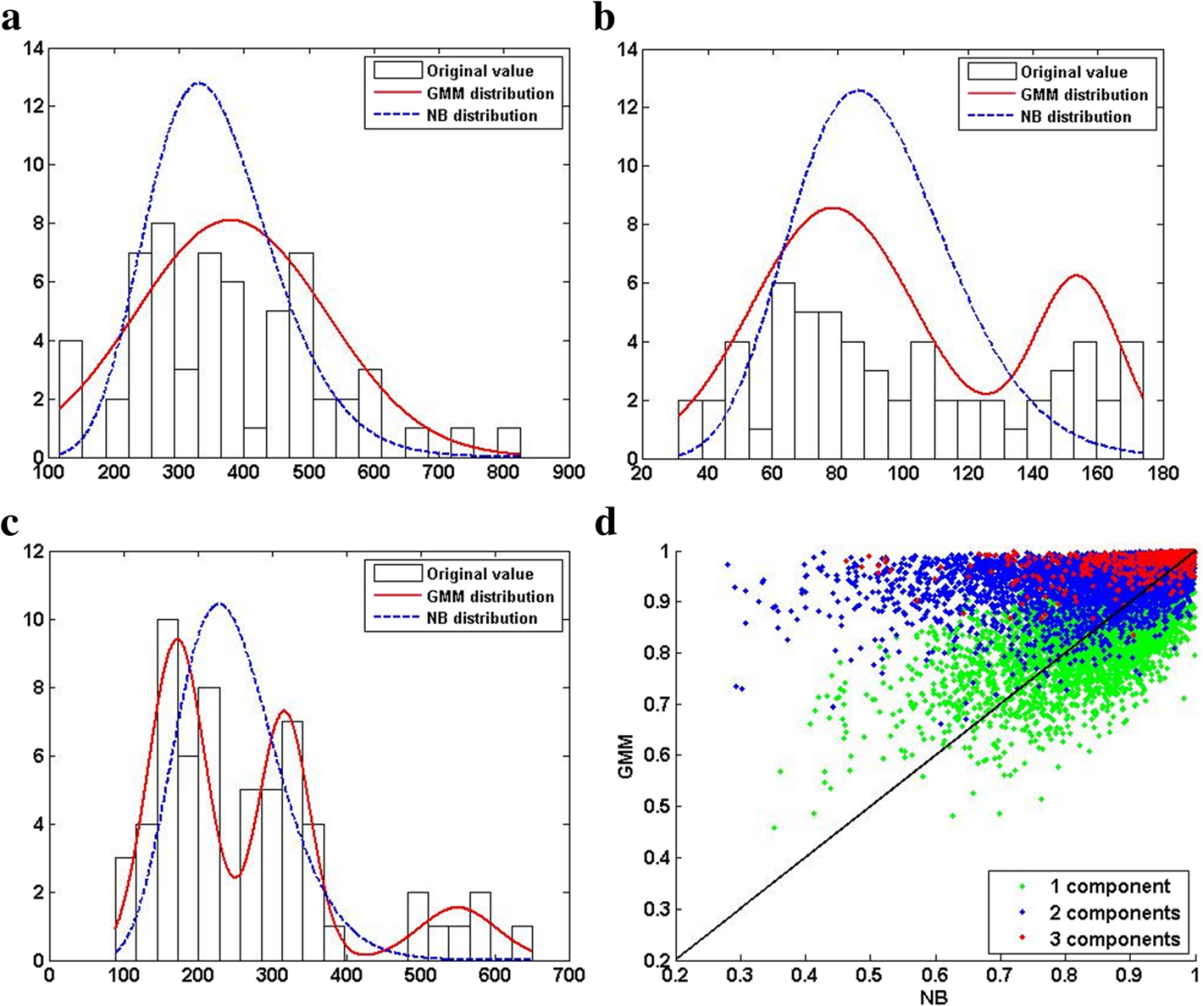
The Montgomery dataset shows that real RNA-seq datasets contain complex distributions. (**a** Gene ID: 64928), (**b** Gene ID: 11244), and (**c** Gene ID: 80169) show the distributions of three genes as examples. The *white bars* represent the histogram of the original data. The *solid-red* and *dashed-blue curves* represent the distributions of the fitted GMM and NB, respectively. See main text for the details of fitting NB and GMM to the original data. The number of Gaussian mixtures are 1, 2, and 3 in (**a**), (**b**), and (**c**), respectively. GMM is better than NB at representing distributions with multiple modes. (**d** Compare correlation coefficients) *Y*-axis: the correlation coefficients between the read-count distributions and the corresponding fitted GMM distribution. *X*-axis: the correlation coefficients between the read-count distributions and the corresponding fitted NB distribution. Each *dot* represents a gene. The distribution of a gene’s read-counts is approximated by a histogram of 20 equal-size bins spanning the read-count value range. The color*s* of dots indicate the most proper numbers of components in a fitted GMM according to the Bayesian Information Criterion: *green* (~44%), *blue* (~50%), and *red* (~6%) correspond to *1*, *2* and *3* components, respectively. About 63.5% of genes are above the *diagonal line* indicating their distributions are more GMM-like. The distributions of the remaining ~36.5% genes are more NB-like. To further investigate this observation, we calculated *N*_*NB*_^*GMM*^ as the number of genes whose advantages of their GMM fits over their NB fits are significant (*p*-value < 0.05) if the distributions of all genes are NBs. If all genes are indeed governed by NBs, *N*_*NB*_^*GMM*^ should be close to the expected number that is 11,573 × 0.05 ≈ 579. We sampled 2000 datasets from the NB fit of each gene, each of which contain 60 samples. For each dataset, we fit a GMM and a NB, and calculated the difference between their fitting scores (i.e., GMM fit score – NB fit score). The score differences across all datasets were collected to approximate the NULL distribution and calculate the *p*-value of the score difference between the GMM and NB fits to the original samples. We got *N*_*NB*_^*GMM*^ = 2442 (> > 579), 1830 of which have 2+ components in their GMMs. Hence we can deduce that the distributions of a substantial number of genes are not NB-like. In a similar way, we calculated *N*_*GMM*_^*NB*^ as the number of genes whose advantages of their NB fits over their GMM fits are significant (*p*-value < 0.05) if the distributions of all genes are GMM. We obtained *N*_*GMM*_^*NB*^ = 2431 (>> 579) indicating that the distributions of a substantial number of genes are not GMM-like. Putting the above together, we conclude that neither NB nor GMM dominates the distributions of genes in the Montgomery dataset

To make the simulation tests as realistic as possible, we simulated the test datasets based on a real RNA-seq dataset – the Montgomery dataset (downloaded from http://bioinf.wehi.edu.au/PublicDatasets/ as of Apr.15^th^, 2015) [42], which contains the transcriptome of 25,702 genes in 60 extended HapMap individuals of European descent. Large number of samples in this dataset allows us to reveal that the distributions in real datasets can be indeed much more complex than often assumed. Nevertheless, the number of replicates in each simulated dataset is much smaller and is within the range of common practice. We first removed genes with extremely low expression profiles (read counts below 10 in more than half of the replicates). For each of the remaining 11,573 genes, we decided whether its read counts could be better modeled by a NB distribution or a Gaussian mixture model (GMM) in the following way. The NB and GMM distributions were estimated by using DESeq2 implemented in R and the statistics toolbox of MATLAB R2013a, respectively. The most proper number of components in a GMM was decided based on the Bayesian Information Criterion. The GMMs of ~44, ~50, and ~6% genes contained 1, 2, and 3 components, respectively. Figure 2a–c show a few typical examples. Then, for each gene, we calculated the correlation between the histograms of its read-counts and the corresponding fitted NB/GMM to decide which distribution was a better fit. The GMMs were truncated at zero because read counts should be non-negative. The distributions of about 63.5 and 36.5% of genes can be better represented by GMM and NB (Fig. 2d), respectively. A simple experiment presented in Fig. 2d caption validates that the distributions of many genes in this dataset are more complex than what assumed conventionally (e.g., NB or Gaussian). Our choice of examining correlation between the histogram of data and its fits was based on two considerations: (a) histogram is commonly used in practice to approximate distributions, and (b) correlation is a widely adopted distance metric. This method is mainly used to show that features have complex patterns of distributions rather than as a rigorous model selection method for determining the exact ratio of GMM to NB, such as the one (63.5 vs. 36.5%) shown above. We consider it sufficient for choosing distributions, which roughly approximate the original ones, for generating data in the following simulation test.

In each simulation test run for comparing the chosen RNA-seq differential expression analysis methods, we simulated *N* read-counts for every gene using the distribution (either NB or GMM) decided to be better in the above way, and randomly divided the simulated read-counts into two equal-size groups to obtain true non-DEFs. The simulation of a gene was repeated until its logarithmic fold-change was not larger than 4.5*σ*_*N*_, where *σ*_*N*_ is the standard deviation of the logarithmic fold-change between two *N*-sample groups randomly chosen from the Montgomery dataset. The 4.5*σ*_*N*_ fold-change threshold was chosen because we observed in the Montgomery dataset that the expected number of fold-changes higher than 4.5*σ*_*N*_ is below 0.05. Then we randomly made *G*_*b*_^*a*^ genes (*a* and *b* are the numbers of up- and down-regulated genes, respectively) as true DEFs in the following way. For each of the chosen genes, we multiplied or divided one of its groups by a factor uniformly sampled between 1.5 and 3.0 to provide a reasonable wide range of differences in expression. Finally, all simulated values were rounded to their nearest integers.

A series of simulation test runs were conducted under 20 different settings: 5 different sample sizes (*N* = 8 [4 vs. 4], 10 [5 vs. 5], 12 [6 vs. 6], 16 [8 vs. 8], and 20 [10 vs. 10]) × 4 different true DEF configurations (*G*_400_^400^, *G*_500_^500^, *G*_600_^600^, and *G*_0_^1000^). At each of the 20 simulation settings, we ran the test 100 times and recorded the results. Our comparisons focus on two key performance factors: (1) the effectiveness of FDR control, namely whether the real FDR is effectively bounded by the target FDR; and (2) the detection power, namely the ability to detect as many true DEFs as possible without violating (1).

#### Integrating multiple basic attributes helps substantially

**Fig. 3:**
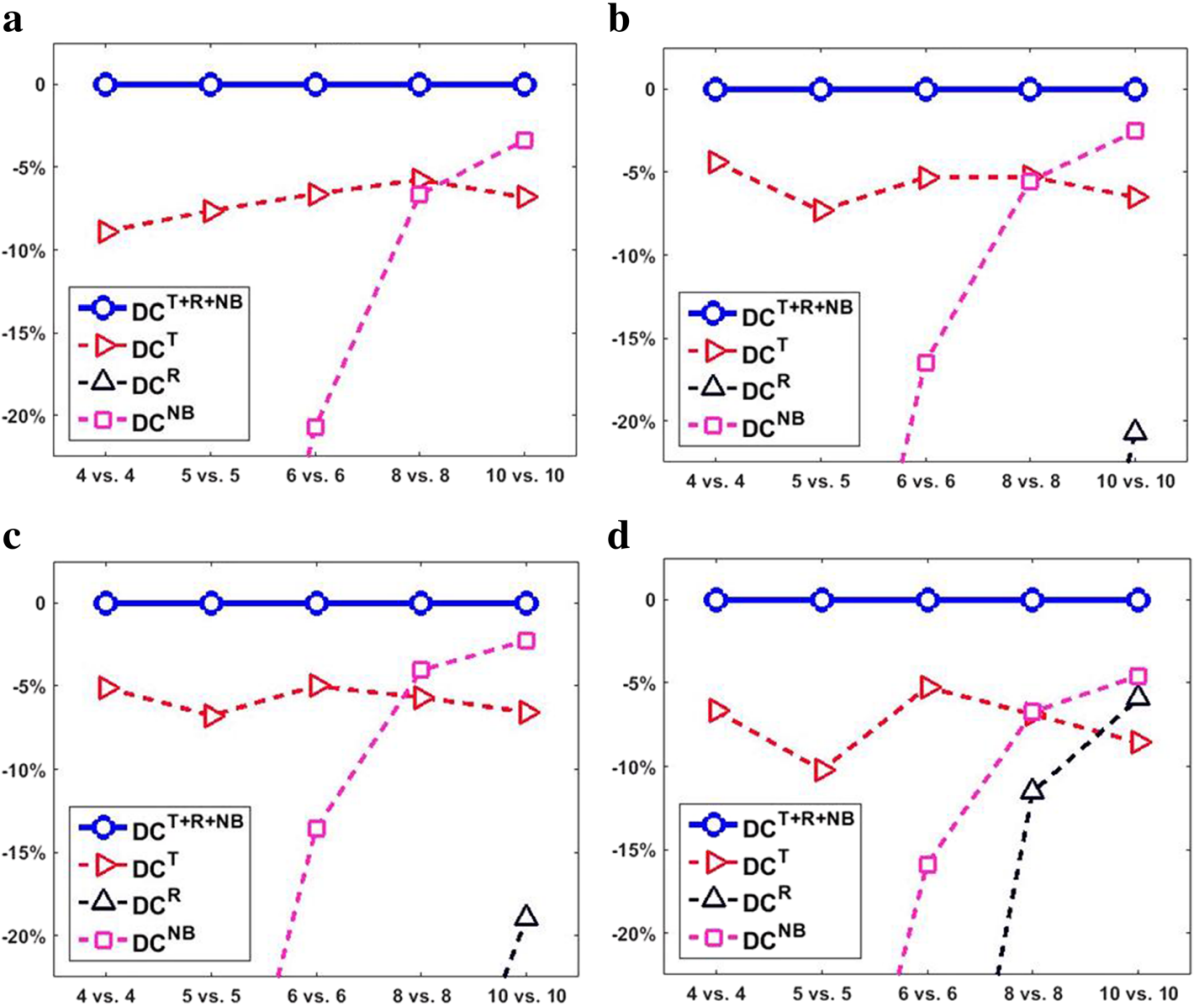
Compares the true DEFs detected by different DC configurations. Four DC configurations (DC^*T*^, DC^*R*^, DC^*NB*^ and DC^*T*+*R*+*NB*^) are compared under different sample sizes (*x*-axis) and different true DEF configurations: (**a**) *G*_400_^400^, (**b**) *G*_500_^500^, (**c**) *G*_600_^600^, and (**d**) *G*_0_^1000^. The target FDR cutoff is 0.05. The *y*-axis indicates the relative differences (in parentage) of the average true DEFs detected by different DC configurations with respect to those detected by DC^*T*+*R*+*NB*^.The plots only display up to 20% relative difference. This figure clearly shows that DC^*T*+*R*+*NB*^ outperformed the remaining DC configurations

Comparing the results of different DC configurations shows that the advantage of integrating multiple basic attributes in detecting DEFs is significant. Figure 3 shows that DC^*T*+*R*+*NB*^ consistently outperformed the three single-attribute DC configurations under all 20 simulation test settings (5 sample sizes × 4 DEF configurations), and single-attribute DC methods (DC^*T*^, DC^*R*^, DC^*NB*^) significantly underperformed the multi-attribute ones. Here we use the results of a typical simulation test setting (6 vs. 6 and *G*_500_^500^) as an example. Even though some individual attributes alone may be inferior to other attributes in detecting DEFs, they can indeed provide substantial enhancements to other attributes. For example, in Table 2, DC^*R*^ detected no DEFs at FDR < 0.01 or FDR < 0.05. Adding *s* ^*R*^ to *s* ^*NB*^ significantly improved the results by 16.35% (paired *t*-test *p*-value = 8.87e-30) at FDR < 0.01 and by 9.62% (paired *t*-test *p*-value = 2.32e-35) at FDR < 0.05. Results across different sample sizes (Table 3) confirm the advantages of integrating multiple basic attributes. Grouping the DEFs detected by DC^*T*^, DC^*NB*^, and DC^*T*+*R*+*NB*^ accordingly to their distribution categories (Table 4), we observe that integrating multiple basic attributes helps to detect DEFs across the whole distribution spectrum. Interestingly, DC^*T*^ on average detected more DEFs governed by NB distributions than DC^*NB*^, which to some extent resonates with the idea of voom, i.e., it is sometimes more important to model the mean-variance relationship correctly than to design the exact distribution of read-counts.

**Table 2:** Compares the results of different DC configurations in a typical simulation test (6 vs. 6; *G*_500_^500^)

Baseline	Target FDR	Add s^*T*^	Add s^*R*^	Add s^*NB*^	Use s^*T*^_,_ s^*R*^, and s^*NB*^
Use s^*T*^ alone	0.01	-	+4.62% (1.84e-08)	+2.84% (8.74e-06)	+5.52% (2.18e-12)
	0.05	-	+2.32% (2.04e-14)	+3.71% (1.06e-23)	+5.31% (3.18e-35)
Use s^*NB*^ alone	0.01	+24.48% (1.98e-41)	+16.35% (8.87e-30)	-	+27.72% (8.76e-44)
	0.05	+14.71% (1.23e-51)	+9.62% (2.32e-35)	-	+16.48% (8.96e-56)

The 1^st^ column lists the single-attribute DC configurations. DC^*R*^ was not displayed because it failed to detect any true DEFs under both FDR targets. The 2^nd^ column lists the target FDR levels (0.01 or 0.05) at which performances are compared. Cells in the 3^rd^~6^th^ columns show the improvements in percentage of multi-attribute DC configurations (indicated by the column headers) over single-attribute DC configurations (indicated by the 1^st^ cells in the corresponding rows). The numbers in parentheses are the paired *t*-test *p*-values showing the significance of the improvement. For example, the cell at the 3^rd^ column and 4^th^ row shows that DC^*T*+*NB*^ outperformed DC^*NB*^ by 24.48% with a paired *t*-test *p*-value of 1.98e-41 at FDR < 0.01. Although DC^*R*^ as a single attribute failed detect any DEFs, adding s^*R*^ to the other two attributes (s^*T*^ and s^*NB*^) significantly improved the performance, as indicated by the 4^th^ column

**Table 3:**
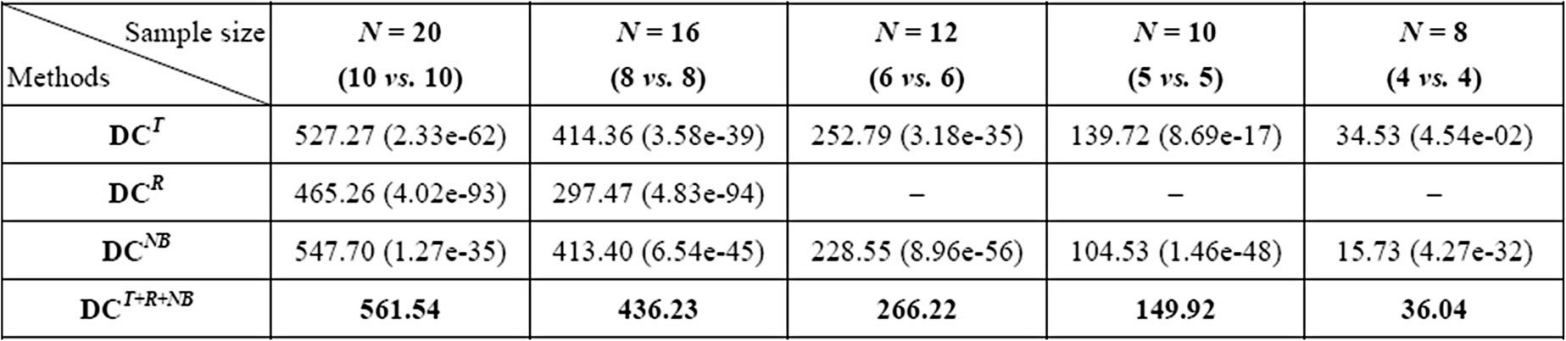
Compares the results of different DC configurations across different sample sizes (FDR < 0.05, *G*_500_^500^) in simulation tests

Compares DC^*T*+*R*+*NB*^ with three single-attribute DC configurations on simulated datasets of various sample sizes at FDR < 0.05 and *G*_500_^500^. Each cell shows the average number of true DEFs detected by a DC configuration under a sample size indicated by the column header. The numbers in parentheses are the paired *t*-test *p*-values indicating how significant DC^*T*+*R*+*NB*^ outperformed the corresponding single-attribute DC configurations under the same simulation test settings. DC^*R*^ detected no DEFs when *N* < 16.

**Table 4:** Compares the average numbers of true DEFs identified in different distribution categories

Detected by	Total	NB	Gaussian	GMM (2 or 3 components)
DC^*T*^	252.79 (3.18e-35)	115.60 (3.16e-27)	55.83 (8.12e-10)	81.36 (2.24e-21)
DC^*NB*^	228.55 (8.96e-56)	104.16 (7.39e-55)	48.33 (7.87e-39)	76.06 (7.01e-35)
DC^*T*+*R*+*NB*^	266.22	121.88	57.54	86.80

Compares the average numbers of true DEFs detected by DC^*T*^, DC^*NB*^, and DC^*T*+*R*+*NB*^ in different distribution categories under the simulation test setting: 6 vs. 6, *G*_500_^500^, and the target FDR < 0.05. DC^*R*^ is not displayed because it detected no DEFs. The numbers in the parentheses are the paired *t*-test *p*-values indicating how significant DC^*T*+*R*+*NB*^ outperformed the corresponding single-attribute DC configurations.

#### No single basic attribute dominates

We also observed that none of the basic attributes consistently performed better than other basic attributes in our simulation tests, which resonates the idea of utilizing multiple attributes. For example in Table 3, under the simulation test setting 10 vs. 10 and *G*_500_^500^, DC^*T*^ on average detected more true DEFs than DC^*NB*^ (370.79 vs. 359.01) at FDR < 0.01, but performed worse than DC^*NB*^ (527.27 vs. 547.70) at FDR < 0.05. Moreover, at FDR < 0.05, DC^*T*^ outperformed DC^*NB*^ on datasets when the sample size was relatively small (e.g., 4 vs. 4, 5 vs. 5 and 6 vs. 6) while DC^*NB*^ outperformed DC^*T*^ when the sample size was larger (e.g., 10 vs. 10). Interestingly, although DC^*R*^ underperformed DC^*T*^ under most test settings, DC^*R*^ outperformed DC^*T*^ under the setting of 10 vs. 10, *G*_0_^1000^ and target FDR < 0.05 (true DEFs: 555.63 by DC^*R*^ vs. 542.29 by DC^*T*^).

#### Compare DC^*T*+*R*+*NB*^ with other DEF detection approaches

We compared DC^*T*+*R*+*NB*^ and 13 other RNA-seq differential expression analysis methods including baySeq, DESeq, EBSeq, edgeR, NBPSeq, SAMseq, ShrinkSeq, TSPM, voom, vst/limma, PoissonSeq, DESeq2, and ODP. Figure 4 shows the average numbers of the detected true DEFs at two typical target FDR levels (0.01 and 0.05) under a typical simulation test setting 6 vs. 6 and *G*_500_^500^ (the results of the rest test settings are provided in Additional file 1: Figures S1–S20). Among those able to effectively control the FDR, DC^*T*+*R*+*NB*^ in general performed the best. At target FDR < 0.01, DC^*T*+*R*+*NB*^ on average detected 99.44 true DEFs, which is significantly better (paired *t*-test *p*-value = 1.79e-66) than the 31.59 true DEFs detected by the best non-DC method (vst/limma). At target FDR < 0.05, DC^*T*+*R*+*NB*^ detected 266.22 true DEFs, which is significantly better (paired *t*-test *p*-value = 1.32e-71) than the 204.59 true DEFs detected by the best non-DC method (vst/limma). Figure 5 compares the average number of true positives detected by different approaches at different target FDR cutoffs (from 0.01 to 0.1 with a step of 0.01) under a typical simulation test setting of 6 vs. 6 and *G*_500_^500^ (the results of other test settings are provided in Additional file 1: Figures S21–40). Figure 5 and Additional file 1: Figures S21–40 show that DC in general performed the best among those effectively controlled FDR.

**Fig. 4:**
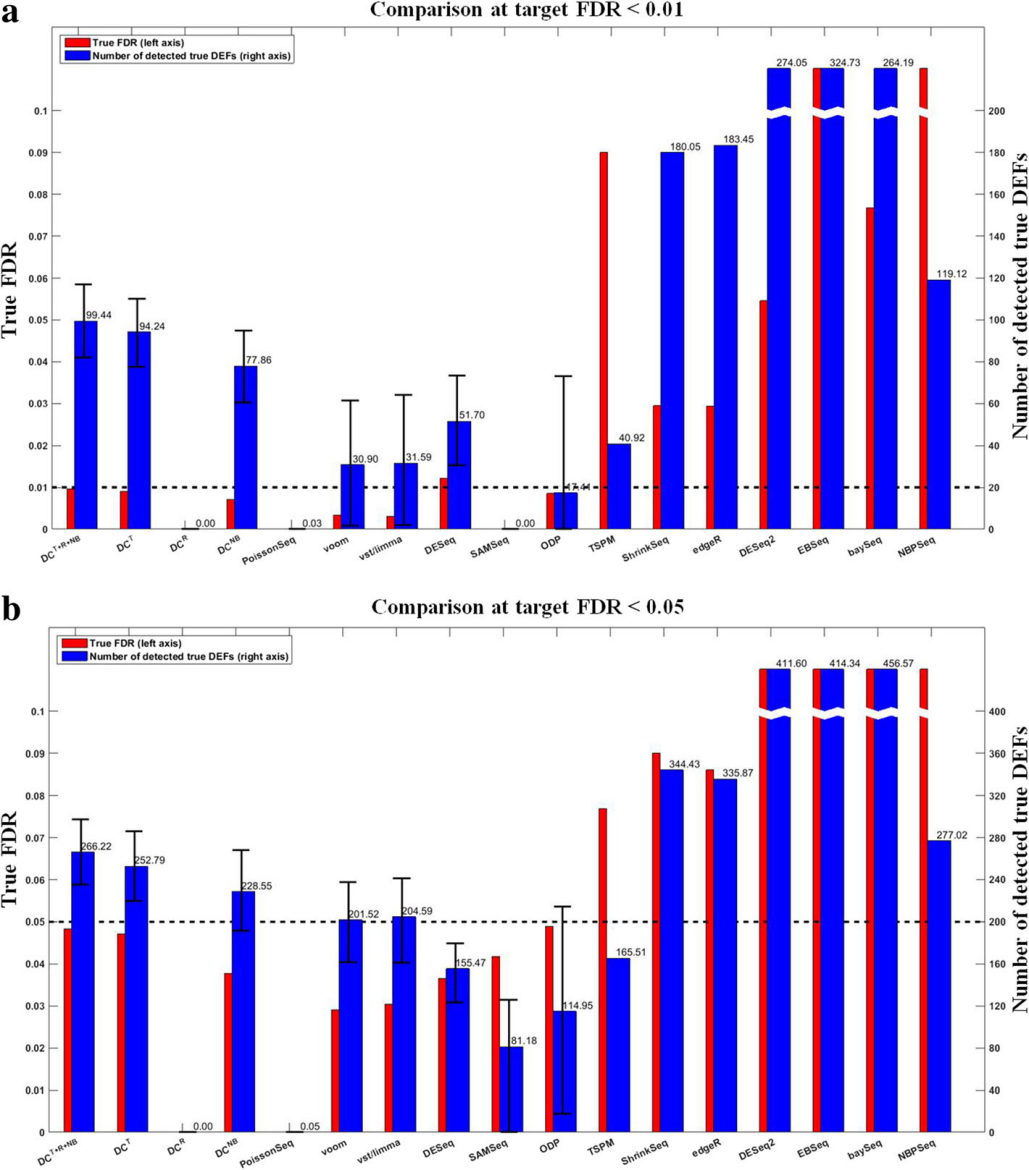
Evaluates RNA-seq differential expression analysis methods using simulated data (6 vs. 6; *G*_500_^500^). Methods are listed along the ***x***-axis. The *red bars* indicate the average true FDRs (refer to the left *y*-axis). The *horizontal dashed line* across the figure marks the target FDR. The *blue bars* indicate the average number of the detected true DEFs (refer to the right *y*-axis). The 90% confidence intervals of the detected DEFs are marked except for those whose true FDRs exceed the target FDR by 10%. **a** target FDR < 0.01. **b** target FDR < 0.05. The true FDRs of DC^*T*+*R*+*NB*^ do not exceed the corresponding target FDRs. DC^*T*+*R*+*NB*^ was the most powerful among those able to effectively control the FDR (see main text for more discussions)

**Fig. 5:**
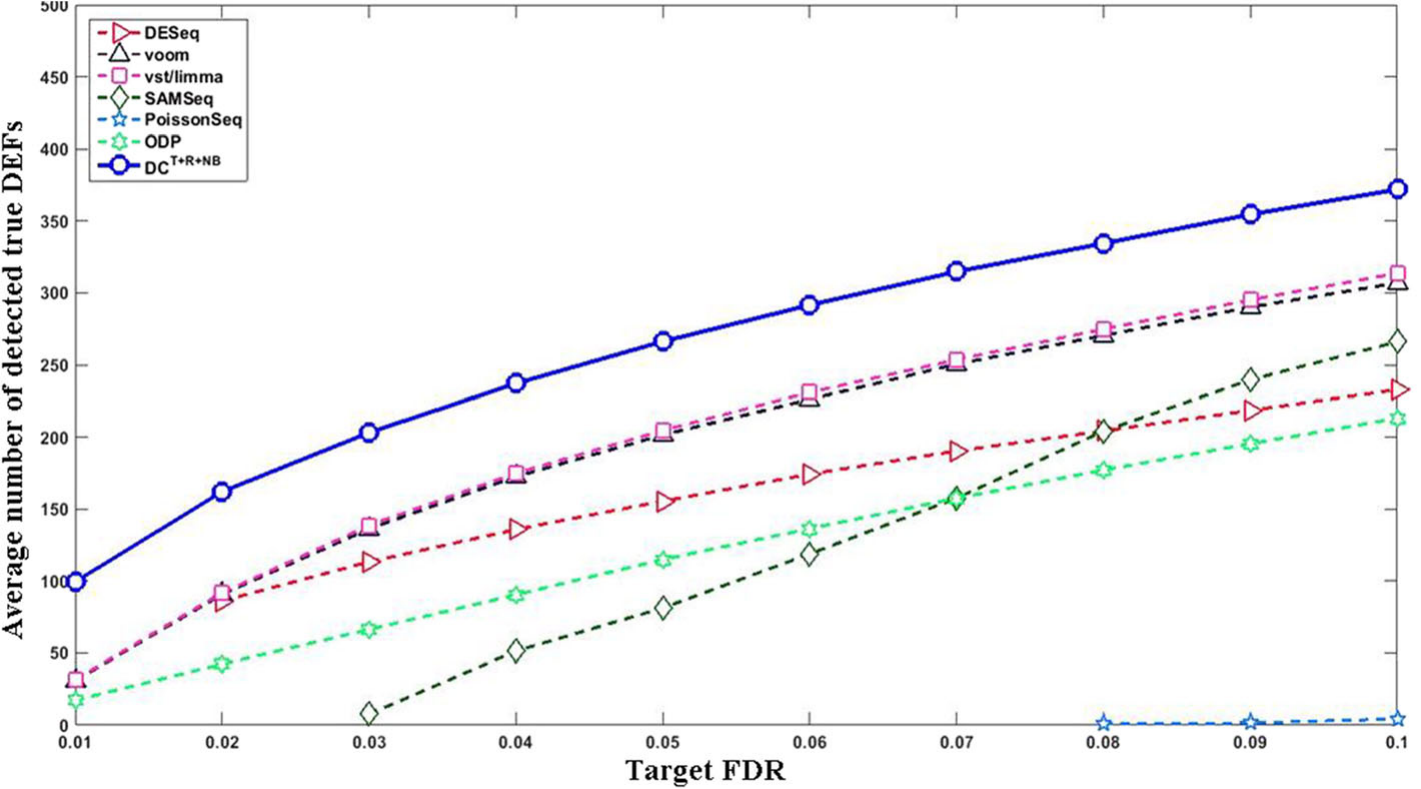
Compare the curves of the true positives vs. the target FDR (6 vs. 6 and *G*_500_^500^). The *x*- and *y*- axes indicate the target FDR cutoff and the average number of true positives, respectively. The *solid curve* with *blue circle* markers represents DC^*T*+*R*+*NB*^ and other curves represent non-DC methods. The result of a method at a particular target FDR is shown in this plot if (1) its average true FDR does not exceed the target FDR by 10%; and (2) its average number of true DEFs is ≥0.5 (rounds up to 1). DC was able to meet all target FDR cutoffs. The results of voom and vst/limma are almost the same

**Fig. 6:**
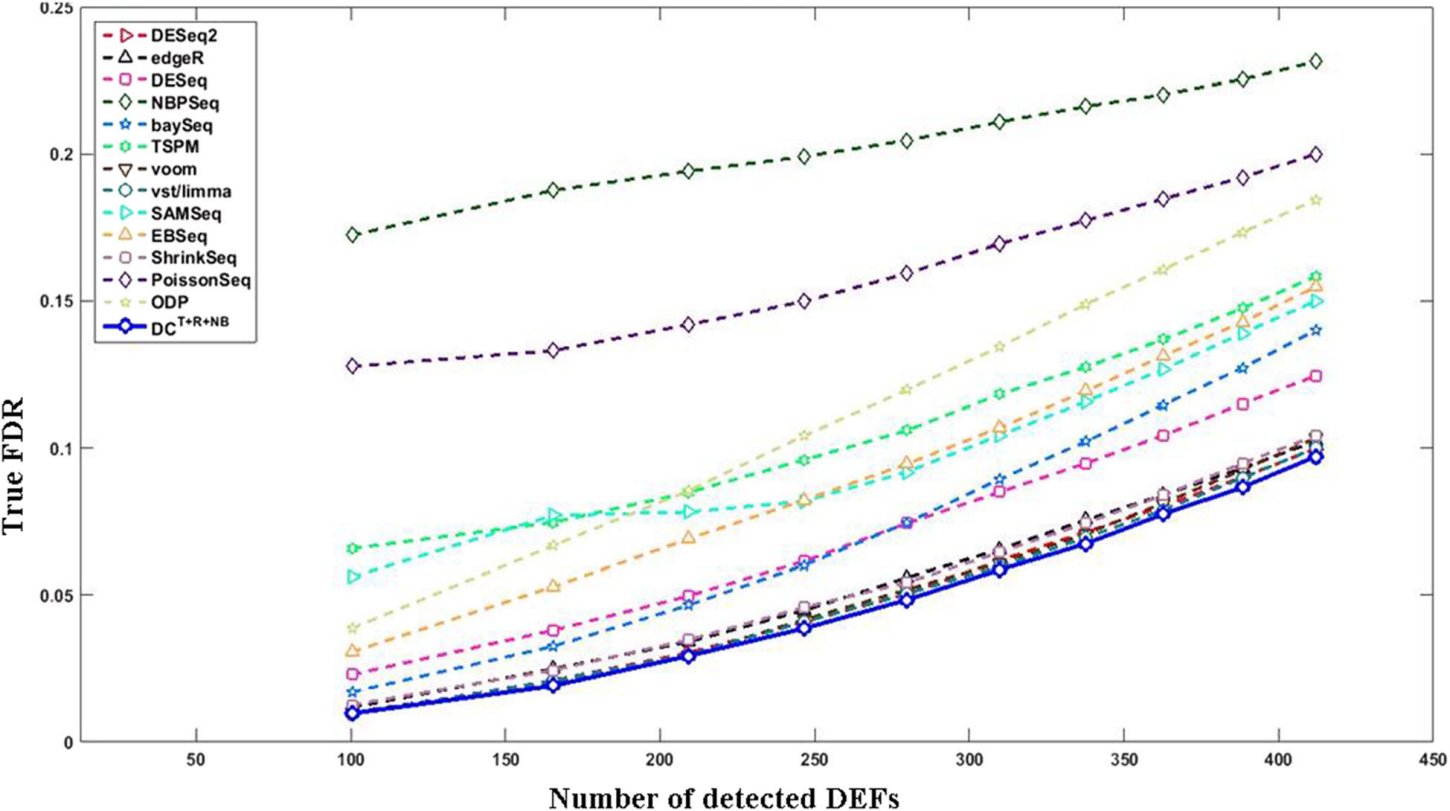
The curves of the true FDR vs. the number of detected DEFs in a typical simulation test (6 vs. 6; *G*_500_^500^). The *x*- and *y*- axes indicate the number of detected DEFs and the average true FDR, respectively. The curve of DC^*T*+*R*+*NB*^ (*solid curve* with *blue circle* markers) in this figure were converted from the results obtained by setting the target FDR between 0.01 and 0.1 with an increasing step of 0.01. The curves of other methods were obtained by letting them call the same number of DEFs detected by DC^*T*+*R*+*NB*^ at each target FDR

In some application scenarios other than ours, users may want to choose a fixed number of top DEFs. To serve this purpose, Fig. 6 compares the results using FDC (false discovery curve: true FDR *vs* number of detected DEFs). The FDCs of other test settings are provided in Additional file 1: Figures S41–60. Figure 6 and Additional file 1: Figures S41–60 show that DC is among the best performers including voom, vst/limma, DESeq2, edgeR, and ShrinkSeq. Here we do not show ROC (false positive rate vs. true positive rate), which is also popular for evaluating machine learning techniques and statistical analysis methods, because FDC and ROC deliver the same information from different viewpoints. Since true FDR can be estimated but usually unknown, FDC and ROC should be used with caution in our application scenario because they do not consider whether a method is able to estimate FDR well. FDC and ROC only depend on the ranks of features’ significance scores regardless of their actual values. Therefore, it is possible that two DEF detection methods can produce the same ROC/FDC although they have quite different capabilities in estimating FDR. Imagining there are two DEF detection methods. The 1^st^ method is biased towards high *p*-values (i.e., it tends to generate very high *p*-values for all features) because it imposes some assumptions. Calling one single significant feature using the 1^st^ method will lead to an extraordinarily high estimated FDR. On the contrary, the 2^nd^ method is biased towards small *p*-values (i.e., it tends to generate very low *p*-values for all features) because it imposes other assumptions. Given a target FDR, the 2^nd^ method will dramatically underestimate its true FDR and call too many false positives. Nevertheless, if the features are ranked in the same order by both methods, they will produce exactly the same ROC/FDC.

#### Effects of sample size and DEF configuration

**Fig. 7:**
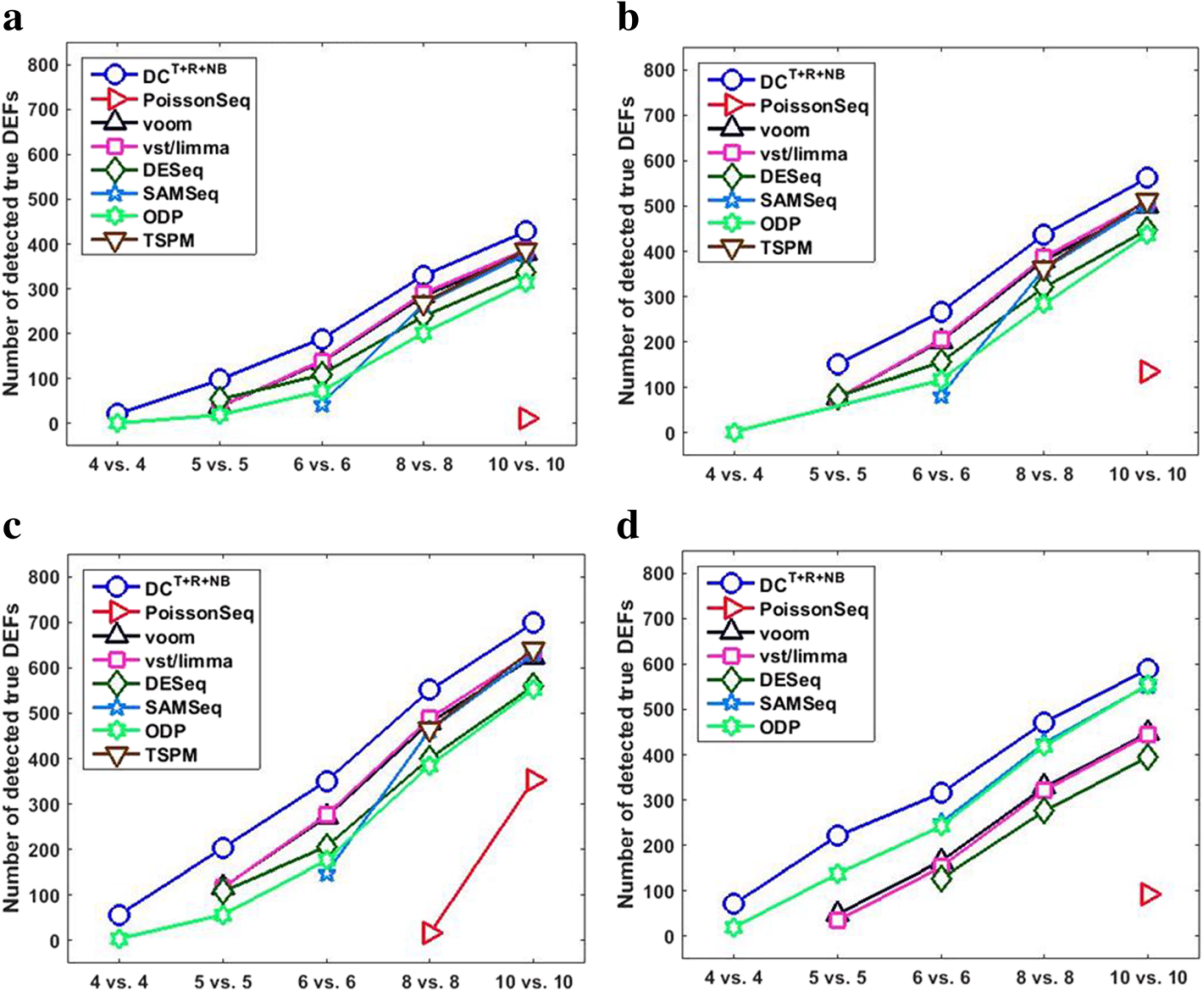
Compares DEF detection results under different test settings at target FDR < 0.05. The *x*- and *y*- axes indicate the sample size and the number of detected true DEFs, respectively. Plots (**a** *G*_400_^400^), (**b** *G*_500_^500^), (**c** *G*_600_^600^), and (**d** *G*_0_^1000^) are the results of DEF configurations *G*_400_^400^, *G*_500_^500^, *G*_600_^600^ and *G*_0_^1000^, respectively. A method is not displayed under a test setting if either its corresponding true FDR exceeds the target FDR by 10% or it on average detected less than 0.5 true DEF

Figure 7 summarizes the effects of “sample sizes + DEF configurations” on DEF detection results at target FDR < 0.05. The result of a method under a particular setting is included if its true FDR does not exceed the target FDR by 10% and it detects on average at least 0.5 true DEFs (rounds up to 1). Under most settings, DC^*T*+*R*+*NB*^ was able to effectively control FDR and detect more DEFs. However, when the sample size is small (4 vs. 4), the average true FDRs of DC^*T*+*R*+*NB*^ were 0.053 and 0.058 for *G*_400_^400^ and *G*_500_^500^, respectively; and ODP was the only method able to detect true DEFs (1.11 under *G*_400_^400^ and 2.57 under *G*_500_^500^) while meeting the FDR target. When the sample size was decreased, all methods detected less DEFs, and it was more difficult to control the FDR, especially when a more stringent target FDR was imposed. For example, when *N* = 8 (4 vs. 4), *G*_500_^500^, and the target FDR < 0.01, DC^*T*+*R*+*NB*^ on average detected less than 20 DEFs, and one single false positive alone would increase its true FDR by 0.05, which is much higher than the target FDR. Smaller sample sizes (2 vs. 2 and 3 vs. 3) were also tested. However, no method was able to control FDR well (i.e., their true FDRs > 110% × the target FDR) or detect at least 0.5 DEFs on average. Hence, the results of 2 vs. 2 and 3 vs. 3 are not shown in Fig. 7. This indicates that it remains challenging to detect DEFs governed by complex distributions when the sample size is small.

### Evaluation using the SEQC/MAQC-III dataset

The US Food and Drug Administration has coordinated a large-scale community effort, the Sequencing Quality Control project (SEQC/MAQC-III), to assess the performance of RNA-seq across laboratories and to test different sequencing platforms and data analysis pipelines [43]. The consortium has generated a RNA-seq datasets (Gene Expression Omnibus accession code: GSE47792) from two reference RNA samples, the Strategene Universal Human Reference RNA (sample A) and the Ambion Human Brain Reference RNA (sample B). This dataset contains two reference feature subsets: (1) 92 synthetic RNAs from the External RNA Control Consortium (i.e., ERCC spike-ins) with four different sample A/sample B ratios (1/2, 2/3, 1 and 4); and (2) ~1000 genes whose sample A/sample B fold-changes were validated using TaqMan qRT-PCR [44]. In the following comparison, we used log2 expression change threshold of 0.5 to select true DEFs from the ~1000 TaqMan qRT-PCR validated genes, and obtained 693 genes denoted as the positive TaqMan genes below. However, due to the extreme difference between samples A and B [45], the positive TaqMan genes only represent a small fraction of those differentially expressed between samples A and B. If we let different DEF detection methods compare the replicates of samples A and those of samples B, their results on the positive ERCC spike-ins and the positive TaqMan genes cannot accurately reflect their overall performances. In addition, all DEF detection methods will detect too many DEFs that dwarf the differences between their detection results. Thus we designed the following procedure to make the positive ERCC spike-ins and the positive TaqMan genes together as a proper reference feature set for evaluating DEF detection methods.

We focused on the SEQC/MAQC-III RNA-seq subset sequenced at the Australian Genome Research Facility using the Illumina HiSeq200, in which each RNA sample has 64 technical replicates (4 libraries per sample and 8-lanes of 2-flow cells per library). First, the low-count genes (5+ reads in less than 10 replicates) were removed. After this step, 14 negative ERCC spike-ins (ratio = 1) and 45 positive ERCC spike-ins (ratio = 1/2, 2/3 or 4) were retained. Then we used the state-of-the-art RNA-seq normalization tool, RUVSeq [45], to normalize all 128 replicates using the negative ERCC spike-ins and 1000 least differentially expressed genes (ranked by edgeR *p*-values) as the *in silico* empirical negative control genes. In particularly, we used RUVg (Remove Unwanted Variation Using Control Genes) and followed the practice of RUVg authors described in the online methods of [45] by dropping the first unwanted factor and retained the next 6 factors. After normalizing the replicates, we randomly chose 12 replicates from one library from the sample A and divide them into two equal-size groups to form the base of non-DEFs (the results using different number of replicates are provided in Additional file 1: Figures S61–72). Occasionally we obtained two very distinct groups because the above normalization procedure could not get rid of all unwanted variations. To avoid this problem, we applied PoissonSeq to calculate the *p*-values of the true non-DEFs being differentially expressed between the chosen groups, and redid grouping if the *p*-value distribution of the true non-DEFs was not closed to uniform between 0 and 1. PoissonSeq was used because the Poisson distribution was reported to be effective for modelling technical replicates [17]. Finally, we replaced the values of the positive ERCC spike-ins and the positive TaqMan genes in one of the chosen groups by their values in 6 randomly selected replicates of sample B. This arrangement should make the positive TaqMan genes as the true DEFs and the remaining genes as the true non-DEFs.

**Fig. 8:**
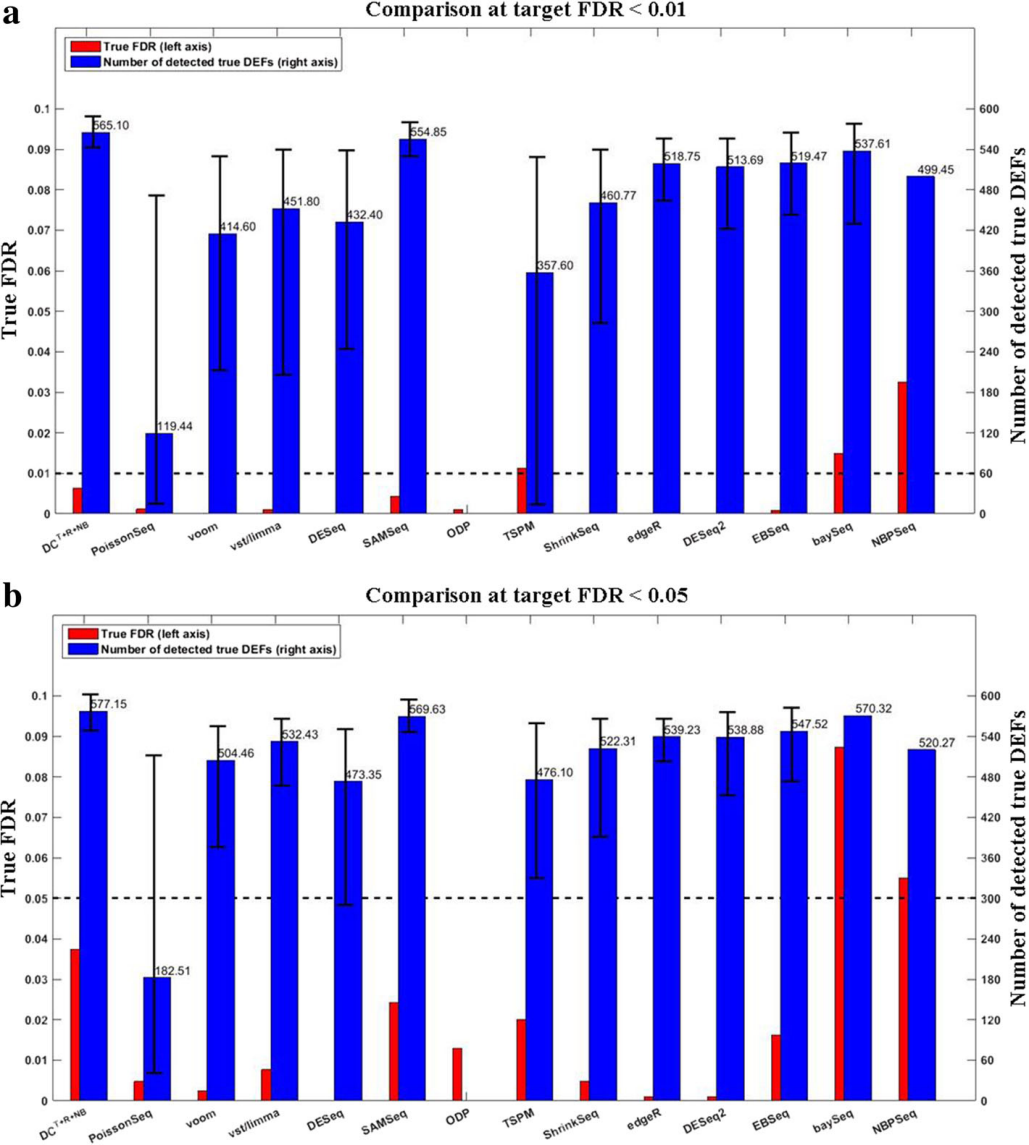
Evaluates RNA-seq differential expression analysis methods using the SEQC/MAQC-III dataset. The *red bars* indicate the average true FDRs (refer to the left *y*-axis). The *horizontal dashed line* across the figure marks the target FDR. The *blue bars* indicate the average number of the detected true DEFs (refer to the right *y*-axis). The 90% confidence intervals of the detected DEFs are marked except for those whose true FDRs exceed the target FDR by 10%. **a** target FDR < 0.01. **b** target FDR < 0.05. At both target FDR levels, most methods effectively controlled the FDR. DC^*T*+*R*+*NB*^ is the most powerful one (see main text for detailed discussions)

**Fig. 9:**
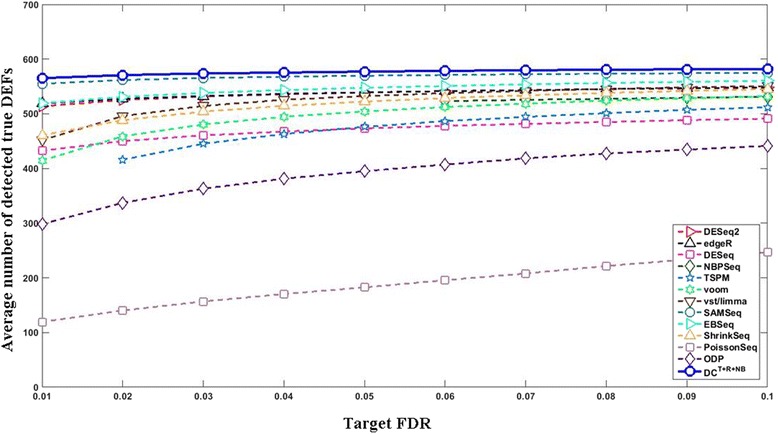
The curves of the true positives vs. the target FDR using the SEQC/MAQC-III dataset. The *x*- and *y*- axes indicate the target FDR level and the average number of true positives, respectively. The *solid curve* with *blue circle* markers represents DC^*T*+*R*+*NB*^, and other curves represent non-DC methods. The result of a method at a particular target FDR is shown if (1) its true FDR does not exceed the target FDR by 10%; and (2) it detects on average ≥0.5 true DEFs (rounds up to 1)

**Fig. 10:**
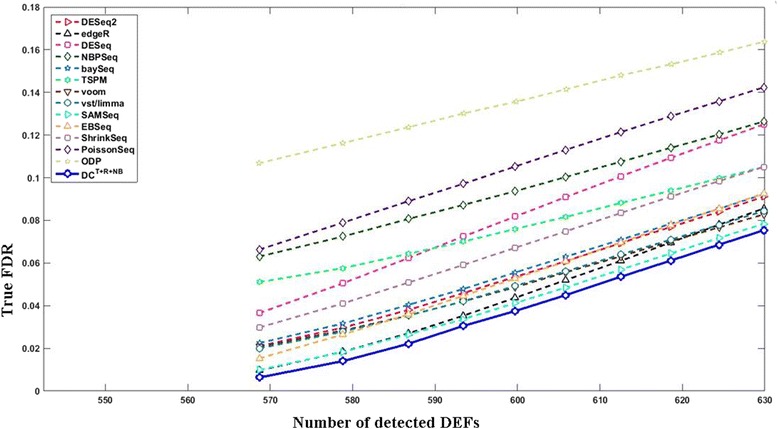
The curves of the true FDR vs. the number of the detected DEFs using the SEQC/MAQC-III dataset. The *x*- and *y*- axes indicate the number of detected DEFs and the average true FDR, respectively. The curve of DC^*T*+*R*+*NB*^ (*solid curve* with *blue circle* markers) in this figure were converted from the results obtained by setting the target FDR between 0.01 and 0.1 with an increasing step of 0.01. The curves of other methods were obtained by letting them call the same number of DEFs detected by DC^*T*+*R*+*NB*^ at each target FDR

**Fig. 11:**
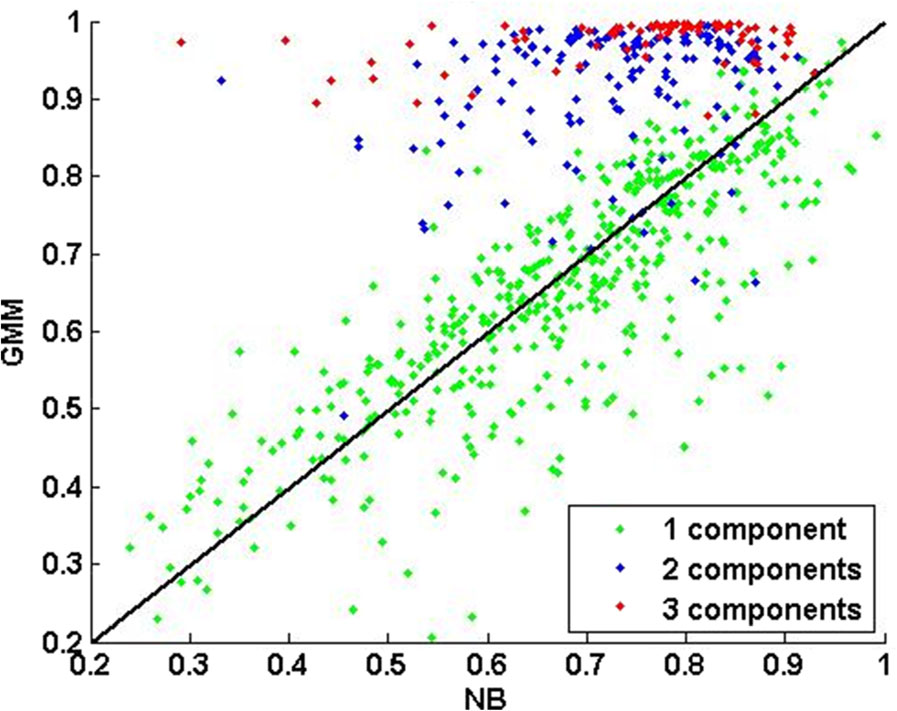
Some positive TaqMan genes have complex distributions in technical replicates (the 1st library of sample B). *Y*-axis: the correlation coefficients between the normalized-count distributions and the corresponding fitted GMM distribution. *X*-axis: the correlation coefficients between the normalized-count distributions and the corresponding fitted NB distribution. Each *dot* represents a gene. The distribution of a gene’s normalized counts is approximated by a histogram of 20 equal-size bins spanning its read-count value range. The colors of dots indicate the most proper numbers of components in a fitted GMM according to the Bayesian Information Criterion: *green* (~67.5%), *blue* (~21.8%), and *red* (~10.7%) correspond to 1, 2 and 3 components, respectively

The data obtained above was then used to benchmark different DEF detection methods. We repeated the above procedure 100 times. The results are summarized in Fig. 8. All DC configurations and most non-DC methods were able to effectively control the FDR at both target FDR levels (0.01 and 0.05). Among those able to effectively control the FDR, DC^*T*+*R*+*NB*^ was the most powerful. At target FDR < 0.01, DC^*T*+*R*+*NB*^ on average detected 565.10 true DEFs, which is significantly better (paired *t*-test *p*-value = 1.58e-25) than the 554.85 true DEFs detected by the best non-DC method (SAMSeq). At target FDR < 0.05, DC^*T*+*R*+*NB*^ on average detected 577.15 true DEFs, which is significantly better (paired *t*-test *p*-value = 5.58e-27) than the 569.63 true DEFs detected by the best non-DC method (SAMSeq). The leads of DC^*T*+*R*+*NB*^ over non-DC methods are not as large as those in the simulation test because we used technical replicates in this experiment. Figures 9 and 10 compare the curves of “the true positives vs. the target FDR” and FDCs, respectively. The supreme performance of DC^*T*+*R*+*NB*^ can be explained by Fig. 11, which shows that the normalized-count distributions of some positive TaqMan genes are complex even within the chosen technical replicate subset.

### Analyze a methylation dataset

To demonstrate the general applicability of DC, we applied it to analyze a DNA methylation dataset generated by Aldinger et al. [46] using the Illumina HumanMethylation27 BeadChip, which can be downloaded as GSE34099 from GEO. This data set contains global DNA methylation of 18 Rett syndrome samples and 19 control samples. Since the nature of this dataset is quite different from typical RNA-seq count data, we did not include methods developed specifically for RNA-seq in this comparison. Instead, we focused on the applicability of DC and assessing the benefits of using more than one attributes. We selected five basic statistics and let DC use two of them in each run: (1) *s* ^*T.SAM*^ – the corrected *t*-statistic [10, 47]; (2) *s* ^*T.log.SAM*^ – the corrected *t*-statistic with logarithmic transformation; (3) *s* ^*R.SAM*^ – the corrected ranksum statistic [10, 47]; (4) *s* ^*T.voom*^ – the moderated *t*-statistic produced by voom; and (5) *s* ^*T.limma*^ – the moderated *t*-statistic produced by limma. The original data values were multiplied by 1000 and then rounded to the nearest integer if an attribute extraction package only accepts integer inputs. The NB-based basic attributes (e.g., the Wald statistic for the NB-based differential expression test by DESeq2) were not used because the distributions of DNA methylation features in this dataset are quite different from the NB distribution.

**Fig. 12:**
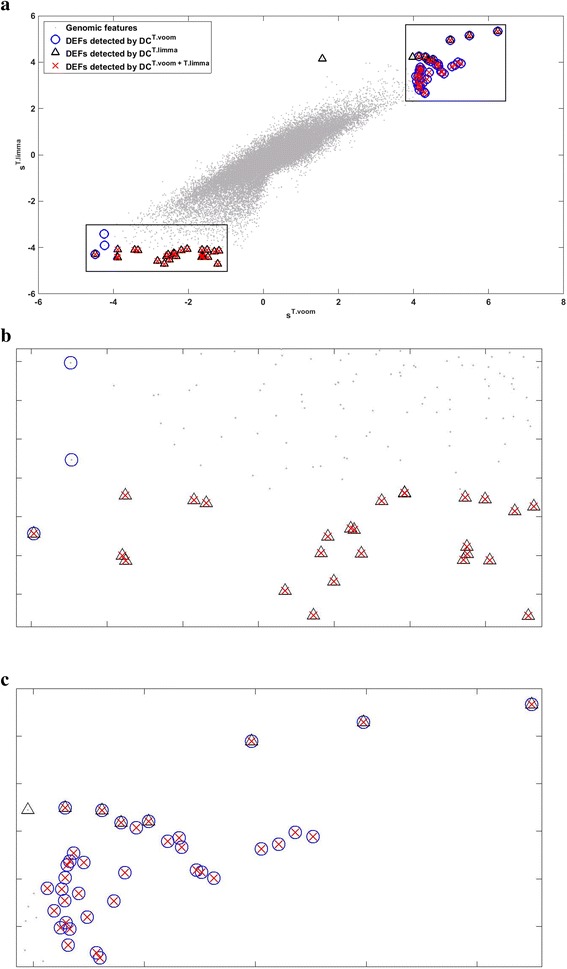
Compares the DEFs detected by DC^*T.voom*^ (*blue circles*), DC^*T.limma*^ (*black triangles*), and DC^*T.limma*+*T.voom*^ (*red crosses*) in GSE34099. Each *dot* represents a feature in the dataset. **a** The *X*-axis and *Y*-axis indicate the *s* ^*T.voom*^ and *s* ^*T.limma*^ attributes, respectively. **b** & **c** are two blow-outs of the corresponding areas in (**a**) for better view. See main text for detailed discussions

The results at FDR < 0.01 (Table 5) show that combining two basic attributes is significantly advantageous over utilizing single ones. For example, DC^*T.limma*+*T.voom*^ detected 63 DEFs, which is much higher than the 35 and 40 DEFs detected by DC^*T.limma*^ and DC^*T.voom*^, respectively. This result is interesting because both *s* ^*T.voom*^ and *s* ^*T.limma*^ are moderated *t*-statistics and voom utilizes limma to calculate its test statistics after applying a log-count per million transformation to the original data. Nevertheless, the integration of *s* ^*T.voom*^ and *s* ^*T.limma*^ by DC can achieve significantly higher detection power than using one of them. The main reason underlying this observation is visualized in Fig. 12: The joint distribution of *s* ^*T.limma*^ and *s* ^*T.voom*^ are quite asymmetric and non-Gaussian. It is more advantageous to use *s* ^*T.voom*^ and *s* ^*T.limma*^ to detect DEFs in the up- and down-regulated regions, respectively. Their advantages can be integrated by DC that rigorously explores the structures in the joint distribution of *s* ^*T.voom*^ and *s* ^*T.limma*^ to achieve better DEF detection results.

**Table 5:** Compares the performances of DC using different pairs of basic attributes on GSE34099

Basic Attribute #2 Basic Attribute #1	*s* ^*T.SAM*^	*s* ^*T.log.SAM*^	*s* ^*R.SAM*^	*s* ^*T.voom*^	*s* ^*T.limma*^
*s* ^*T.SAM*^	27	43	44	61	37
*s* ^*T.log.SAM*^	43	25	39	43	46
*s* ^*R.SAM*^	44	39	35	56	46
*s* ^*T.voom*^	61	43	56	40	63
*s* ^*T.limma*^	37	46	46	63	35

The first column and row indicate the basic attributes used by DC. The diagonal cells list the numbers of DEFs detected by DC using single attributes. The rest of cells list the numbers of DEFs detected by DC using different combinations of two basic attributes

## Discussions

Conventional methods for differential expression analysis often use individual basic attributes (e.g., fold-change, ranksum statistics, or other statistics based on simple distributional assumptions), which may significantly underestimate the complexity observed in reality. This is partially because the datasets, which were available when those analysis methods were developed, usually contained only a few replicates. It can also be due to underestimation of the underlying biological variations. We have shown in this paper that insufficient characterization of differential expression information could lead to low detection power and/or higher-than-expected FDRs. It is expected that future studies will produce sufficiently large number of replicates because the collaboration scales are quickly growing larger and the rapid advances of high-throughput technologies will bring down the experimental cost dramatically. Therefore, it is important to develop novel DEF detection methods with better capability of dealing with complex differential expression patterns. To this end, we proposed to utilize multiple basic attributes to better capture differential expression information and formulate the problem of detecting DEFs as optimizing discriminant boundary constrained by a user-defined FDR cutoff in a multi-dimensional space. We have developed the Discriminant-Cut (DC) algorithm for dealing with a special family of discriminant functions (i.e., linear boundaries). The comparison of DC with several existing DEF detection methods using simulated datasets and the SEQC/MAQC-III RNA-seq dataset confirms the advantages of DC in handling complex differential expression patterns. In addition, we also show an application of DC to analyze microarray datasets, and expect that DC can be used (maybe with slight extensions) to analyze many different types of high-throughput datasets. In the future, we will explore our approach for meta-analysis [48] that integrate multiple datasets.

Using linear discriminant functions is an effective step forward, but it may not be powerful enough to fully utilize large-scale datasets. More powerful methods can be developed in the future by exploring more sophisticated discriminant function families and learning techniques. Discriminant analysis by integrating heterogeneous attributes is popular in many machine-learning research and its applications (e.g., computer vision, natural language processing, speech recognition, etc.). It is mostly done in supervised way that can rely on labelled information to perform calibration. Our approach is unsupervised and uses the estimated FDR for self-calibration. This kind of machine learning problem has not been widely researched, and hence can be of great interest to future research.

Our approach greatly benefits from the attributes designed by previous research on differential analysis (such as, SAM, DESeq2, voom, vst/limma, and so on). We believe that we are far from fully exploring the potentials of those attributes. On the other hand, it is possible that some attributes may be redundant (i.e., can be replaced by combinations of other attributes) or their information cannot be effectively utilized by the chosen discriminant function family. Which attributes are effective depends on the characteristics of the dataset under analysis. DC already has certain attribute selection capability because it applies the L_1_ regularization. However, we believe attribute selection remains an open problem and can be domain specific. We will investigate this problem in the context of detecting DEFs in the future. As far as we know, our work is the first one that formerly introduces unsupervised multi-dimension discriminant analysis to DEF detection, which can be a new direction to significantly advance the DEF detection research as supported by our experimental results.

## Conclusion

This paper presents a novel machine learning methodology for robust differential expression analysis, which can be a new avenue to significantly advance research on large-scale differential expression analysis. The corresponding mathematical model was formulated as a constrained optimization problem aiming to maximize discoveries satisfying a user-defined FDR constraint. An effective algorithm, Discriminant-Cut, was developed to solve an instantiation of this problem. Extensive comparisons of Discriminant-Cut with a couple of cutting edge methods were carried out to demonstrate its robustness and effectiveness.

## Additional file

Additional file 1: Additional documentation. (DOCX 9047 kb)

## Abbreviations

DC: Discriminant-Cut
DEF: Differential expressed features
FDR: False discovery rate
