# Supplementary material for: Robust differential expression analysis by learning discriminant boundary in multi-dimensional space of statistical attributes
Source: BMC Bioinformatics. 2016 Dec 19;17:541. doi: 10.1186/s12859-016-1386-x (PMC5168810; doi:10.1186/s12859-016-1386-x)
Supplement: Additional file 1: — Additional documentation. (DOCX 9047 kb) [file 12859_2016_1386_MOESM1_ESM.docx]

**Robust differential expression analysis by learning discriminant boundary in multi-dimensional space of statistical attributes**

**Contents**

[1. Discriminant-Cut Algorithm 2](#_Toc436604907)

[1.1. Speed up Discriminant-Cut 2](#_Toc436604908)

[1.2. Instructions for Using Discriminant-Cut 3](#_Toc436604909)

[2. Additional Simulation Test Results 6](#_Toc436604910)

[2.1. Number of Detected True DEFs and True FDR of Different Methods 6](#_Toc436604911)

[2.2. Curves of True Positives against Target FDR 26](#_Toc436604912)

[2.3. Curves of True FDR against Number of Detected DEFs 46](#_Toc436604913)

[3. Additional SEQC/MAQC-III Test Results 66](#_Toc436604914)

[4. R-codes for the RNA-seq DEF Detection Methods Used in Comparisons 78](#_Toc436604915)

[4.1. DESeq2 78](#_Toc436604916)

[4.2. DESeq 78](#_Toc436604917)

[4.3. voom/limma 78](#_Toc436604918)

[4.4. vst/limma 78](#_Toc436604919)

[4.5. edgeR 79](#_Toc436604920)

[4.6. NBPSeq 79](#_Toc436604921)

[4.7. baySeq 79](#_Toc436604922)

[4.8. EBSeq 79](#_Toc436604923)

[4.9. TSPM 80](#_Toc436604924)

[4.10. SAMSeq 80](#_Toc436604925)

[4.11. ShrinkBayes 80](#_Toc436604926)

[4.12. PoissonSeq 81](#_Toc436604927)

[4.13. ODP 81](#_Toc436604928)

[References 81](#_Toc436604929)

# Discriminant-Cut Algorithm

**Algorithm S1 –** [$f^{+}\left( \cdot\right),f^{-}\left( \cdot\right), \Gamma^{+}, \Gamma^{-}$] = **Discriminant-Cut**($\mathbb{S}\left( \boldsymbol{X} \right), \Psi$)

**Inputs:**

1. $\mathbb{S}\left( \boldsymbol{X} \right)=\left\{ s_{i}^{\left( k \right)} \right\}_{i=1\ldots M;k=1\ldots K}$ is the basic attribute set, where $s_{i}^{\left( k \right)}$ is the *k*-th basic attribute of *i*-th feature in the dataset $\boldsymbol{X}$.
2. $\Psi$ is the desired FDR (e.g., 0.05) chosen by the user.

**Outputs:**

1. $f^{+}\left( \cdot\right)$ and $f^{-}\left( \cdot\right)$ are the best discriminant functions to detect the up- and down-regulated DEFs, respectively.
2. $\Gamma^{+}$ and $\Gamma^{-}$ are the up- and down-regulated DEF sets, respectively.

**Steps:**

1. Initialize $\Gamma^{+}\leftarrow\emptyset$, $\Gamma^{-}\leftarrow\emptyset$.
2. Populate a candidate function set from the homogeneous linear function family to cover the function space with an empirically decided resolution:

$$\mathcal{G}_{K}=\left\{ \left. g\left( s_{1},\ldots,s_{K} \right)=\sum_{i=1}^{K} w_{i}d_{i} \right|w_{i}\in\left\{ 0,0.1,\ldots,1 \right\},\sum_{i=1}^{K} w_{i}=1 \right\}$$

3. For each $g\left( \cdot\right)\in\mathcal{G}_{K}$:
   1. Calculate homogeneous discriminant set $\mathbb{C}\leftarrow\left\{ g\left( s_{i}^{\left( 1 \right)},s_{i}^{\left( 2 \right)},\ldots,s_{i}^{\left( K \right)} \right) \right\}_{i=1\ldots M}$.
   2. Use binary search to adjust $\tau^{+}>0$ to maximize $\left| \Gamma\left( \boldsymbol{X},g-\tau^{+} \right) \right|$ while making sure ${\hat{\mathrm{FDR}}}_{\lambda}\left( \boldsymbol{X},g-\tau^{+} \right)<\Psi$. Eq. (8) is used to estimate ${\hat{\mathrm{FDR}}}_{\lambda}\left( \cdot\right)$ and $\lambda$ is by default set to 0.5. If , set $f^{+}\leftarrow g-\tau^{+}$ and $\Gamma^{+}\leftarrow\underset{\tau^{+}}{\mathrm{argmax}} \Gamma\left( \boldsymbol{X},f^{+} \right)$.
   3. Use binary search to adjust $\tau^{-}>0$ to maximize $\left| \Gamma\left( \boldsymbol{X},-g-\tau^{-} \right) \right|$ while making sure ${\hat{\mathrm{FDR}}}_{\lambda}\left( \boldsymbol{X},-g-\tau^{-} \right)<\Psi$. If , set $f^{-}\leftarrow-g-\tau^{-}$ and $\Gamma^{-}\leftarrow\underset{\tau^{-}}{\mathrm{argmax}} \Gamma\left( \boldsymbol{X},f^{-} \right)$.
4. Re-adjust $\Gamma^{+}$and $\Gamma^{-}$ for $f^{+}$ and $f^{-}$ using an independent permutation set other than the one used in FDR estimations in Step (3).
5. Return $f^{+},f^{-}, \Gamma^{+}, \Gamma^{-}$ .

## Speed up Discriminant-Cut

The following heuristics were designed to significantly improve the speed of **Algorithm S1**. They

1. Discriminant-Cut needs to query the set $\mathbb{C}^{*}=\left\{ \mathbb{C}_{b}^{*} \right\}_{b=1\ldots B}$ ($\mathbb{C}_{b}^{*}$ is the corresponding homogeneous discriminant set for $\boldsymbol{X}_{b}^{\boldsymbol{*}}$) when estimating FDR at step 3.2 and 3.3, which need to calculate Eq. (5) and Eq. (6) in the main text. This calculation can be sped up by applying the Quicksort algorithm to sort elements in $\mathbb{C}_{b}^{*}$ for each $b=1\ldots B$, and then use binary search on $\mathbb{D}^{*}$ to calculate Eq. (5) and Eq. (6).
2. Discriminant-Cut maximizes the number of detected DEFs while making sure the FDR is under controlled. If a sub-optimal solution, $\Gamma\left( \boldsymbol{X},g-\tau\right)$ is able to detect *x* DEFs, we only need to consider other candidates $g\in\mathcal{G}_{K}$ who are able to find at least *x*+1 DEFs while its estimated FDR is lower than the target FDR. This can dramatically reduce our search space (both the function and its constant term $\tau$).

## Instructions for Using Discriminant-Cut

The executable of Discriminant-Cut for Windows OS can be downloaded from the link below:
(<http://combio.cs.brandeis.edu/hong/DC/index.html>)

- Download and unpack Discriminant-Cut executable to local disk.
- Install MATLAB Runtime (MCRInstaller.exe) if MATLAB is not installed.
- Install R as well as package “limma” and “DESeq2” so that Discriminant-Cut can call them to generate basic attributes. “voom” is included in the “limma” package.
- Include the full path to “Rscript.exe” (e.g., C:\Program Files\R\R-3.1.2\bin\x64\Rscript.exe) in Rpath.txt. Do not include any other characters.
- Prepare the dataset in the following format, and put it under the “datasets” folder.

| ##Parameter1=Val1 | | | | | | | | | |
| --- | --- | --- | --- | --- | --- | --- | --- | --- | --- |
| ##Parameter2=Val2 | | | | | | | | | |
| … | | | | | | | | | |
| ##ParameterK=ValK | | | | | | | | | |
| NAME | DESC | DEF | 1 | 1 | … | 2 | 2 | … |
| Gene1 | Desc1 | 1 | XX | XX | … | XX | XX | … |
| Gene2 | Desc2 | 0 | XX | XX | … | XX | XX | … |
| … | … | … | … | … | … | … | … | … |
| GeneM | DescM | 0 | XX | XX | … | XX | XX | … |

The first *K* lines starting with “##”are annotations and are not utilized by Discriminant-Cut. The result lines contain *N*_1_+*N*_2_+3 tab-delimited columns. The *K*+1-th line contains the column headers. The first three columns are “NAME” (unique ID), “DESC” (annotations, e.g., gene name/symbol) and “DEF” (binary values indicating if a genomic feature is a true DEF. 0 – non-DEF; 1 – true DEF). The next *N*_1_ columns are all “1”s indicating the 1^st^ group. The last *N*_2_ columns are all “2”s indicating the 2^nd^ group. The rest *M* lines store the values of *M* genomic features, respectively. DC uses the “DEF” column to calculate the true FDR. If the ground truth is unknown, simply put 0s in the “DEF” column and ignore that the true FDR output by DC. Missing values are not supported.

- In the command line window, navigate to the directory where DiscriminantCut.exe is located. Run Discriminant-Cut using the following command:

*DiscriminantCut dataFile attList targetFDR nPerm resolution FPmethod*

Or simply type “*DiscriminantCut*” without any arguments to see the detailed descriptions about each argument. Using “*DiscriminantCut dataFile*” to run DC on the “dataFile” under the “datasets” folder with default settings: linear combination of three basic attributes (calculated by voom, ranksum and DESeq2), target FDR < 0.05, 100 permutations, empirical resolution = {0, 0.1, …, 0.9, 1}, and the median function is used to estimate expected false positive among permutations. We suggest using at least 600 permutations if the sample size is large enough to make the FDR estimation more accurate.

- Wait for a few seconds for the executable to launch MATLAB runtime environment. While Discriminant-Cut is running, several temporary files (“data\[dataFile].mat”, “stats\[dataFile].mat”, “results\[dataFile].mat”) will be saved to avoid repeating some computations in the future:
  - “data\[dataFile].mat” contains the dataset in MATLAB format to facilitate loading the data in the future.
  - “stats\[dataFile].mat” saves basic attributes of the original and permuted data. When user re-analyse the dataset with some different augment settings (e.g., changing the FDR cutoff from 0.05 to 0.01), Discriminant-Cut will utilize the pre-computed basic attributes. This feature is useful because calculating the basic attributes, especially those require to run external R scripts, are very time-consuming. Note that *nPerm* (i.e., the permutation number argument) will be omitted by Discriminant-Cut if it finds a basic attribute file of this dataset exists. To re-analyse the same dataset with a different permutation number, user needs to manually delete the temporary file “stats\[dataFile].mat” and the corresponding “results\[dataFile].mat”.
  - “results\[dataFile].mat” saves the analyse results as MATLAB structure. DC saves the analysis results using different augment settings together in this file. Note that DC will not re-analyze the dataset if it detects the result of the same augment setting exists in this file. To re-analyse the dataset using the same augment setting, you need to manually delete “results\[dataFile].mat” and then run Discriminant-Cut. This feature is useful for accessing the results repeatedly.
- After DC finishes running, a report file named “[dataFile]_yyyymmdd_HHMMSS.text” will be saved under the working directory. Open it in any text editor to see the results.

# Additional Simulation Test Results

## Number of Detected True DEFs and True FDR of Different Methods

**Simulation Test Results of *N* = 8,** $\boldsymbol{G}_{\mathbf{500}}^{\mathbf{500}}$


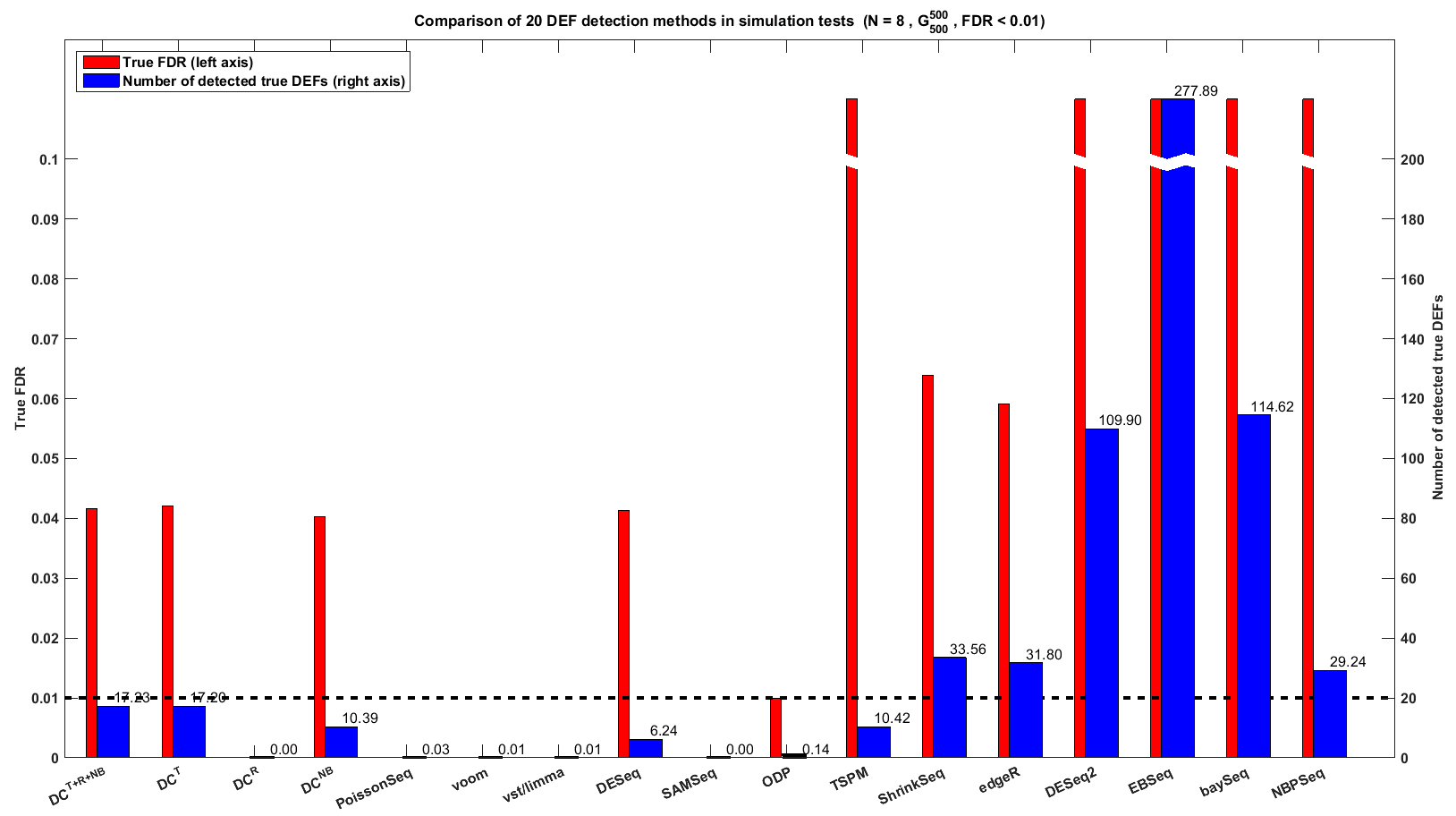

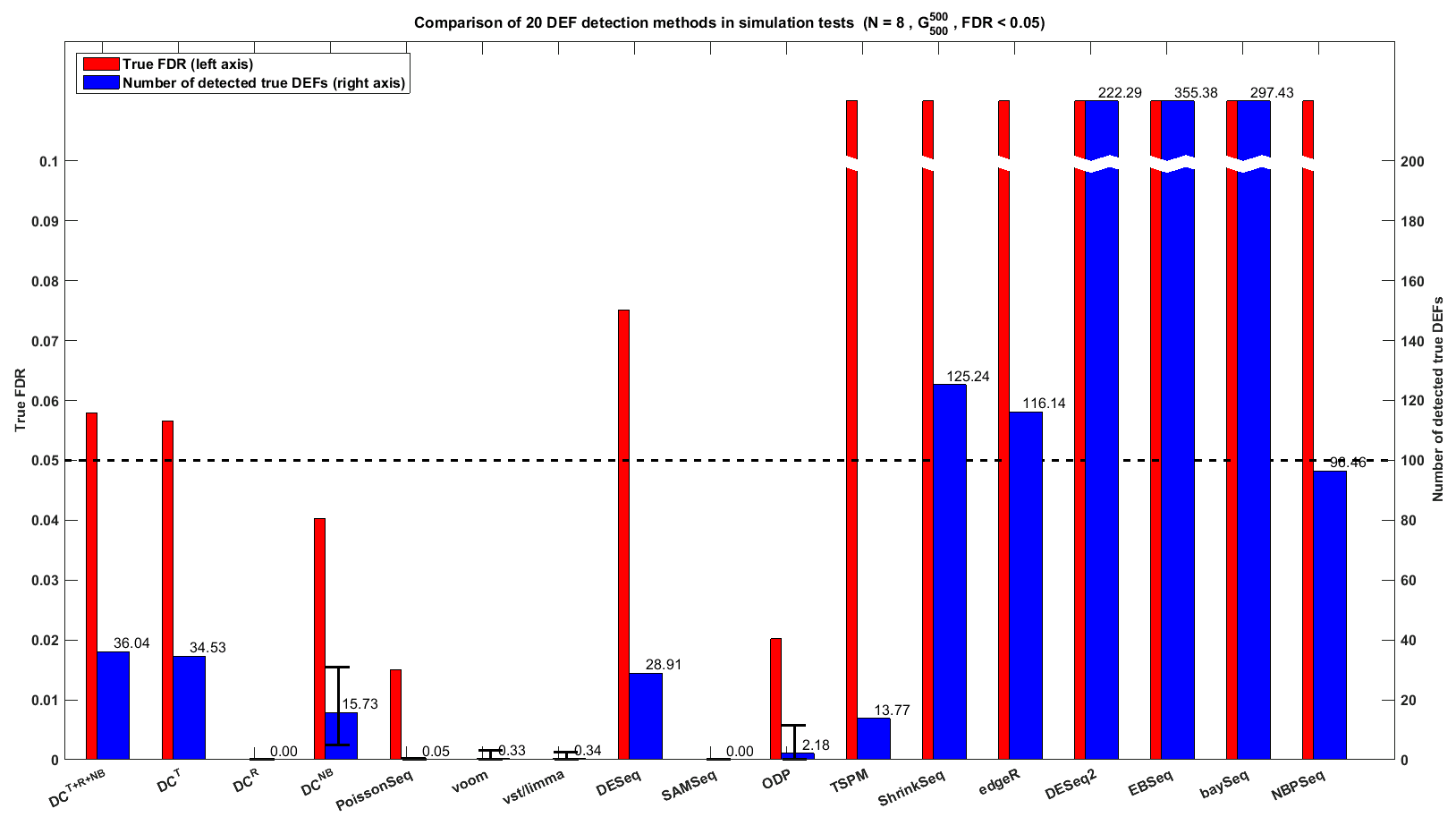


**(a) Comparison at target FDR < 0.01**

**(b) Comparison at target FDR < 0.05**

**True FDR**

**Number of detected true DEFs**

**Number of detected true DEFs**

**True FDR**

**Figure S1. Evaluates RNA-seq differential expression analysis methods using simulated data (4 *vs.* 4; $\boldsymbol{G}_{\boldsymbol{500}}^{\boldsymbol{500}}$)**. Methods are listed along the ***x***-axis. The red bars indicate the average true FDRs (refer to the left ***y***-axis). The horizontal dashed line across the figure marks the target FDR. The blue bars indicate the average number of the detected true DEFs (refer to the right ***y***-axis). The 90% confidence intervals of the detected DEFs are marked except for those whose true FDRs exceed the target FDR by 10%. **(a)** target FDR < 0.01. **(b)** target FDR < 0.05.

**Simulation Test Results of *N* = 10,** $\boldsymbol{G}_{\mathbf{500}}^{\mathbf{500}}$


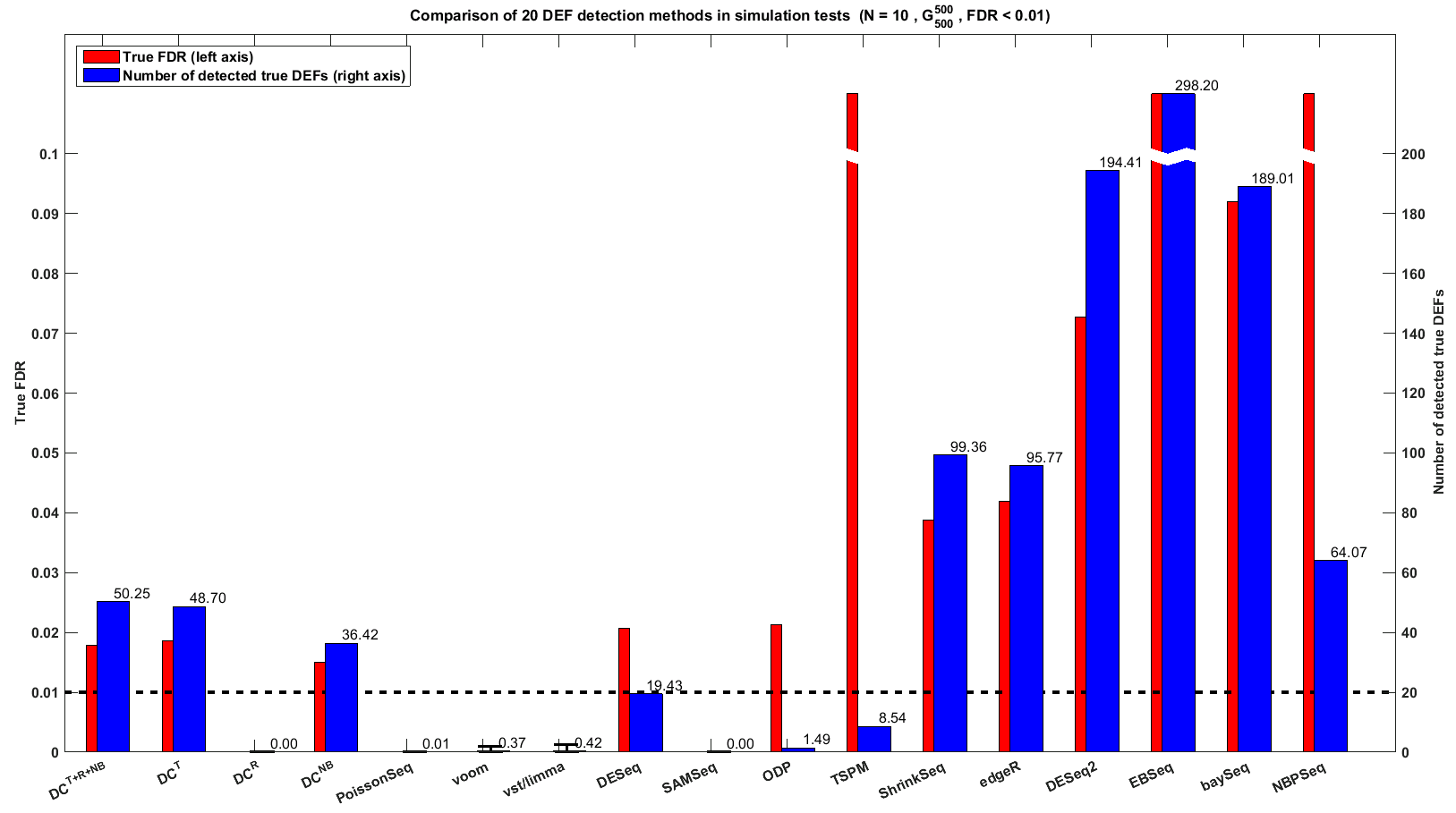

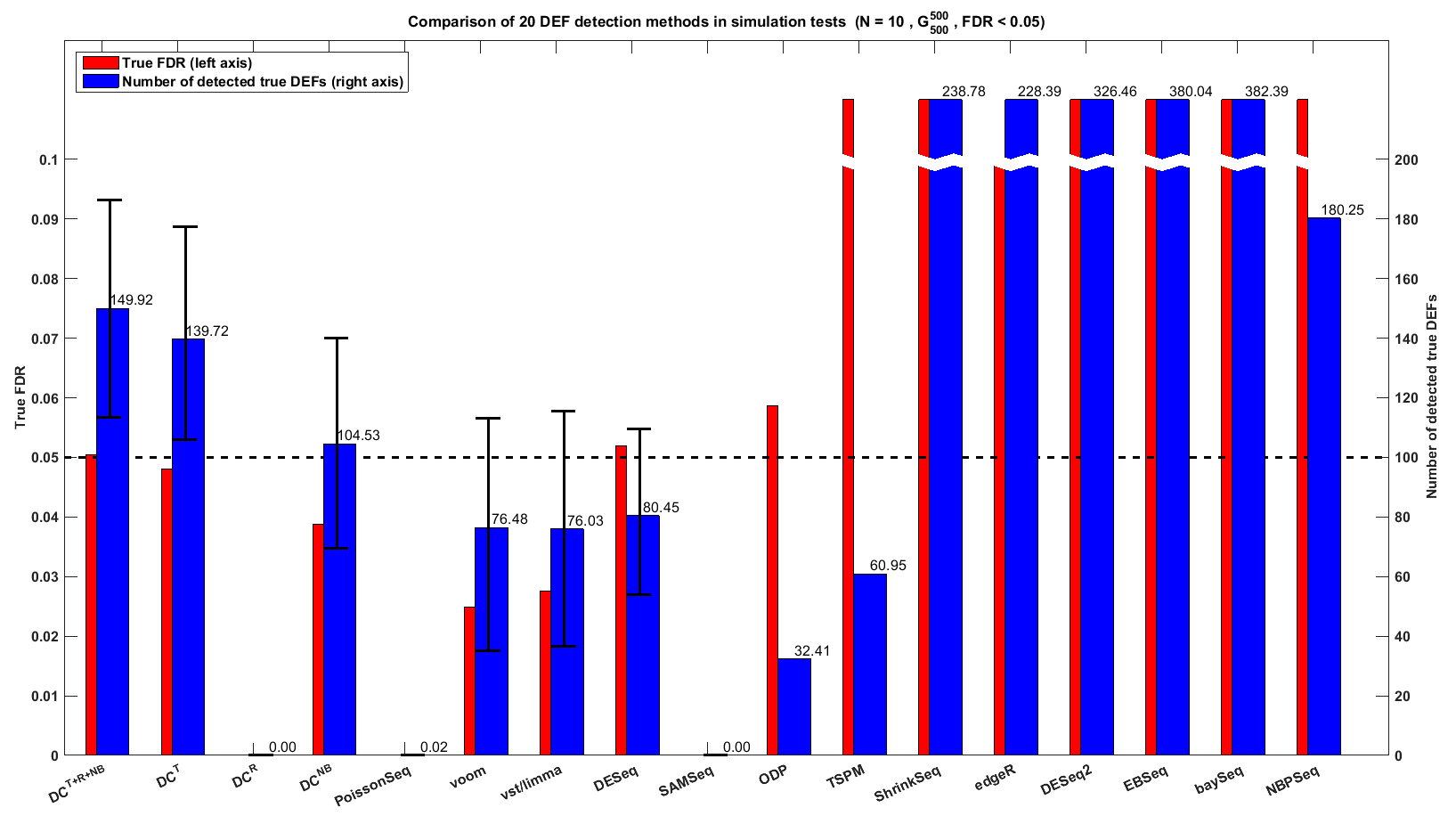


**(a) Comparison at target FDR < 0.01**

**(b) Comparison at target FDR < 0.05**

**True FDR**

**Number of detected true DEFs**

**Number of detected true DEFs**

**True FDR**

**Figure S2. Evaluates RNA-seq differential expression analysis methods using simulated data (5 *vs.* 5; $\boldsymbol{G}_{\boldsymbol{500}}^{\boldsymbol{500}}$)**. Methods are listed along the ***x***-axis. The red bars indicate the average true FDRs (refer to the left ***y***-axis). The horizontal dashed line across the figure marks the target FDR. The blue bars indicate the average number of the detected true DEFs (refer to the right ***y***-axis). The 90% confidence intervals of the detected DEFs are marked except for those whose true FDRs exceed the target FDR by 10%. **(a)** target FDR < 0.01. **(b)** target FDR < 0.05.

**Simulation Test Results of *N* = 12,** $\boldsymbol{G}_{\mathbf{500}}^{\mathbf{500}}$


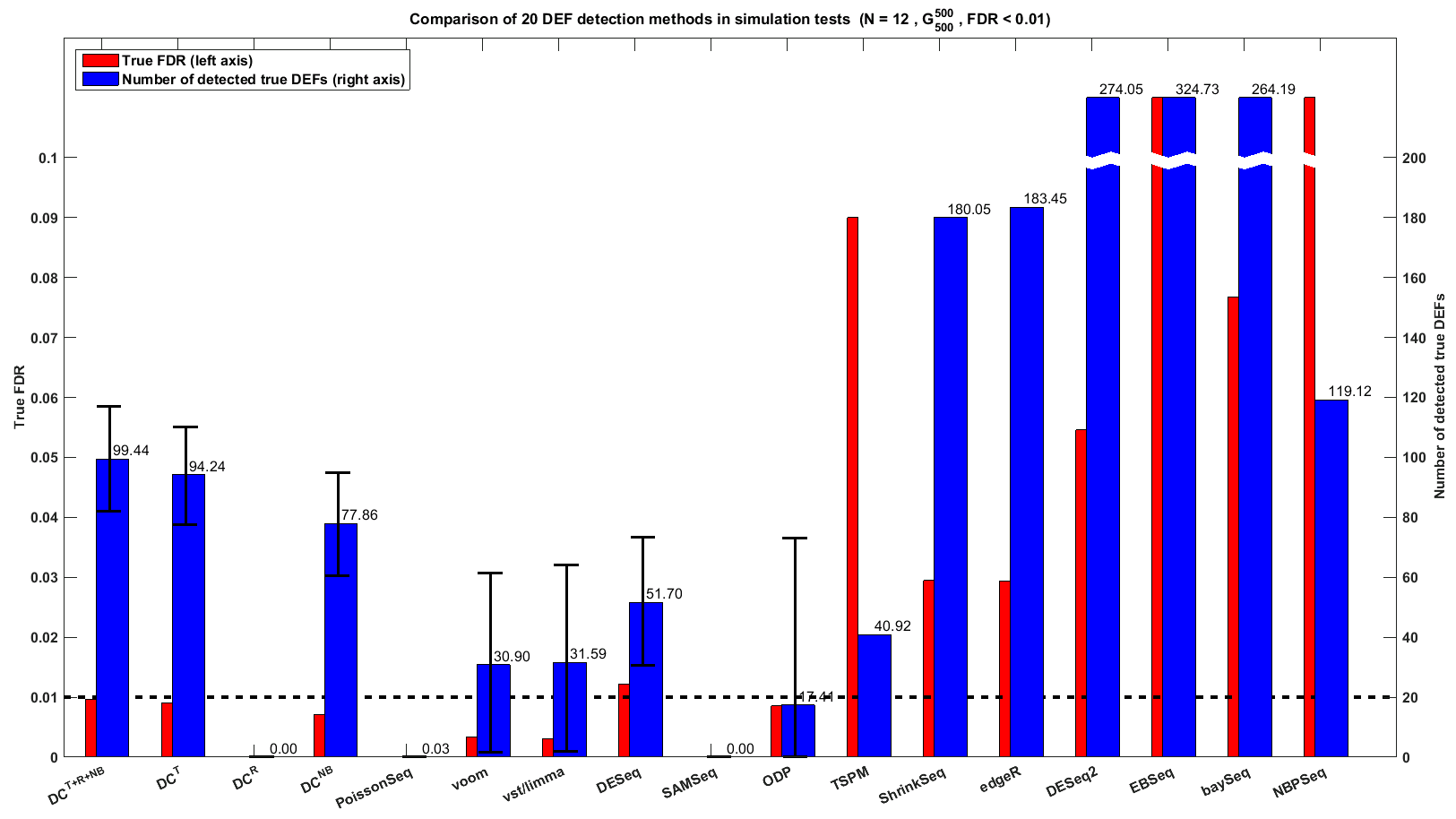

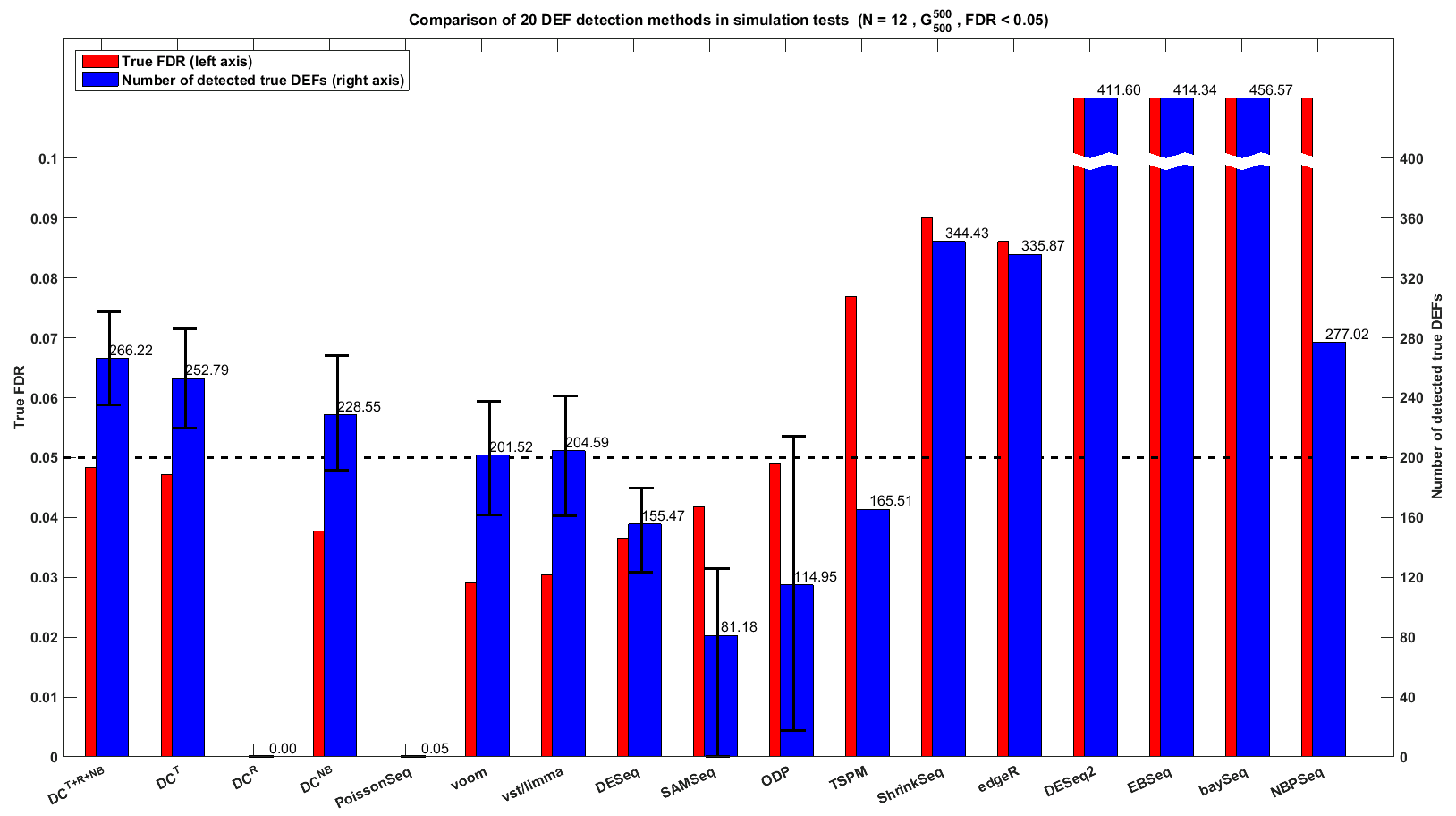


**(a) Comparison at target FDR < 0.01**

**(b) Comparison at target FDR < 0.05**

**True FDR**

**Number of detected true DEFs**

**Number of detected true DEFs**

**True FDR**

**Figure S3. Evaluates RNA-seq differential expression analysis methods using simulated data (6 *vs.* 6; $\boldsymbol{G}_{\boldsymbol{500}}^{\boldsymbol{500}}$)**. Methods are listed along the ***x***-axis. The red bars indicate the average true FDRs (refer to the left ***y***-axis). The horizontal dashed line across the figure marks the target FDR. The blue bars indicate the average number of the detected true DEFs (refer to the right ***y***-axis). The 90% confidence intervals of the detected DEFs are marked except for those whose true FDRs exceed the target FDR by 10%. **(a)** target FDR < 0.01. **(b)** target FDR < 0.05.

**Simulation Test Results of *N* = 16,** $\boldsymbol{G}_{\mathbf{500}}^{\mathbf{500}}$


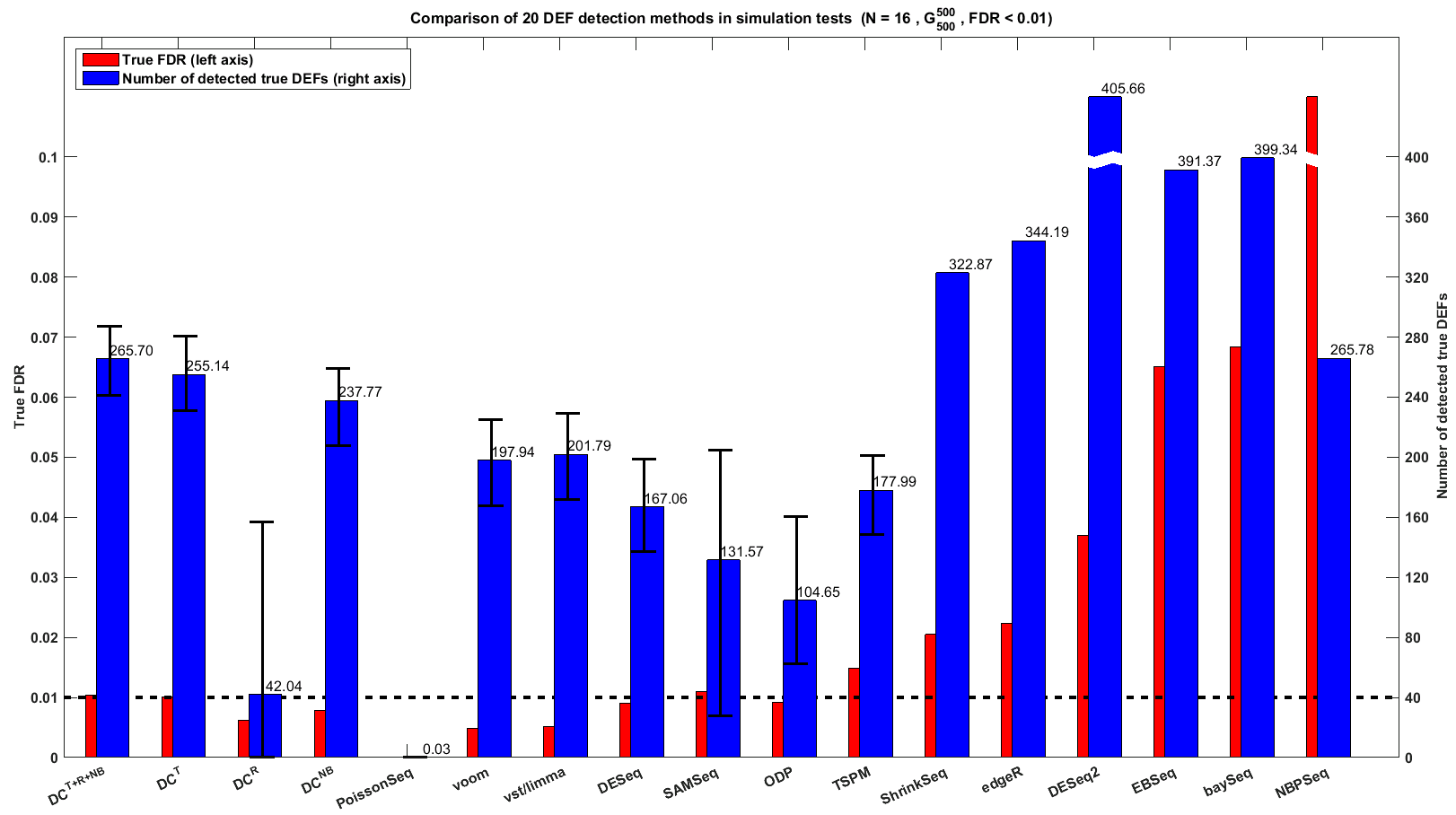

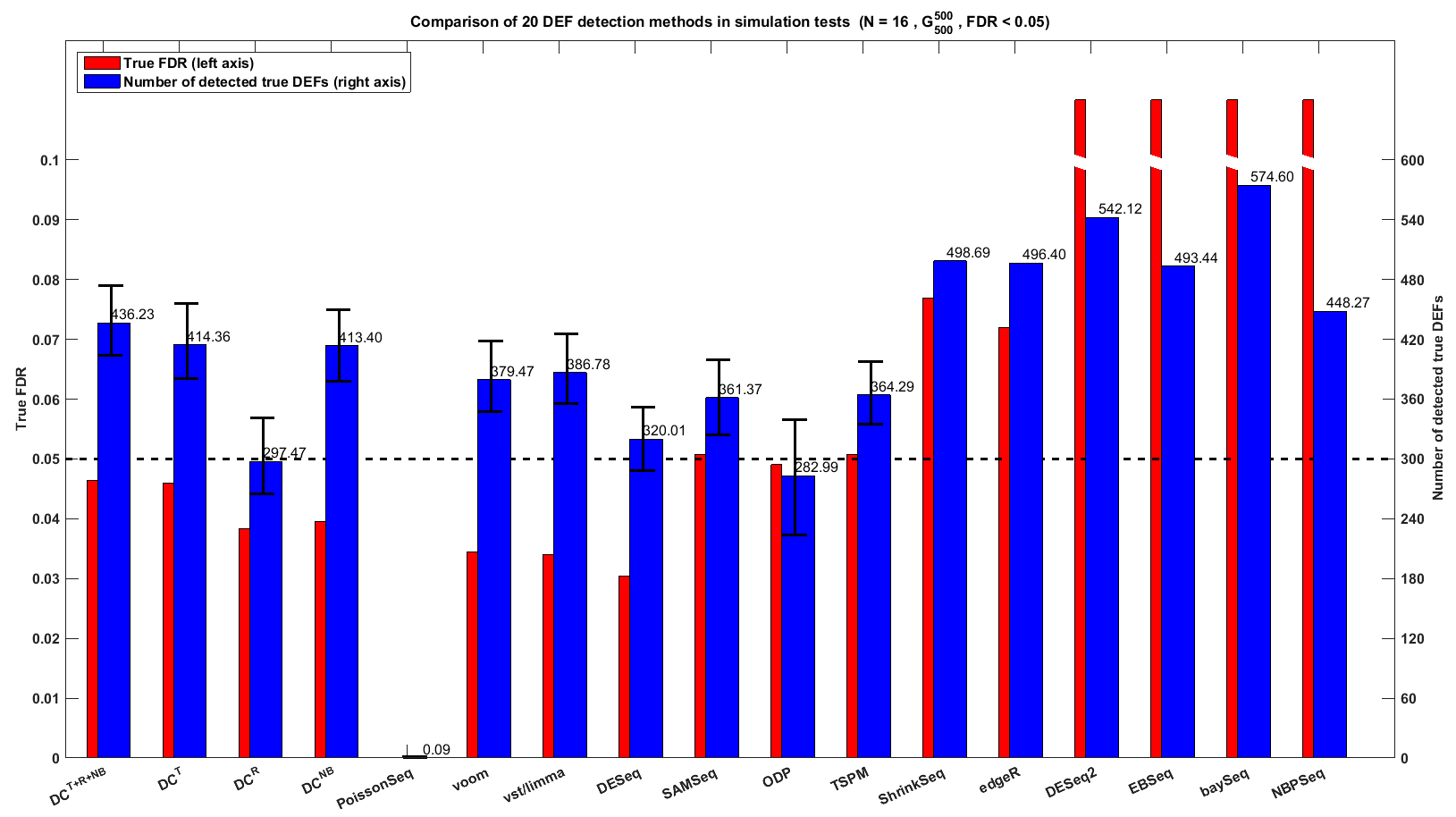


**(a) Comparison at target FDR < 0.01**

**(b) Comparison at target FDR < 0.05**

**True FDR**

**Number of detected true DEFs**

**Number of detected true DEFs**

**True FDR**

**Figure S4. Evaluates RNA-seq differential expression analysis methods using simulated data (8 *vs.* 8; $\boldsymbol{G}_{\boldsymbol{500}}^{\boldsymbol{500}}$)**. Methods are listed along the ***x***-axis. The red bars indicate the average true FDRs (refer to the left ***y***-axis). The horizontal dashed line across the figure marks the target FDR. The blue bars indicate the average number of the detected true DEFs (refer to the right ***y***-axis). The 90% confidence intervals of the detected DEFs are marked except for those whose true FDRs exceed the target FDR by 10%. **(a)** target FDR < 0.01. **(b)** target FDR < 0.05.

**Simulation Test Results of *N* = 20,** $\boldsymbol{G}_{\mathbf{500}}^{\mathbf{500}}$


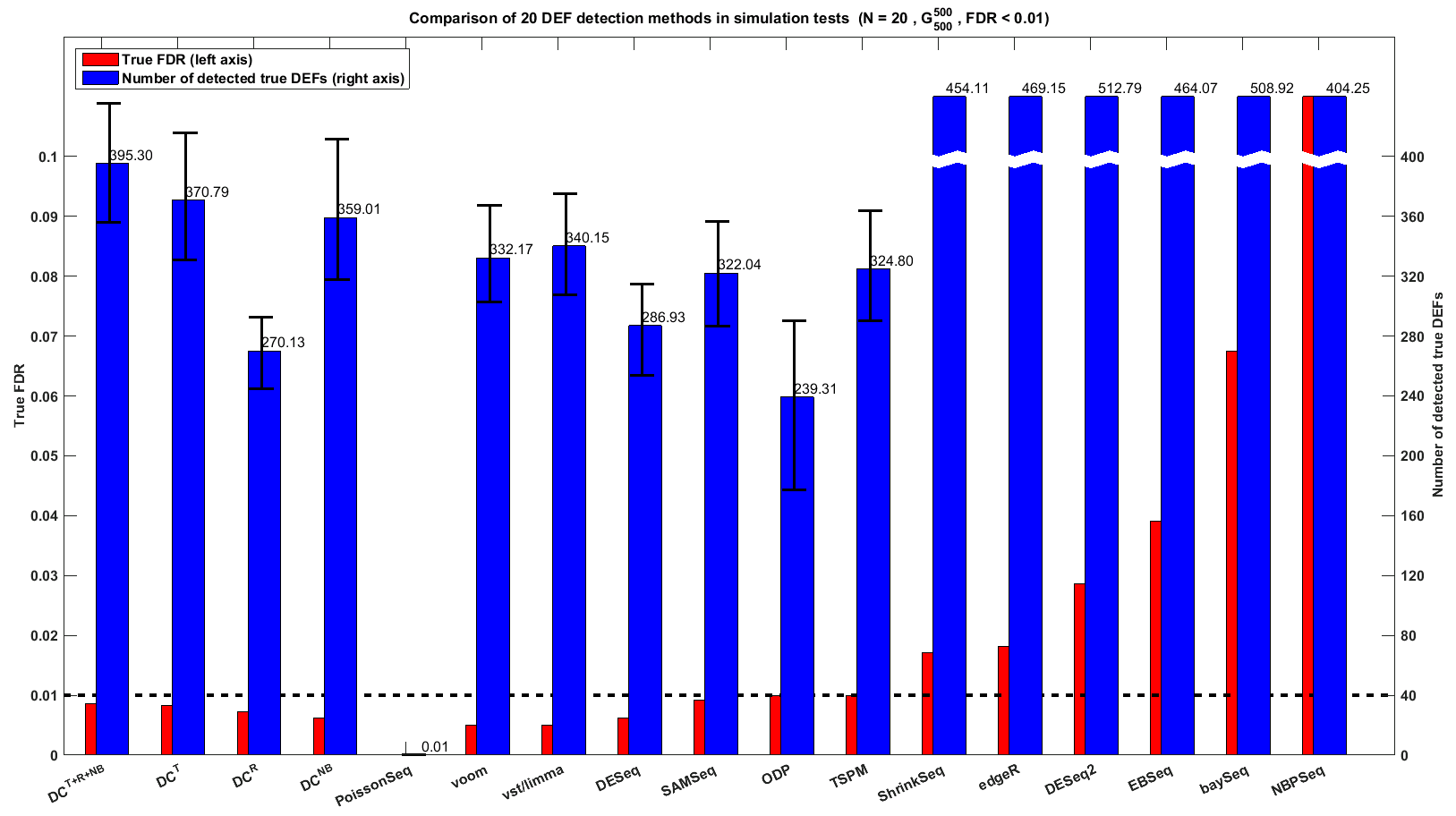

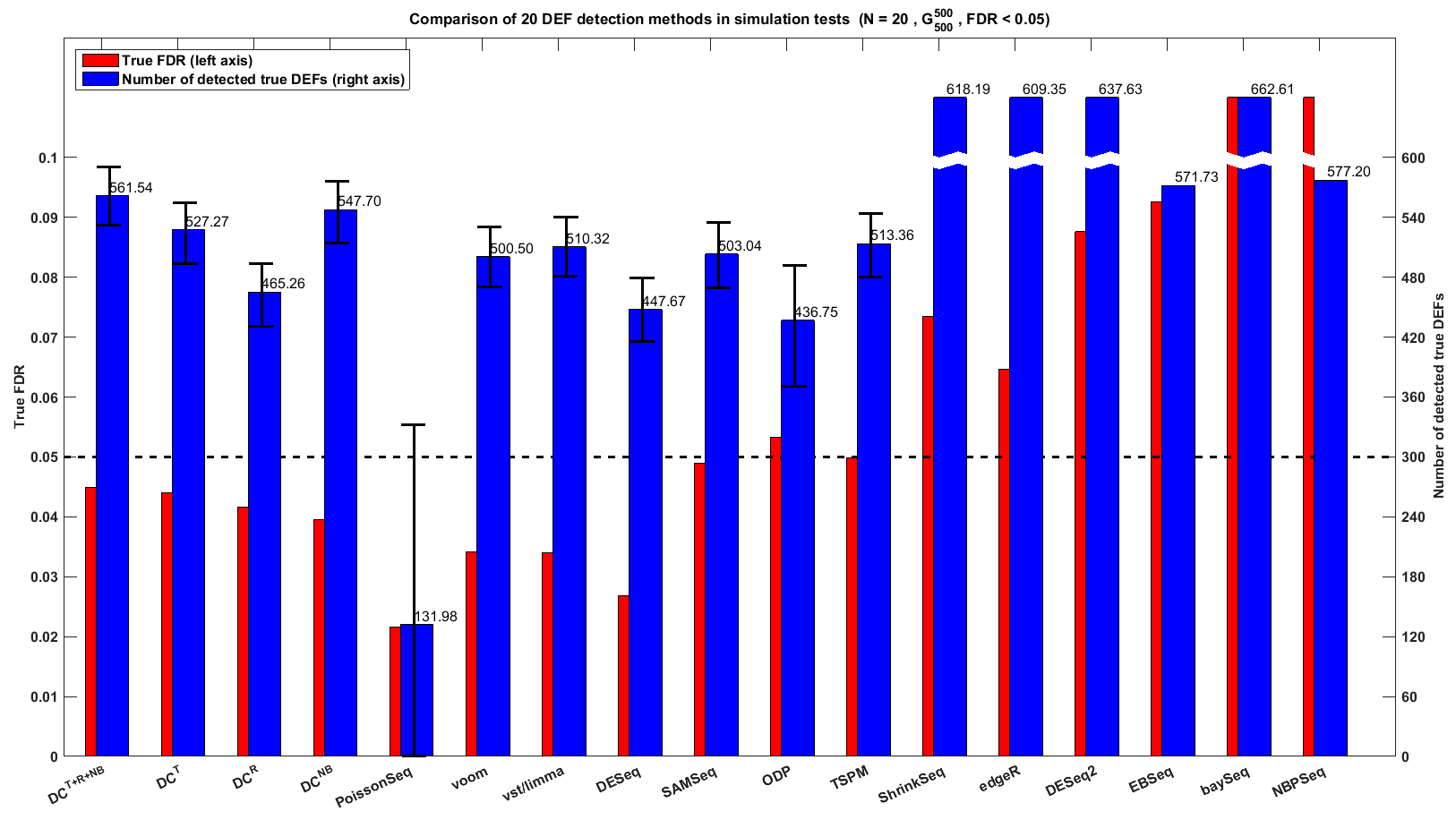


**(a) Comparison at target FDR < 0.01**

**(b) Comparison at target FDR < 0.05**

**True FDR**

**Number of detected true DEFs**

**Number of detected true DEFs**

**True FDR**

**Figure S5. Evaluates RNA-seq differential expression analysis methods using simulated data (10 *vs.* 10; $\boldsymbol{G}_{\boldsymbol{500}}^{\boldsymbol{500}}$)**. Methods are listed along the ***x***-axis. The red bars indicate the average true FDRs (refer to the left ***y***-axis). The horizontal dashed line across the figure marks the target FDR. The blue bars indicate the average number of the detected true DEFs (refer to the right ***y***-axis). The 90% confidence intervals of the detected DEFs are marked except for those whose true FDRs exceed the target FDR by 10%. **(a)** target FDR < 0.01. **(b)** target FDR < 0.05.

**Simulation Test Results of *N* = 8,** $\boldsymbol{G}_{\mathbf{600}}^{\mathbf{600}}$


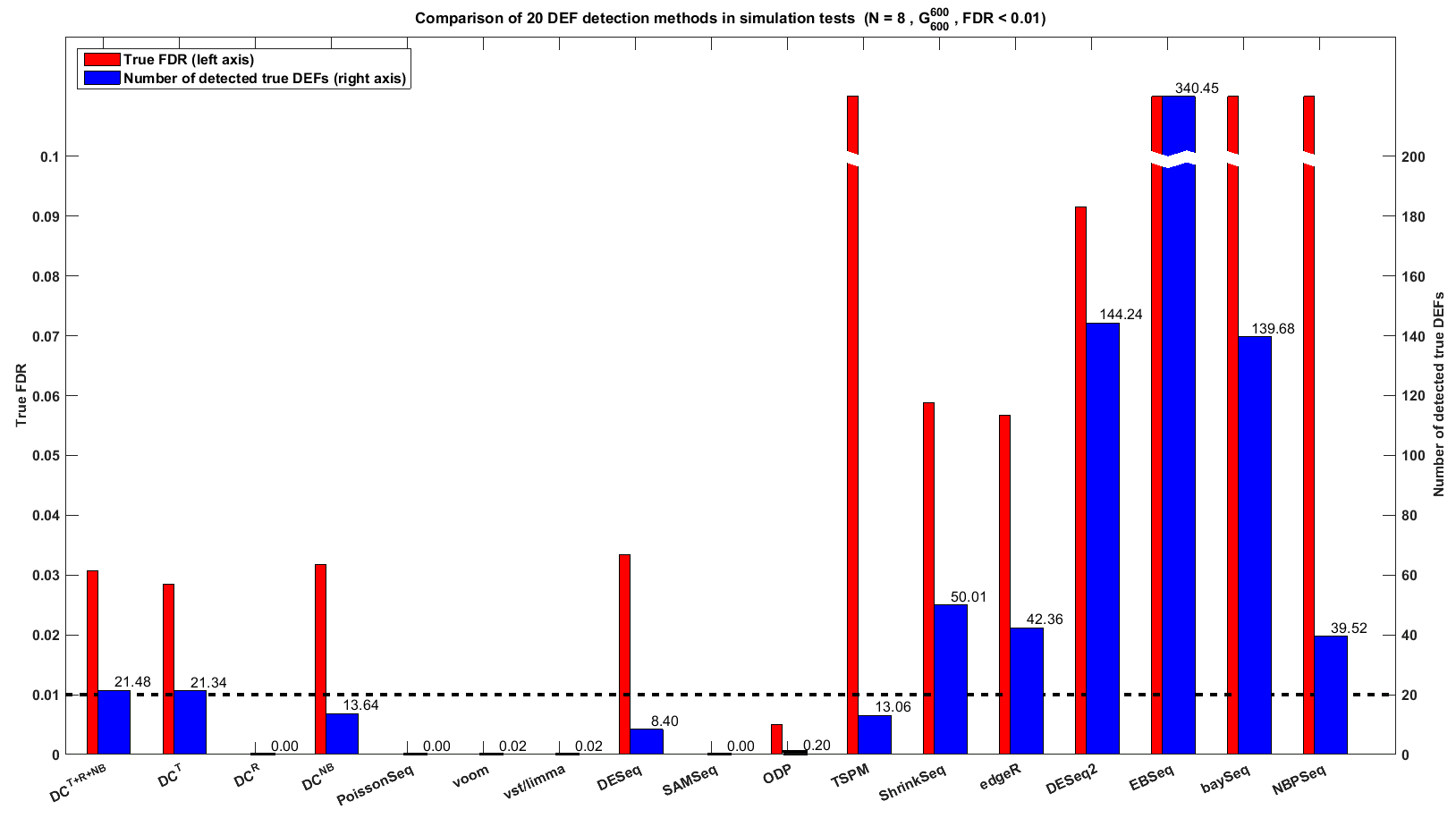

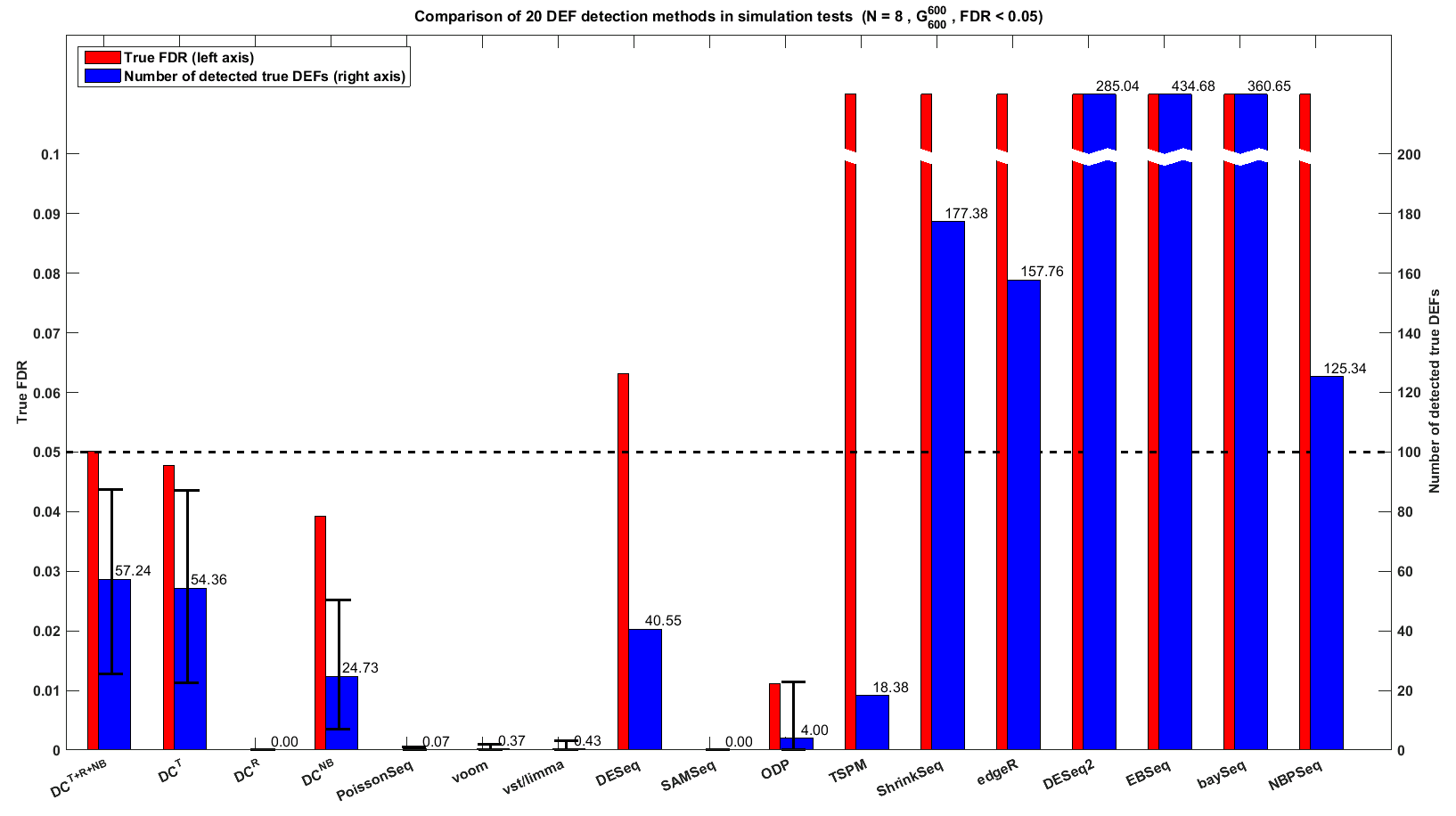


**(a) Comparison at target FDR < 0.01**

**(b) Comparison at target FDR < 0.05**

**True FDR**

**Number of detected true DEFs**

**Number of detected true DEFs**

**True FDR**

**Figure S6. Evaluates RNA-seq differential expression analysis methods using simulated data (4 *vs.* 4; $\boldsymbol{G}_{\boldsymbol{600}}^{\boldsymbol{600}}$)**. Methods are listed along the ***x***-axis. The red bars indicate the average true FDRs (refer to the left ***y***-axis). The horizontal dashed line across the figure marks the target FDR. The blue bars indicate the average number of the detected true DEFs (refer to the right ***y***-axis). The 90% confidence intervals of the detected DEFs are marked except for those whose true FDRs exceed the target FDR by 10%. **(a)** target FDR < 0.01. **(b)** target FDR < 0.05.

**Simulation Test Results of *N* = 10,** $\boldsymbol{G}_{\mathbf{600}}^{\mathbf{600}}$


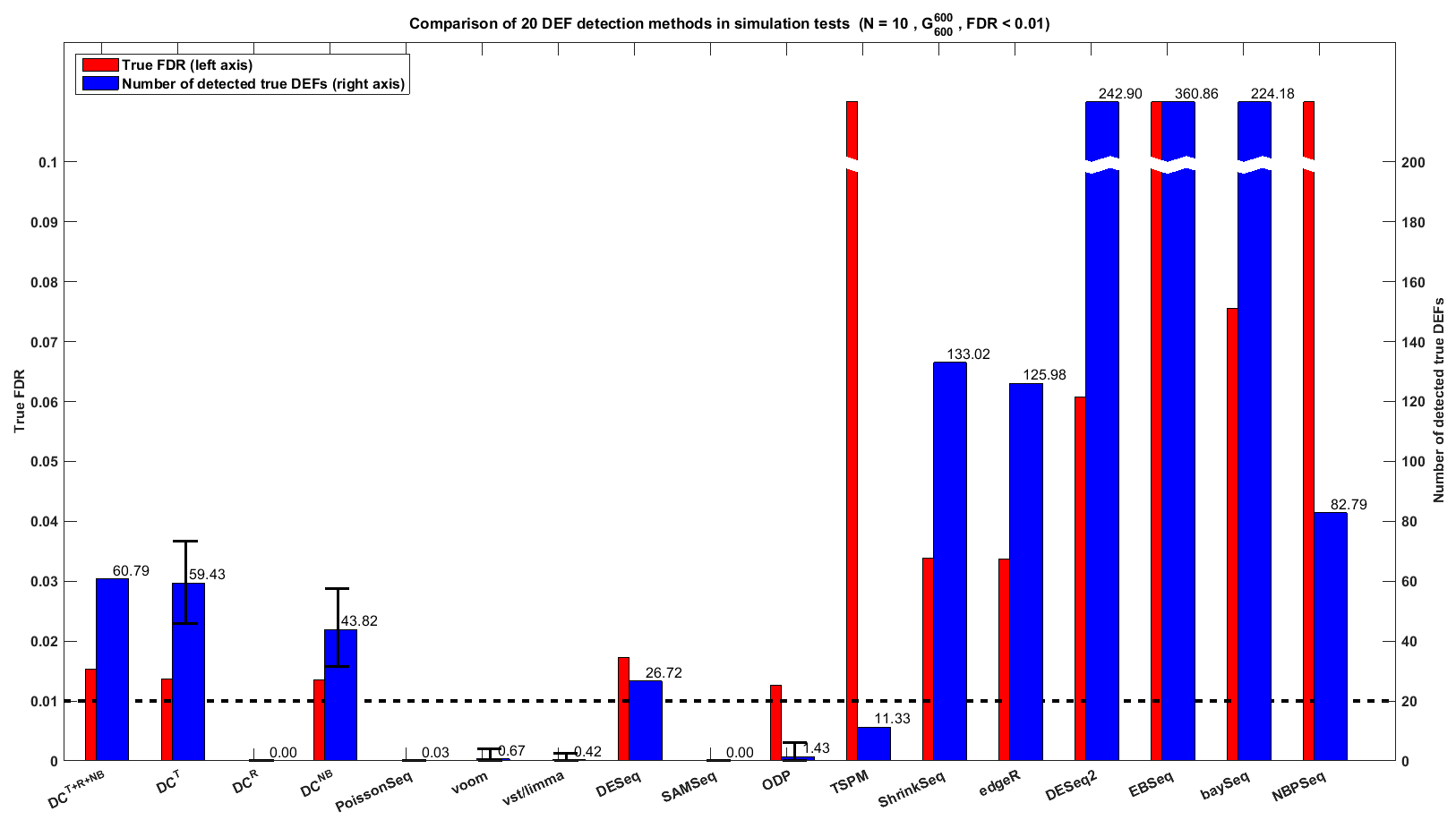

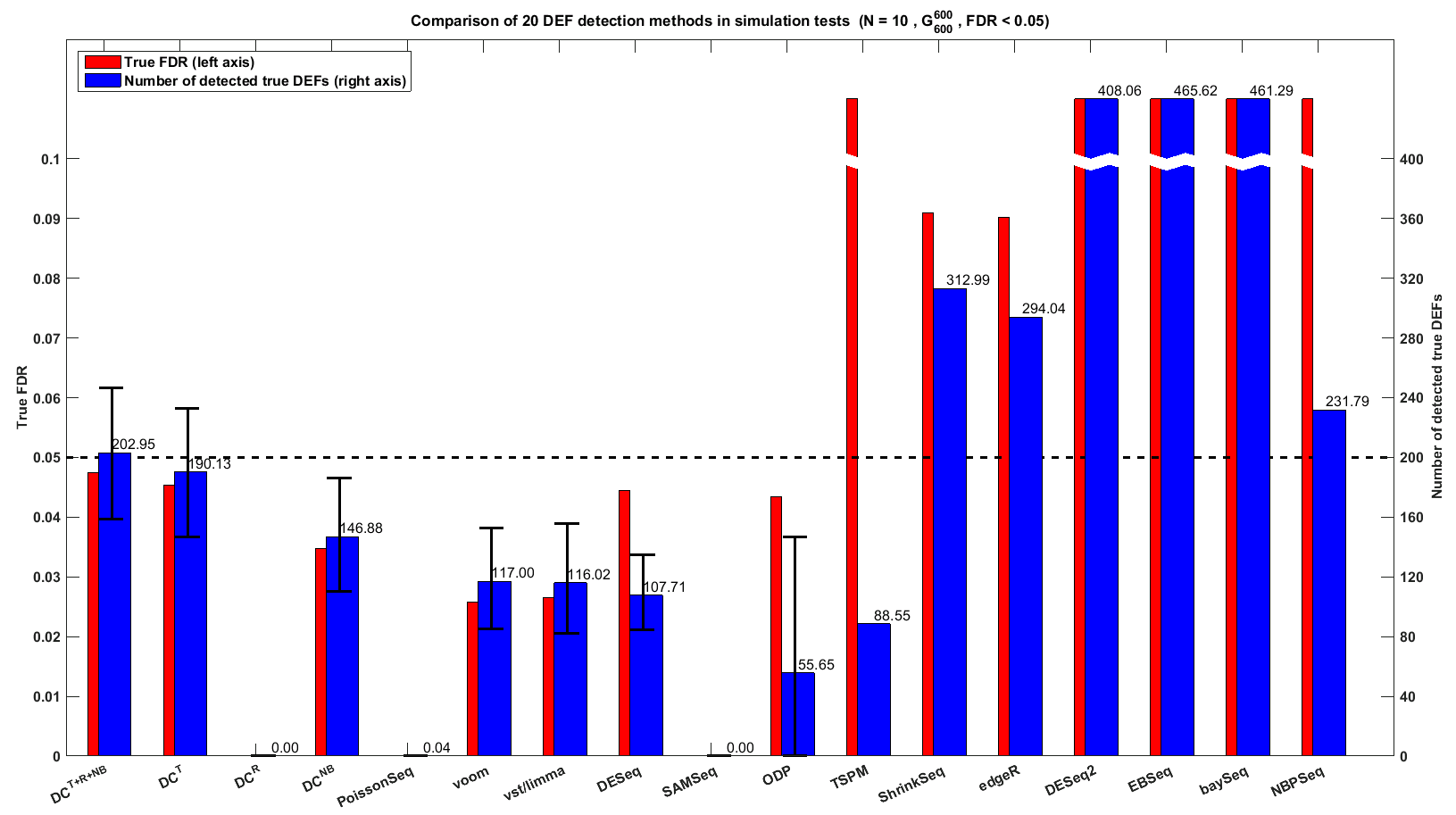


**(a) Comparison at target FDR < 0.01**

**(b) Comparison at target FDR < 0.05**

**True FDR**

**Number of detected true DEFs**

**Number of detected true DEFs**

**True FDR**

**Figure S7. Evaluates RNA-seq differential expression analysis methods using simulated data (5 *vs.* 5; $\boldsymbol{G}_{\boldsymbol{600}}^{\boldsymbol{600}}$)**. Methods are listed along the ***x***-axis. The red bars indicate the average true FDRs (refer to the left ***y***-axis). The horizontal dashed line across the figure marks the target FDR. The blue bars indicate the average number of the detected true DEFs (refer to the right ***y***-axis). The 90% confidence intervals of the detected DEFs are marked except for those whose true FDRs exceed the target FDR by 10%. **(a)** target FDR < 0.01. **(b)** target FDR < 0.05.

**Simulation Test Results of *N* = 12,** $\boldsymbol{G}_{\mathbf{600}}^{\mathbf{600}}$


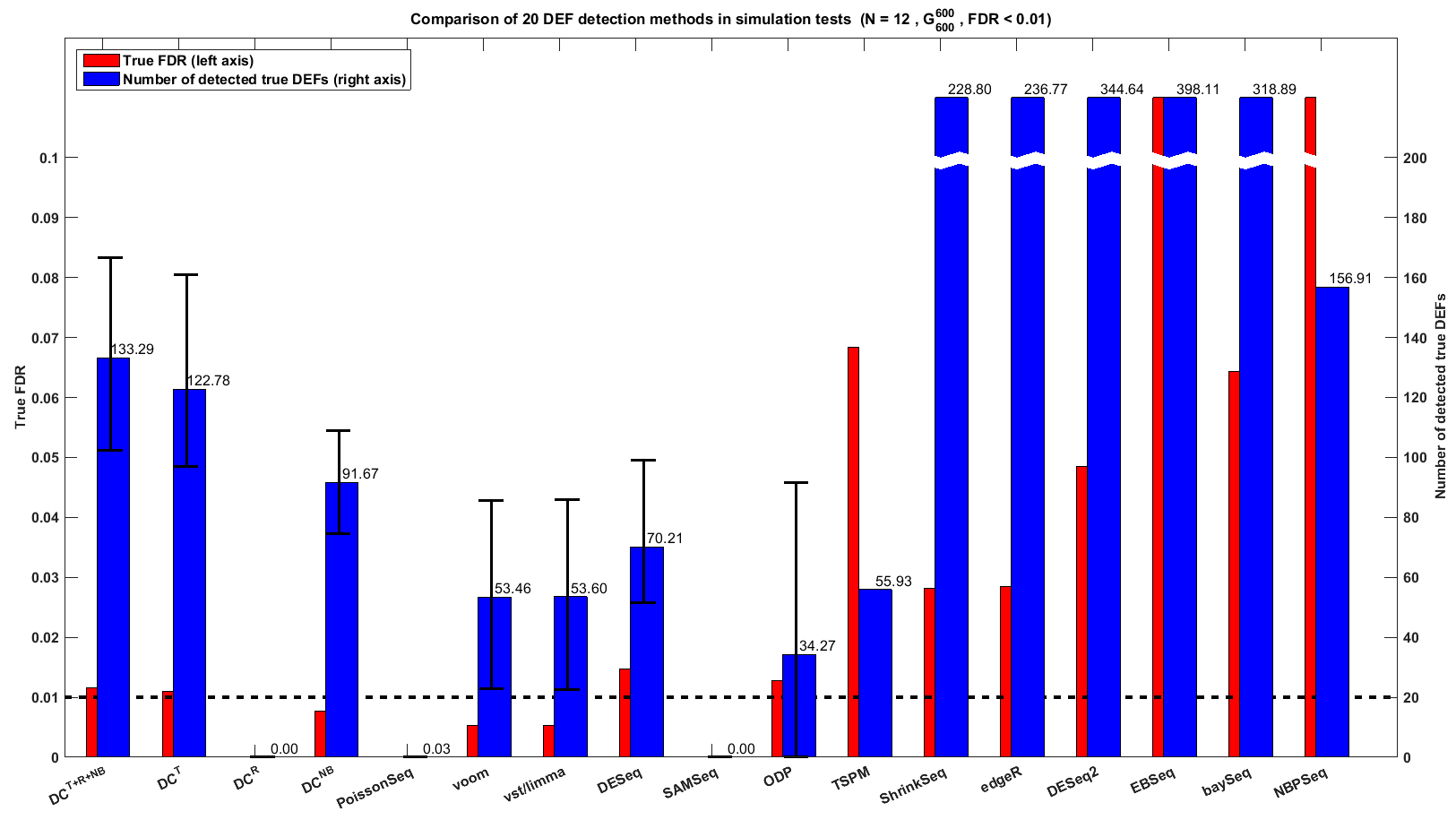

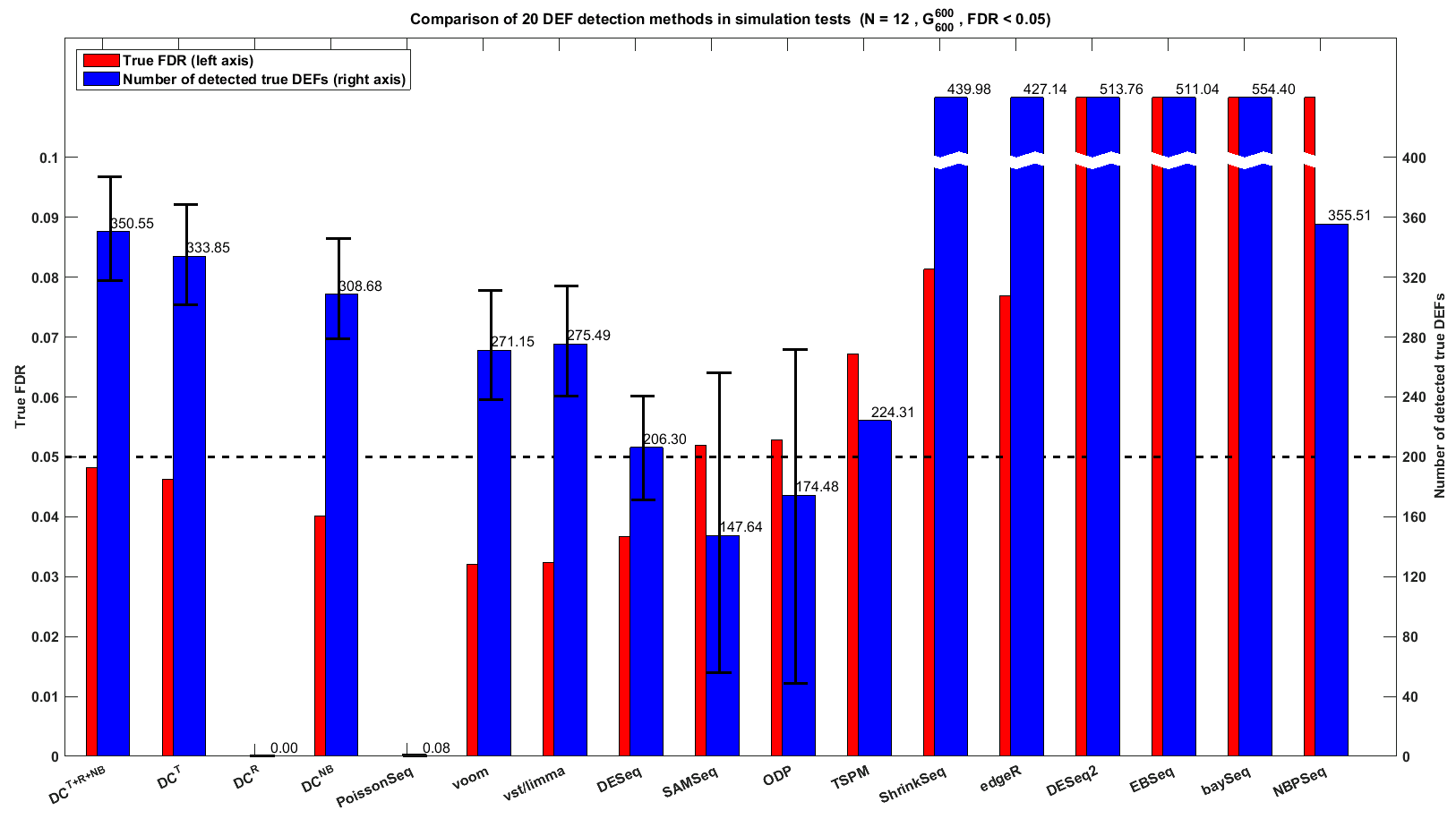


**(a) Comparison at target FDR < 0.01**

**(b) Comparison at target FDR < 0.05**

**True FDR**

**Number of detected true DEFs**

**Number of detected true DEFs**

**True FDR**

**Figure S8. Evaluates RNA-seq differential expression analysis methods using simulated data (6 *vs.* 6; $\boldsymbol{G}_{\boldsymbol{600}}^{\boldsymbol{600}}$)**. Methods are listed along the ***x***-axis. The red bars indicate the average true FDRs (refer to the left ***y***-axis). The horizontal dashed line across the figure marks the target FDR. The blue bars indicate the average number of the detected true DEFs (refer to the right ***y***-axis). The 90% confidence intervals of the detected DEFs are marked except for those whose true FDRs exceed the target FDR by 10%. **(a)** target FDR < 0.01. **(b)** target FDR < 0.05.

**Simulation Test Results of *N* = 16,** $\boldsymbol{G}_{\mathbf{600}}^{\mathbf{600}}$


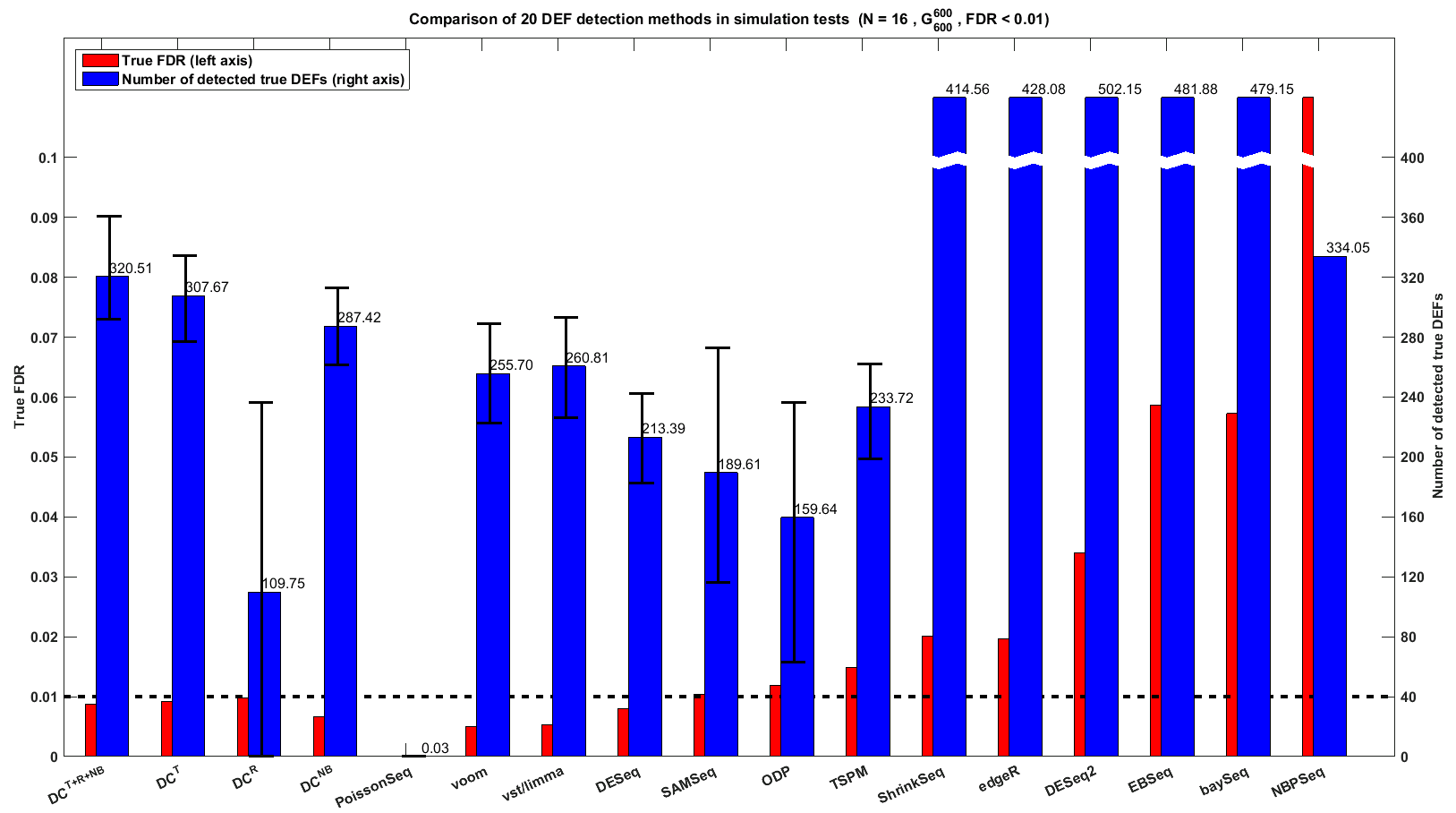

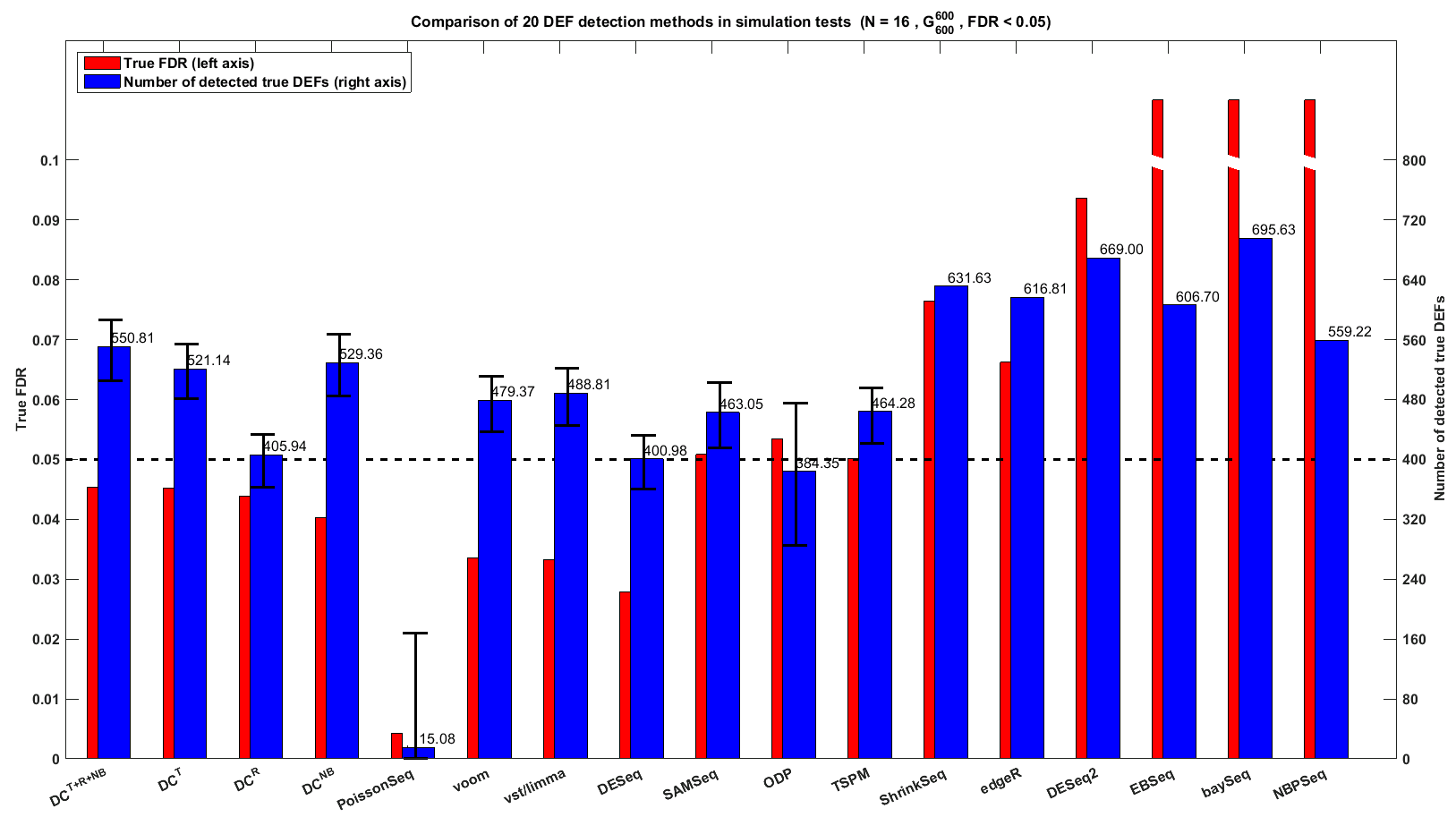


**(a) Comparison at target FDR < 0.01**

**(b) Comparison at target FDR < 0.05**

**True FDR**

**Number of detected true DEFs**

**Number of detected true DEFs**

**True FDR**

**Figure S9. Evaluates RNA-seq differential expression analysis methods using simulated data (8 *vs.* 8; $\boldsymbol{G}_{\boldsymbol{600}}^{\boldsymbol{600}}$)**. Methods are listed along the ***x***-axis. The red bars indicate the average true FDRs (refer to the left ***y***-axis). The horizontal dashed line across the figure marks the target FDR. The blue bars indicate the average number of the detected true DEFs (refer to the right ***y***-axis). The 90% confidence intervals of the detected DEFs are marked except for those whose true FDRs exceed the target FDR by 10%. **(a)** target FDR < 0.01. **(b)** target FDR < 0.05.

**Simulation Test Results of *N* = 20,** $\boldsymbol{G}_{\mathbf{600}}^{\mathbf{600}}$


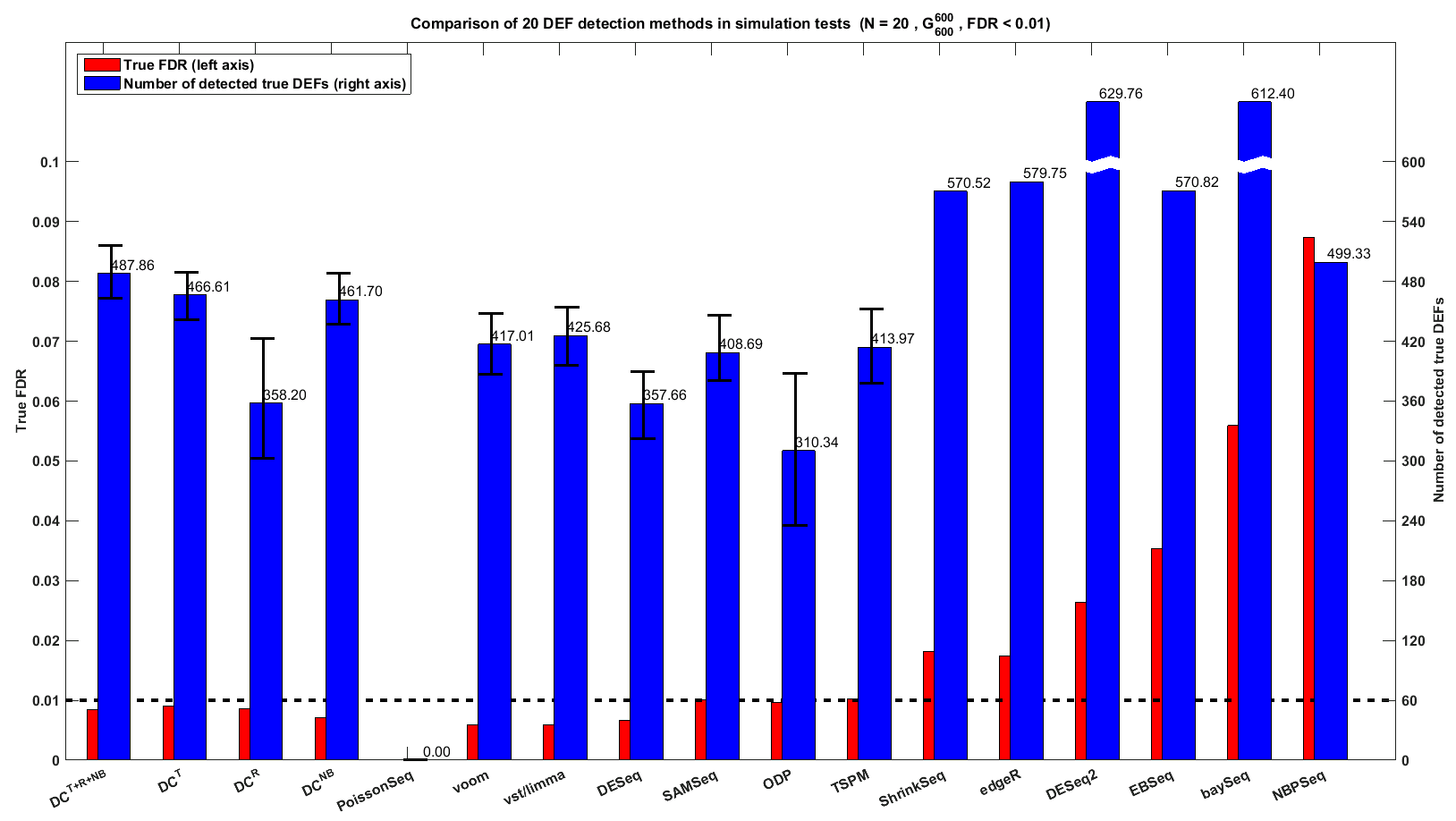

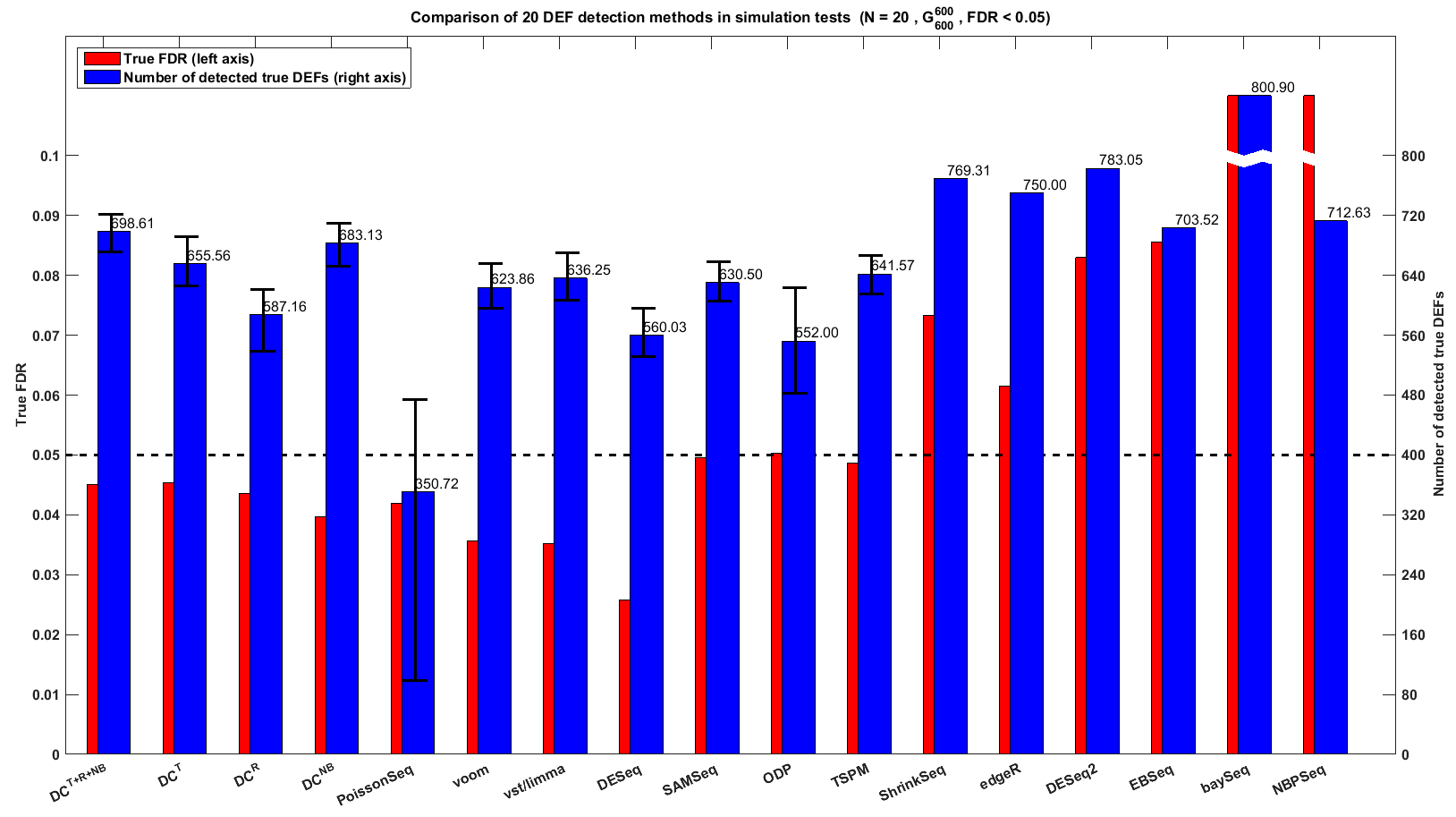


**(a) Comparison at target FDR < 0.01**

**(b) Comparison at target FDR < 0.05**

**True FDR**

**Number of detected true DEFs**

**Number of detected true DEFs**

**True FDR**

**Figure S10. Evaluates RNA-seq differential expression analysis methods using simulated data (10 *vs.* 10; $\boldsymbol{G}_{\boldsymbol{600}}^{\boldsymbol{600}}$)**. Methods are listed along the ***x***-axis. The red bars indicate the average true FDRs (refer to the left ***y***-axis). The horizontal dashed line across the figure marks the target FDR. The blue bars indicate the average number of the detected true DEFs (refer to the right ***y***-axis). The 90% confidence intervals of the detected DEFs are marked except for those whose true FDRs exceed the target FDR by 10%. **(a)** target FDR < 0.01. **(b)** target FDR < 0.05.

**Simulation Test Results of *N* = 8,** $\boldsymbol{G}_{\mathbf{400}}^{\mathbf{400}}$


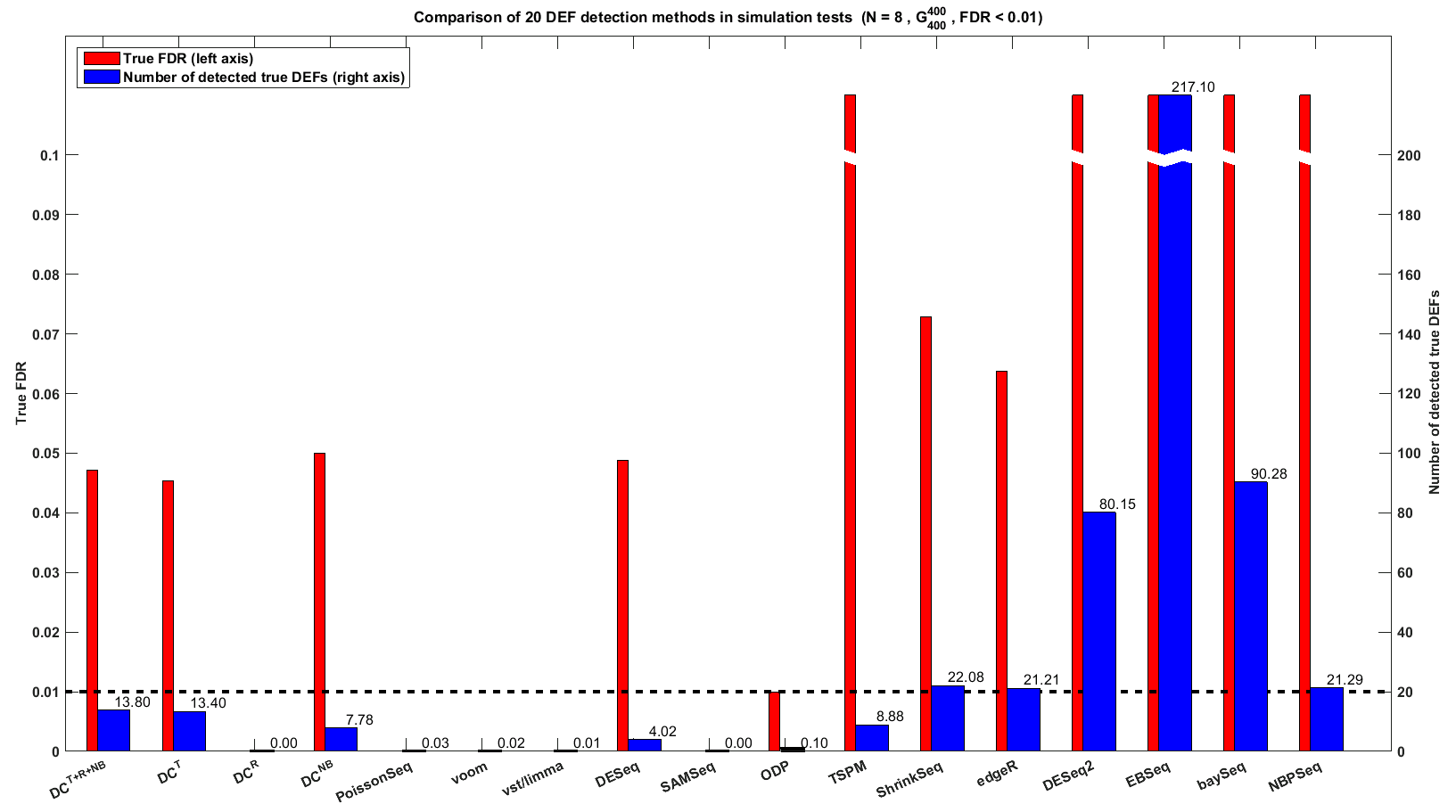

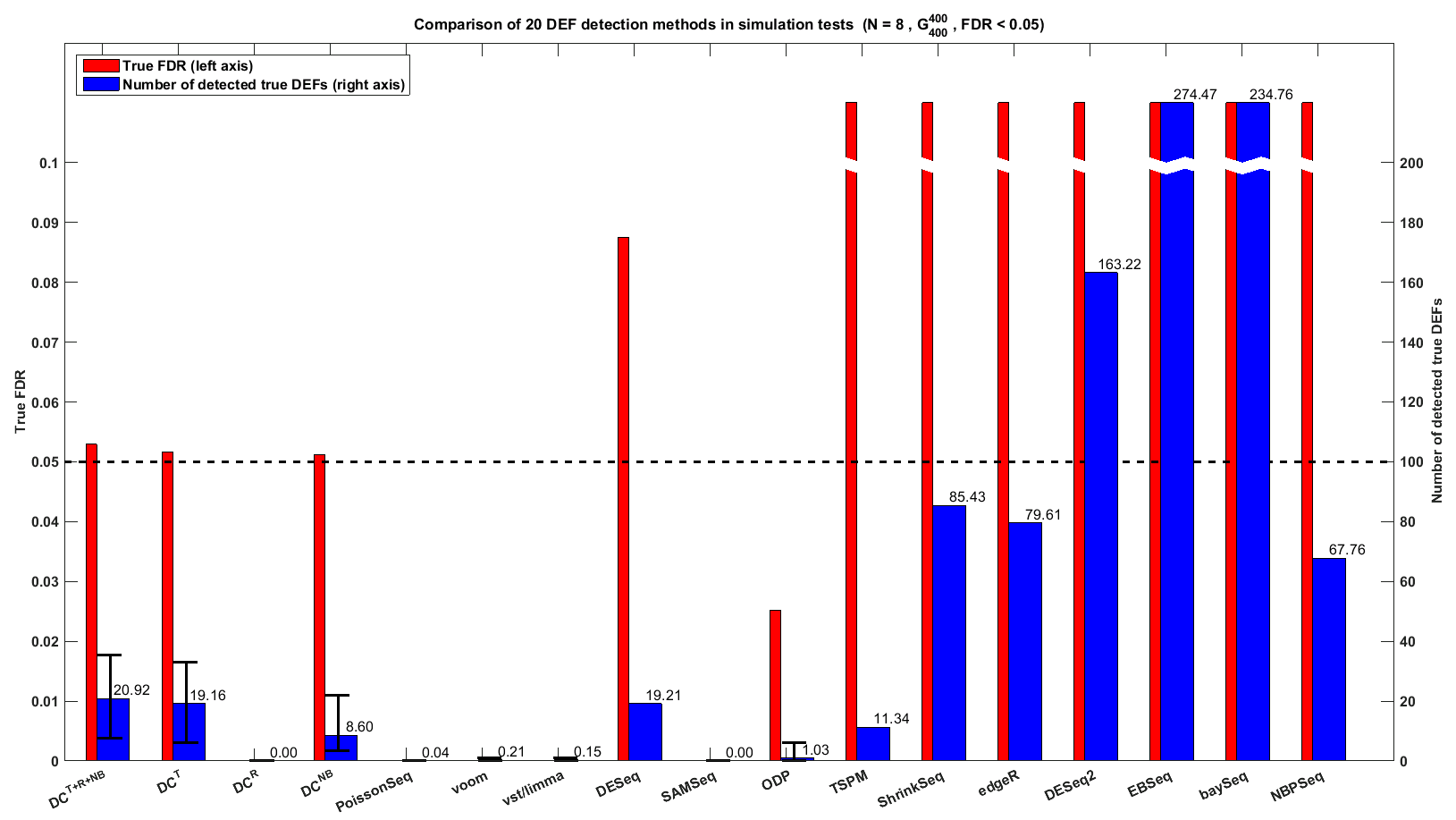


**(a) Comparison at target FDR < 0.01**

**(b) Comparison at target FDR < 0.05**

**True FDR**

**Number of detected true DEFs**

**Number of detected true DEFs**

**True FDR**

**Figure S11. Evaluates RNA-seq differential expression analysis methods using simulated data (4 *vs.* 4; $\boldsymbol{G}_{\boldsymbol{400}}^{\boldsymbol{400}}$)**. Methods are listed along the ***x***-axis. The red bars indicate the average true FDRs (refer to the left ***y***-axis). The horizontal dashed line across the figure marks the target FDR. The blue bars indicate the average number of the detected true DEFs (refer to the right ***y***-axis). The 90% confidence intervals of the detected DEFs are marked except for those whose true FDRs exceed the target FDR by 10%. **(a)** target FDR < 0.01. **(b)** target FDR < 0.05.

**Simulation Test Results of *N* = 10,** $\boldsymbol{G}_{\mathbf{400}}^{\mathbf{400}}$


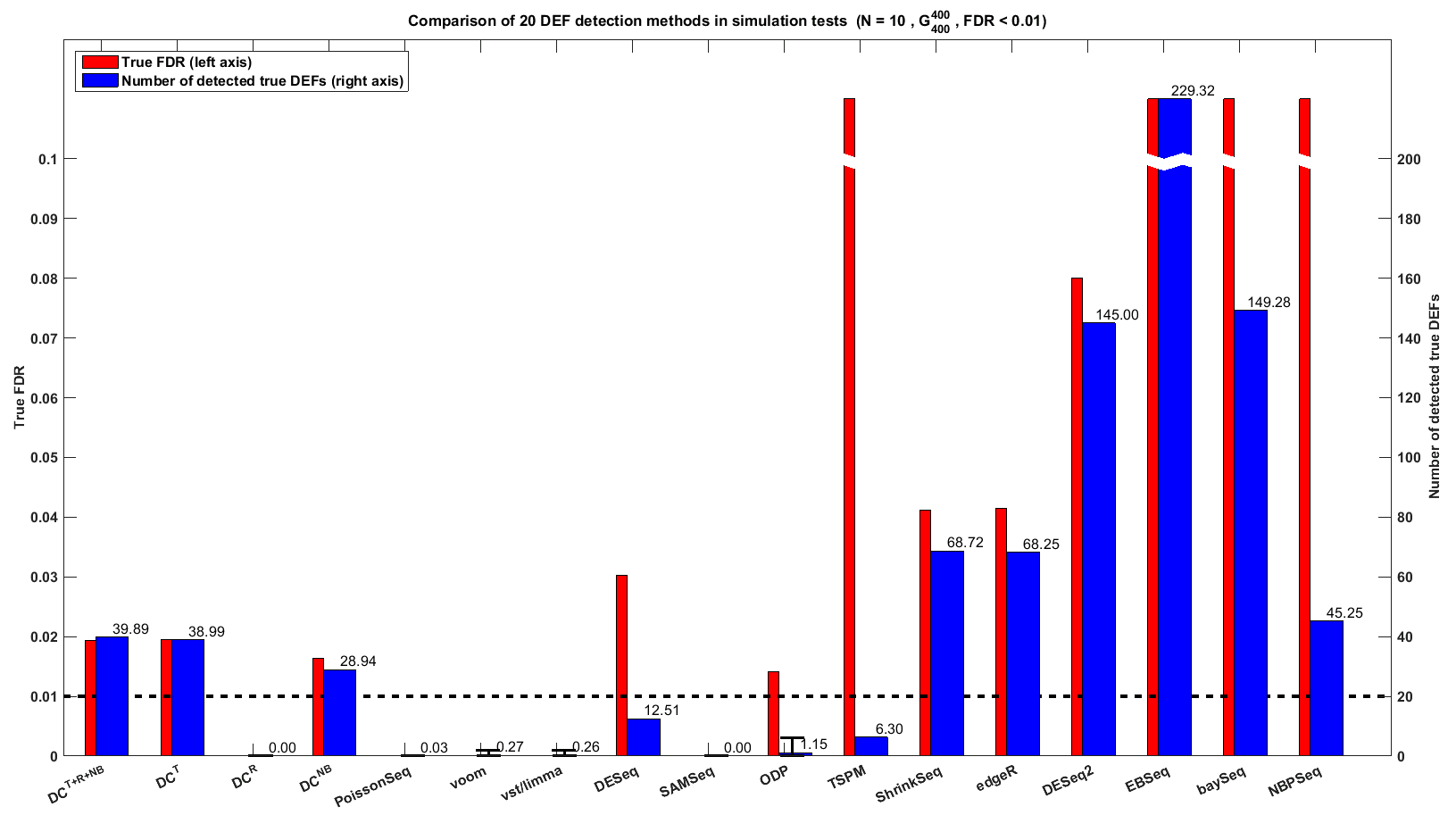

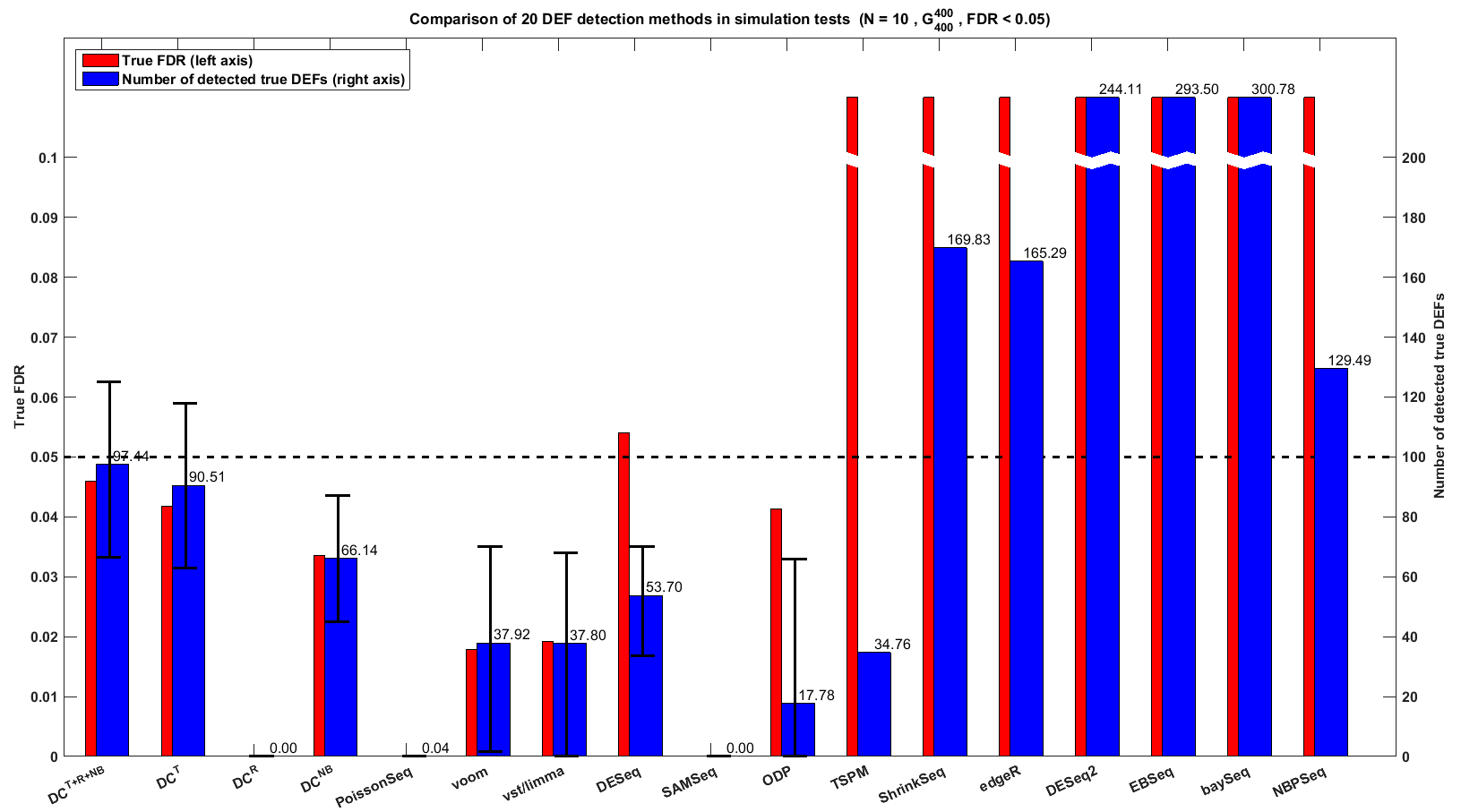


**(a) Comparison at target FDR < 0.01**

**(b) Comparison at target FDR < 0.05**

**True FDR**

**Number of detected true DEFs**

**Number of detected true DEFs**

**True FDR**

**Figure S12. Evaluates RNA-seq differential expression analysis methods using simulated data (5 *vs.* 5; $\boldsymbol{G}_{\boldsymbol{400}}^{\boldsymbol{400}}$)**. Methods are listed along the ***x***-axis. The red bars indicate the average true FDRs (refer to the left ***y***-axis). The horizontal dashed line across the figure marks the target FDR. The blue bars indicate the average number of the detected true DEFs (refer to the right ***y***-axis). The 90% confidence intervals of the detected DEFs are marked except for those whose true FDRs exceed the target FDR by 10%. **(a)** target FDR < 0.01. **(b)** target FDR < 0.05.

**Simulation Test Results of *N* = 12,** $\boldsymbol{G}_{\mathbf{400}}^{\mathbf{400}}$


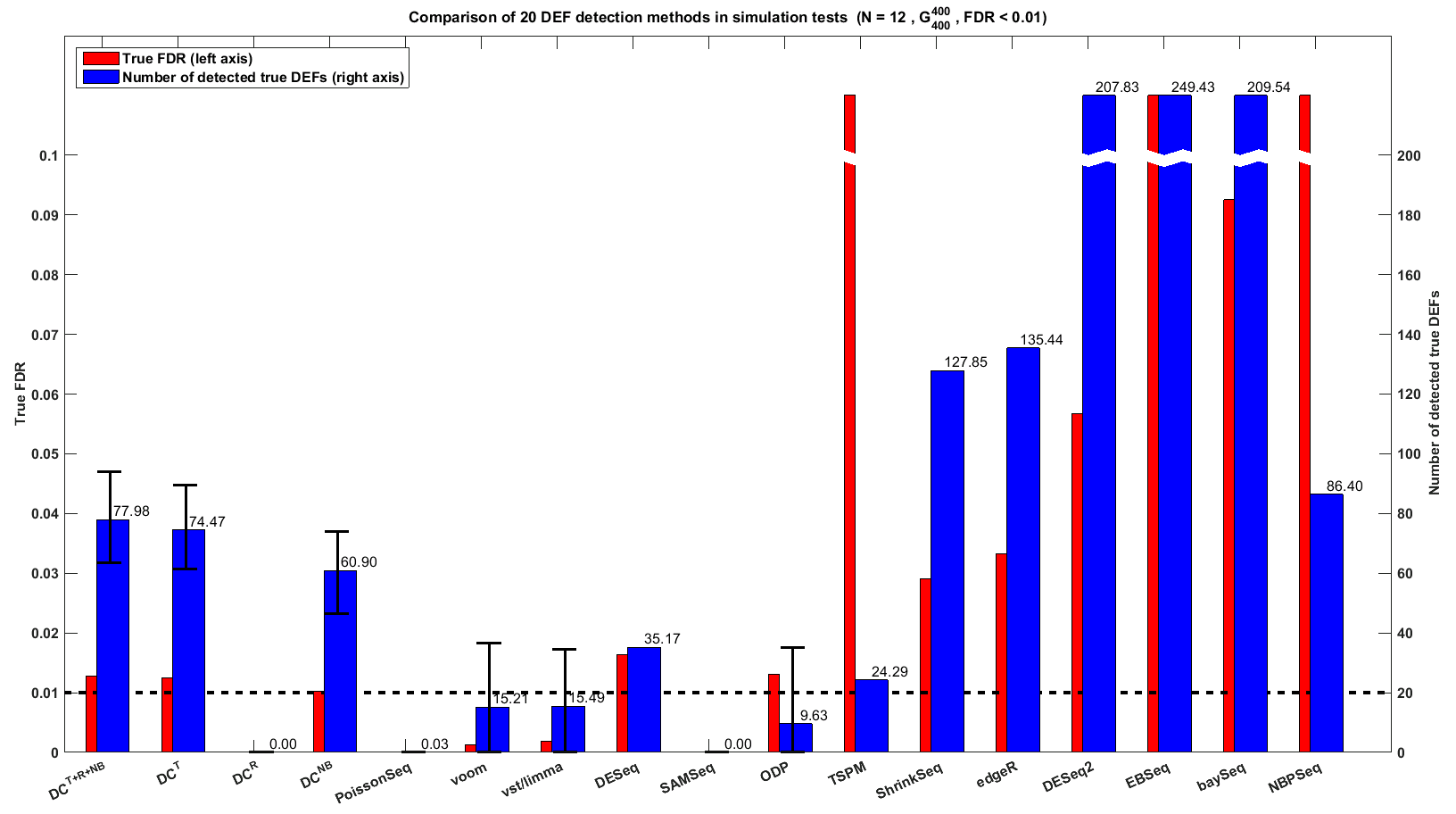

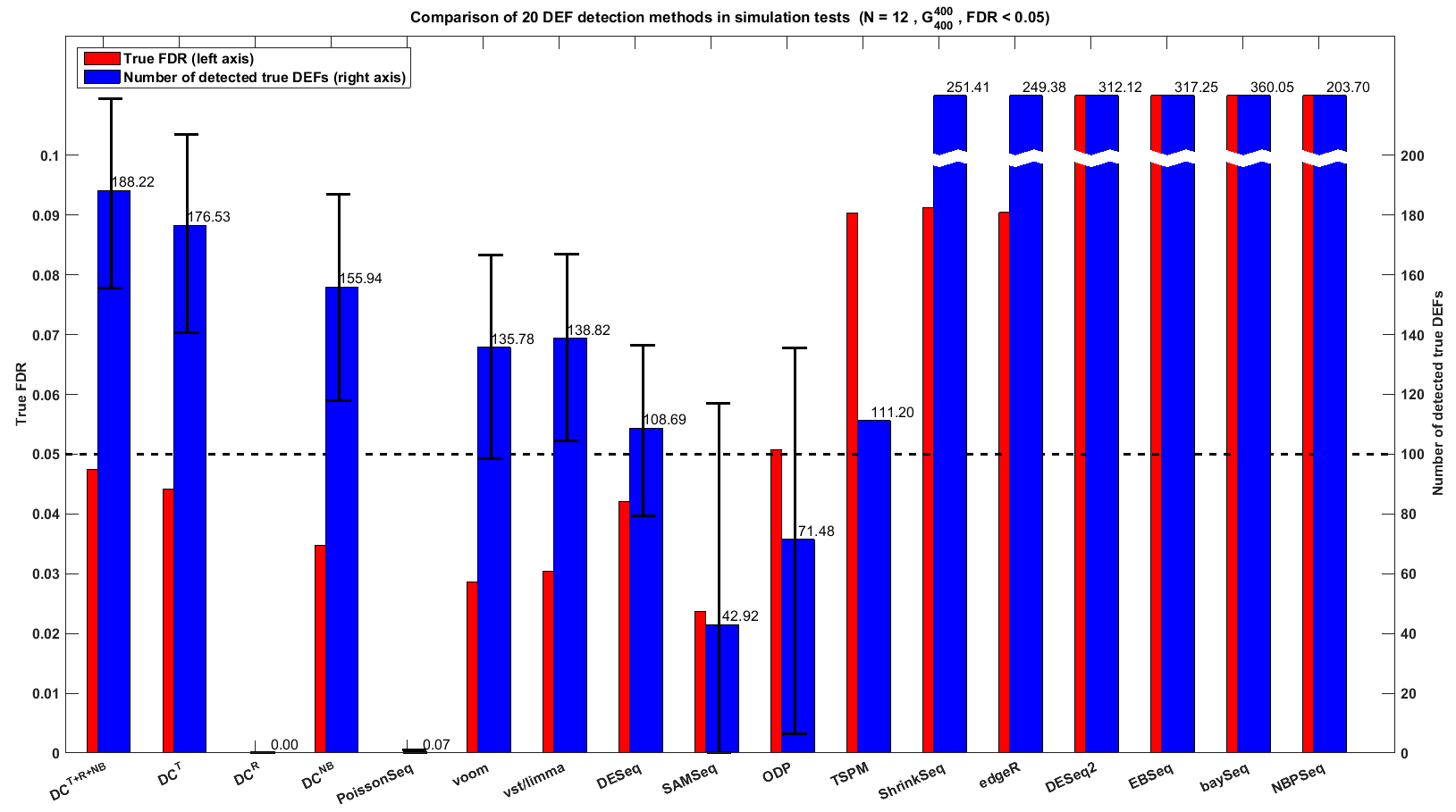


**(a) Comparison at target FDR < 0.01**

**(b) Comparison at target FDR < 0.05**

**True FDR**

**Number of detected true DEFs**

**Number of detected true DEFs**

**True FDR**

**Figure S13. Evaluates RNA-seq differential expression analysis methods using simulated data (6 *vs.* 6; $\boldsymbol{G}_{\boldsymbol{400}}^{\boldsymbol{400}}$)**. Methods are listed along the ***x***-axis. The red bars indicate the average true FDRs (refer to the left ***y***-axis). The horizontal dashed line across the figure marks the target FDR. The blue bars indicate the average number of the detected true DEFs (refer to the right ***y***-axis). The 90% confidence intervals of the detected DEFs are marked except for those whose true FDRs exceed the target FDR by 10%. **(a)** target FDR < 0.01. **(b)** target FDR < 0.05.

**Simulation Test Results of *N* = 16,** $\boldsymbol{G}_{\mathbf{400}}^{\mathbf{400}}$


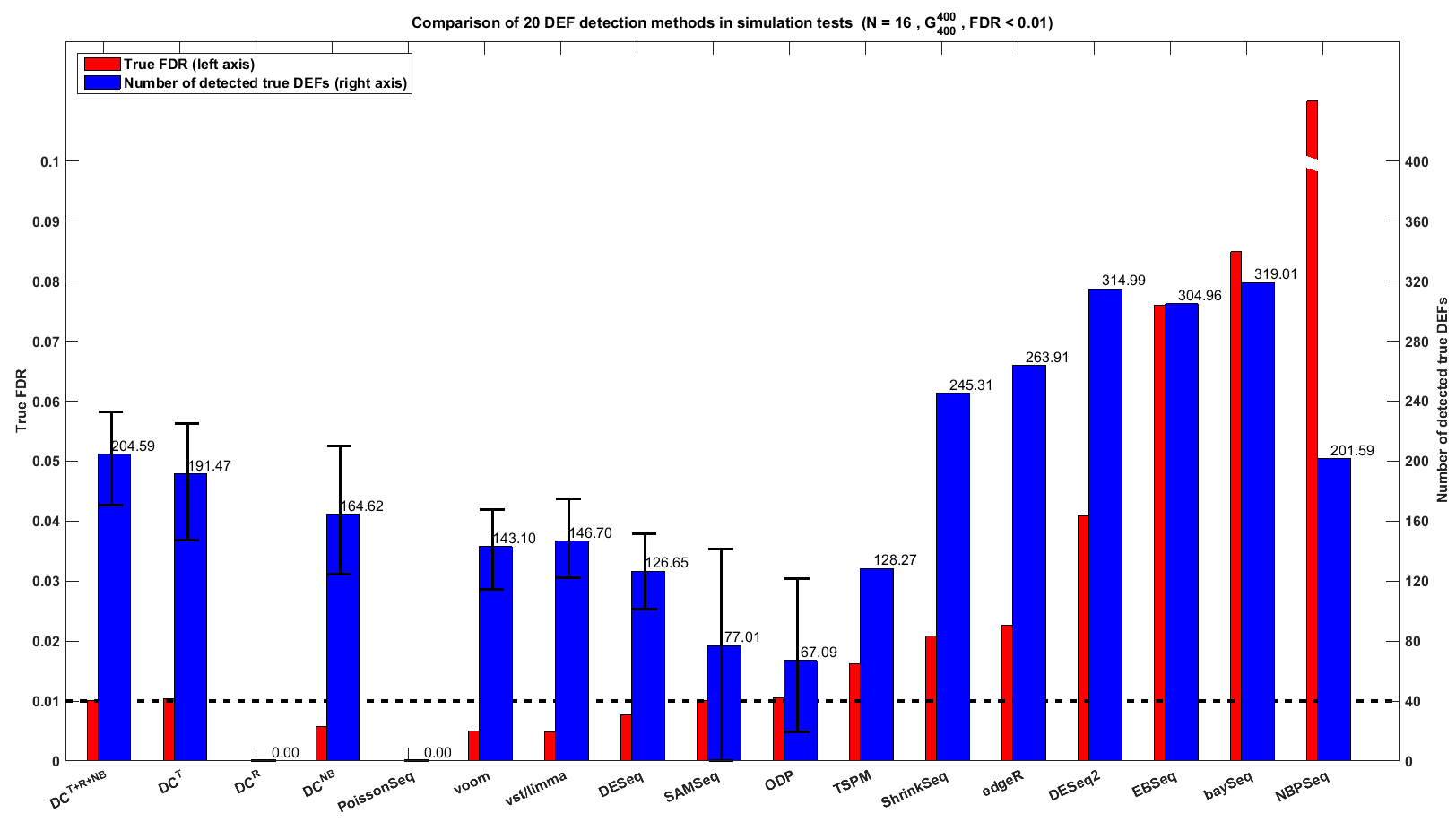

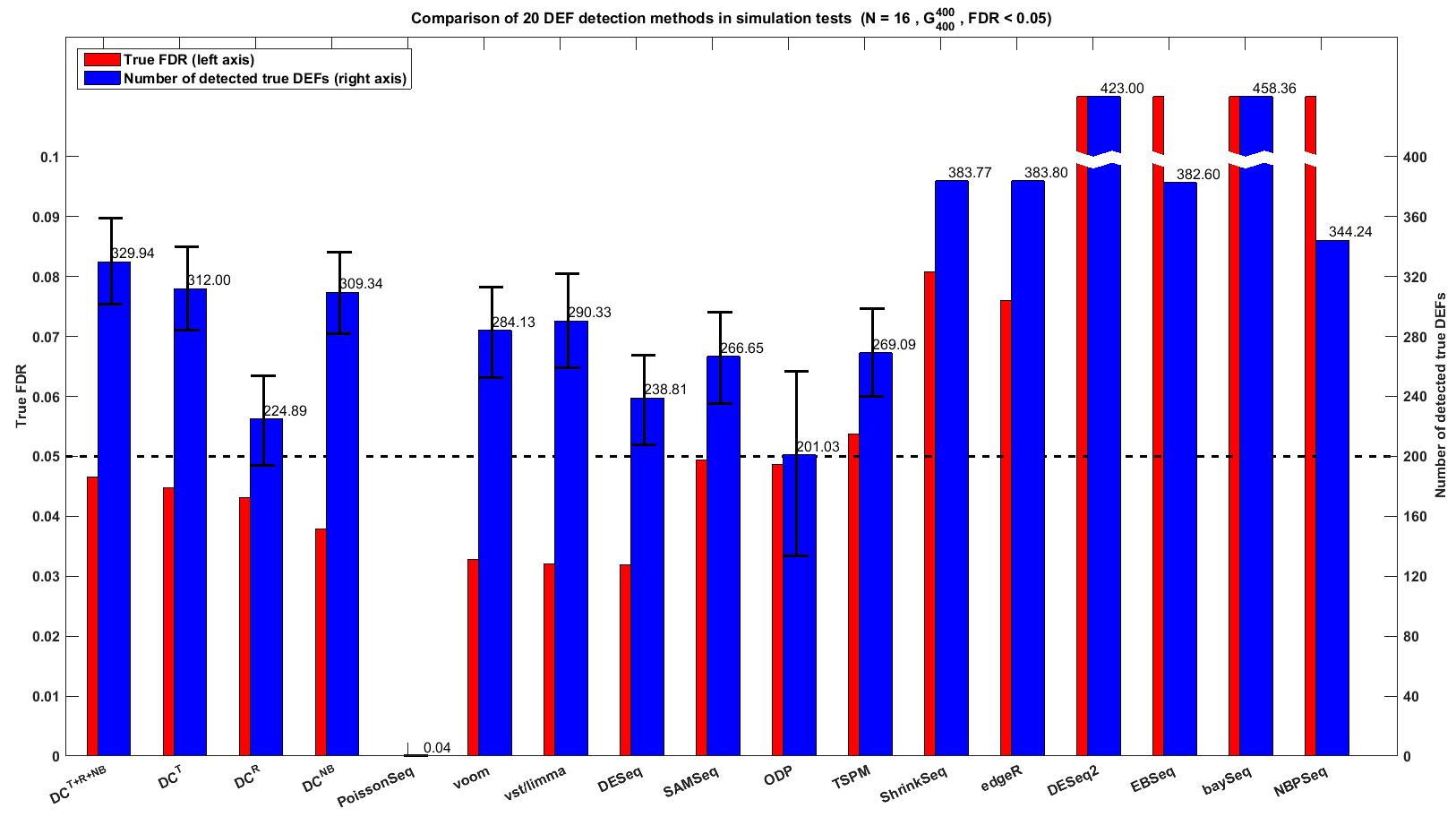


**(a) Comparison at target FDR < 0.01**

**(b) Comparison at target FDR < 0.05**

**True FDR**

**Number of detected true DEFs**

**Number of detected true DEFs**

**True FDR**

**Figure S14. Evaluates RNA-seq differential expression analysis methods using simulated data (8 *vs.* 8; $\boldsymbol{G}_{\boldsymbol{400}}^{\boldsymbol{400}}$)**. Methods are listed along the ***x***-axis. The red bars indicate the average true FDRs (refer to the left ***y***-axis). The horizontal dashed line across the figure marks the target FDR. The blue bars indicate the average number of the detected true DEFs (refer to the right ***y***-axis). The 90% confidence intervals of the detected DEFs are marked except for those whose true FDRs exceed the target FDR by 10%. **(a)** target FDR < 0.01. **(b)** target FDR < 0.05.

**Simulation Test Results of *N* = 20,** $\boldsymbol{G}_{\mathbf{400}}^{\mathbf{400}}$


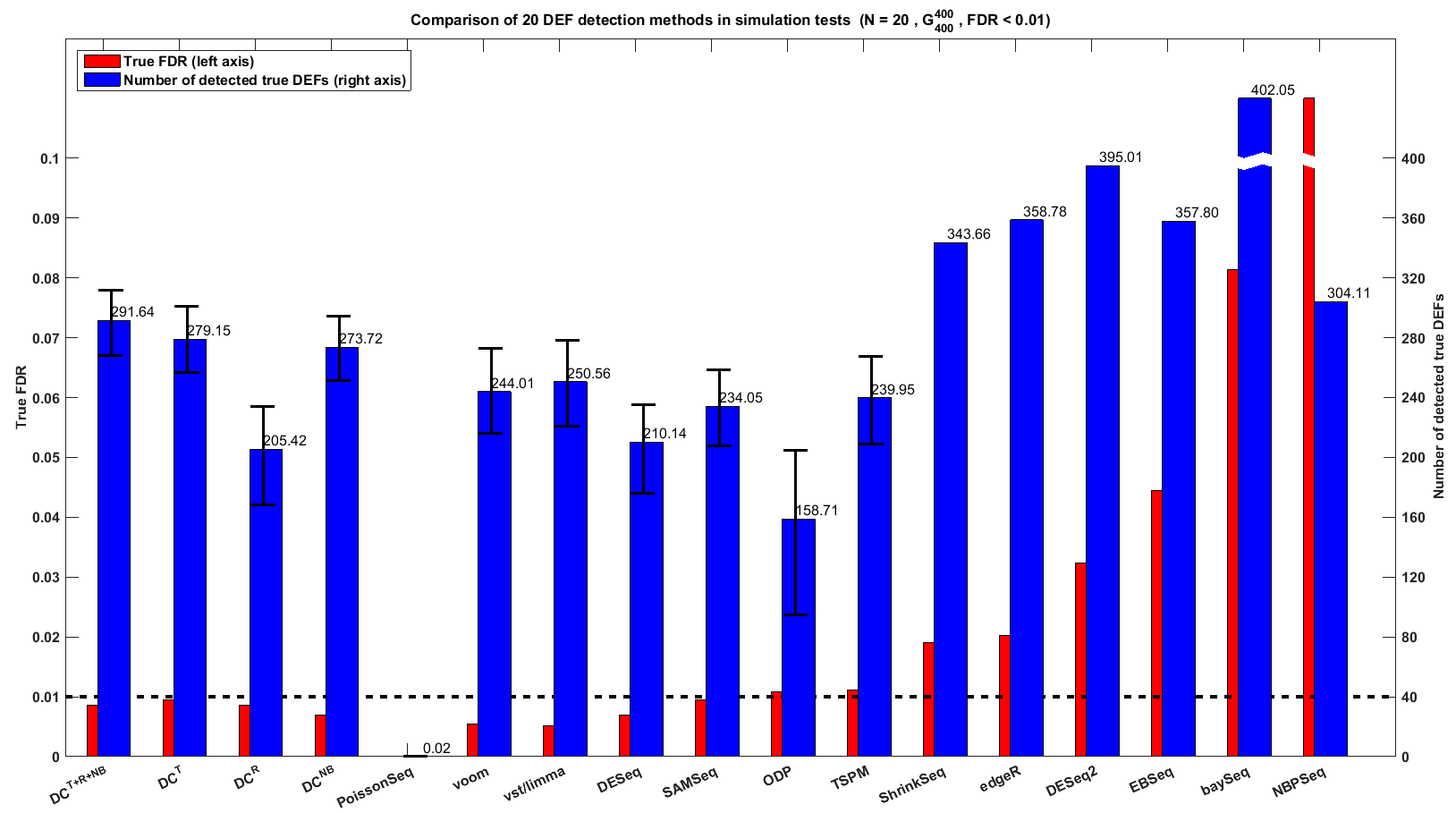

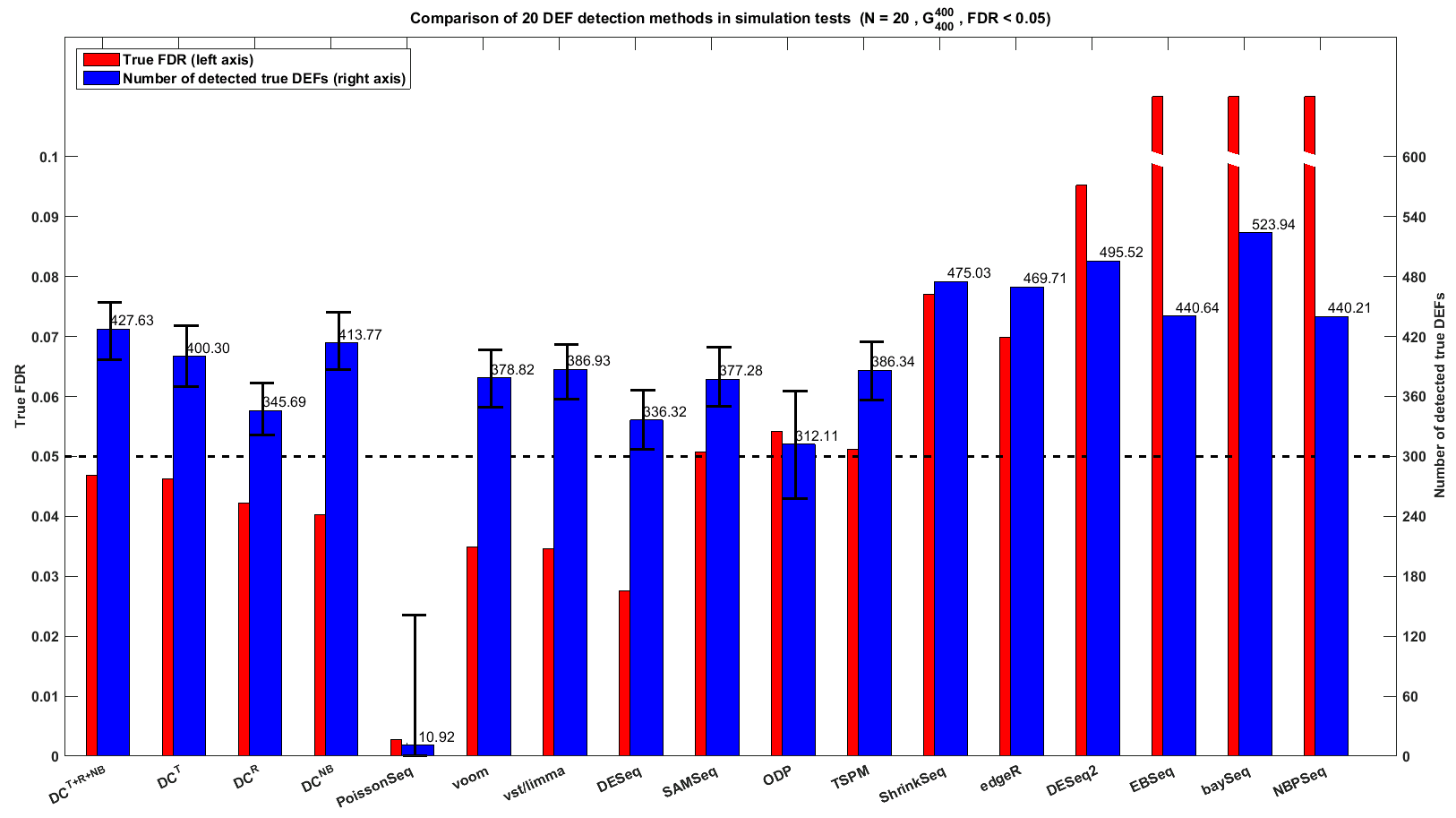


**(a) Comparison at target FDR < 0.01**

**(b) Comparison at target FDR < 0.05**

**True FDR**

**Number of detected true DEFs**

**Number of detected true DEFs**

**True FDR**

**Figure S15. Evaluates RNA-seq differential expression analysis methods using simulated data (10 *vs.* 10; $\boldsymbol{G}_{\boldsymbol{400}}^{\boldsymbol{400}}$)**. Methods are listed along the ***x***-axis. The red bars indicate the average true FDRs (refer to the left ***y***-axis). The horizontal dashed line across the figure marks the target FDR. The blue bars indicate the average number of the detected true DEFs (refer to the right ***y***-axis). The 90% confidence intervals of the detected DEFs are marked except for those whose true FDRs exceed the target FDR by 10%. **(a)** target FDR < 0.01. **(b)** target FDR < 0.05.

**Simulation Test Results of *N* = 8,** $\boldsymbol{G}_{\mathbf{0}}^{\mathbf{1000}}$


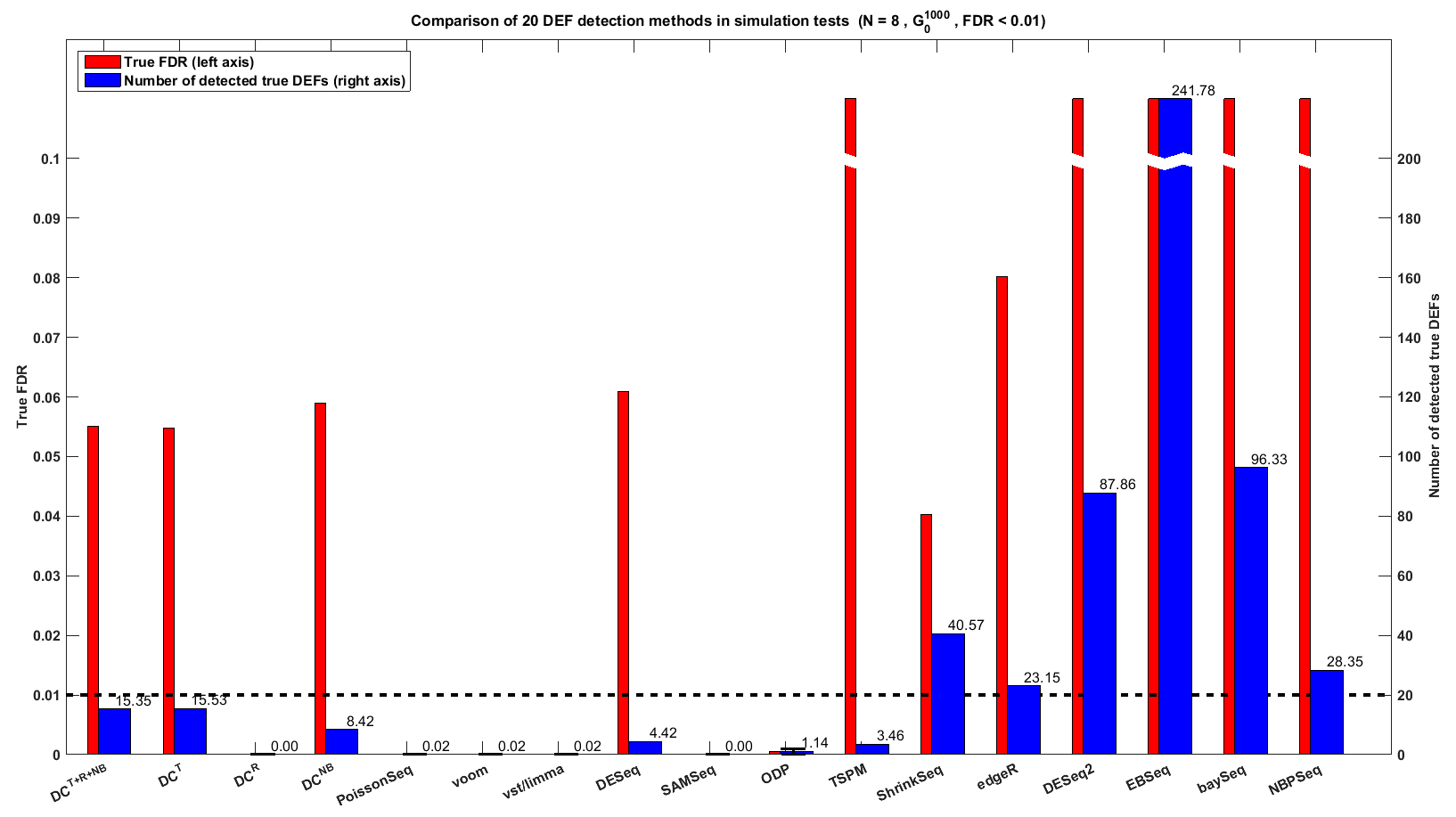

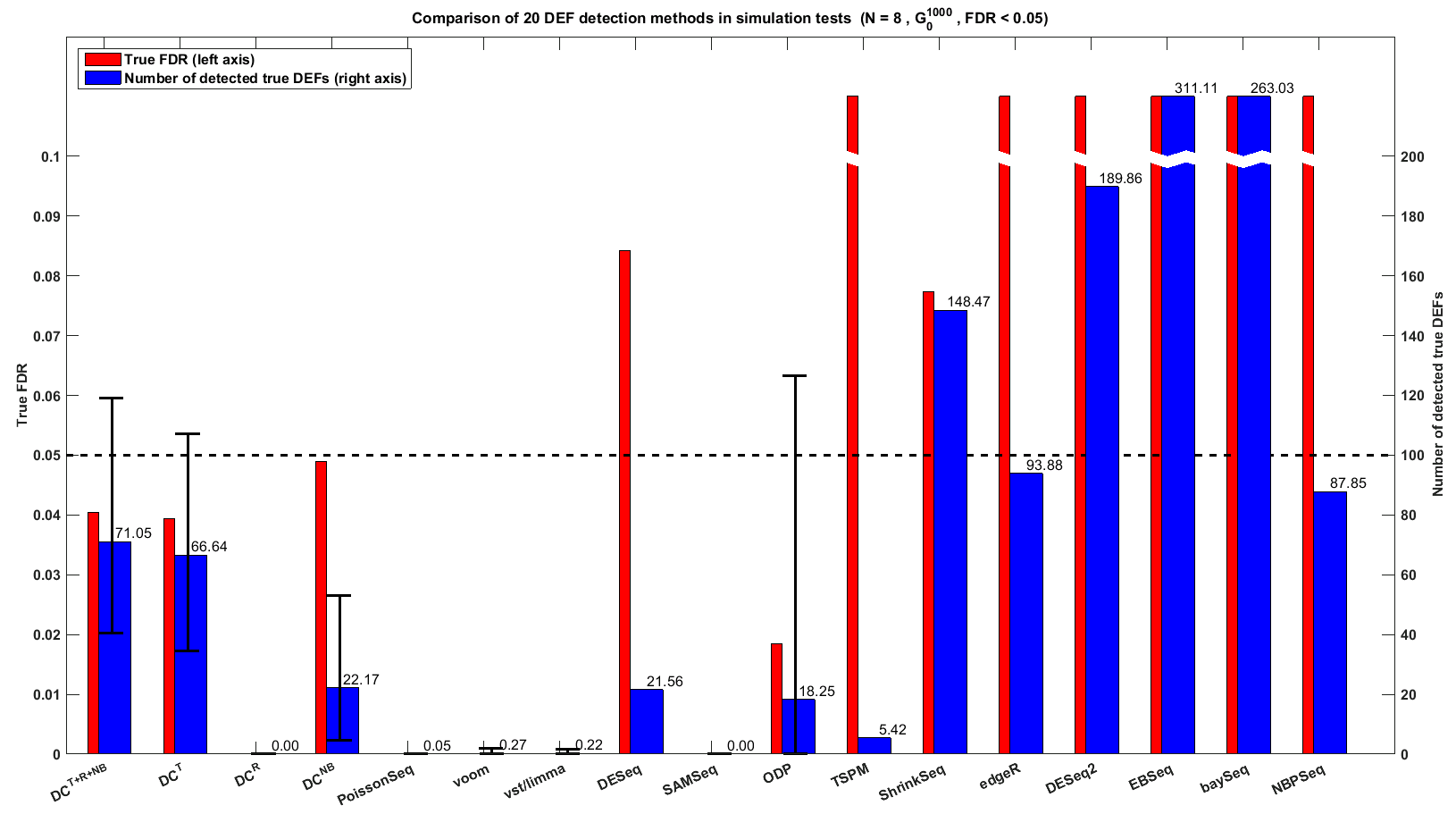


**(a) Comparison at target FDR < 0.01**

**(b) Comparison at target FDR < 0.05**

**True FDR**

**Number of detected true DEFs**

**Number of detected true DEFs**

**True FDR**

**Figure S16. Evaluates RNA-seq differential expression analysis methods using simulated data (4 *vs.* 4; $\boldsymbol{G}_{\boldsymbol{0}}^{\boldsymbol{1000}}$)**. Methods are listed along the ***x***-axis. The red bars indicate the average true FDRs (refer to the left ***y***-axis). The horizontal dashed line across the figure marks the target FDR. The blue bars indicate the average number of the detected true DEFs (refer to the right ***y***-axis). The 90% confidence intervals of the detected DEFs are marked except for those whose true FDRs exceed the target FDR by 10%. **(a)** target FDR < 0.01. **(b)** target FDR < 0.05.

**Simulation Test Results of *N* = 10,** $\boldsymbol{G}_{\boldsymbol{0}}^{\boldsymbol{1000}}$


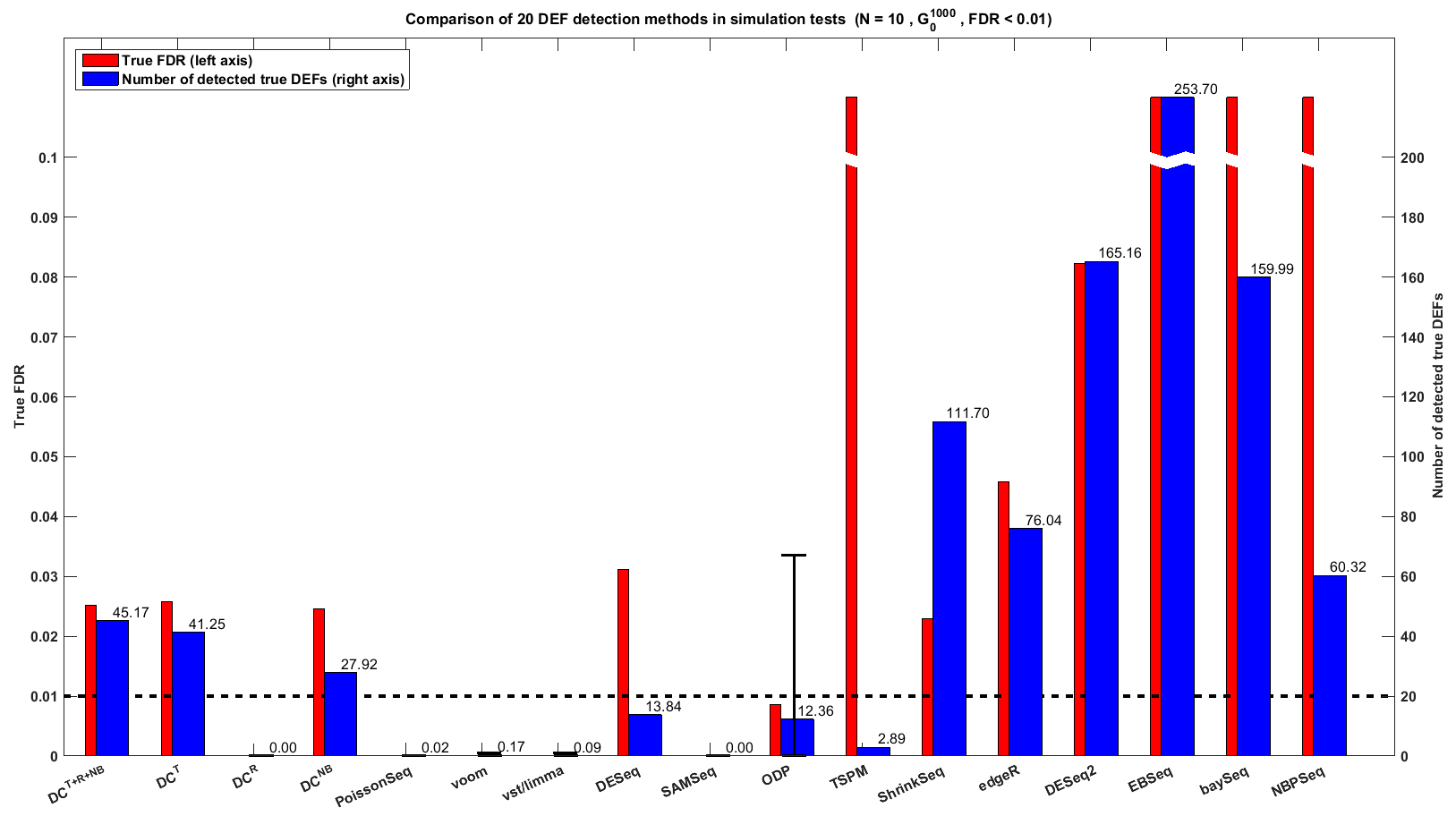

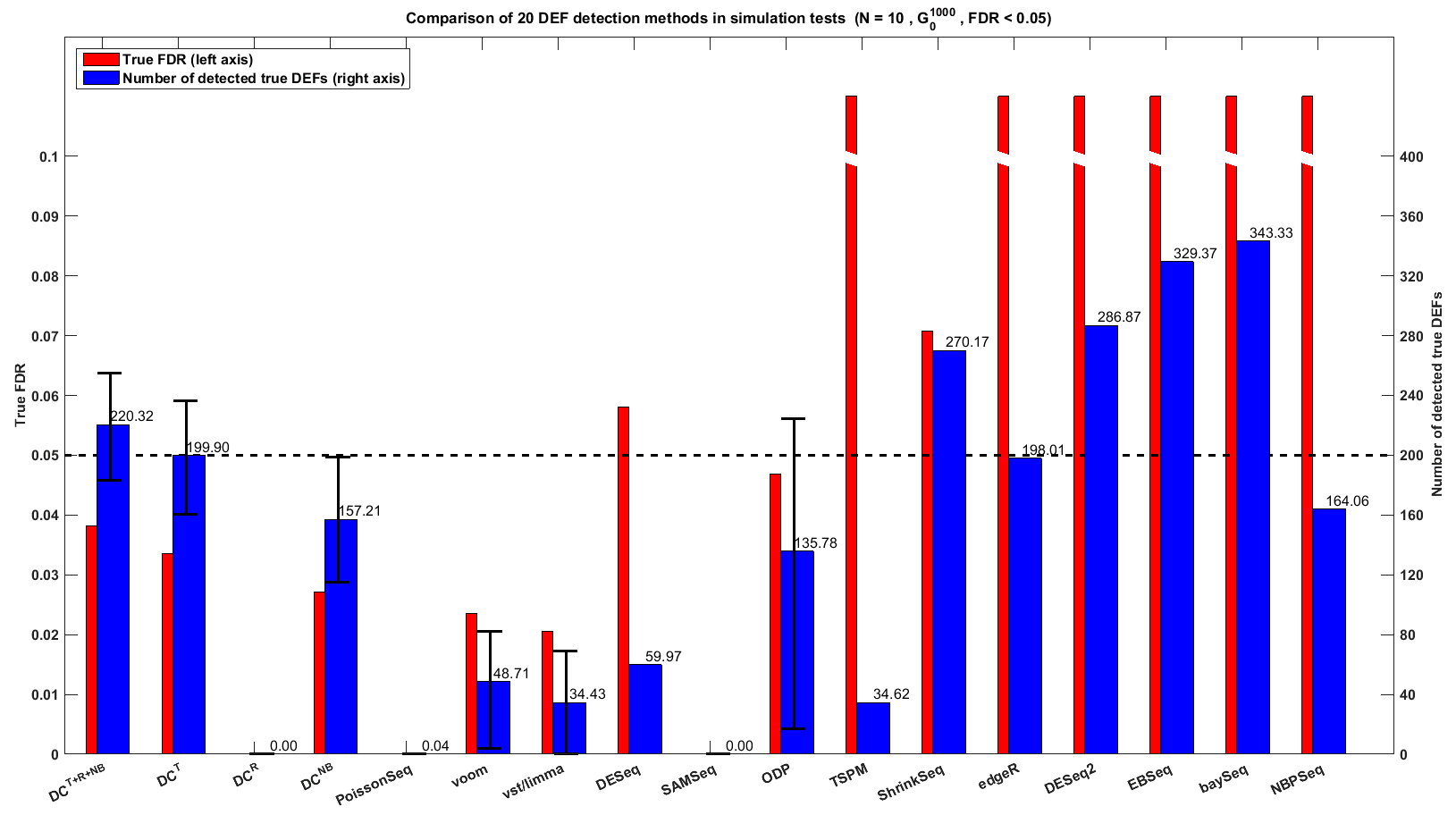


**(a) Comparison at target FDR < 0.01**

**(b) Comparison at target FDR < 0.05**

**True FDR**

**Number of detected true DEFs**

**Number of detected true DEFs**

**True FDR**

**Figure S17. Evaluates RNA-seq differential expression analysis methods using simulated data (5 *vs.* 5; $\boldsymbol{G}_{\boldsymbol{0}}^{\boldsymbol{1000}}$)**. Methods are listed along the ***x***-axis. The red bars indicate the average true FDRs (refer to the left ***y***-axis). The horizontal dashed line across the figure marks the target FDR. The blue bars indicate the average number of the detected true DEFs (refer to the right ***y***-axis). The 90% confidence intervals of the detected DEFs are marked except for those whose true FDRs exceed the target FDR by 10%. **(a)** target FDR < 0.01. **(b)** target FDR < 0.05.

**Simulation Test Results of *N* = 12,** $\boldsymbol{G}_{\boldsymbol{0}}^{\boldsymbol{1000}}$


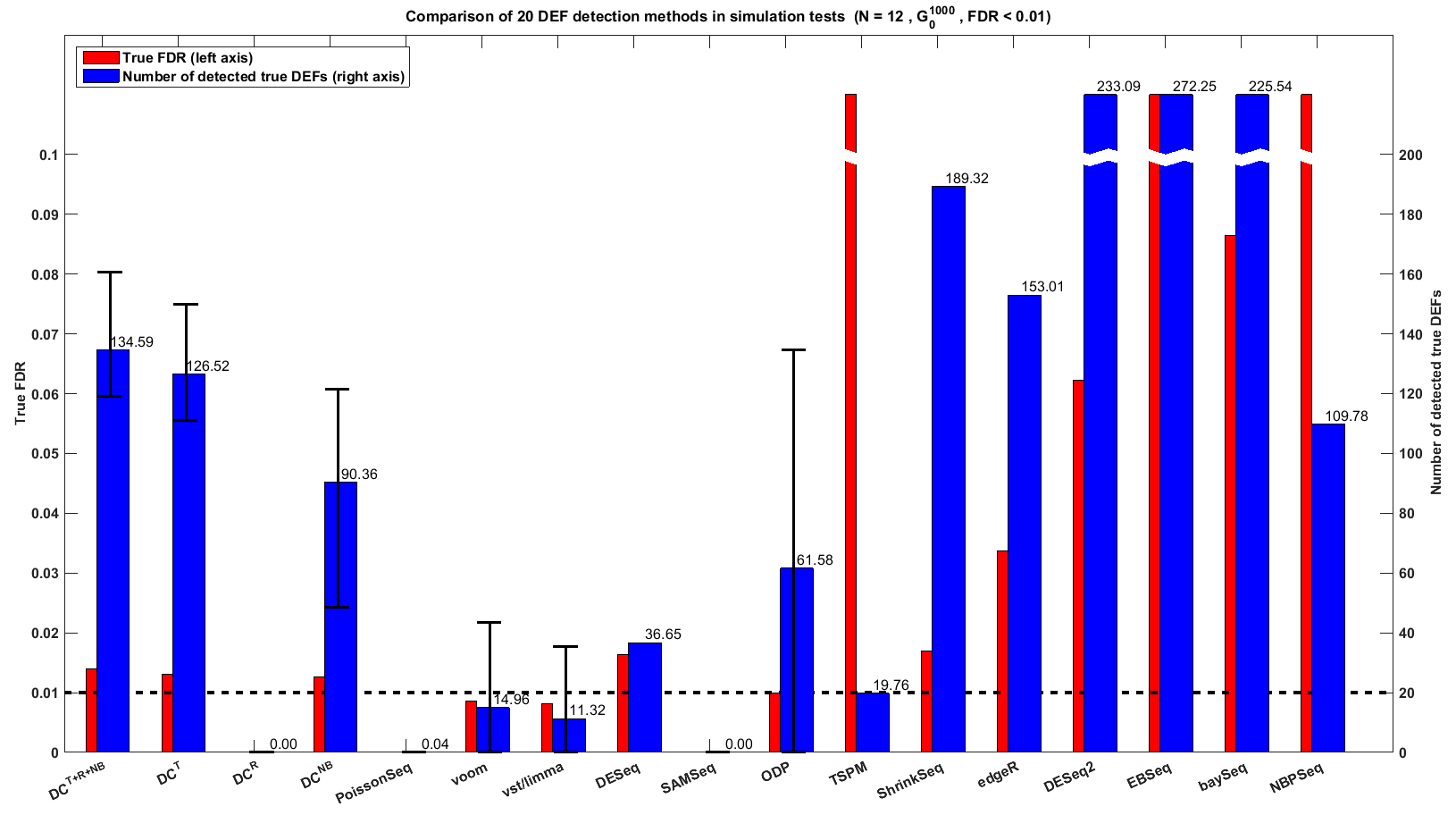

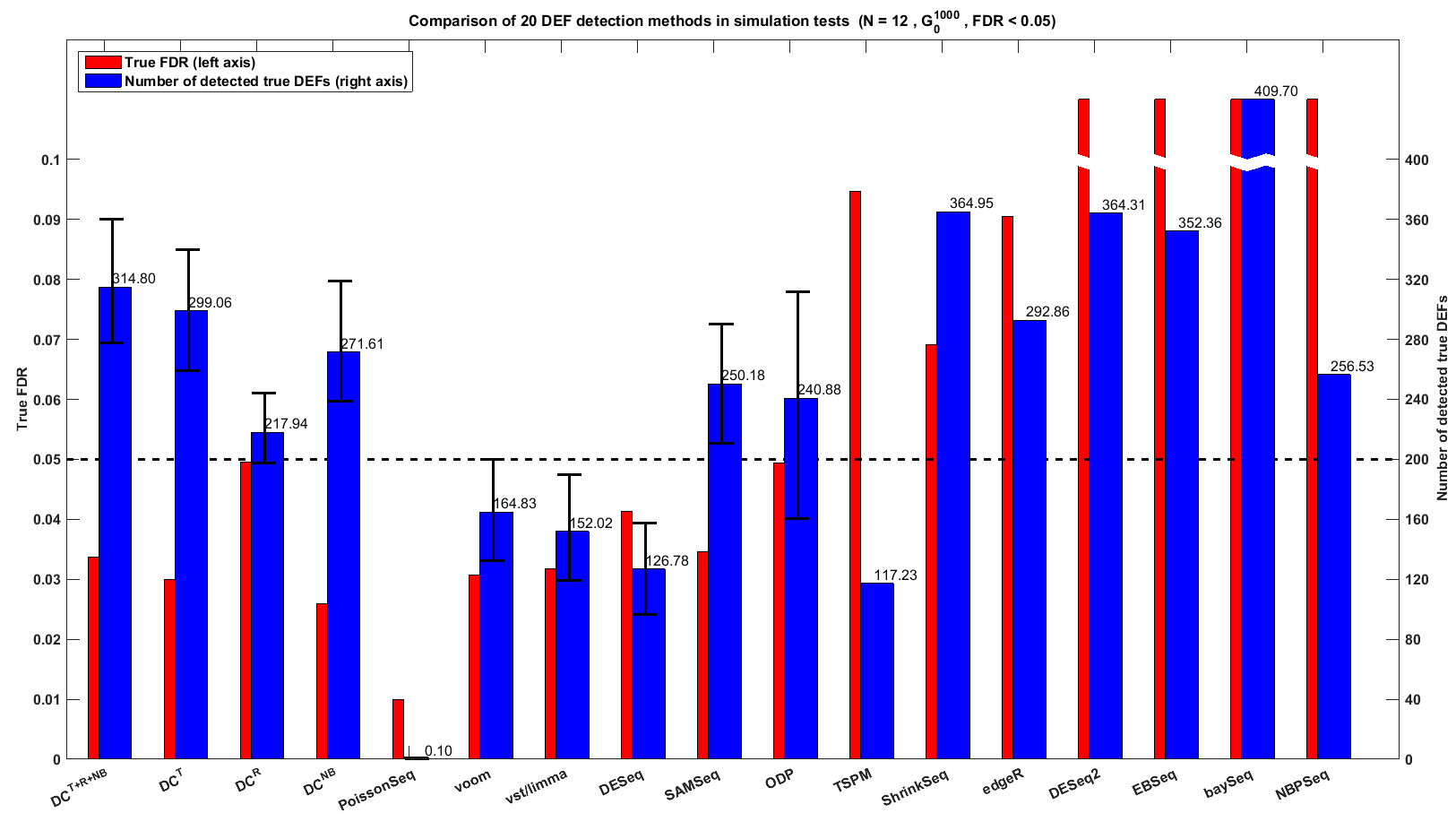


**(a) Comparison at target FDR < 0.01**

**(b) Comparison at target FDR < 0.05**

**True FDR**

**Number of detected true DEFs**

**Number of detected true DEFs**

**True FDR**

**Figure S18. Evaluates RNA-seq differential expression analysis methods using simulated data (6 *vs.* 6; $\boldsymbol{G}_{\boldsymbol{0}}^{\boldsymbol{1000}}$)**. Methods are listed along the ***x***-axis. The red bars indicate the average true FDRs (refer to the left ***y***-axis). The horizontal dashed line across the figure marks the target FDR. The blue bars indicate the average number of the detected true DEFs (refer to the right ***y***-axis). The 90% confidence intervals of the detected DEFs are marked except for those whose true FDRs exceed the target FDR by 10%. **(a)** target FDR < 0.01. **(b)** target FDR < 0.05.

**Simulation Test Results of *N* = 16,** $\boldsymbol{G}_{\boldsymbol{0}}^{\boldsymbol{1000}}$


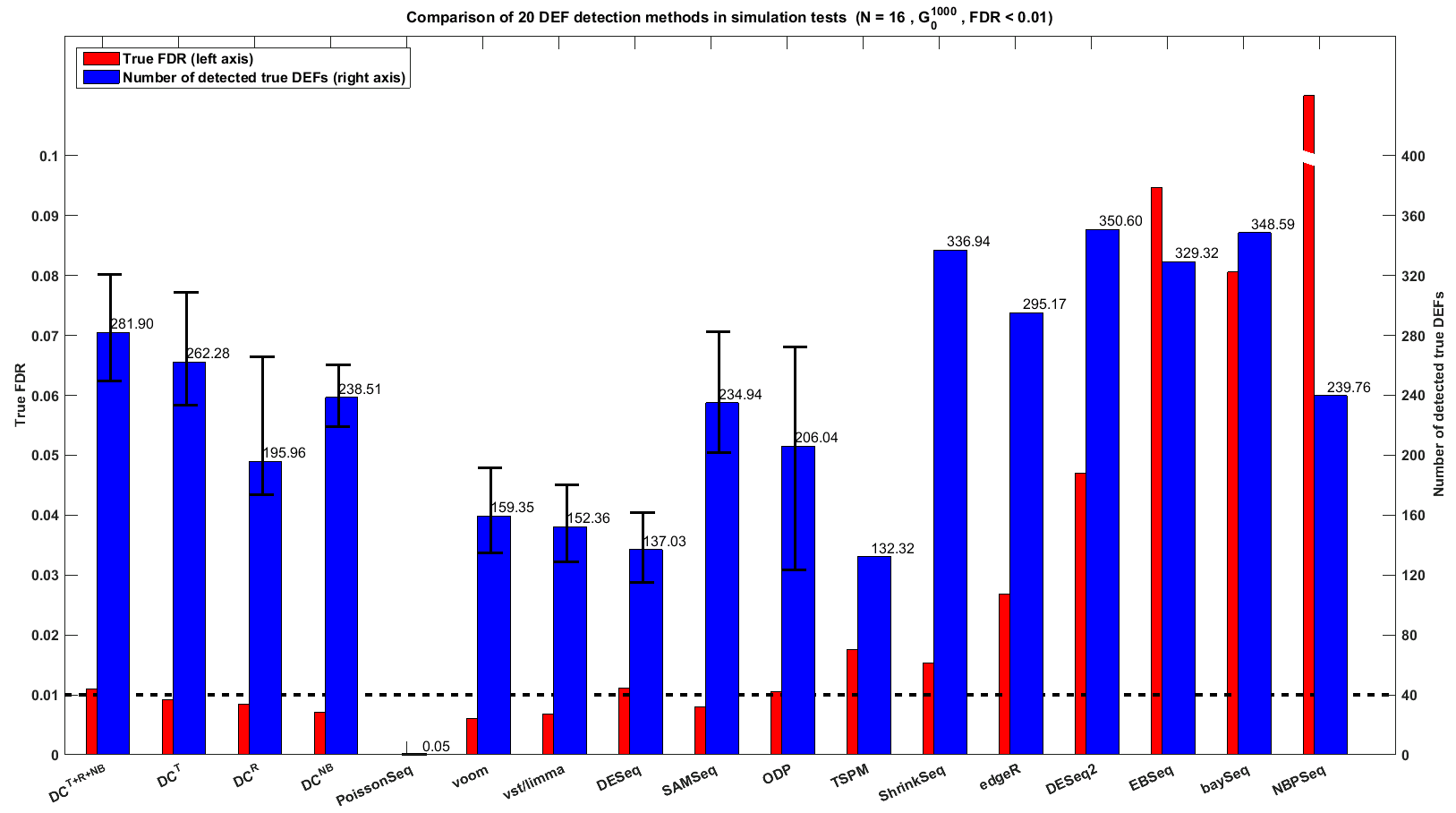

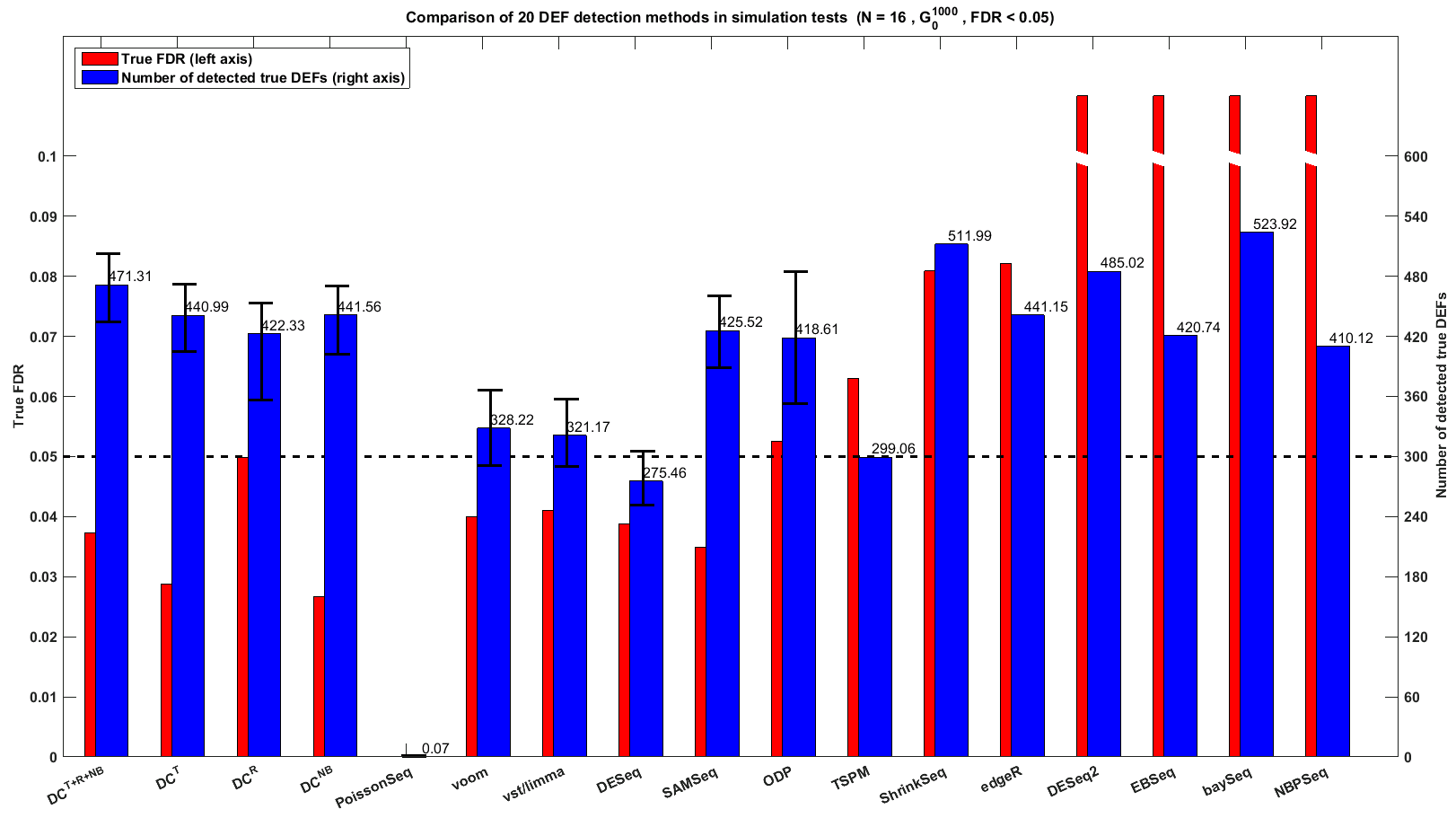


**(a) Comparison at target FDR < 0.01**

**(b) Comparison at target FDR < 0.05**

**True FDR**

**Number of detected true DEFs**

**Number of detected true DEFs**

**True FDR**

**Figure S19. Evaluates RNA-seq differential expression analysis methods using simulated data (8 *vs.* 8; $\boldsymbol{G}_{\boldsymbol{0}}^{\boldsymbol{1000}}$)**. Methods are listed along the ***x***-axis. The red bars indicate the average true FDRs (refer to the left ***y***-axis). The horizontal dashed line across the figure marks the target FDR. The blue bars indicate the average number of the detected true DEFs (refer to the right ***y***-axis). The 90% confidence intervals of the detected DEFs are marked except for those whose true FDRs exceed the target FDR by 10%. **(a)** target FDR < 0.01. **(b)** target FDR < 0.05.

**Simulation Test Results of *N* = 20,** $\boldsymbol{G}_{\mathbf{0}}^{\mathbf{1000}}$


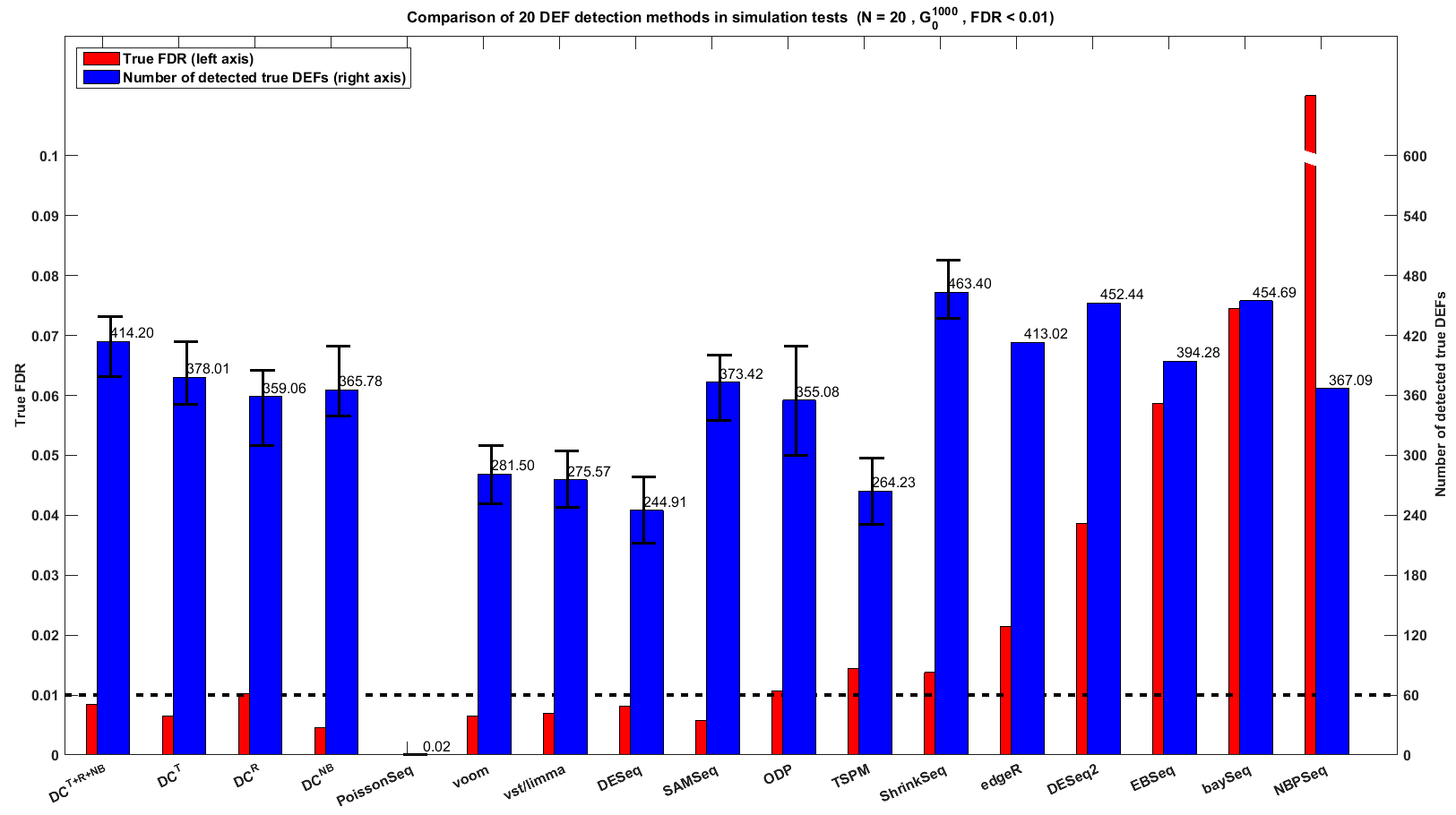

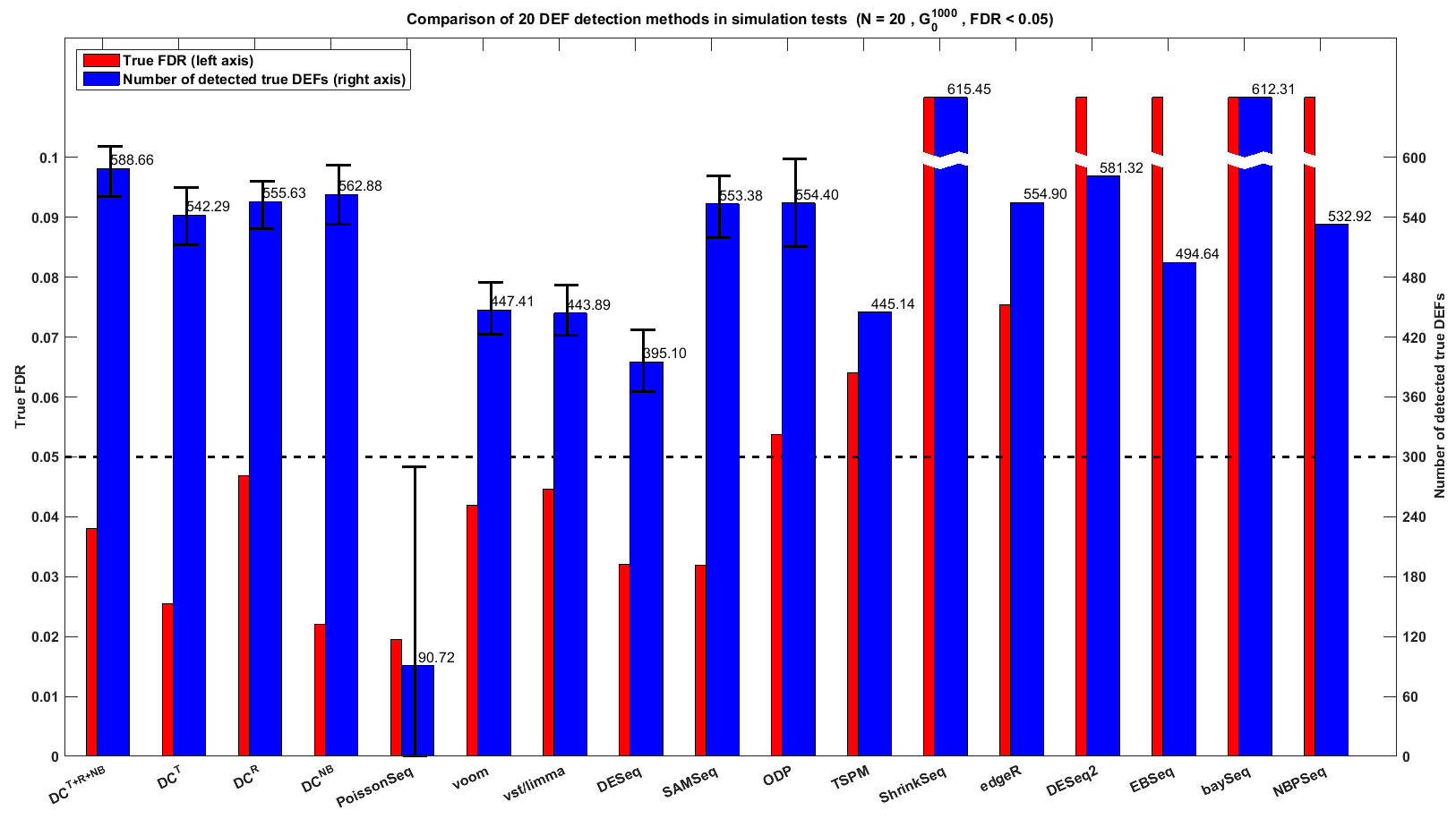


**(a) Comparison at target FDR < 0.01**

**(b) Comparison at target FDR < 0.05**

**True FDR**

**Number of detected true DEFs**

**Number of detected true DEFs**

**True FDR**

**Figure S20. Evaluates RNA-seq differential expression analysis methods using simulated data (10 *vs.* 10; $\boldsymbol{G}_{\boldsymbol{0}}^{\boldsymbol{1000}}$)**. Methods are listed along the ***x***-axis. The red bars indicate the average true FDRs (refer to the left ***y***-axis). The horizontal dashed line across the figure marks the target FDR. The blue bars indicate the average number of the detected true DEFs (refer to the right ***y***-axis). The 90% confidence intervals of the detected DEFs are marked except for those whose true FDRs exceed the target FDR by 10%. **(a)** target FDR < 0.01. **(b)** target FDR < 0.05.

## Curves of True Positives against Target FDR

**Simulation Test Results of *N* = 8,** $\boldsymbol{G}_{\mathbf{500}}^{\mathbf{500}}$


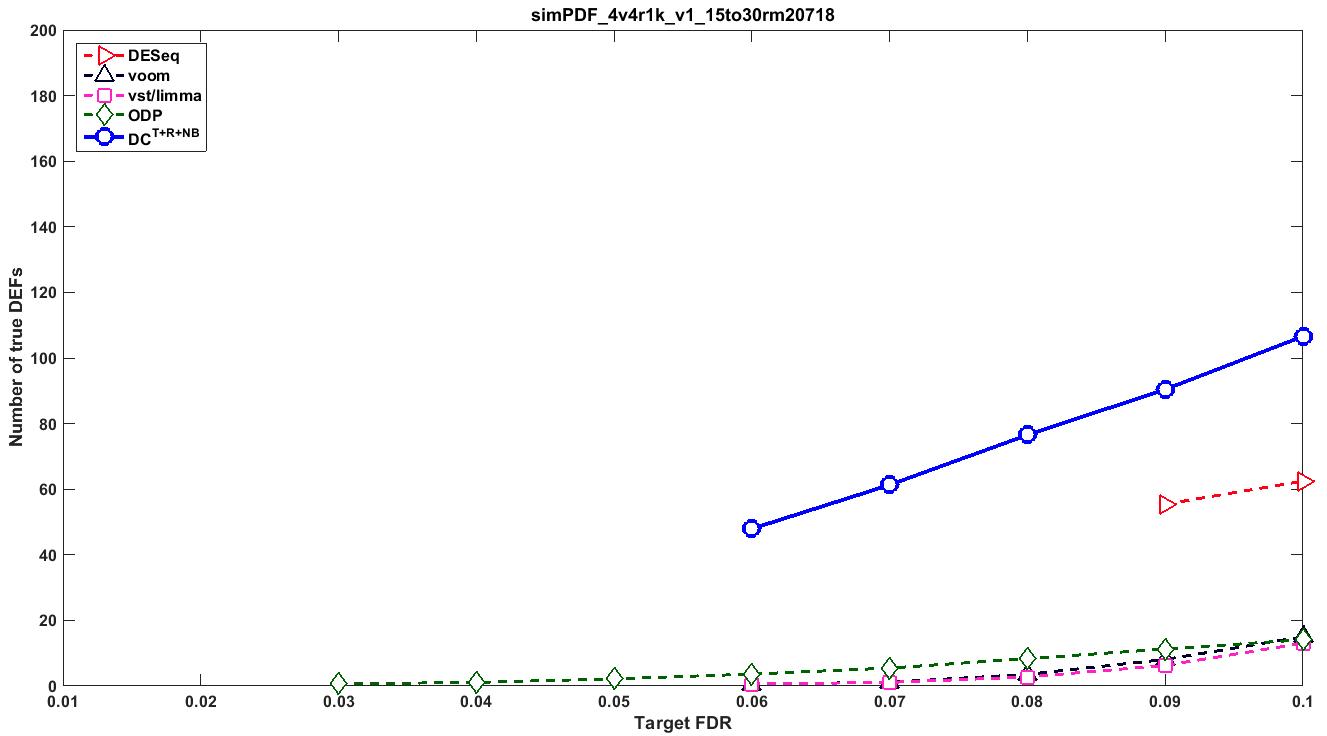


**Target FDR**

**Average number of detected true DEFs**

**Figure S21. Compare the curves of the true positives *vs.* the target FDR (4 *vs.* 4 and $\boldsymbol{G}_{\boldsymbol{500}}^{\boldsymbol{500}}$)**. The ***x***- and ***y***- axes indicate the target FDR cutoff and the average number of true positives, respectively. The solid curve with blue circle markers represents DC*^T+R+NB^* and other curves represent non-DC methods. The result of a method at a particular target FDR is shown in this plot if (1) its average true FDR does not exceed the target FDR by 10%; and (2) its average number of true DEFs is ≥ 0.5 (rounds up to 1).

**Simulation Test Results of *N* = 10,** $\boldsymbol{G}_{\mathbf{500}}^{\mathbf{500}}$


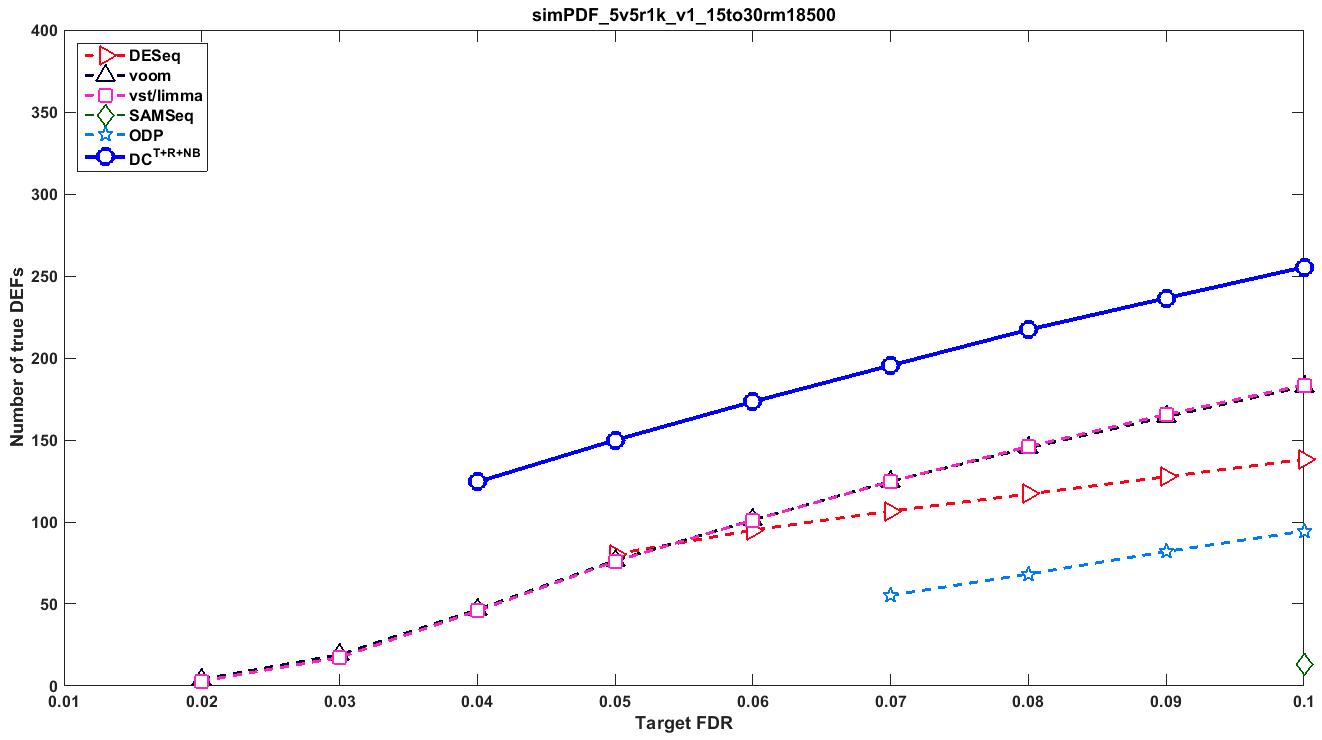


**Target FDR**

**Average number of detected true DEFs**

**Figure S22. Compare the curves of the true positives *vs.* the target FDR (5 *vs.* 5 and $\boldsymbol{G}_{\boldsymbol{500}}^{\boldsymbol{500}}$)**. The ***x***- and ***y***- axes indicate the target FDR cutoff and the average number of true positives, respectively. The solid curve with blue circle markers represents DC*^T+R+NB^* and other curves represent non-DC methods. The result of a method at a particular target FDR is shown in this plot if (1) its average true FDR does not exceed the target FDR by 10%; and (2) its average number of true DEFs is ≥ 0.5 (rounds up to 1).

**Simulation Test Results of *N* = 12,** $\boldsymbol{G}_{\mathbf{500}}^{\mathbf{500}}$


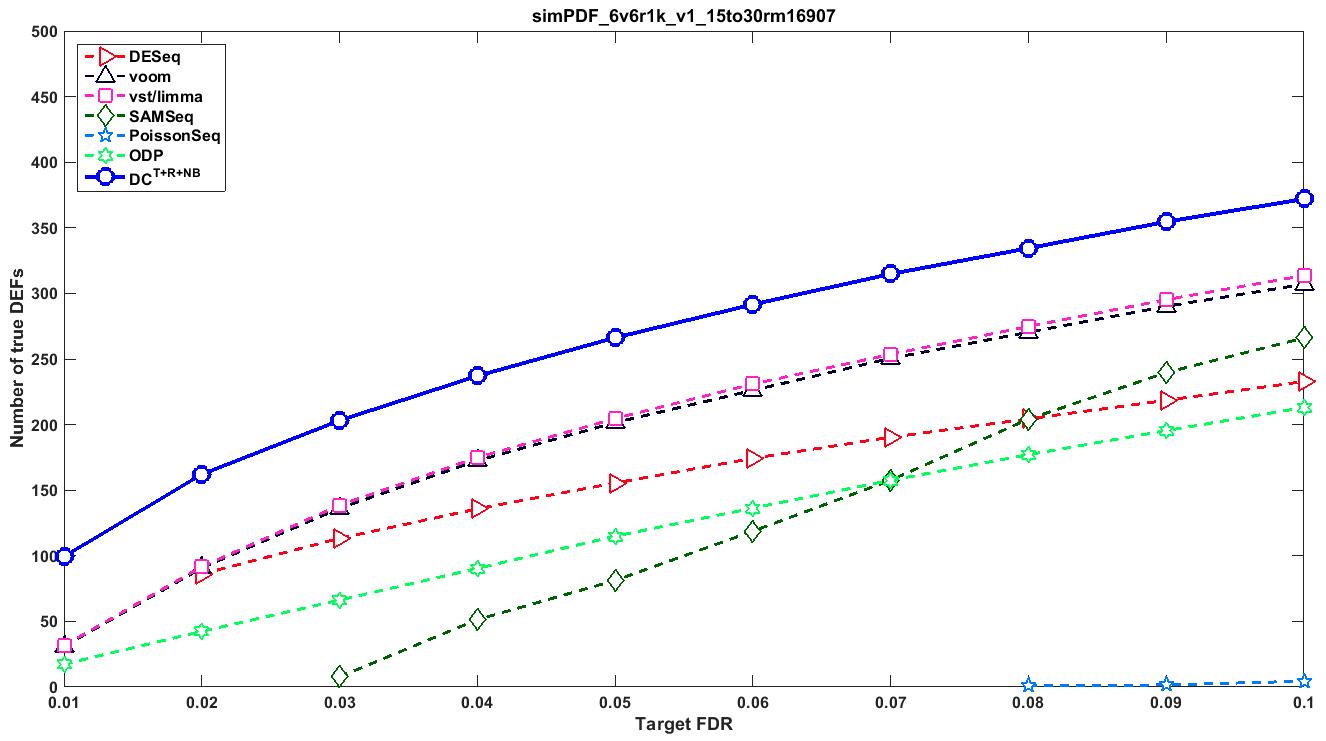


**Target FDR**

**Average number of detected true DEFs**

**Figure S23. Compare the curves of the true positives *vs.* the target FDR (6 *vs.* 6 and $\boldsymbol{G}_{\boldsymbol{500}}^{\boldsymbol{500}}$)**. The ***x***- and ***y***- axes indicate the target FDR cutoff and the average number of true positives, respectively. The solid curve with blue circle markers represents DC*^T+R+NB^* and other curves represent non-DC methods. The result of a method at a particular target FDR is shown in this plot if (1) its average true FDR does not exceed the target FDR by 10%; and (2) its average number of true DEFs is ≥ 0.5 (rounds up to 1).

**Simulation Test Results of *N* = 16,** $\boldsymbol{G}_{\mathbf{500}}^{\mathbf{500}}$


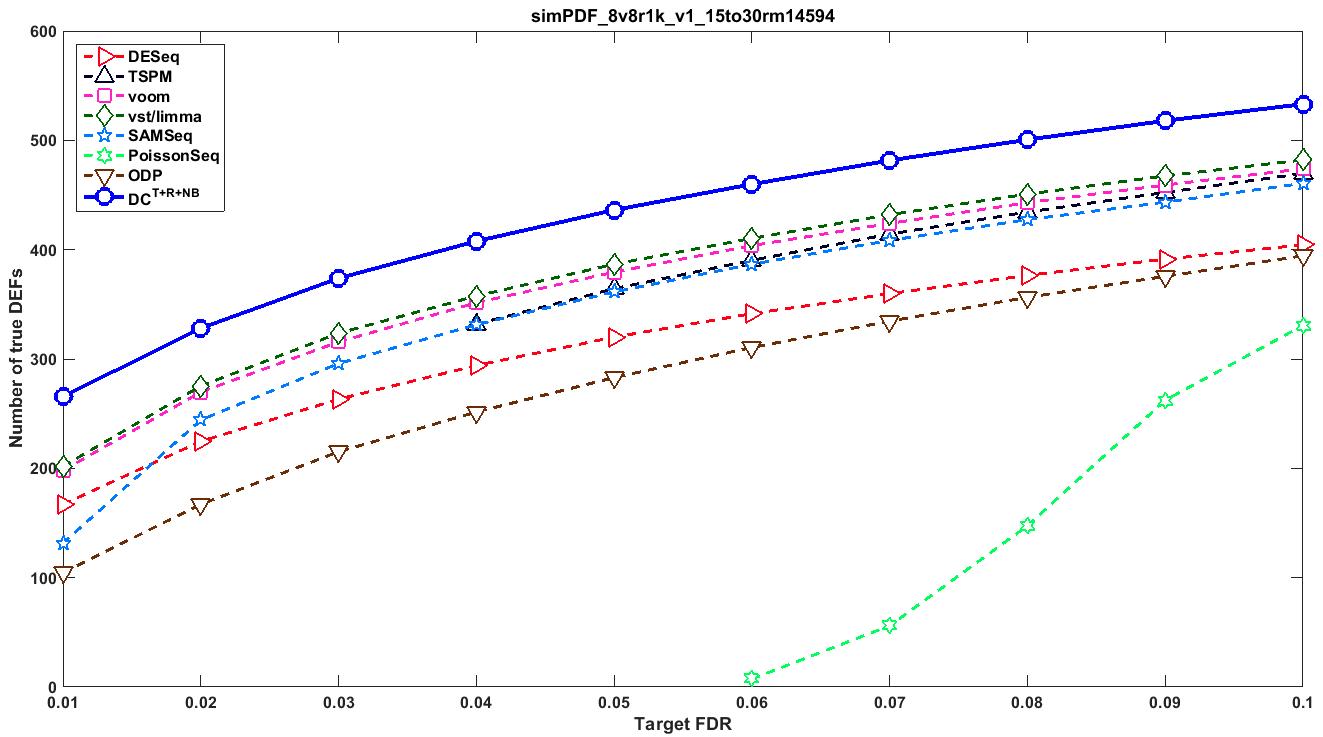


**Target FDR**

**Average number of detected true DEFs**

**Figure S24. Compare the curves of the true positives *vs.* the target FDR (8 *vs.* 8 and $\boldsymbol{G}_{\boldsymbol{500}}^{\boldsymbol{500}}$)**. The ***x***- and ***y***- axes indicate the target FDR cutoff and the average number of true positives, respectively. The solid curve with blue circle markers represents DC*^T+R+NB^* and other curves represent non-DC methods. The result of a method at a particular target FDR is shown in this plot if (1) its average true FDR does not exceed the target FDR by 10%; and (2) its average number of true DEFs is ≥ 0.5 (rounds up to 1).

**Simulation Test Results of *N* = 20,** $\boldsymbol{G}_{\mathbf{500}}^{\mathbf{500}}$


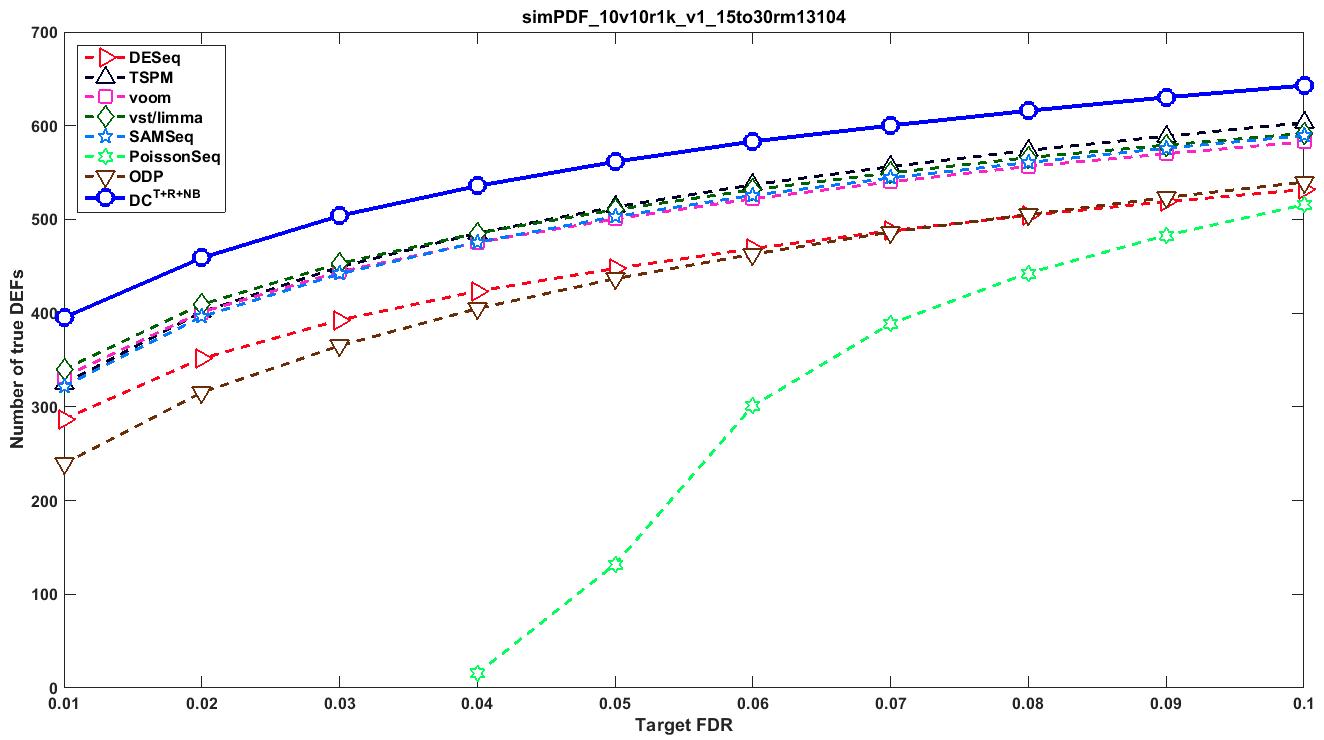


**Target FDR**

**Average number of detected true DEFs**

**Figure S25. Compare the curves of the true positives *vs.* the target FDR (10 *vs.* 10 and $\boldsymbol{G}_{\boldsymbol{500}}^{\boldsymbol{500}}$)**. The ***x***- and ***y***- axes indicate the target FDR cutoff and the average number of true positives, respectively. The solid curve with blue circle markers represents DC*^T+R+NB^* and other curves represent non-DC methods. The result of a method at a particular target FDR is shown in this plot if (1) its average true FDR does not exceed the target FDR by 10%; and (2) its average number of true DEFs is ≥ 0.5 (rounds up to 1).

**Simulation Test Results of *N* = 8,** $\boldsymbol{G}_{\mathbf{600}}^{\mathbf{600}}$


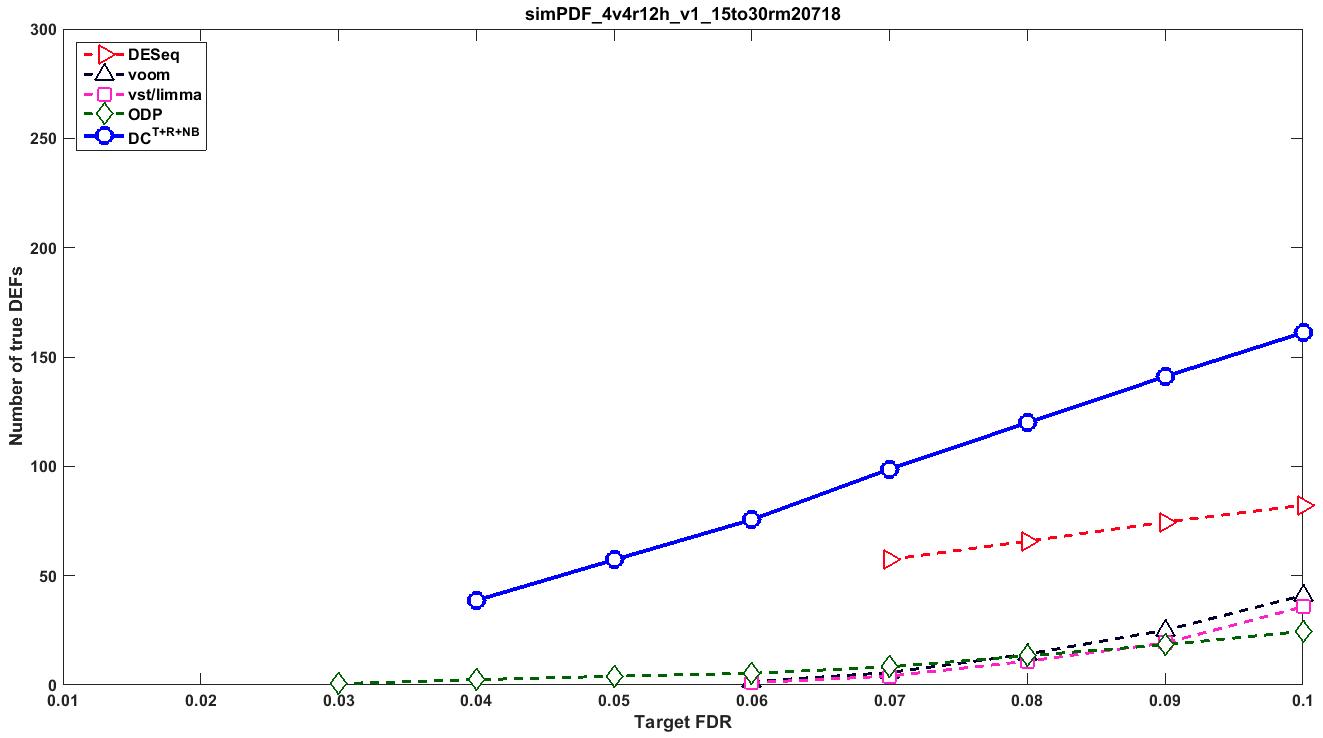


**Target FDR**

**Average number of detected true DEFs**

**Figure S26. Compare the curves of the true positives *vs.* the target FDR (4 *vs.* 4 and $\boldsymbol{G}_{\boldsymbol{600}}^{\boldsymbol{600}}$)**. The ***x***- and ***y***- axes indicate the target FDR cutoff and the average number of true positives, respectively. The solid curve with blue circle markers represents DC*^T+R+NB^* and other curves represent non-DC methods. The result of a method at a particular target FDR is shown in this plot if (1) its average true FDR does not exceed the target FDR by 10%; and (2) its average number of true DEFs is ≥ 0.5 (rounds up to 1).

**Simulation Test Results of *N* = 10,** $\boldsymbol{G}_{\mathbf{600}}^{\mathbf{600}}$


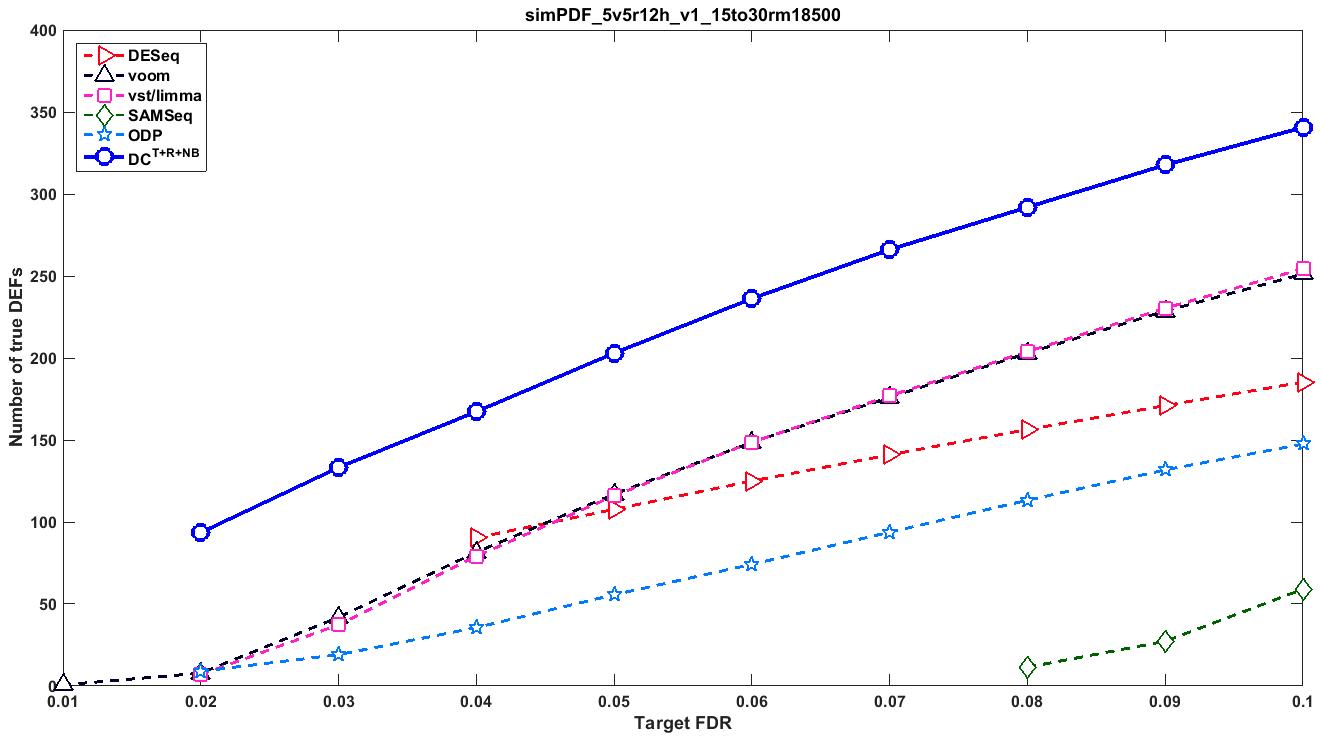


**Target FDR**

**Average number of detected true DEFs**

**Figure S27. Compare the curves of the true positives *vs.* the target FDR (5 *vs.* 5 and $\boldsymbol{G}_{\boldsymbol{600}}^{\boldsymbol{600}}$)**. The ***x***- and ***y***- axes indicate the target FDR cutoff and the average number of true positives, respectively. The solid curve with blue circle markers represents DC*^T+R+NB^* and other curves represent non-DC methods. The result of a method at a particular target FDR is shown in this plot if (1) its average true FDR does not exceed the target FDR by 10%; and (2) its average number of true DEFs is ≥ 0.5 (rounds up to 1).

**Simulation Test Results of *N* = 12,** $\boldsymbol{G}_{\mathbf{600}}^{\mathbf{600}}$


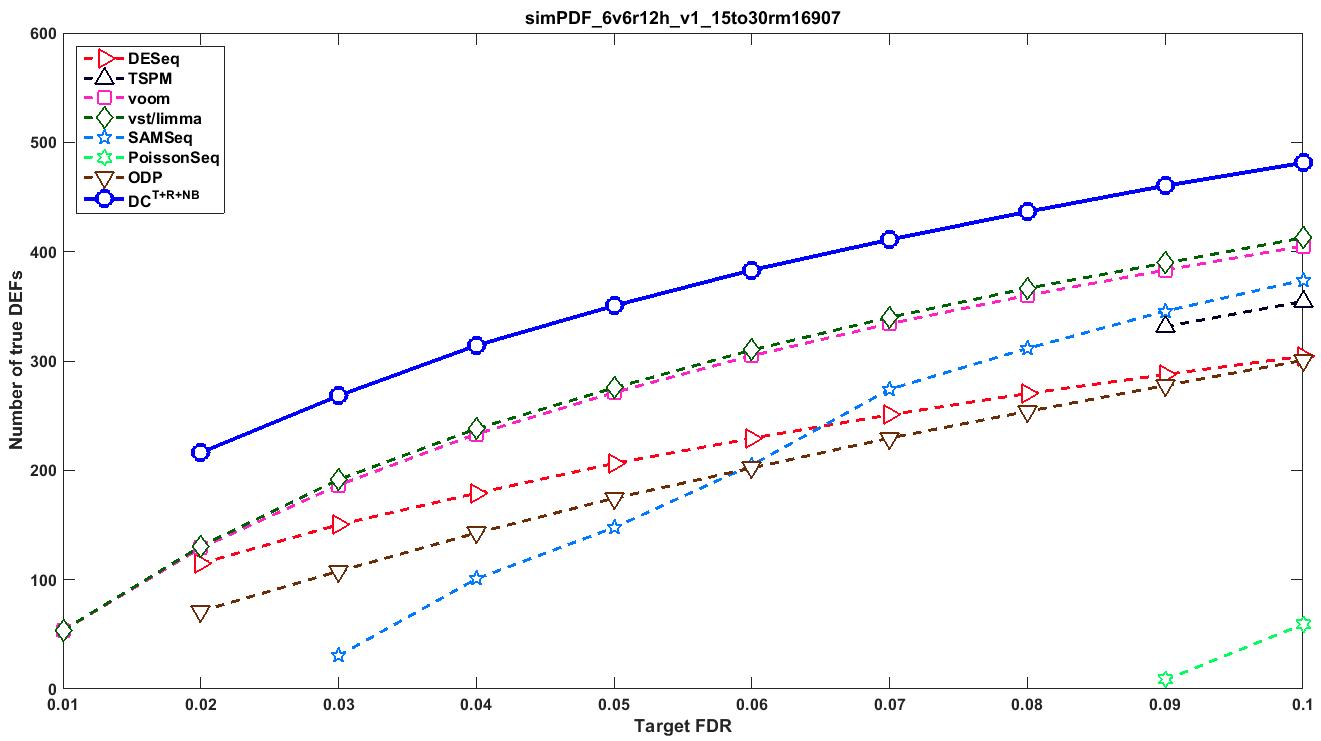


**Target FDR**

**Average number of detected true DEFs**

**Figure S28. Compare the curves of the true positives *vs.* the target FDR (6 *vs.* 6 and $\boldsymbol{G}_{\boldsymbol{600}}^{\boldsymbol{600}}$)**. The ***x***- and ***y***- axes indicate the target FDR cutoff and the average number of true positives, respectively. The solid curve with blue circle markers represents DC*^T+R+NB^* and other curves represent non-DC methods. The result of a method at a particular target FDR is shown in this plot if (1) its average true FDR does not exceed the target FDR by 10%; and (2) its average number of true DEFs is ≥ 0.5 (rounds up to 1).

**Simulation Test Results of *N* = 16,** $\boldsymbol{G}_{\mathbf{600}}^{\mathbf{600}}$


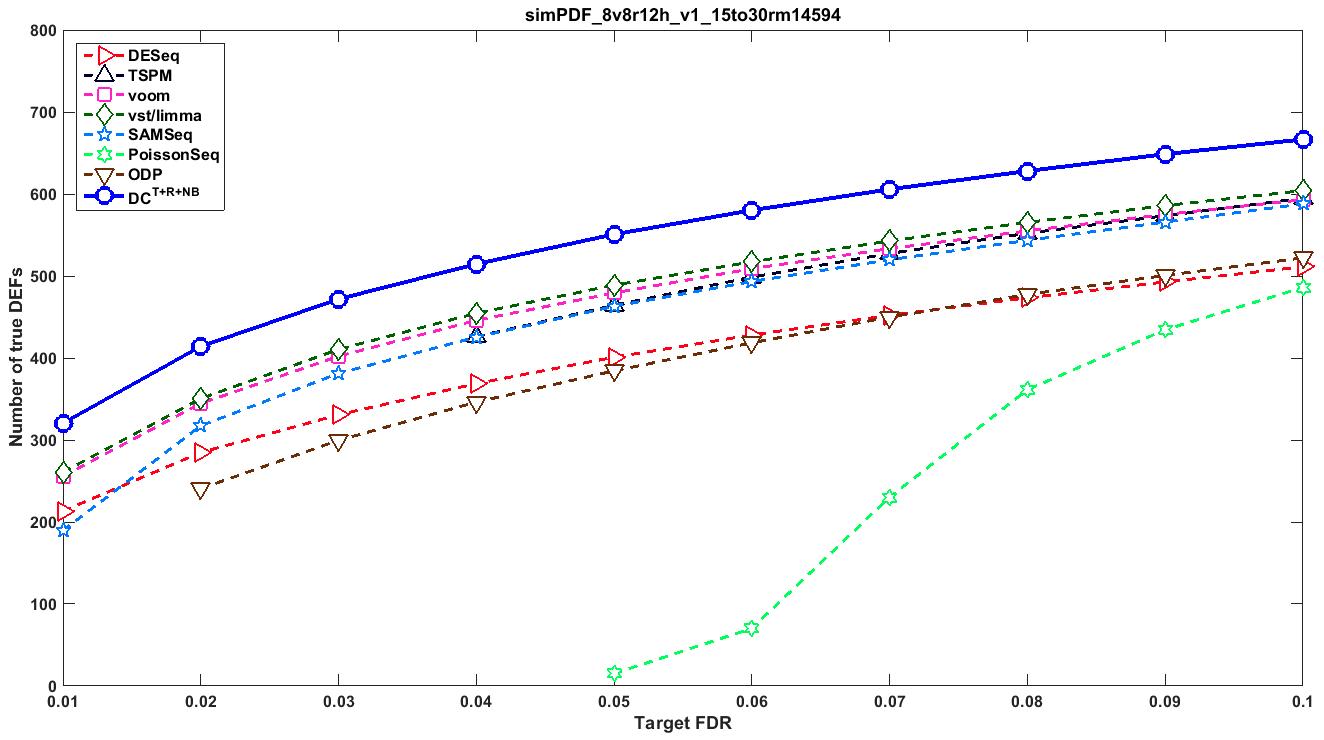


**Target FDR**

**Average number of detected true DEFs**

**Figure S29. Compare the curves of the true positives *vs.* the target FDR (8 *vs.* 8 and $\boldsymbol{G}_{\boldsymbol{600}}^{\boldsymbol{600}}$)**. The ***x***- and ***y***- axes indicate the target FDR cutoff and the average number of true positives, respectively. The solid curve with blue circle markers represents DC*^T+R+NB^* and other curves represent non-DC methods. The result of a method at a particular target FDR is shown in this plot if (1) its average true FDR does not exceed the target FDR by 10%; and (2) its average number of true DEFs is ≥ 0.5 (rounds up to 1).

**Simulation Test Results of *N* = 20,** $\boldsymbol{G}_{\mathbf{600}}^{\mathbf{600}}$


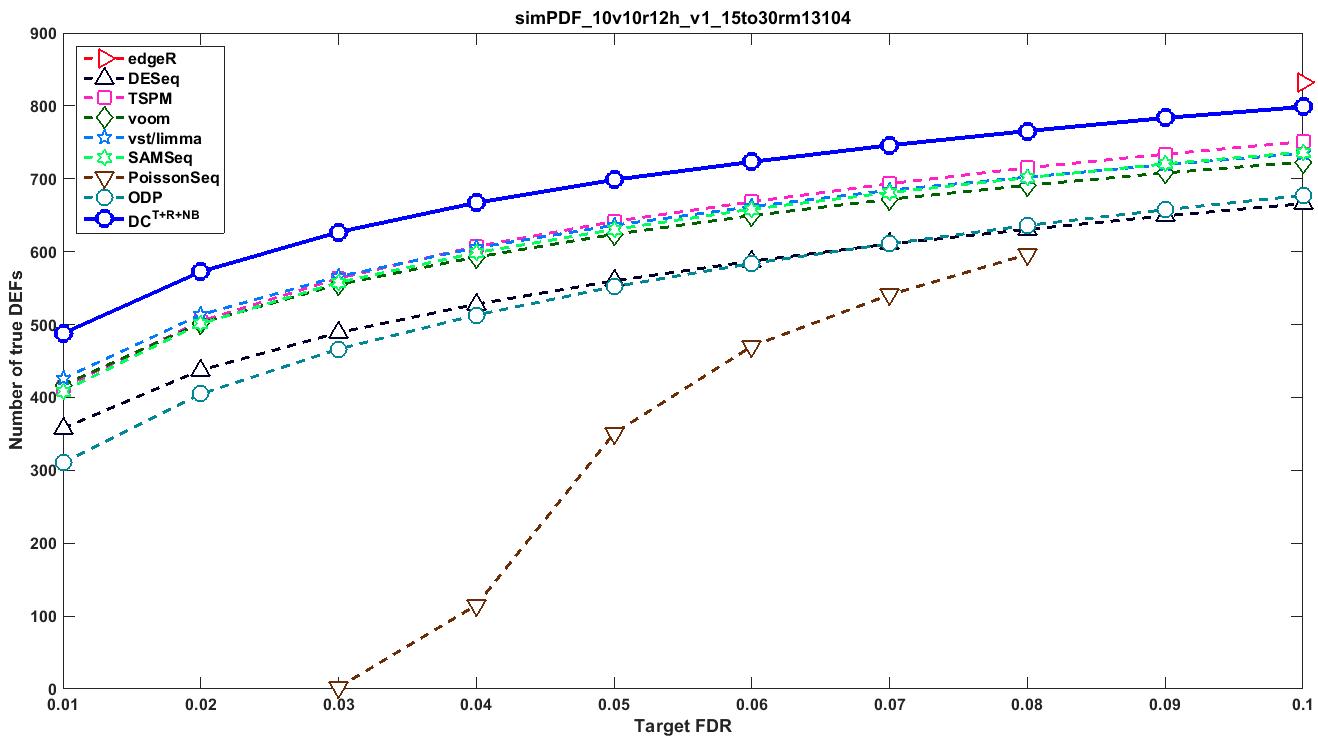


**Target FDR**

**Average number of detected true DEFs**

**Figure S30. Compare the curves of the true positives *vs.* the target FDR (10 *vs.* 10 and $\boldsymbol{G}_{\boldsymbol{600}}^{\boldsymbol{600}}$)**. The ***x***- and ***y***- axes indicate the target FDR cutoff and the average number of true positives, respectively. The solid curve with blue circle markers represents DC*^T+R+NB^* and other curves represent non-DC methods. The result of a method at a particular target FDR is shown in this plot if (1) its average true FDR does not exceed the target FDR by 10%; and (2) its average number of true DEFs is ≥ 0.5 (rounds up to 1).

**Simulation Test Results of *N* = 8,** $\boldsymbol{G}_{\mathbf{400}}^{\mathbf{400}}$


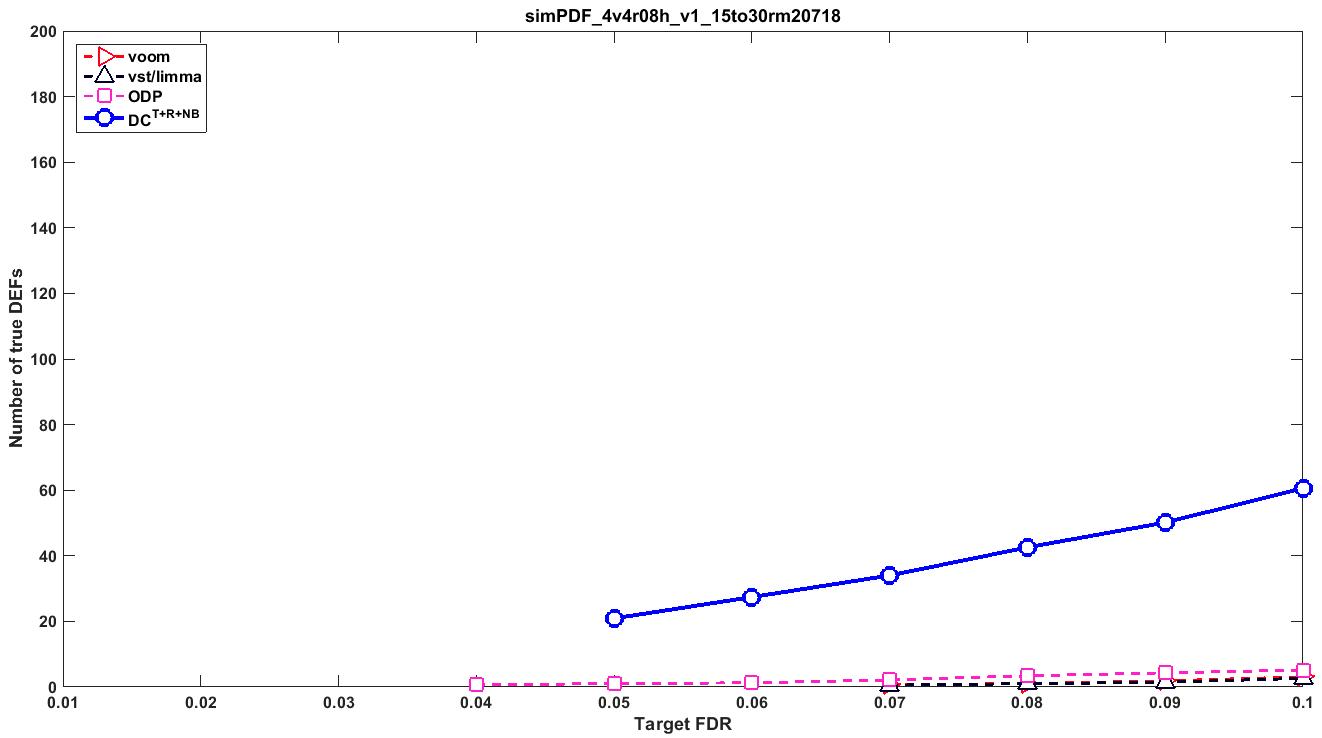


**Target FDR**

**Average number of detected true DEFs**

**Figure S31. Compare the curves of the true positives *vs.* the target FDR (4 *vs.* 4 and $\boldsymbol{G}_{\boldsymbol{400}}^{\boldsymbol{400}}$)**. The ***x***- and ***y***- axes indicate the target FDR cutoff and the average number of true positives, respectively. The solid curve with blue circle markers represents DC*^T+R+NB^* and other curves represent non-DC methods. The result of a method at a particular target FDR is shown in this plot if (1) its average true FDR does not exceed the target FDR by 10%; and (2) its average number of true DEFs is ≥ 0.5 (rounds up to 1).

**Simulation Test Results of *N* = 10,** $\boldsymbol{G}_{\mathbf{400}}^{\mathbf{400}}$


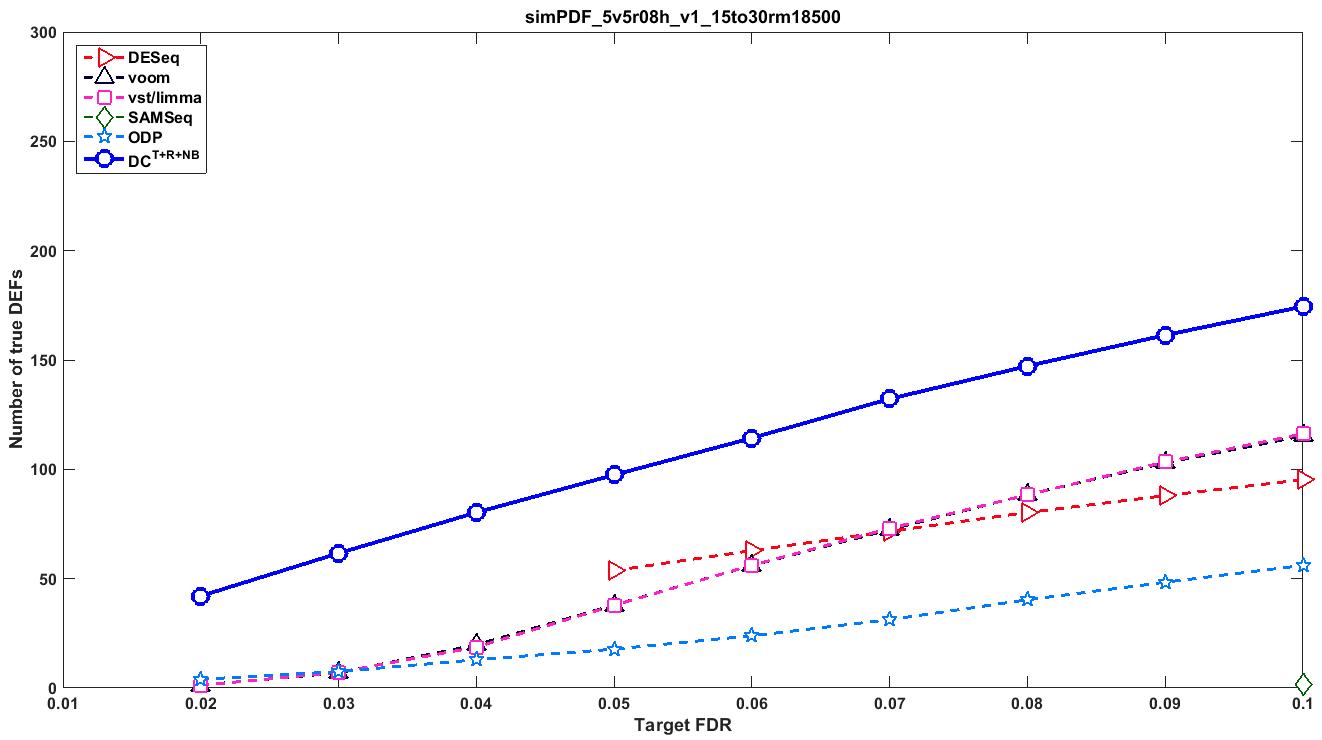


**Target FDR**

**Average number of detected true DEFs**

**Figure S32. Compare the curves of the true positives *vs.* the target FDR (5 *vs.* 5 and $\boldsymbol{G}_{\boldsymbol{400}}^{\boldsymbol{400}}$)**. The ***x***- and ***y***- axes indicate the target FDR cutoff and the average number of true positives, respectively. The solid curve with blue circle markers represents DC*^T+R+NB^* and other curves represent non-DC methods. The result of a method at a particular target FDR is shown in this plot if (1) its average true FDR does not exceed the target FDR by 10%; and (2) its average number of true DEFs is ≥ 0.5 (rounds up to 1).

**Simulation Test Results of *N* = 12,** $\boldsymbol{G}_{\mathbf{400}}^{\mathbf{400}}$


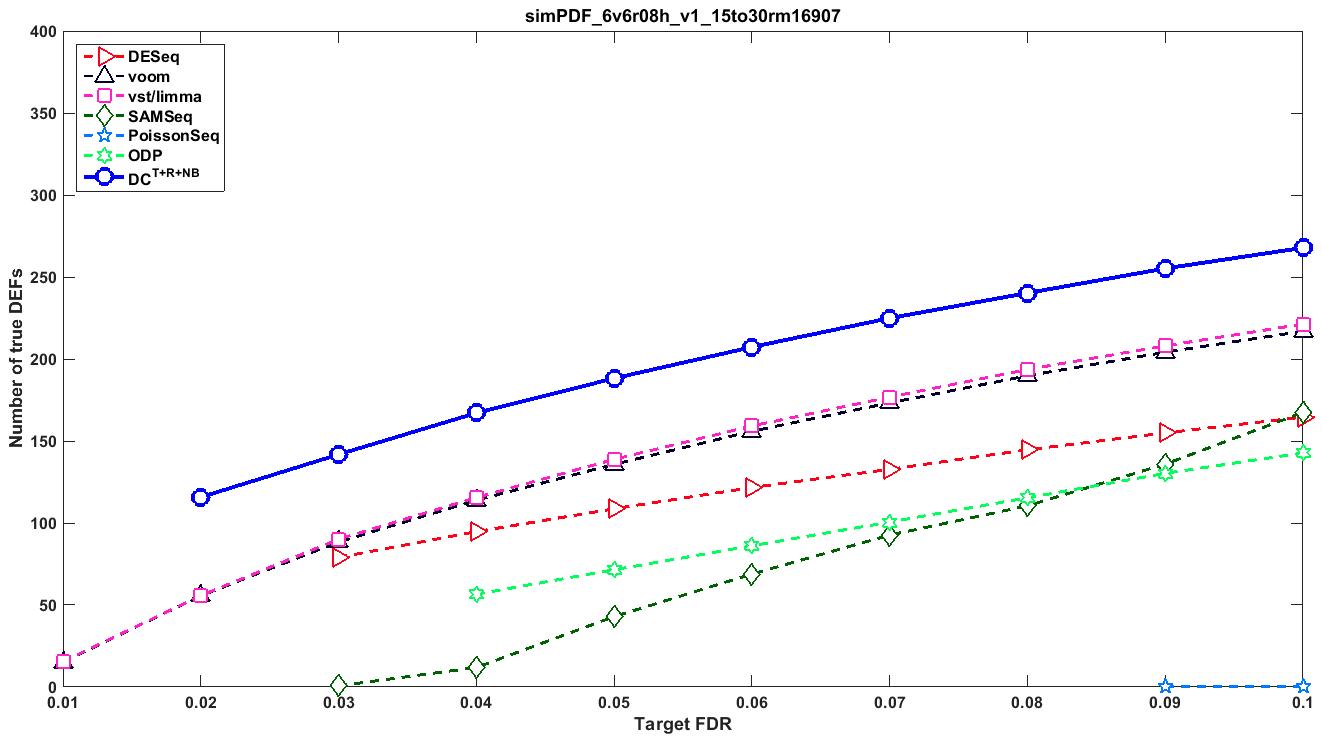


**Target FDR**

**Average number of detected true DEFs**

**Figure S33. Compare the curves of the true positives *vs.* the target FDR (6 *vs.* 6 and $\boldsymbol{G}_{\boldsymbol{400}}^{\boldsymbol{400}}$)**. The ***x***- and ***y***- axes indicate the target FDR cutoff and the average number of true positives, respectively. The solid curve with blue circle markers represents DC*^T+R+NB^* and other curves represent non-DC methods. The result of a method at a particular target FDR is shown in this plot if (1) its average true FDR does not exceed the target FDR by 10%; and (2) its average number of true DEFs is ≥ 0.5 (rounds up to 1).

**Simulation Test Results of *N* = 16,** $\boldsymbol{G}_{\mathbf{400}}^{\mathbf{400}}$


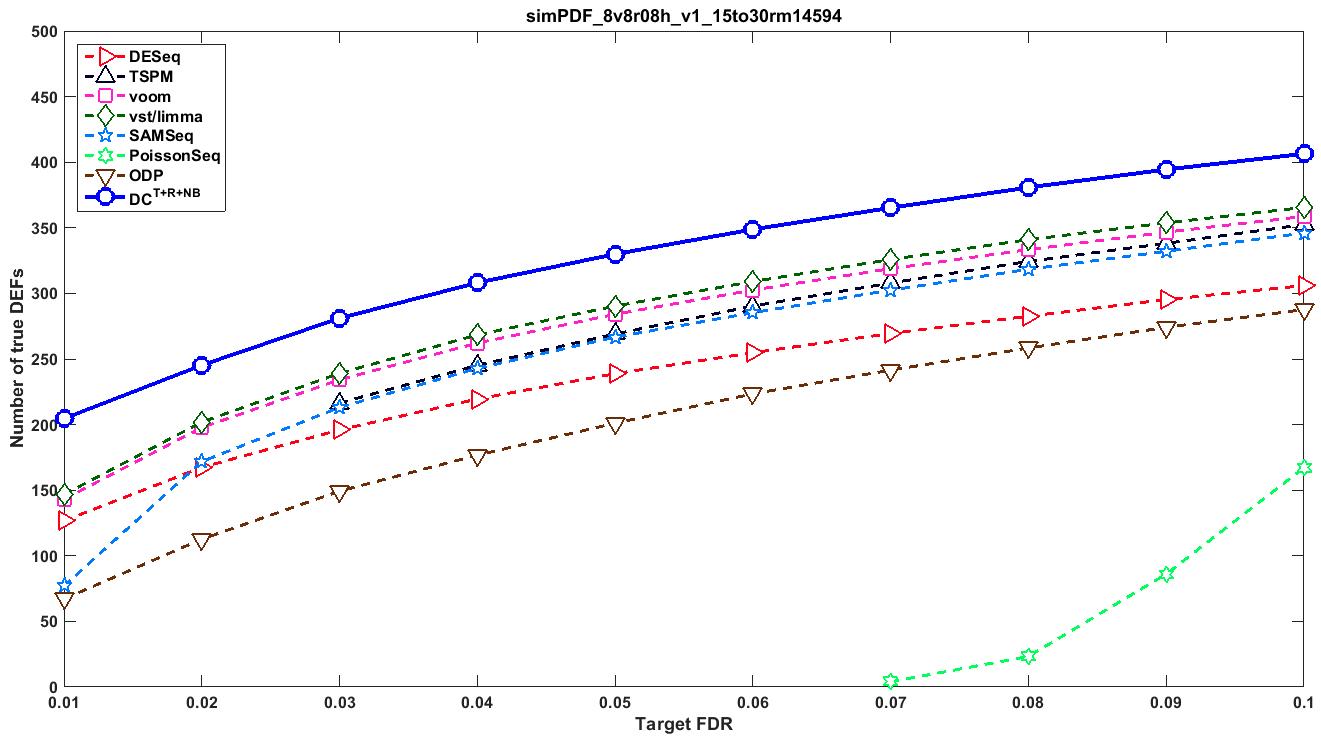


**Target FDR**

**Average number of detected true DEFs**

**Figure S34. Compare the curves of the true positives *vs.* the target FDR (8 *vs.* 8 and $\boldsymbol{G}_{\boldsymbol{400}}^{\boldsymbol{400}}$)**. The ***x***- and ***y***- axes indicate the target FDR cutoff and the average number of true positives, respectively. The solid curve with blue circle markers represents DC*^T+R+NB^* and other curves represent non-DC methods. The result of a method at a particular target FDR is shown in this plot if (1) its average true FDR does not exceed the target FDR by 10%; and (2) its average number of true DEFs is ≥ 0.5 (rounds up to 1).

**Simulation Test Results of *N* = 20,** $\boldsymbol{G}_{\mathbf{400}}^{\mathbf{400}}$


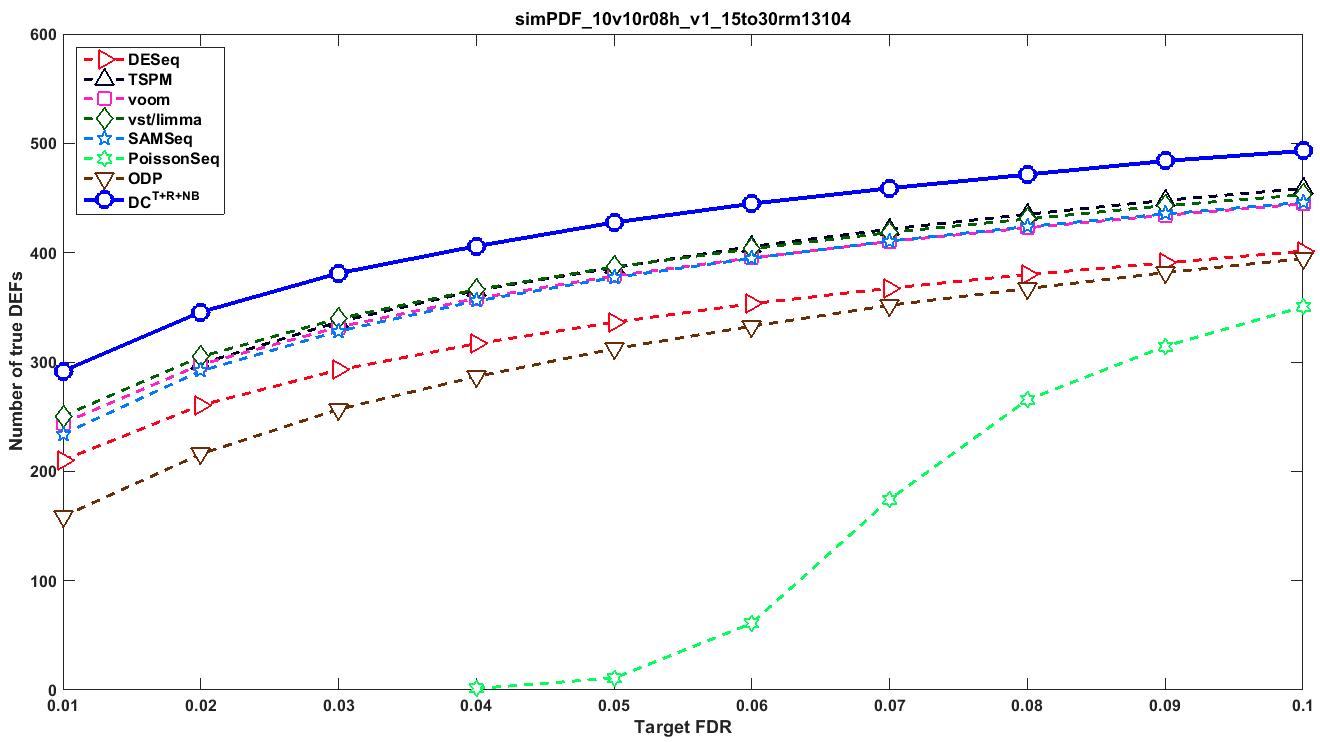


**Target FDR**

**Average number of detected true DEFs**

**Figure S35. Compare the curves of the true positives *vs.* the target FDR (10 *vs.* 10 and $\boldsymbol{G}_{\boldsymbol{400}}^{\boldsymbol{400}}$)**. The ***x***- and ***y***- axes indicate the target FDR cutoff and the average number of true positives, respectively. The solid curve with blue circle markers represents DC*^T+R+NB^* and other curves represent non-DC methods. The result of a method at a particular target FDR is shown in this plot if (1) its average true FDR does not exceed the target FDR by 10%; and (2) its average number of true DEFs is ≥ 0.5 (rounds up to 1).

**Simulation Test Results of *N* = 8,** $\boldsymbol{G}_{\mathbf{0}}^{\mathbf{1000}}$


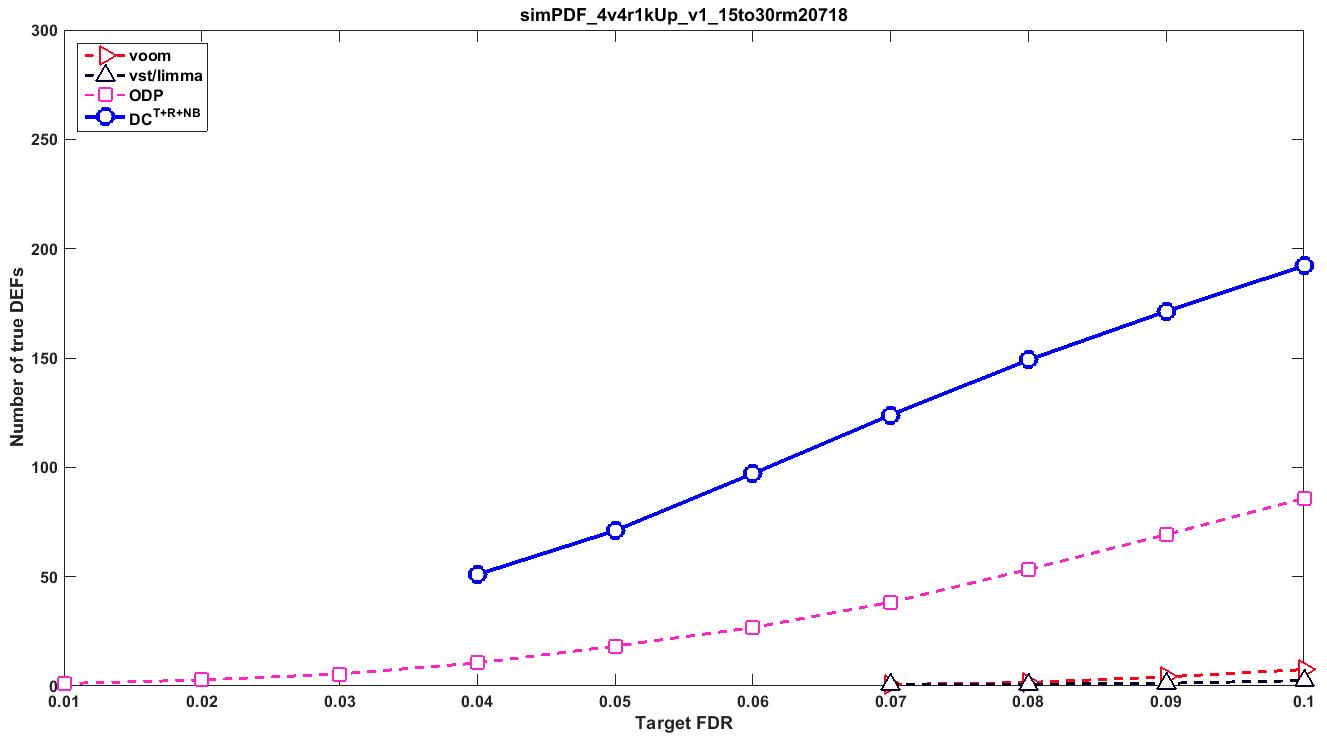


**Target FDR**

**Average number of detected true DEFs**

**Figure S36. Compare the curves of the true positives *vs.* the target FDR (4 *vs.* 4 and $\boldsymbol{G}_{\boldsymbol{0}}^{\boldsymbol{1000}}$)**. The ***x***- and ***y***- axes indicate the target FDR cutoff and the average number of true positives, respectively. The solid curve with blue circle markers represents DC*^T+R+NB^* and other curves represent non-DC methods. The result of a method at a particular target FDR is shown in this plot if (1) its average true FDR does not exceed the target FDR by 10%; and (2) its average number of true DEFs is ≥ 0.5 (rounds up to 1).

**Simulation Test Results of *N* = 10,** $\boldsymbol{G}_{\boldsymbol{0}}^{\boldsymbol{1000}}$


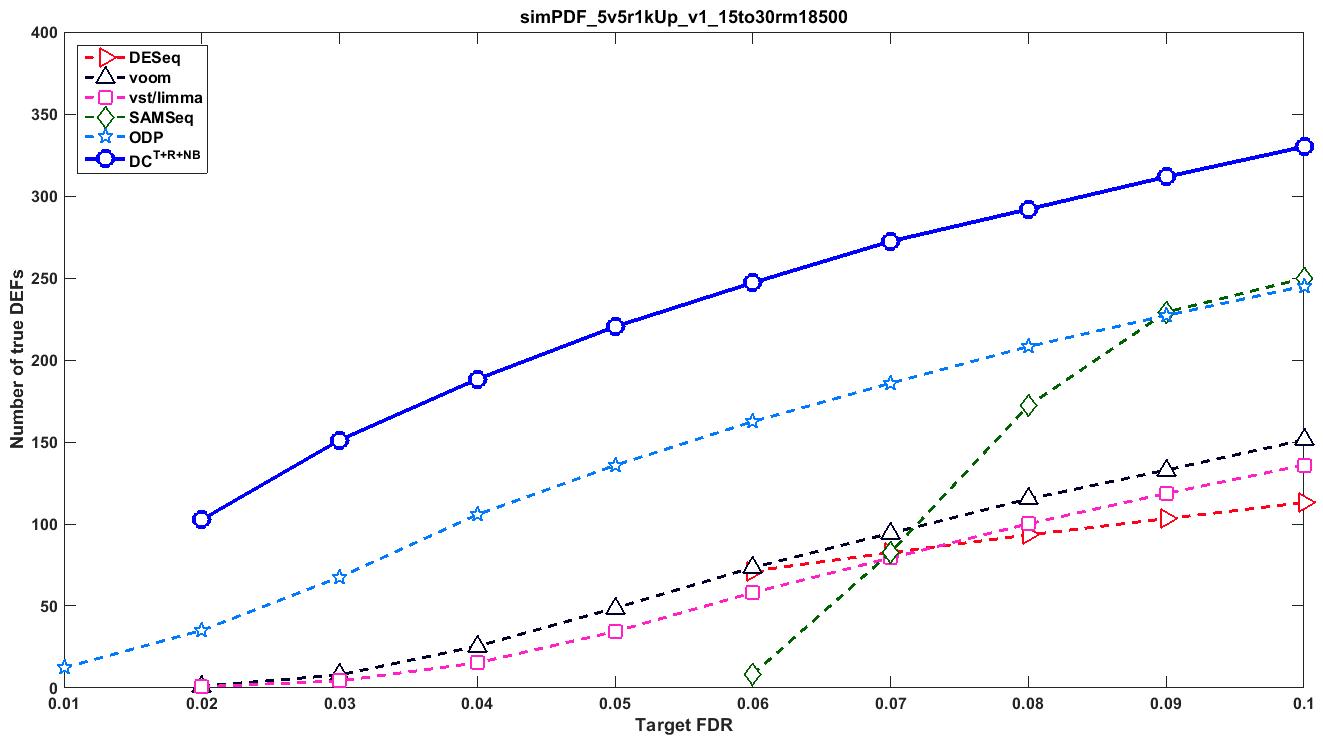


**Target FDR**

**Average number of detected true DEFs**

**Figure S37. Compare the curves of the true positives *vs.* the target FDR (5 *vs.* 5 and $\boldsymbol{G}_{\boldsymbol{0}}^{\boldsymbol{1000}}$)**. The ***x***- and ***y***- axes indicate the target FDR cutoff and the average number of true positives, respectively. The solid curve with blue circle markers represents DC*^T+R+NB^* and other curves represent non-DC methods. The result of a method at a particular target FDR is shown in this plot if (1) its average true FDR does not exceed the target FDR by 10%; and (2) its average number of true DEFs is ≥ 0.5 (rounds up to 1).

**Simulation Test Results of *N* = 12,** $\boldsymbol{G}_{\boldsymbol{0}}^{\boldsymbol{1000}}$


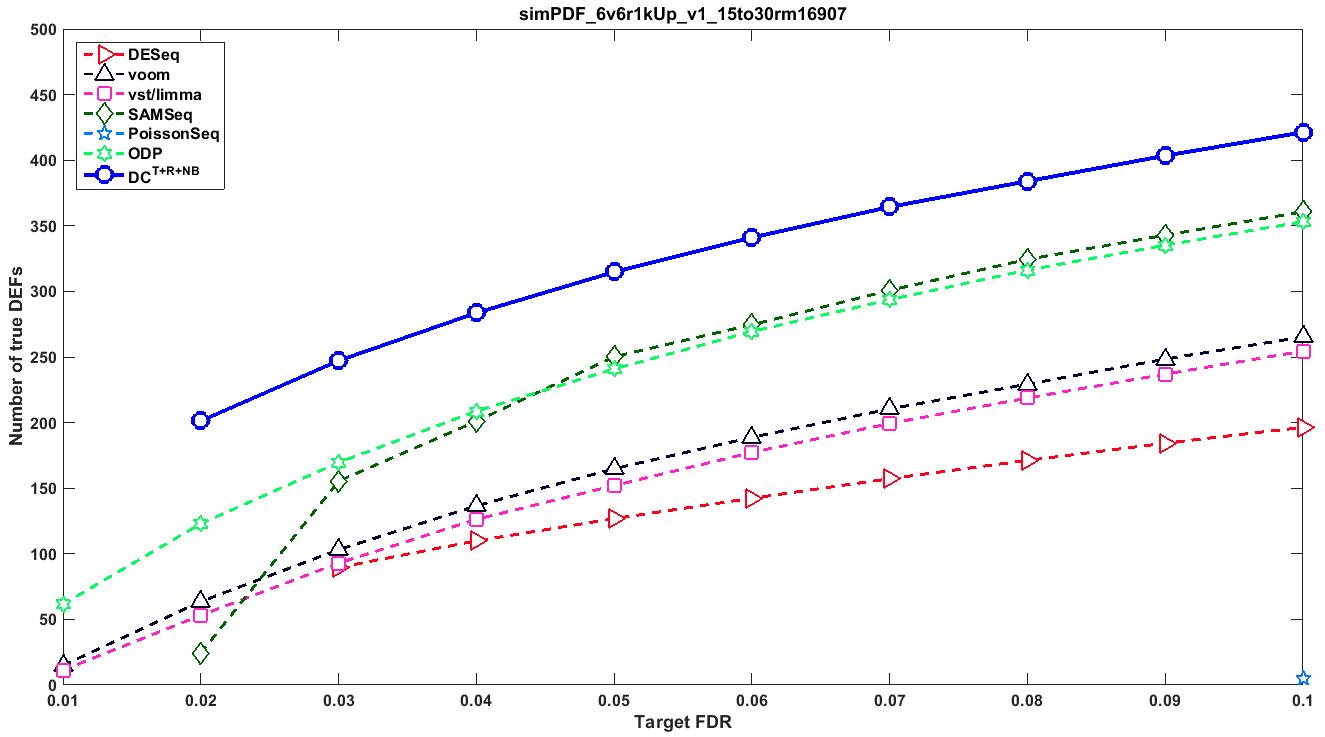


**Target FDR**

**Average number of detected true DEFs**

**Figure S38. Compare the curves of the true positives *vs.* the target FDR (6 *vs.* 6 and $\boldsymbol{G}_{\boldsymbol{0}}^{\boldsymbol{1000}}$)**. The ***x***- and ***y***- axes indicate the target FDR cutoff and the average number of true positives, respectively. The solid curve with blue circle markers represents DC*^T+R+NB^* and other curves represent non-DC methods. The result of a method at a particular target FDR is shown in this plot if (1) its average true FDR does not exceed the target FDR by 10%; and (2) its average number of true DEFs is ≥ 0.5 (rounds up to 1).

**Simulation Test Results of *N* = 16,** $\boldsymbol{G}_{\boldsymbol{0}}^{\boldsymbol{1000}}$


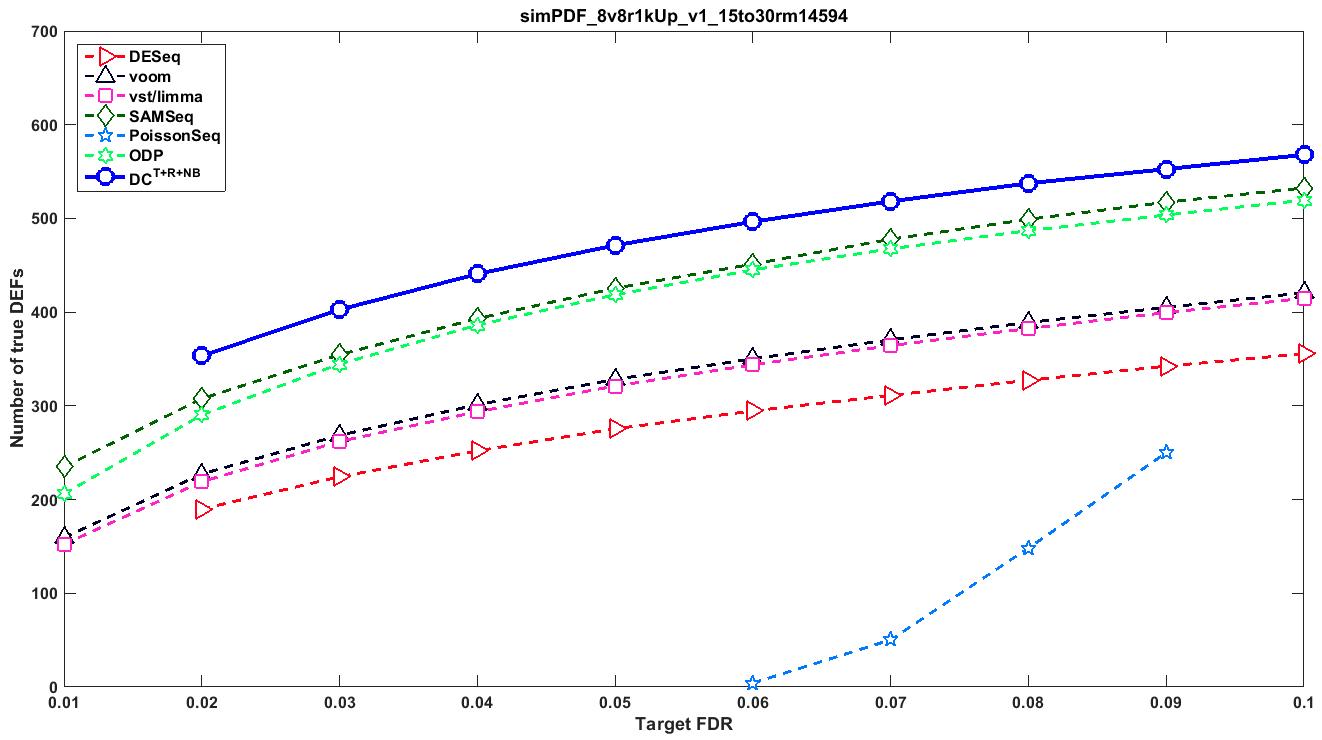


**Target FDR**

**Average number of detected true DEFs**

**Figure S39. Compare the curves of the true positives *vs.* the target FDR (8 *vs.* 8 and $\boldsymbol{G}_{\boldsymbol{0}}^{\boldsymbol{1000}}$)**. The ***x***- and ***y***- axes indicate the target FDR cutoff and the average number of true positives, respectively. The solid curve with blue circle markers represents DC*^T+R+NB^* and other curves represent non-DC methods. The result of a method at a particular target FDR is shown in this plot if (1) its average true FDR does not exceed the target FDR by 10%; and (2) its average number of true DEFs is ≥ 0.5 (rounds up to 1).

**Simulation Test Results of *N* = 20,** $\boldsymbol{G}_{\mathbf{0}}^{\mathbf{1000}}$


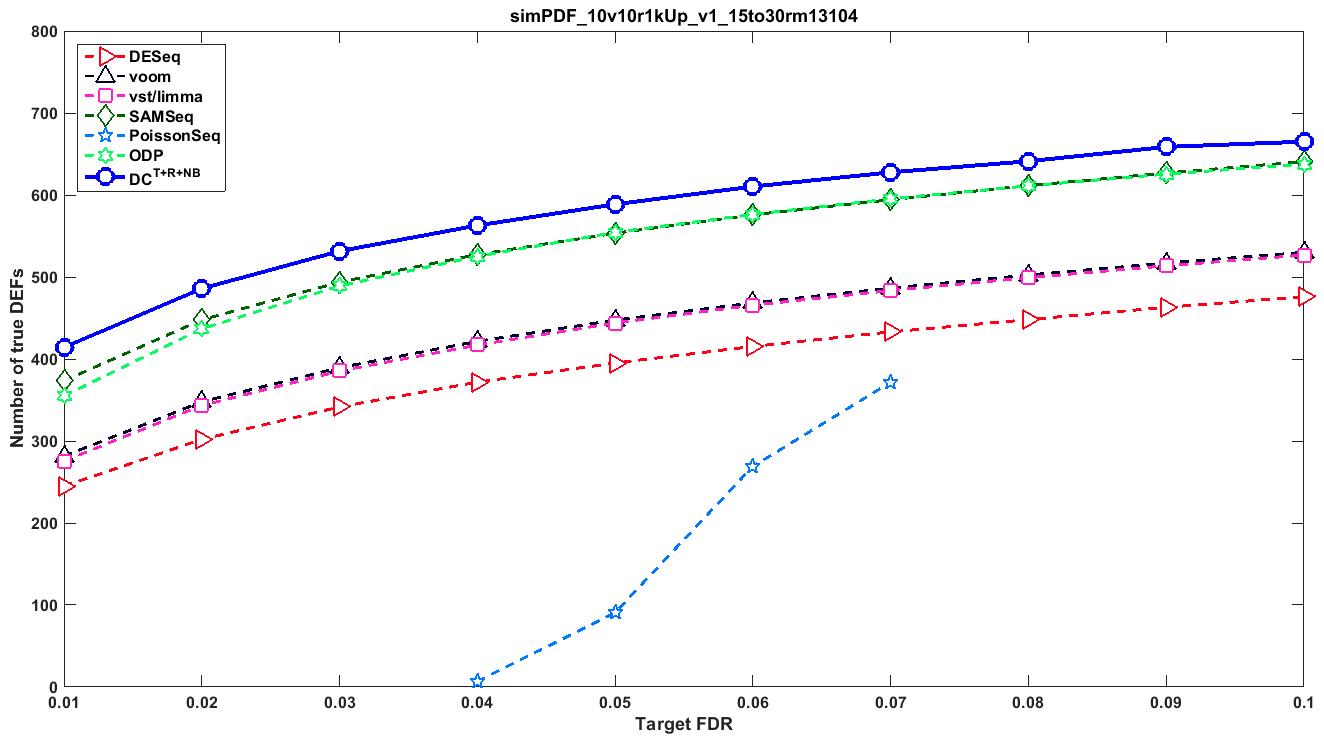


**Target FDR**

**Average number of detected true DEFs**

**Figure S40. Compare the curves of the true positives *vs.* the target FDR (10 *vs.* 10 and $\boldsymbol{G}_{\boldsymbol{0}}^{\boldsymbol{1000}}$)**. The ***x***- and ***y***- axes indicate the target FDR cutoff and the average number of true positives, respectively. The solid curve with blue circle markers represents DC*^T+R+NB^* and other curves represent non-DC methods. The result of a method at a particular target FDR is shown in this plot if (1) its average true FDR does not exceed the target FDR by 10%; and (2) its average number of true DEFs is ≥ 0.5 (rounds up to 1).

## Curves of True FDR against Number of Detected DEFs

**Simulation Test Results of *N* = 8,** $\boldsymbol{G}_{\mathbf{500}}^{\mathbf{500}}$


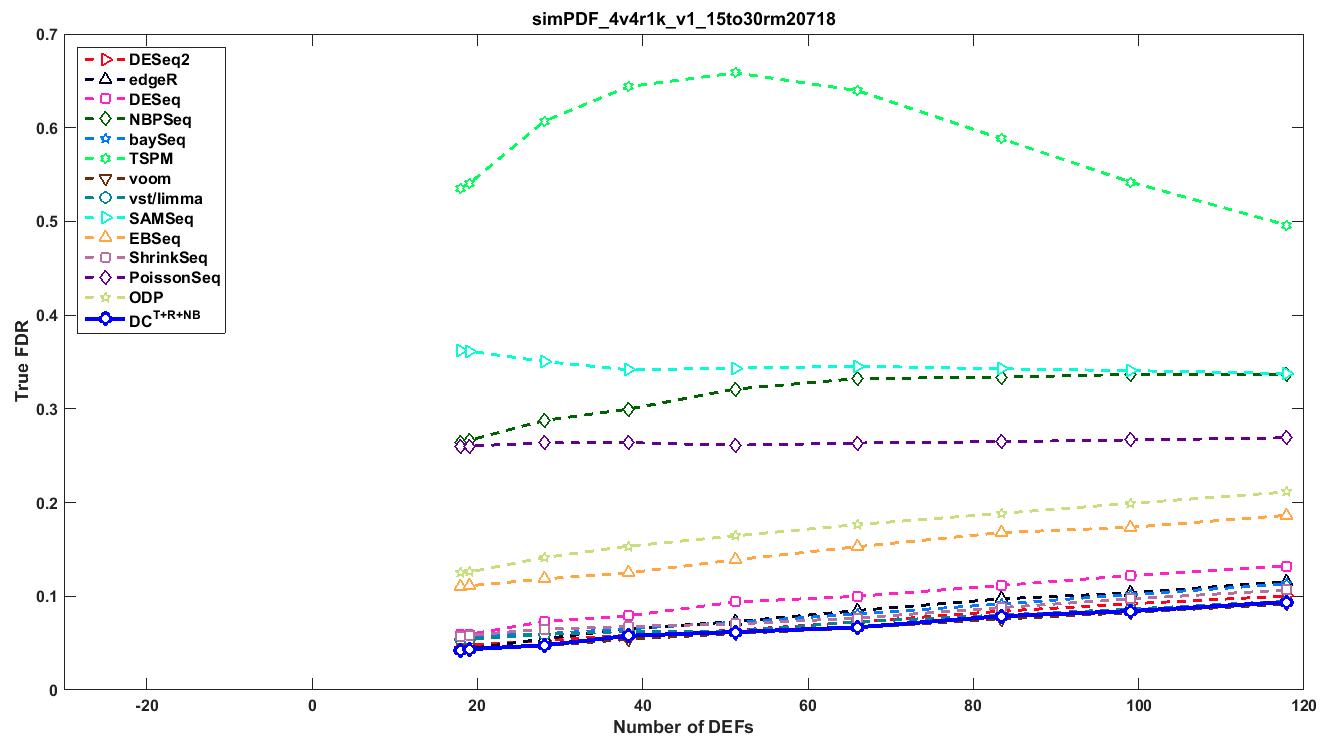


**Number of detected DEFs**

**True FDR**

**Figure S41. The curves of the true FDR *vs.* the number of detected DEFs in a typical simulation test (4 *vs.* 4; $\boldsymbol{G}_{\boldsymbol{500}}^{\boldsymbol{500}}$)**. The ***x***- and ***y***- axes indicate the number of detected DEFs and the average true FDR, respectively. The curve of DC*^T+R+NB^* (solid curve with blue circle markers) in this figure were converted from the results obtained by setting the target FDR between 0.01 and 0.1 with an increasing step of 0.01. The curves of other methods were obtained by letting them call the same number of DEFs detected by DC*^T+R+NB^* at each target FDR.

**Simulation Test Results of *N* = 10,** $\boldsymbol{G}_{\mathbf{500}}^{\mathbf{500}}$


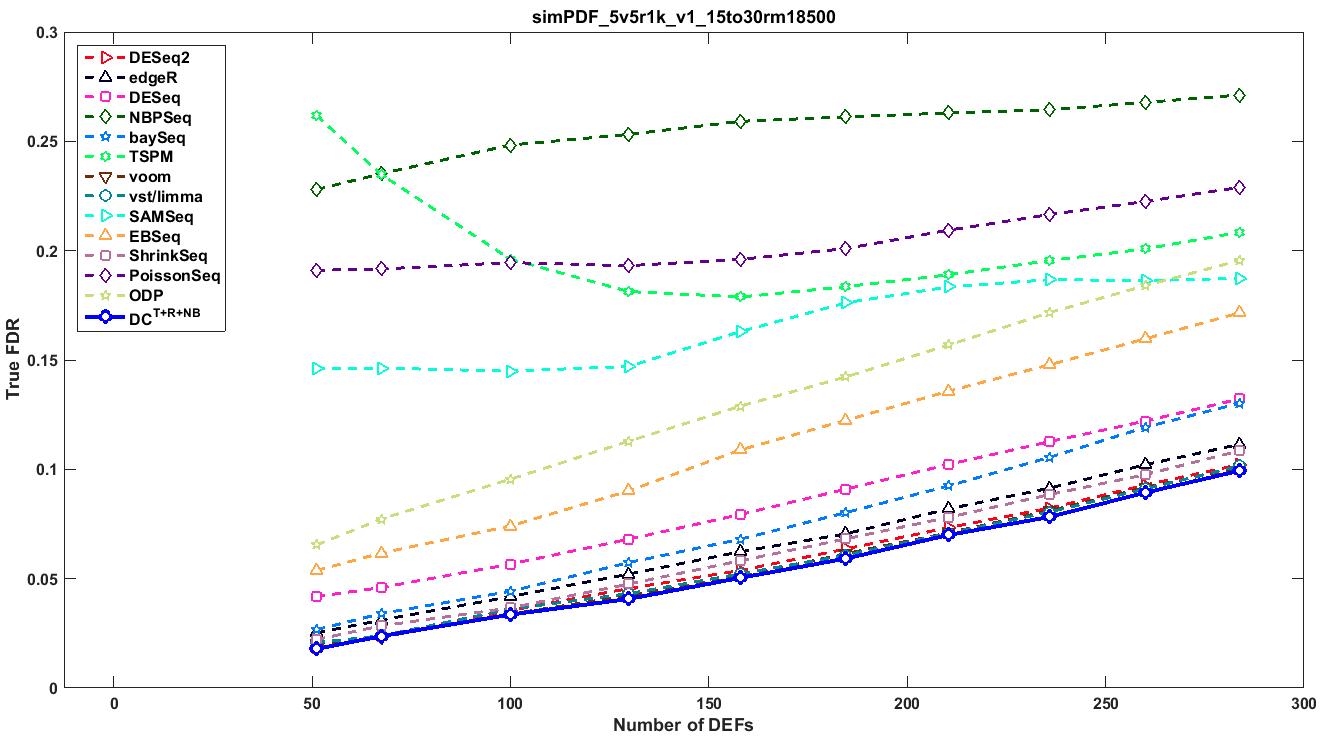


**Number of detected DEFs**

**True FDR**

**Figure S42. The curves of the true FDR *vs.* the number of detected DEFs in a typical simulation test (5 *vs.* 5; $\boldsymbol{G}_{\boldsymbol{500}}^{\boldsymbol{500}}$)**. The ***x***- and ***y***- axes indicate the number of detected DEFs and the average true FDR, respectively. The curve of DC*^T+R+NB^* (solid curve with blue circle markers) in this figure were converted from the results obtained by setting the target FDR between 0.01 and 0.1 with an increasing step of 0.01. The curves of other methods were obtained by letting them call the same number of DEFs detected by DC*^T+R+NB^* at each target FDR.

**Simulation Test Results of *N* = 12,** $\boldsymbol{G}_{\mathbf{500}}^{\mathbf{500}}$


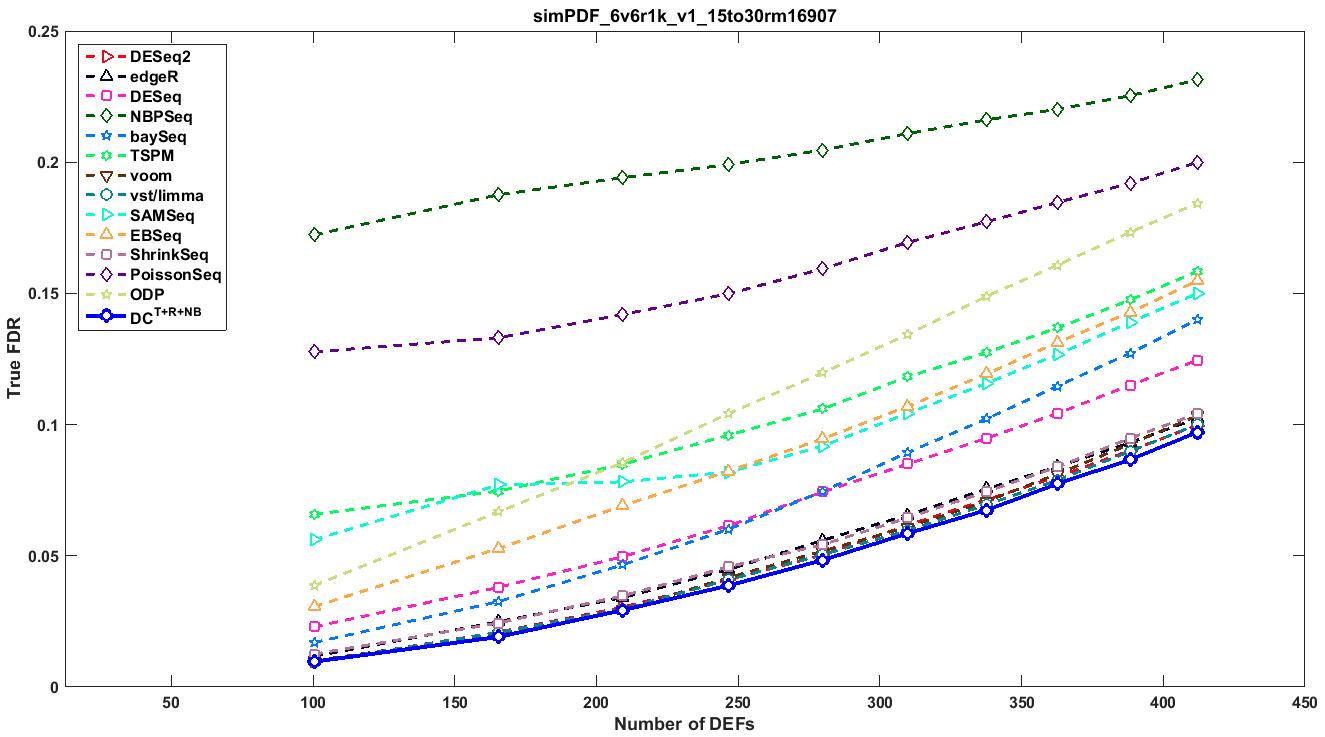


**Number of detected DEFs**

**True FDR**

**Figure S43. The curves of the true FDR *vs.* the number of detected DEFs in a typical simulation test (6 *vs.* 6; $\boldsymbol{G}_{\boldsymbol{500}}^{\boldsymbol{500}}$)**. The ***x***- and ***y***- axes indicate the number of detected DEFs and the average true FDR, respectively. The curve of DC*^T+R+NB^* (solid curve with blue circle markers) in this figure were converted from the results obtained by setting the target FDR between 0.01 and 0.1 with an increasing step of 0.01. The curves of other methods were obtained by letting them call the same number of DEFs detected by DC*^T+R+NB^* at each target FDR.

**Simulation Test Results of *N* = 16,** $\boldsymbol{G}_{\mathbf{500}}^{\mathbf{500}}$


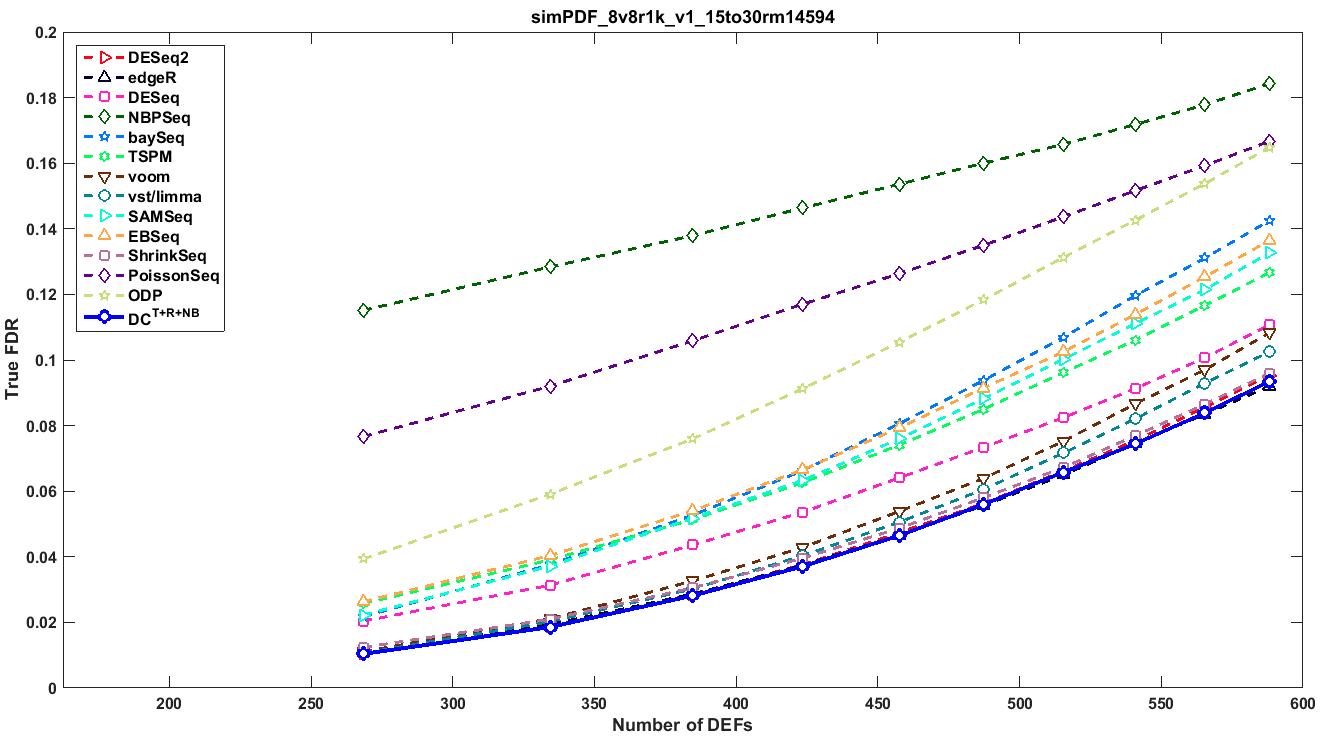


**Number of detected DEFs**

**True FDR**

**Figure S44. The curves of the true FDR *vs.* the number of detected DEFs in a typical simulation test (8 *vs.* 8; $\boldsymbol{G}_{\boldsymbol{500}}^{\boldsymbol{500}}$)**. The ***x***- and ***y***- axes indicate the number of detected DEFs and the average true FDR, respectively. The curve of DC*^T+R+NB^* (solid curve with blue circle markers) in this figure were converted from the results obtained by setting the target FDR between 0.01 and 0.1 with an increasing step of 0.01. The curves of other methods were obtained by letting them call the same number of DEFs detected by DC*^T+R+NB^* at each target FDR.

**Simulation Test Results of *N* = 20,** $\boldsymbol{G}_{\mathbf{500}}^{\mathbf{500}}$


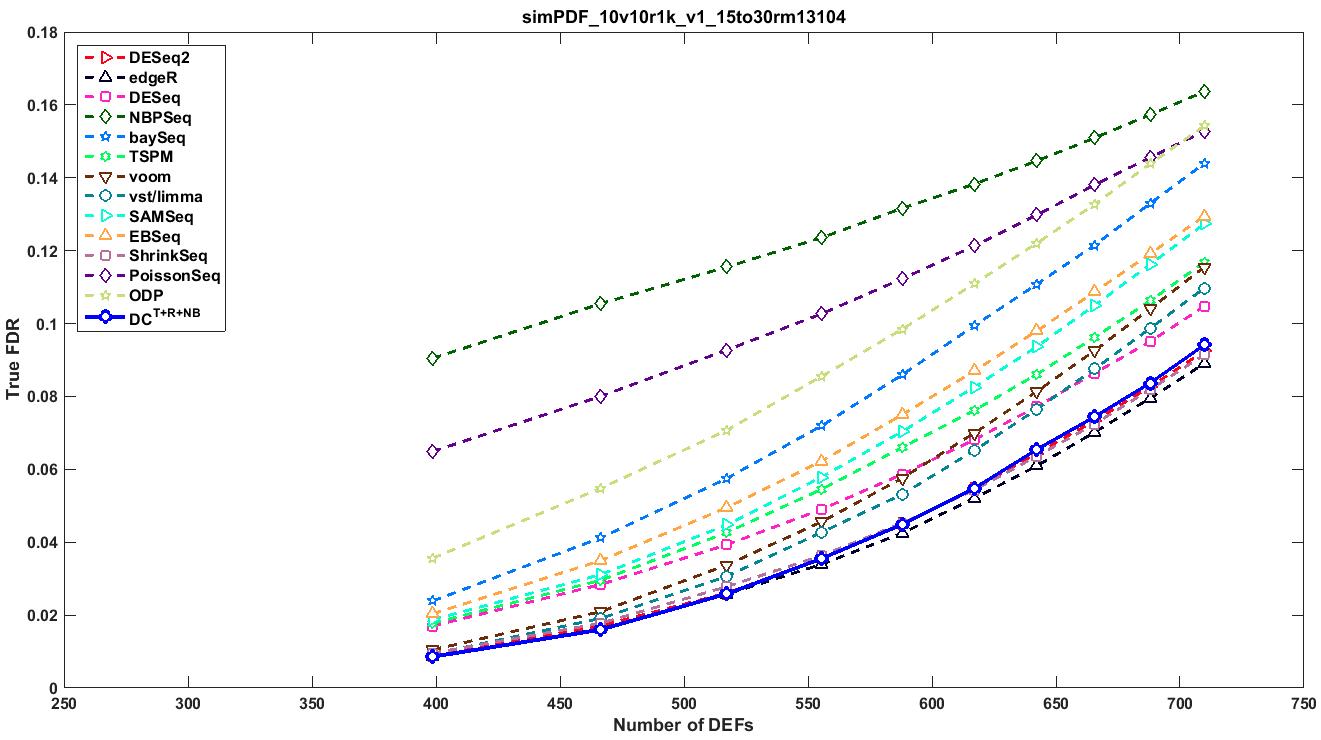


**Number of detected DEFs**

**True FDR**

**Figure S45. The curves of the true FDR *vs.* the number of detected DEFs in a typical simulation test (10 *vs.* 10; $\boldsymbol{G}_{\boldsymbol{500}}^{\boldsymbol{500}}$)**. The ***x***- and ***y***- axes indicate the number of detected DEFs and the average true FDR, respectively. The curve of DC*^T+R+NB^* (solid curve with blue circle markers) in this figure were converted from the results obtained by setting the target FDR between 0.01 and 0.1 with an increasing step of 0.01. The curves of other methods were obtained by letting them call the same number of DEFs detected by DC*^T+R+NB^* at each target FDR.

**Simulation Test Results of *N* = 8,** $\boldsymbol{G}_{\mathbf{600}}^{\mathbf{600}}$


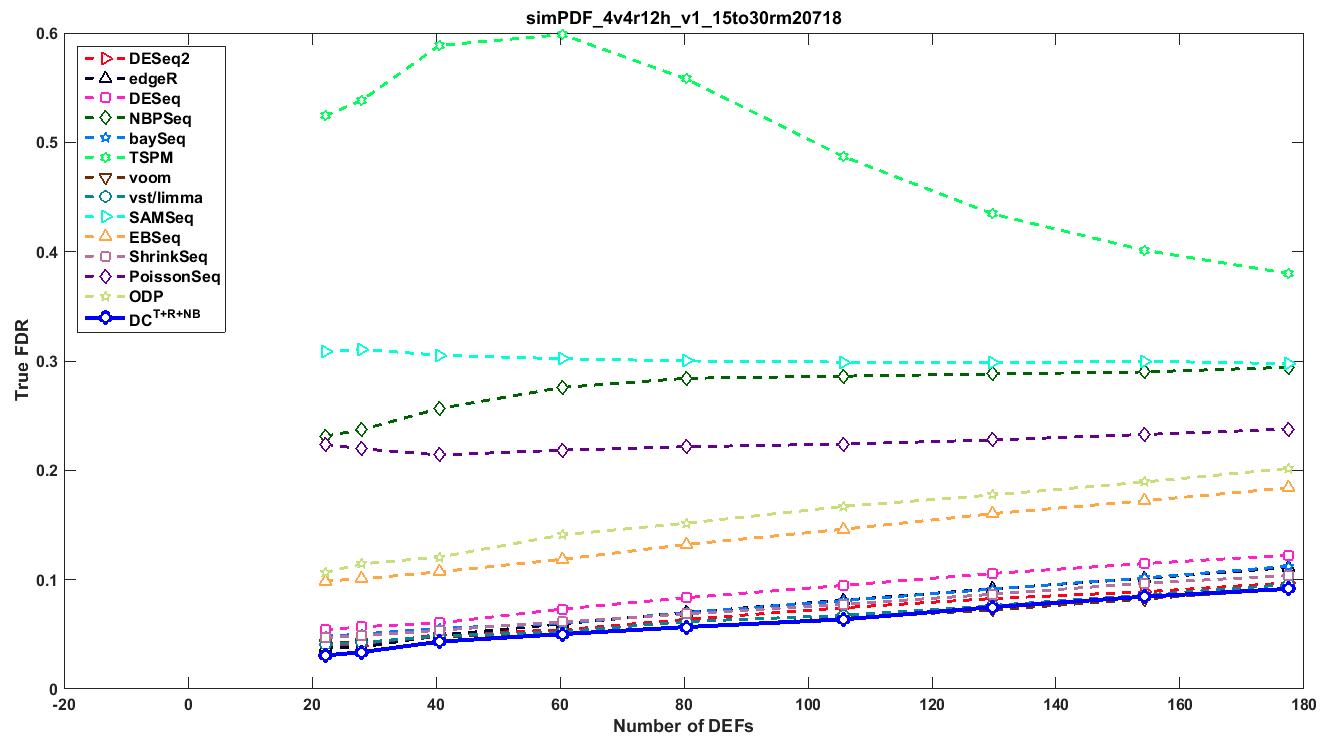


**Number of detected DEFs**

**True FDR**

**Figure S46. The curves of the true FDR *vs.* the number of detected DEFs in a typical simulation test (4 *vs.* 4; $\boldsymbol{G}_{\boldsymbol{600}}^{\boldsymbol{600}}$)**. The ***x***- and ***y***- axes indicate the number of detected DEFs and the average true FDR, respectively. The curve of DC*^T+R+NB^* (solid curve with blue circle markers) in this figure were converted from the results obtained by setting the target FDR between 0.01 and 0.1 with an increasing step of 0.01. The curves of other methods were obtained by letting them call the same number of DEFs detected by DC*^T+R+NB^* at each target FDR.

**Simulation Test Results of *N* = 10,** $\boldsymbol{G}_{\mathbf{600}}^{\mathbf{600}}$


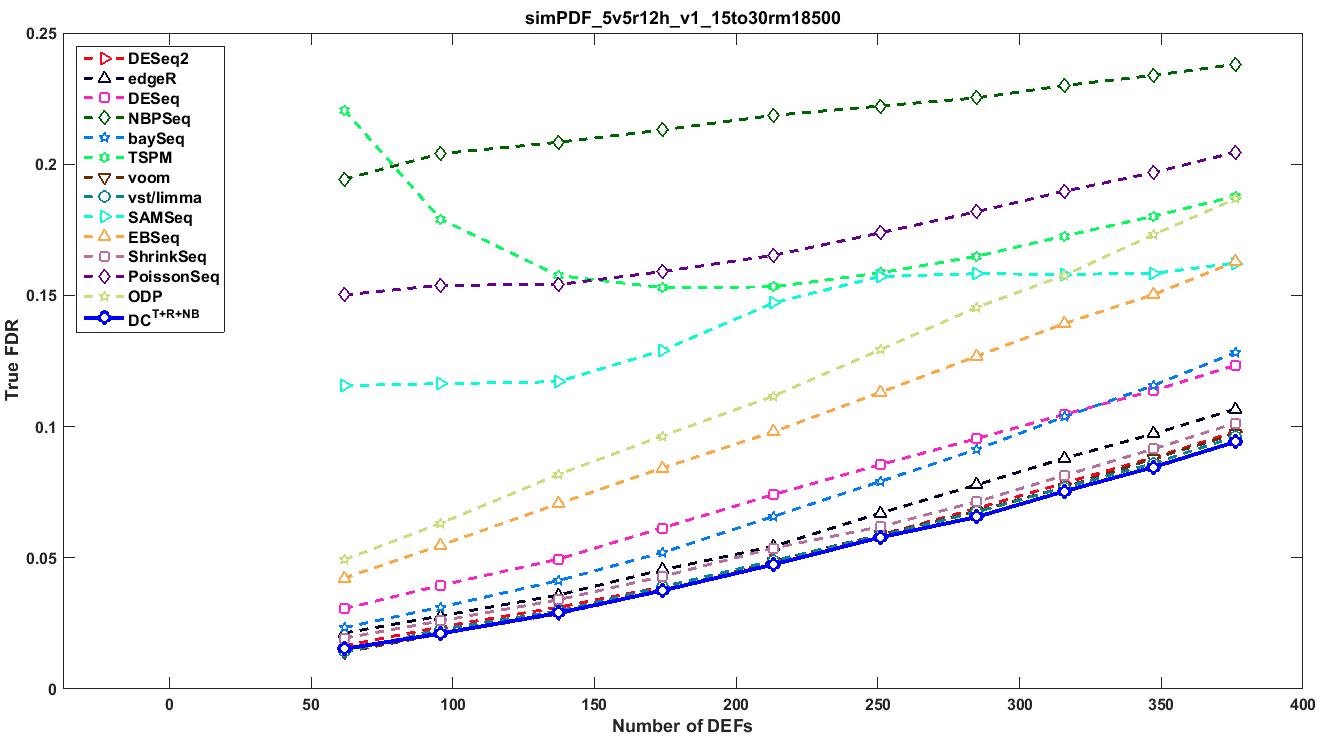


**Number of detected DEFs**

**True FDR**

**Figure S47. The curves of the true FDR *vs.* the number of detected DEFs in a typical simulation test (5 *vs.* 5; $\boldsymbol{G}_{\boldsymbol{600}}^{\boldsymbol{600}}$)**. The ***x***- and ***y***- axes indicate the number of detected DEFs and the average true FDR, respectively. The curve of DC*^T+R+NB^* (solid curve with blue circle markers) in this figure were converted from the results obtained by setting the target FDR between 0.01 and 0.1 with an increasing step of 0.01. The curves of other methods were obtained by letting them call the same number of DEFs detected by DC*^T+R+NB^* at each target FDR.

**Simulation Test Results of *N* = 12,** $\boldsymbol{G}_{\mathbf{600}}^{\mathbf{600}}$


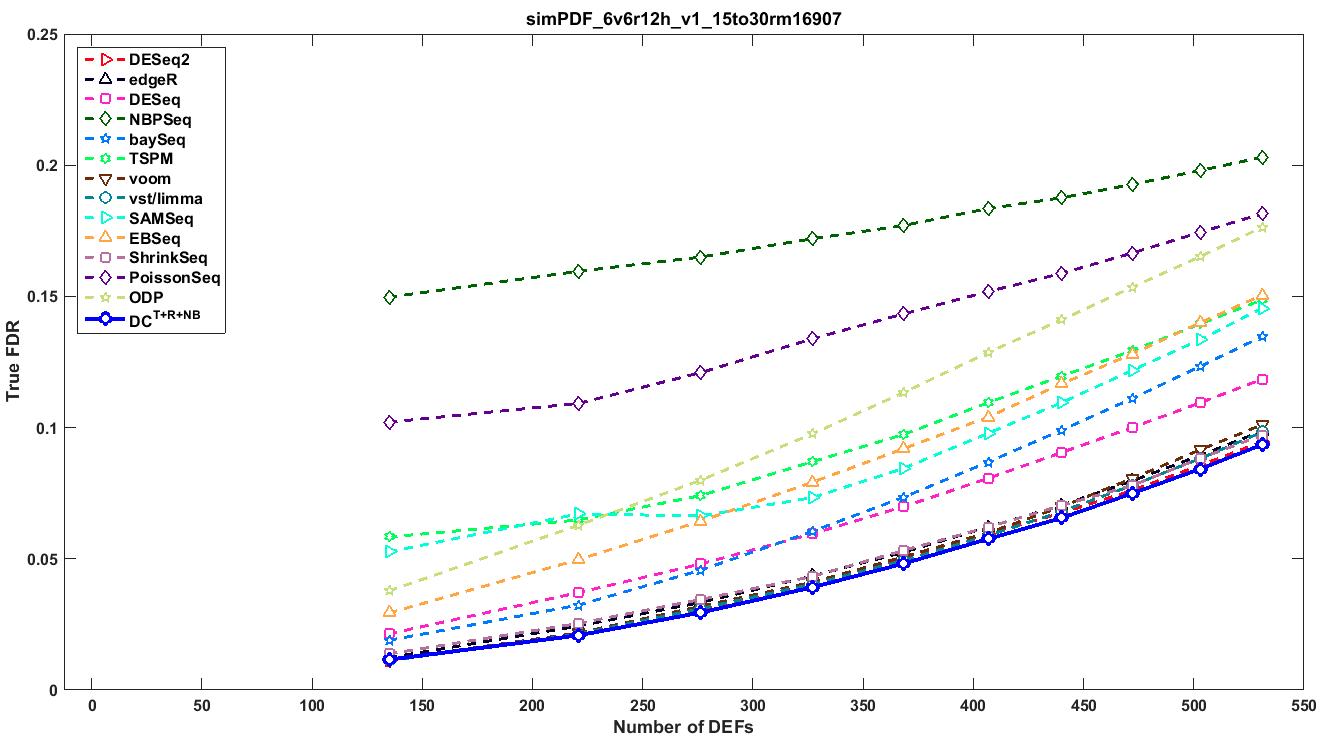


**Number of detected DEFs**

**True FDR**

**Figure S48. The curves of the true FDR *vs.* the number of detected DEFs in a typical simulation test (6 *vs.* 6; $\boldsymbol{G}_{\boldsymbol{600}}^{\boldsymbol{600}}$)**. The ***x***- and ***y***- axes indicate the number of detected DEFs and the average true FDR, respectively. The curve of DC*^T+R+NB^* (solid curve with blue circle markers) in this figure were converted from the results obtained by setting the target FDR between 0.01 and 0.1 with an increasing step of 0.01. The curves of other methods were obtained by letting them call the same number of DEFs detected by DC*^T+R+NB^* at each target FDR.

**Simulation Test Results of *N* = 16,** $\boldsymbol{G}_{\mathbf{600}}^{\mathbf{600}}$


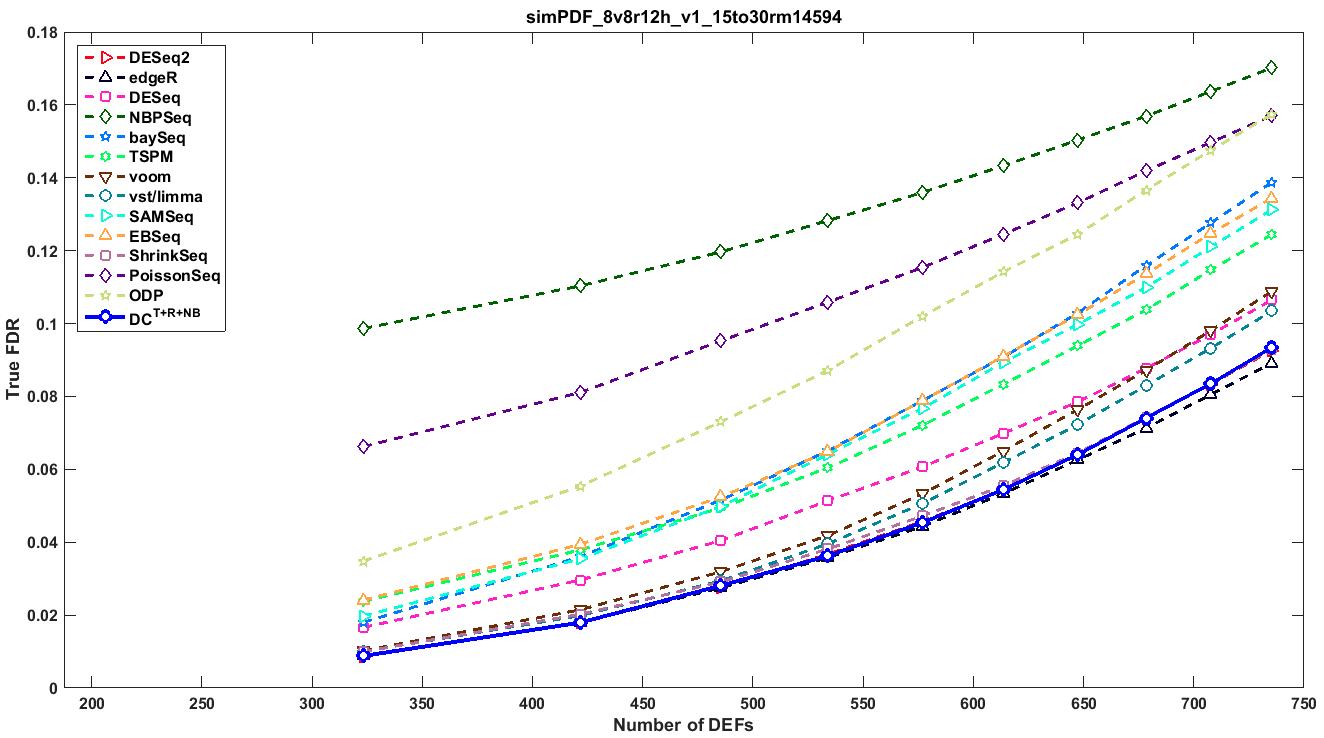


**Number of detected DEFs**

**True FDR**

**Figure S49. The curves of the true FDR *vs.* the number of detected DEFs in a typical simulation test (8 *vs.* 8; $\boldsymbol{G}_{\boldsymbol{600}}^{\boldsymbol{600}}$)**. The ***x***- and ***y***- axes indicate the number of detected DEFs and the average true FDR, respectively. The curve of DC*^T+R+NB^* (solid curve with blue circle markers) in this figure were converted from the results obtained by setting the target FDR between 0.01 and 0.1 with an increasing step of 0.01. The curves of other methods were obtained by letting them call the same number of DEFs detected by DC*^T+R+NB^* at each target FDR.

**Simulation Test Results of *N* = 20,** $\boldsymbol{G}_{\mathbf{600}}^{\mathbf{600}}$


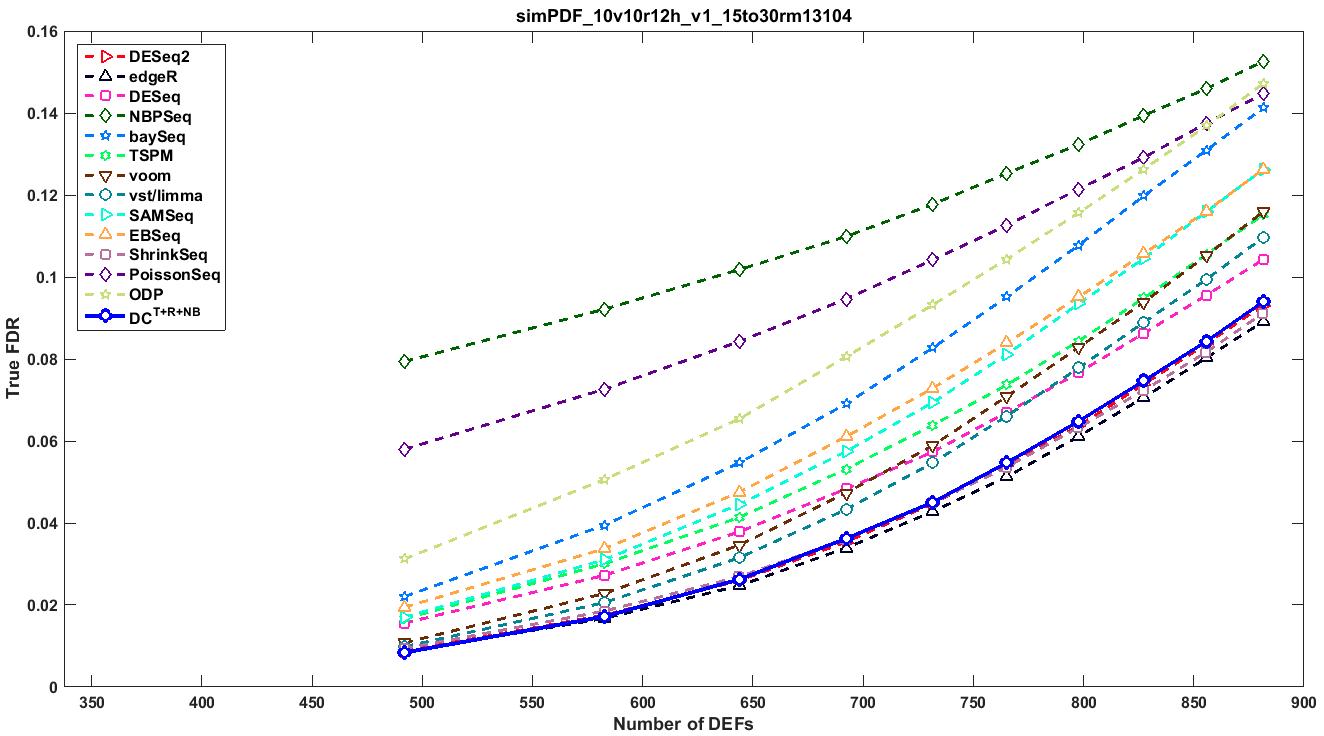


**Number of detected DEFs**

**True FDR**

**Figure S50. The curves of the true FDR *vs.* the number of detected DEFs in a typical simulation test (10 *vs.* 10; $\boldsymbol{G}_{\boldsymbol{600}}^{\boldsymbol{600}}$)**. The ***x***- and ***y***- axes indicate the number of detected DEFs and the average true FDR, respectively. The curve of DC*^T+R+NB^* (solid curve with blue circle markers) in this figure were converted from the results obtained by setting the target FDR between 0.01 and 0.1 with an increasing step of 0.01. The curves of other methods were obtained by letting them call the same number of DEFs detected by DC*^T+R+NB^* at each target FDR.

**Simulation Test Results of *N* = 8,** $\boldsymbol{G}_{\mathbf{400}}^{\mathbf{400}}$


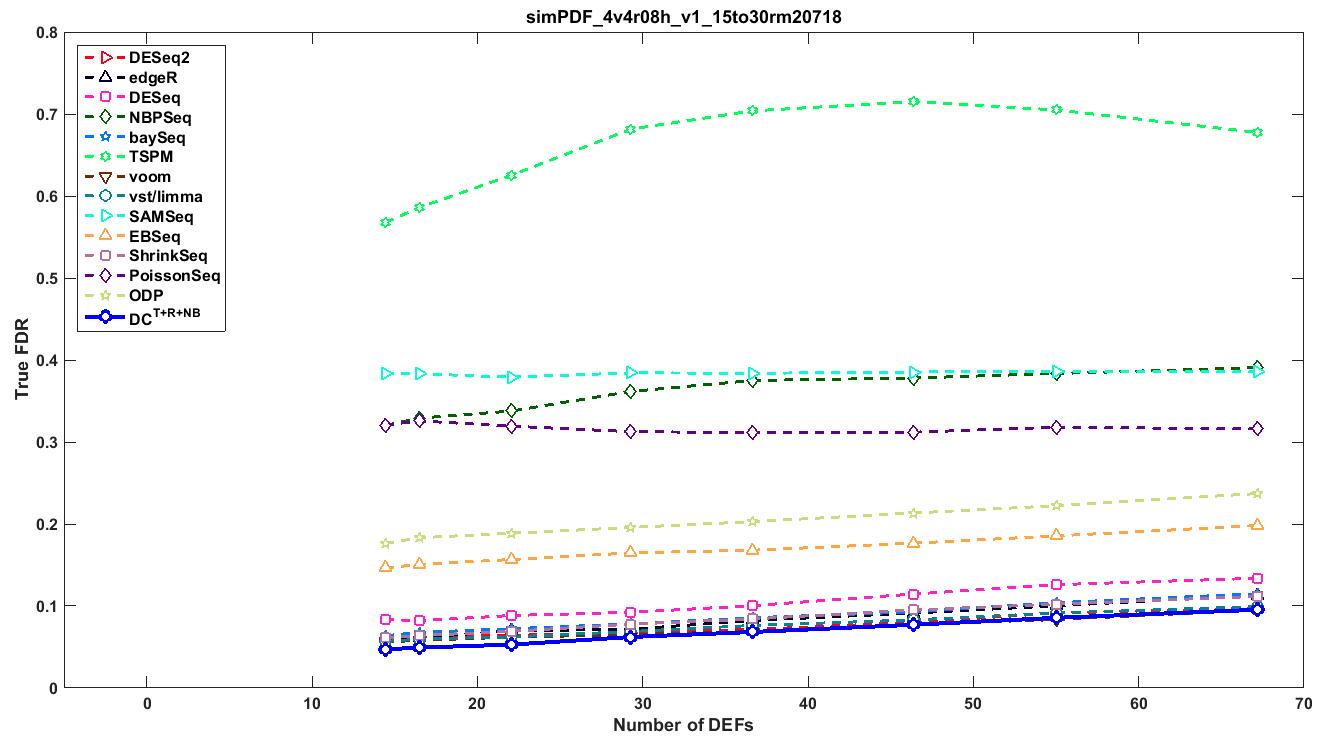


**Number of detected DEFs**

**True FDR**

**Figure S51. The curves of the true FDR *vs.* the number of detected DEFs in a typical simulation test (4 *vs.* 4; $\boldsymbol{G}_{\boldsymbol{400}}^{\boldsymbol{400}}$)**. The ***x***- and ***y***- axes indicate the number of detected DEFs and the average true FDR, respectively. The curve of DC*^T+R+NB^* (solid curve with blue circle markers) in this figure were converted from the results obtained by setting the target FDR between 0.01 and 0.1 with an increasing step of 0.01. The curves of other methods were obtained by letting them call the same number of DEFs detected by DC*^T+R+NB^* at each target FDR.

**Simulation Test Results of *N* = 10,** $\boldsymbol{G}_{\mathbf{400}}^{\mathbf{400}}$


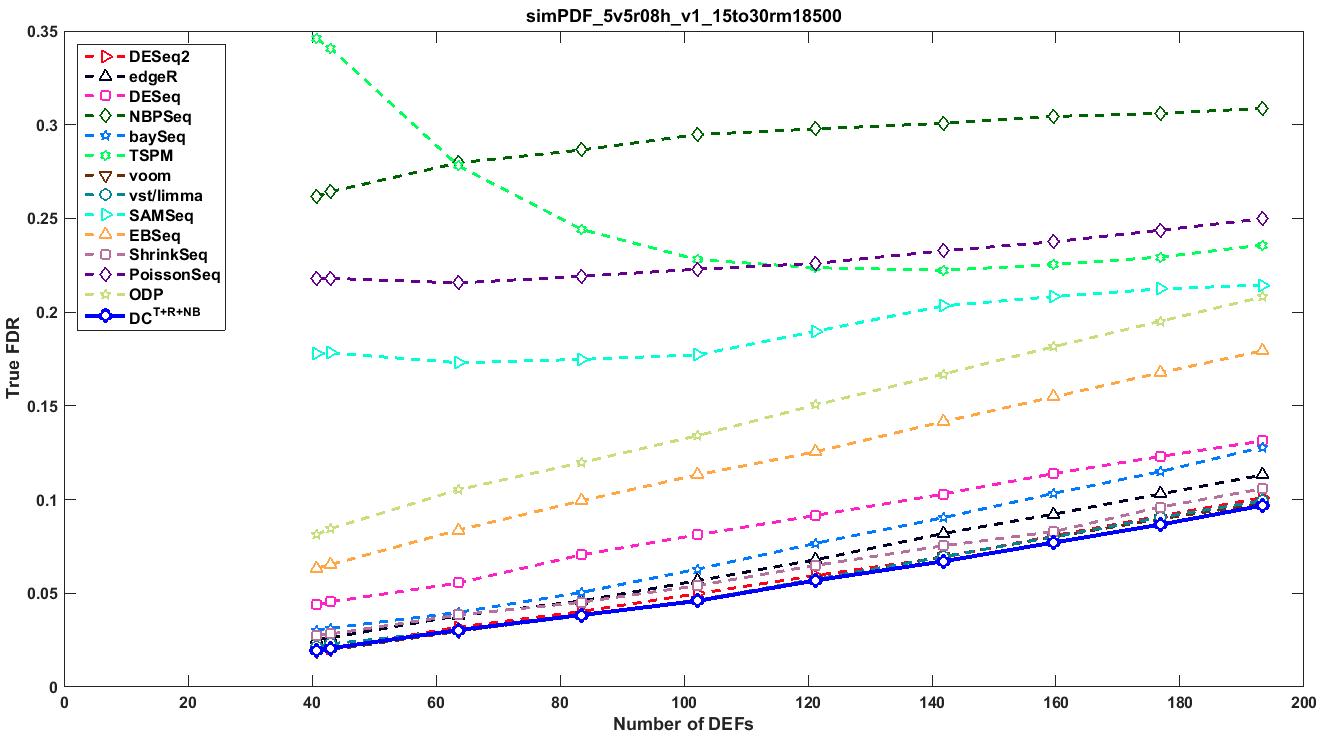


**Number of detected DEFs**

**True FDR**

**Figure S52. The curves of the true FDR *vs.* the number of detected DEFs in a typical simulation test (5 *vs.* 5; $\boldsymbol{G}_{\boldsymbol{400}}^{\boldsymbol{400}}$)**. The ***x***- and ***y***- axes indicate the number of detected DEFs and the average true FDR, respectively. The curve of DC*^T+R+NB^* (solid curve with blue circle markers) in this figure were converted from the results obtained by setting the target FDR between 0.01 and 0.1 with an increasing step of 0.01. The curves of other methods were obtained by letting them call the same number of DEFs detected by DC*^T+R+NB^* at each target FDR.

**Simulation Test Results of *N* = 12,** $\boldsymbol{G}_{\mathbf{400}}^{\mathbf{400}}$


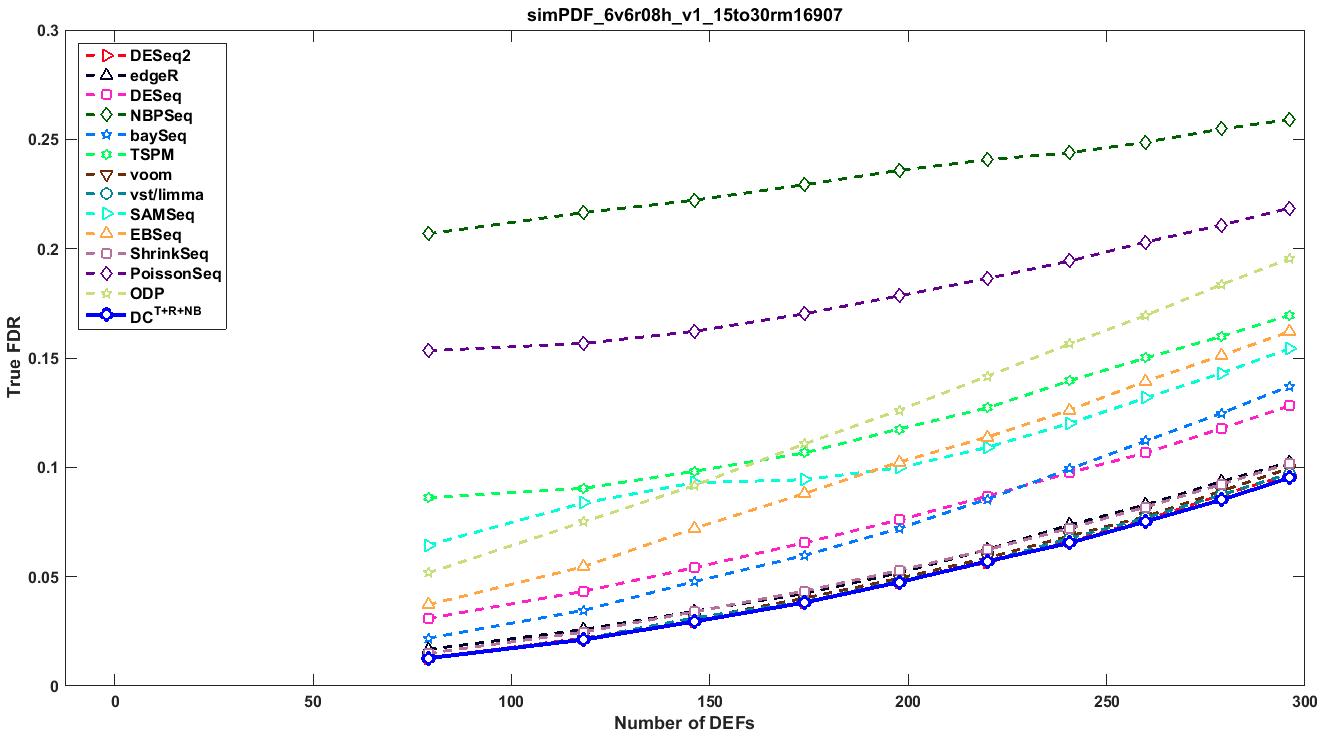


**Number of detected DEFs**

**True FDR**

**Figure S53. The curves of the true FDR *vs.* the number of detected DEFs in a typical simulation test (6 *vs.* 6; $\boldsymbol{G}_{\boldsymbol{400}}^{\boldsymbol{400}}$)**. The ***x***- and ***y***- axes indicate the number of detected DEFs and the average true FDR, respectively. The curve of DC*^T+R+NB^* (solid curve with blue circle markers) in this figure were converted from the results obtained by setting the target FDR between 0.01 and 0.1 with an increasing step of 0.01. The curves of other methods were obtained by letting them call the same number of DEFs detected by DC*^T+R+NB^* at each target FDR.

**Simulation Test Results of *N* = 16,** $\boldsymbol{G}_{\mathbf{400}}^{\mathbf{400}}$


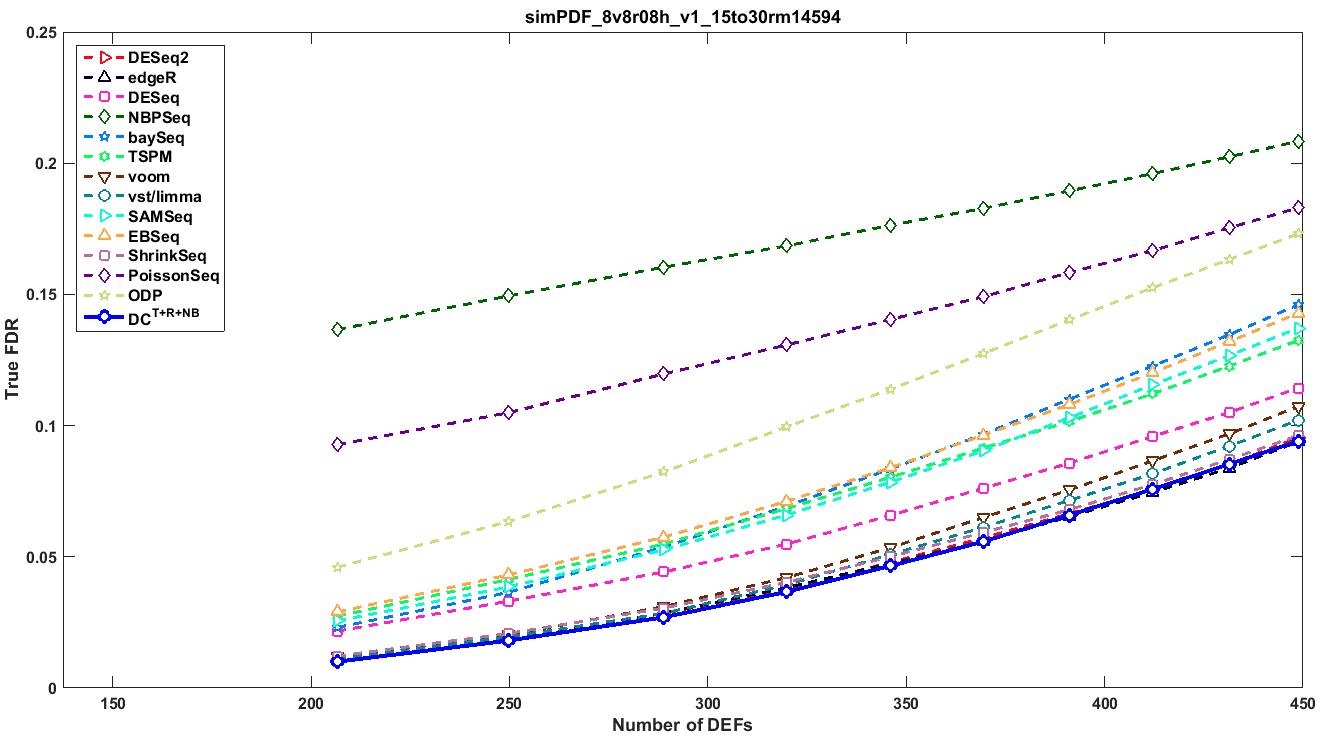


**Number of detected DEFs**

**True FDR**

**Figure S54. The curves of the true FDR *vs.* the number of detected DEFs in a typical simulation test (8 *vs.* 8; $\boldsymbol{G}_{\boldsymbol{400}}^{\boldsymbol{400}}$)**. The ***x***- and ***y***- axes indicate the number of detected DEFs and the average true FDR, respectively. The curve of DC*^T+R+NB^* (solid curve with blue circle markers) in this figure were converted from the results obtained by setting the target FDR between 0.01 and 0.1 with an increasing step of 0.01. The curves of other methods were obtained by letting them call the same number of DEFs detected by DC*^T+R+NB^* at each target FDR.

**Simulation Test Results of *N* = 20,** $\boldsymbol{G}_{\mathbf{400}}^{\mathbf{400}}$


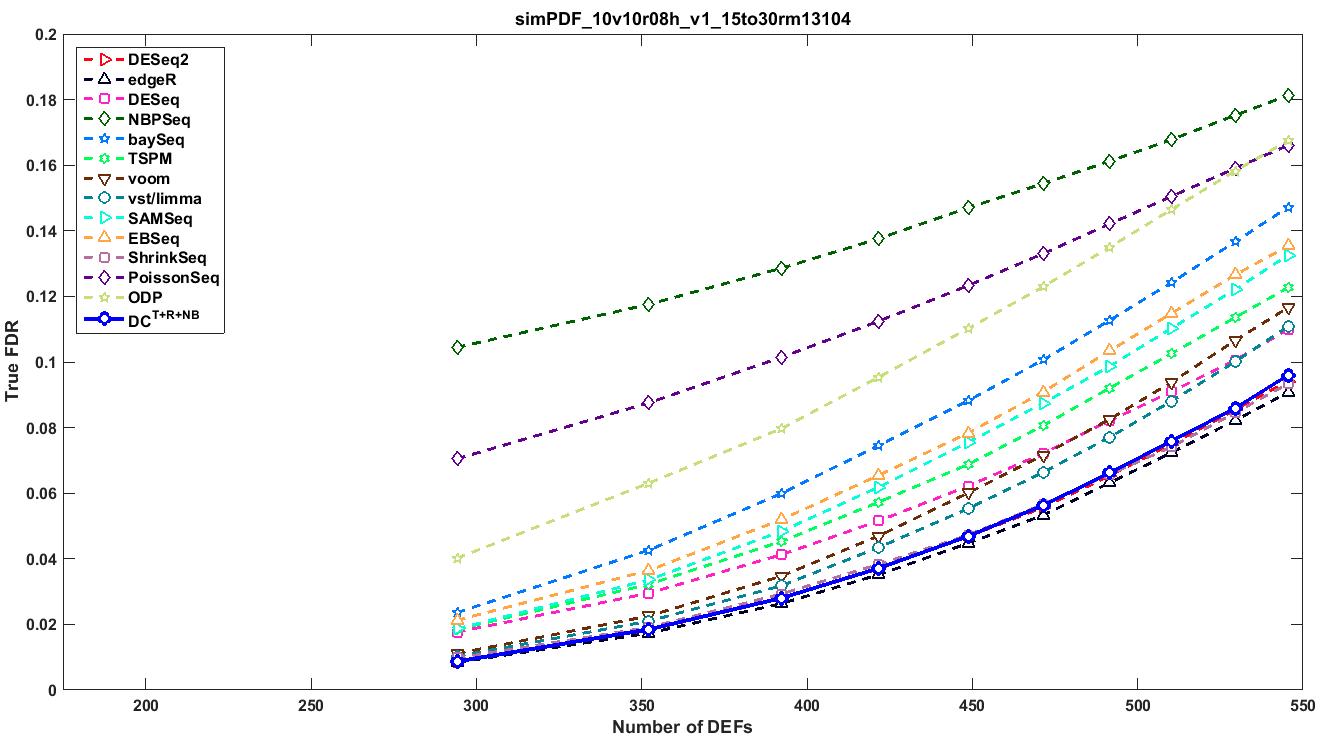


**Number of detected DEFs**

**True FDR**

**Figure S55. The curves of the true FDR *vs.* the number of detected DEFs in a typical simulation test (10 *vs.* 10; $\boldsymbol{G}_{\boldsymbol{400}}^{\boldsymbol{400}}$)**. The ***x***- and ***y***- axes indicate the number of detected DEFs and the average true FDR, respectively. The curve of DC*^T+R+NB^* (solid curve with blue circle markers) in this figure were converted from the results obtained by setting the target FDR between 0.01 and 0.1 with an increasing step of 0.01. The curves of other methods were obtained by letting them call the same number of DEFs detected by DC*^T+R+NB^* at each target FDR.

**Simulation Test Results of *N* = 8,** $\boldsymbol{G}_{\mathbf{0}}^{\mathbf{1000}}$


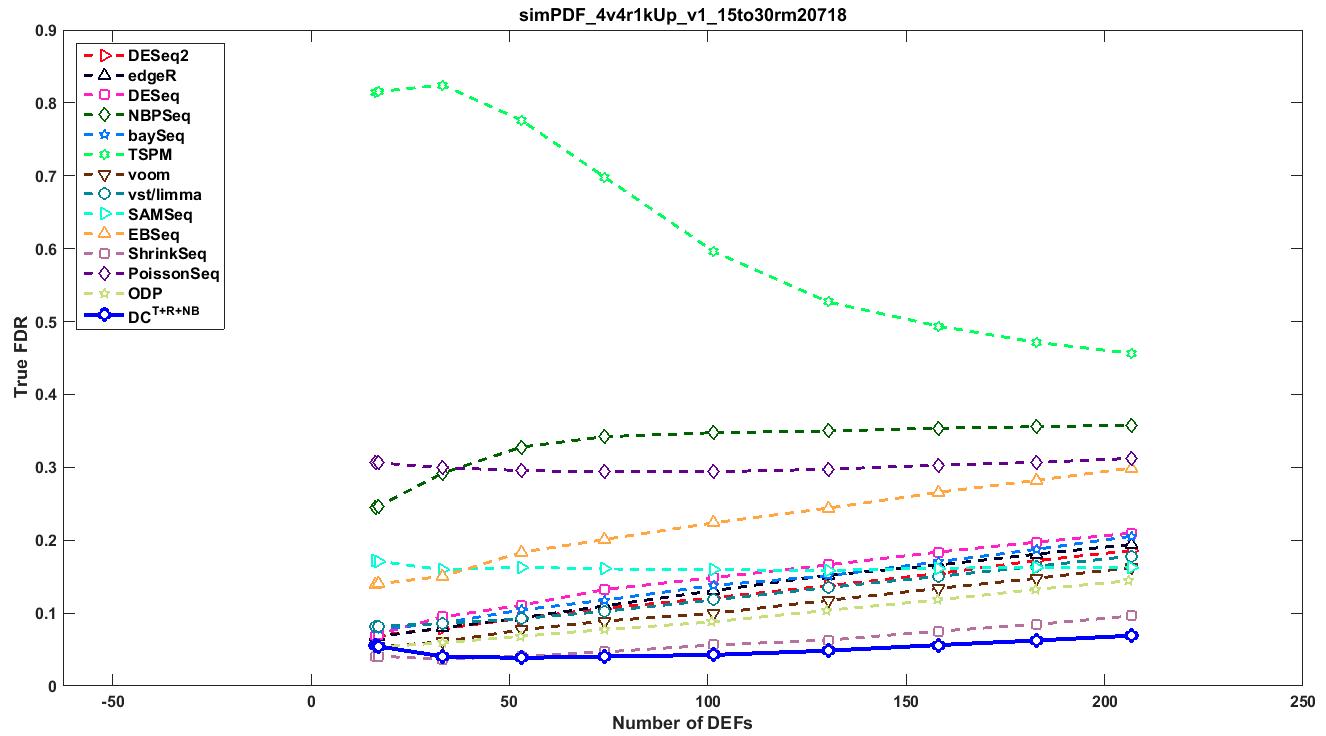


**Number of detected DEFs**

**True FDR**

**Figure S56. The curves of the true FDR *vs.* the number of detected DEFs in a typical simulation test (4 *vs.* 4; $\boldsymbol{G}_{\boldsymbol{0}}^{\boldsymbol{1000}}$)**. The ***x***- and ***y***- axes indicate the number of detected DEFs and the average true FDR, respectively. The curve of DC*^T+R+NB^* (solid curve with blue circle markers) in this figure were converted from the results obtained by setting the target FDR between 0.01 and 0.1 with an increasing step of 0.01. The curves of other methods were obtained by letting them call the same number of DEFs detected by DC*^T+R+NB^* at each target FDR.

**Simulation Test Results of *N* = 10,** $\boldsymbol{G}_{\boldsymbol{0}}^{\boldsymbol{1000}}$


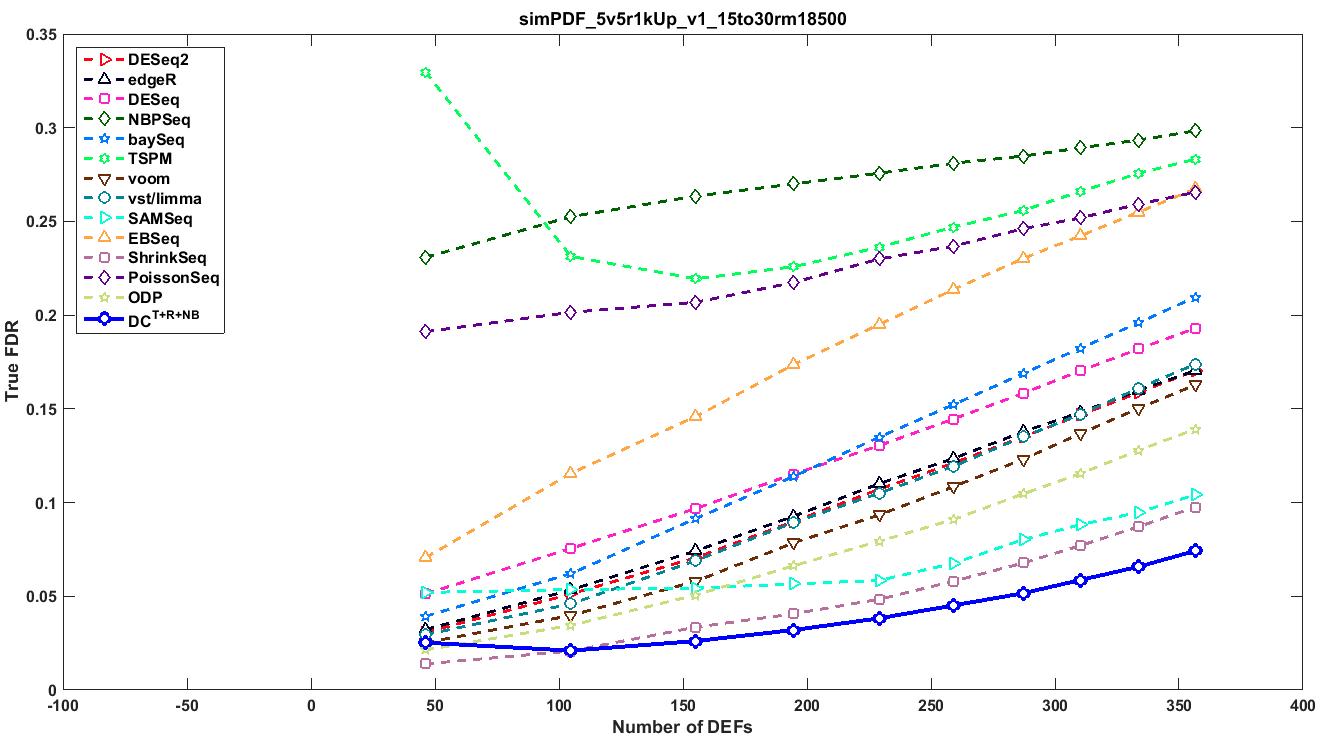


**Number of detected DEFs**

**True FDR**

**Figure S57. The curves of the true FDR *vs.* the number of detected DEFs in a typical simulation test (5 *vs.* 5; $\boldsymbol{G}_{\boldsymbol{0}}^{\boldsymbol{1000}}$)**. The ***x***- and ***y***- axes indicate the number of detected DEFs and the average true FDR, respectively. The curve of DC*^T+R+NB^* (solid curve with blue circle markers) in this figure were converted from the results obtained by setting the target FDR between 0.01 and 0.1 with an increasing step of 0.01. The curves of other methods were obtained by letting them call the same number of DEFs detected by DC*^T+R+NB^* at each target FDR.

**Simulation Test Results of *N* = 12,** $\boldsymbol{G}_{\boldsymbol{0}}^{\boldsymbol{1000}}$


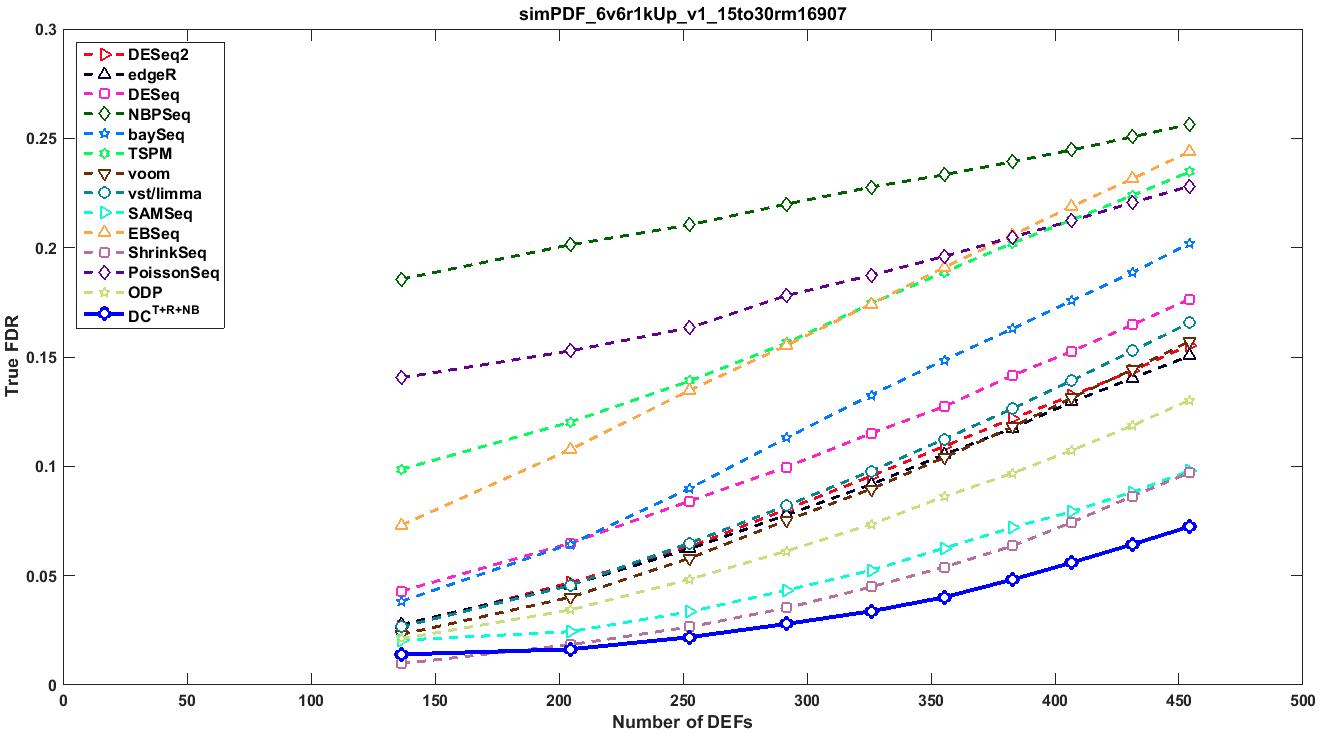


**Number of detected DEFs**

**True FDR**

**Figure S58. The curves of the true FDR *vs.* the number of detected DEFs in a typical simulation test (6 *vs.* 6; $\boldsymbol{G}_{\boldsymbol{0}}^{\boldsymbol{1000}}$)**. The ***x***- and ***y***- axes indicate the number of detected DEFs and the average true FDR, respectively. The curve of DC*^T+R+NB^* (solid curve with blue circle markers) in this figure were converted from the results obtained by setting the target FDR between 0.01 and 0.1 with an increasing step of 0.01. The curves of other methods were obtained by letting them call the same number of DEFs detected by DC*^T+R+NB^* at each target FDR.

**Simulation Test Results of *N* = 16,** $\boldsymbol{G}_{\boldsymbol{0}}^{\boldsymbol{1000}}$


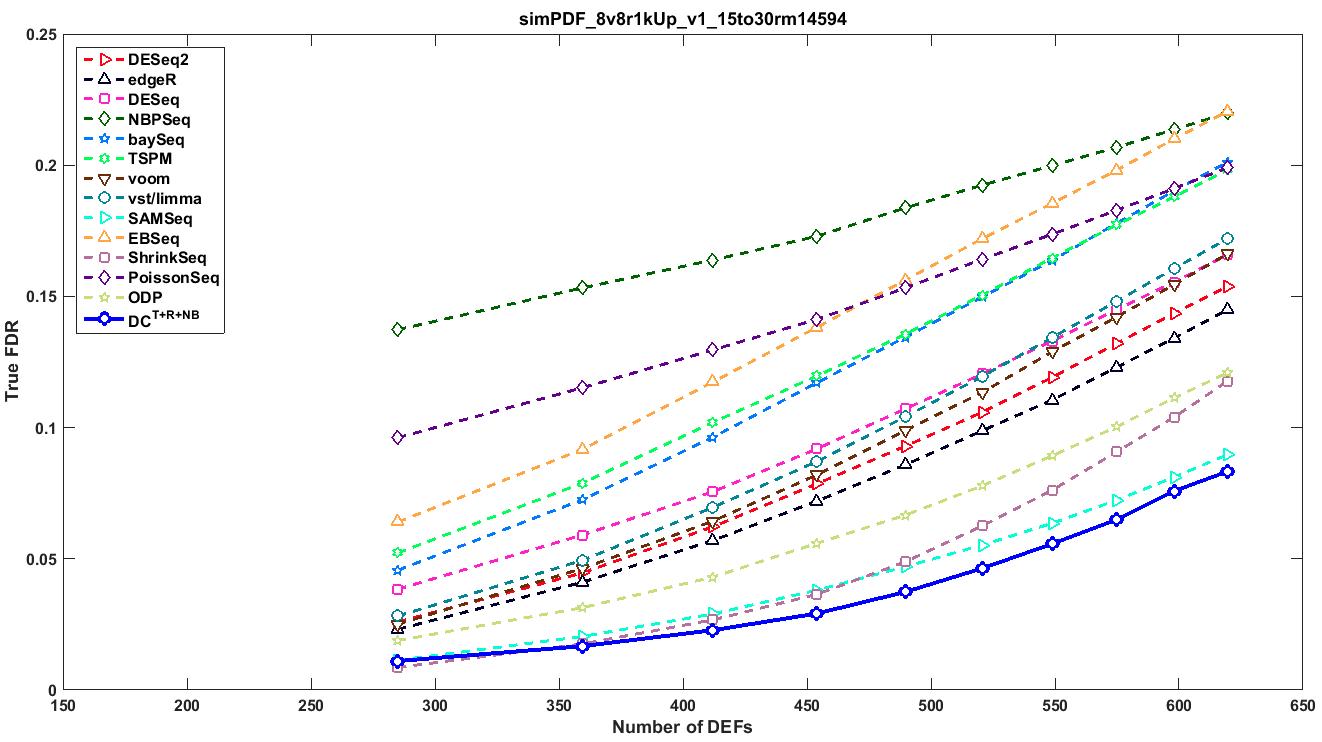


**Number of detected DEFs**

**True FDR**

**Figure S59. The curves of the true FDR *vs.* the number of detected DEFs in a typical simulation test (8 *vs.* 8; $\boldsymbol{G}_{\boldsymbol{0}}^{\boldsymbol{1000}}$)**. The ***x***- and ***y***- axes indicate the number of detected DEFs and the average true FDR, respectively. The curve of DC*^T+R+NB^* (solid curve with blue circle markers) in this figure were converted from the results obtained by setting the target FDR between 0.01 and 0.1 with an increasing step of 0.01. The curves of other methods were obtained by letting them call the same number of DEFs detected by DC*^T+R+NB^* at each target FDR.

**Simulation Test Results of *N* = 20,** $\boldsymbol{G}_{\mathbf{0}}^{\mathbf{1000}}$


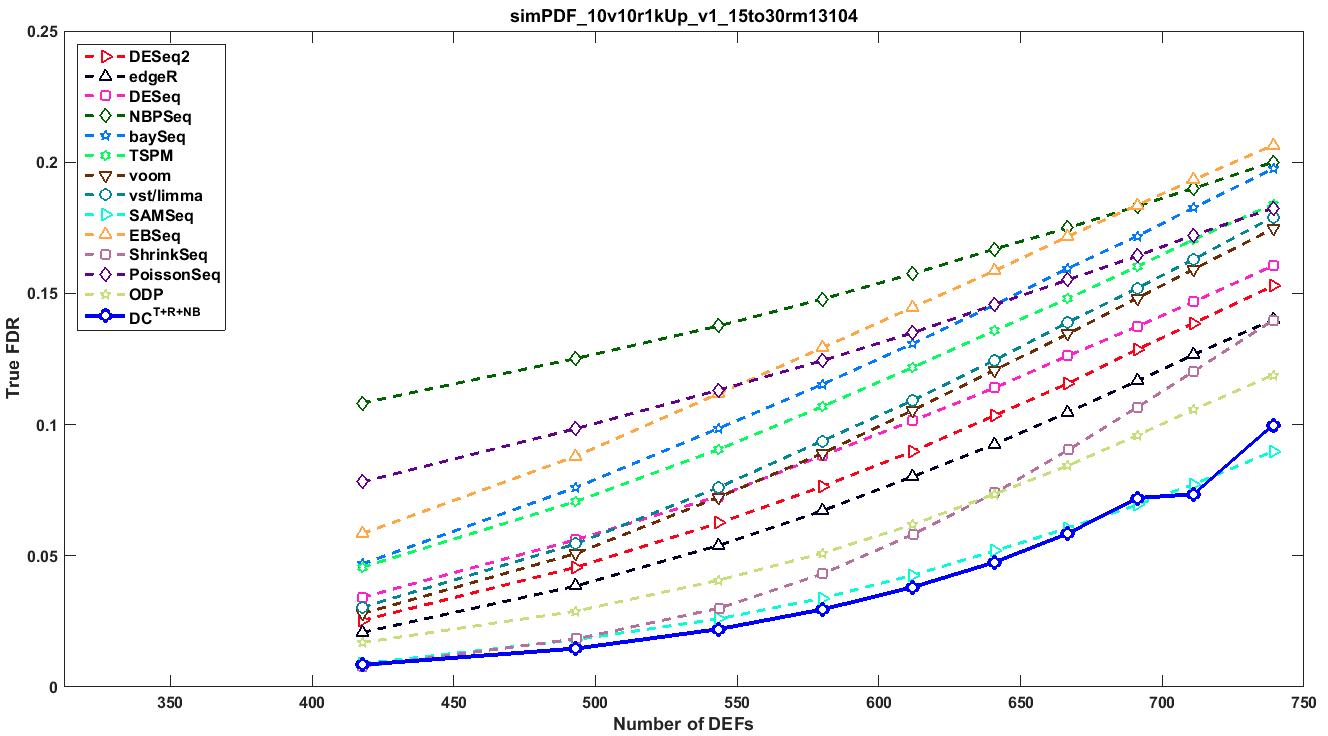


**Number of detected DEFs**

**True FDR**

**Figure S60. The curves of the true FDR *vs.* the number of detected DEFs in a typical simulation test (10 *vs.* 10; $\boldsymbol{G}_{\boldsymbol{0}}^{\boldsymbol{1000}}$)**. The ***x***- and ***y***- axes indicate the number of detected DEFs and the average true FDR, respectively. The curve of DC*^T+R+NB^* (solid curve with blue circle markers) in this figure were converted from the results obtained by setting the target FDR between 0.01 and 0.1 with an increasing step of 0.01. The curves of other methods were obtained by letting them call the same number of DEFs detected by DC*^T+R+NB^* at each target FDR.

# Additional SEQC/MAQC-III Test Results

**Test Results of *N* = 8**


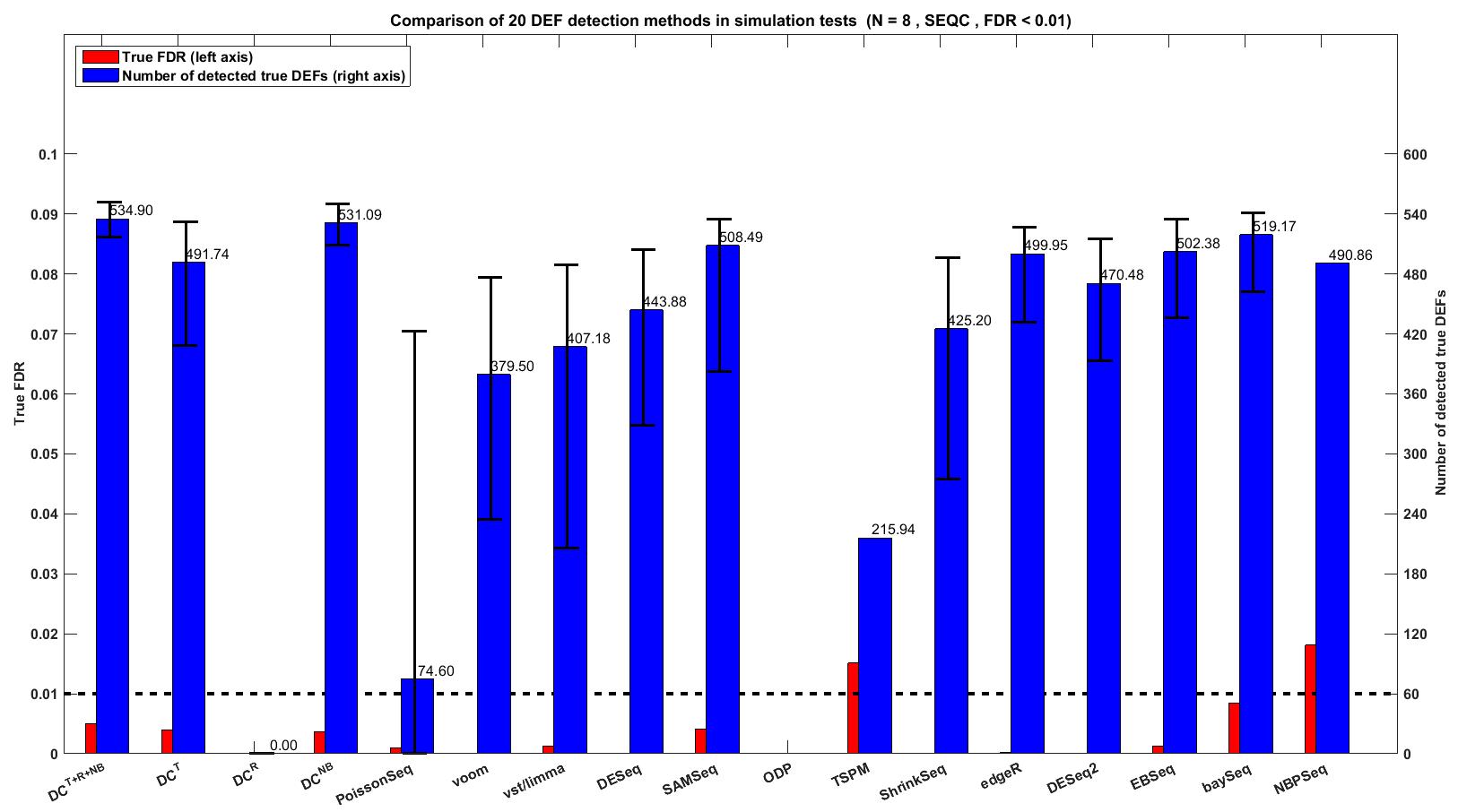

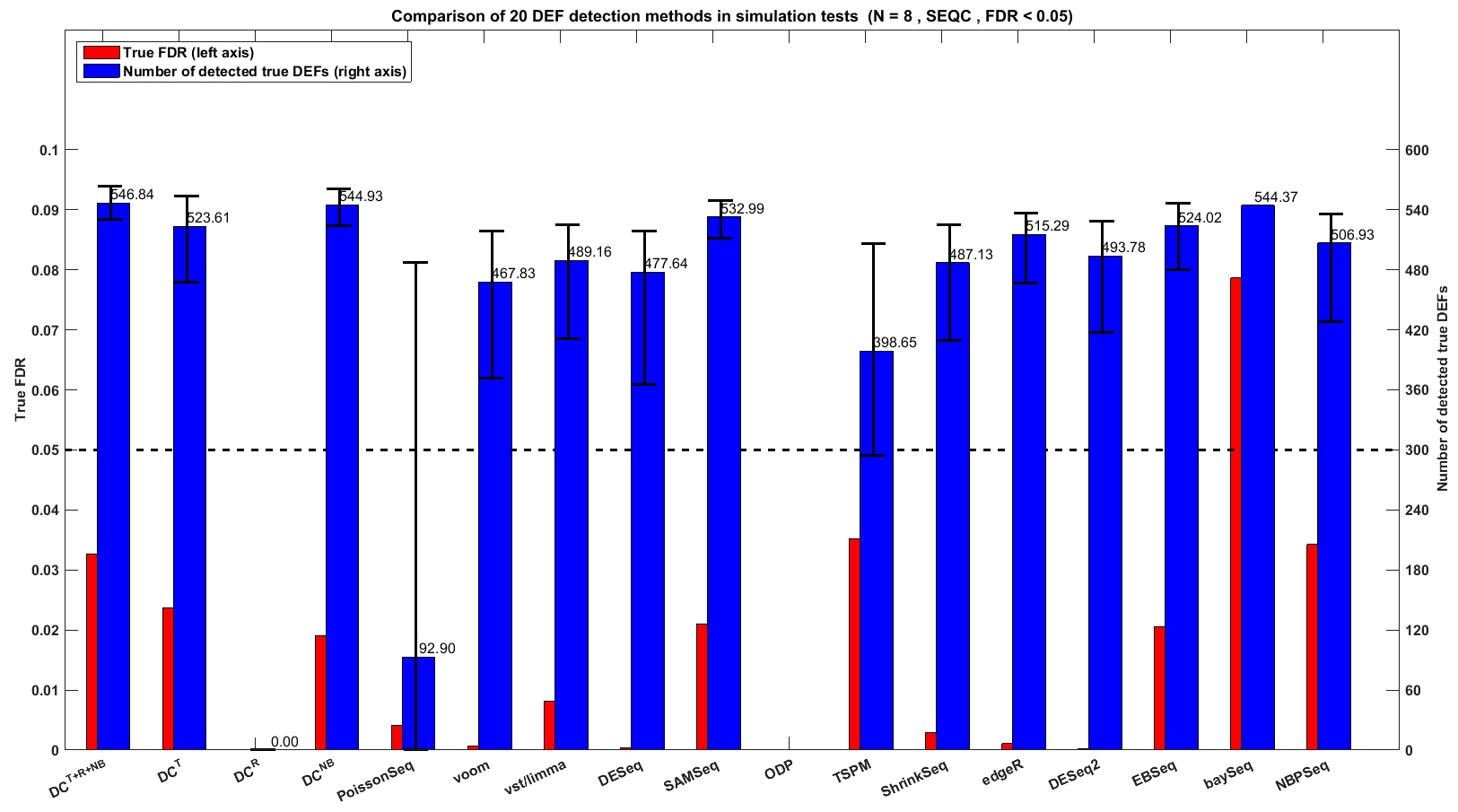


**(a) Comparison at target FDR < 0.01**

**(b) Comparison at target FDR < 0.05**

**True FDR**

**Number of detected true DEFs**

**Number of detected true DEFs**

**True FDR**

**Figure S61. Evaluates RNA-seq differential expression analysis methods using the SEQC/MAQC-III dataset (4 *vs.* 4).** The red bars indicate the average true FDRs (refer to the left ***y***-axis). The horizontal dashed line across the figure marks the target FDR. The blue bars indicate the average number of the detected true DEFs (refer to the right ***y***-axis). The 90% confidence intervals of the detected DEFs are marked except for those whose true FDRs exceed the target FDR by 10%. **(a)** target FDR < 0.01. **(b)** target FDR < 0.05.


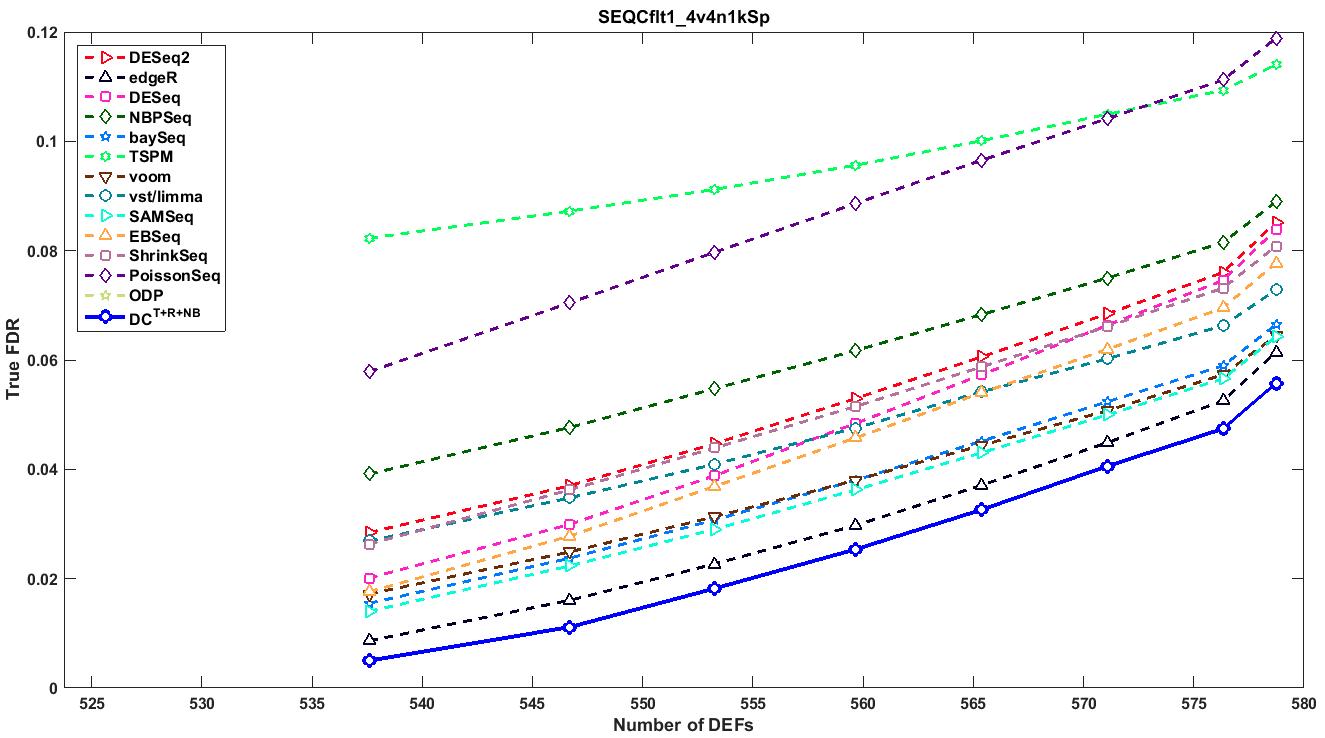


**Number of detected DEFs**

**True FDR**

**Figure S62. The curves of the true positives *vs.* the target FDR using the SEQC/MAQC-III dataset (4 *vs.* 4)**. The ***x***- and ***y***- axes indicate the target FDR level and the average number of true positives, respectively. The solid curve with blue circle markers represents DC*^T+R+NB^*, and other curves represent non-DC methods. The result of a method at a particular target FDR is shown if (1) its true FDR does not exceed the target FDR by 10%; and (2) it detects on average ≥ 0.5 true DEFs (rounds up to 1).


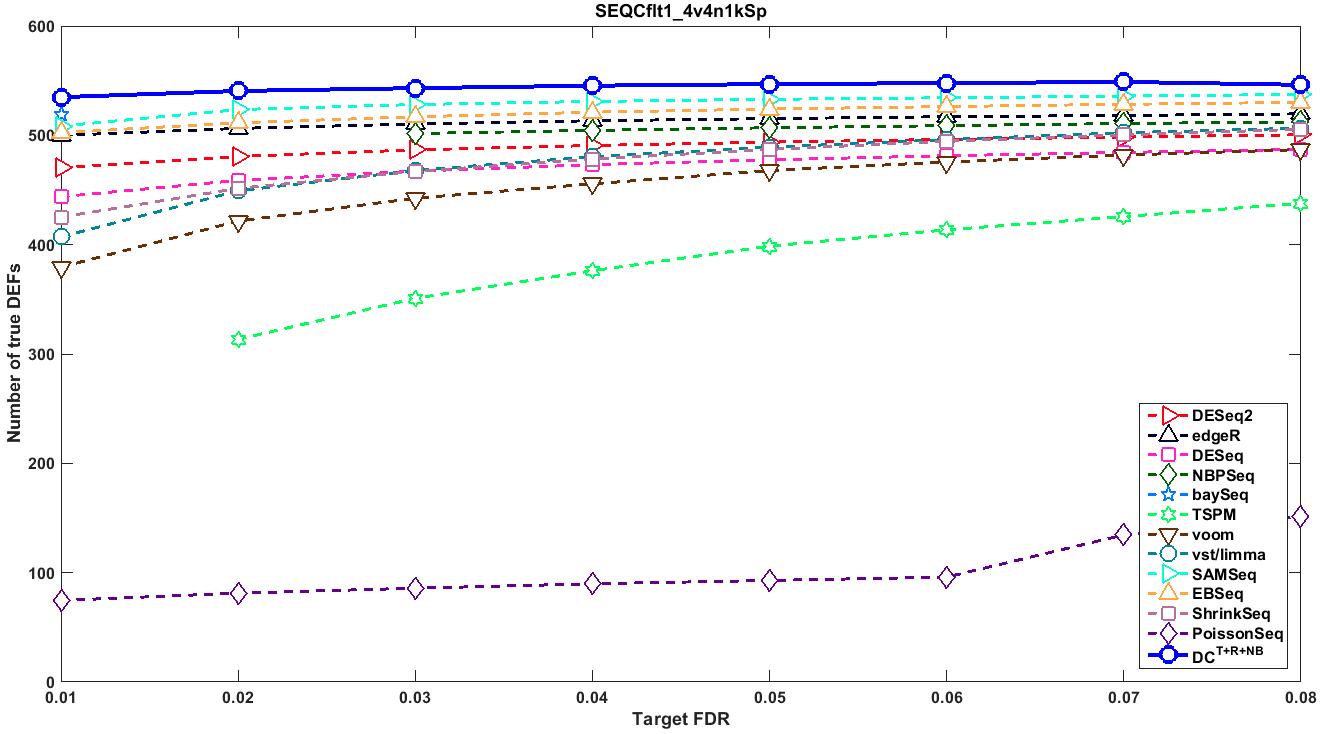


**Target FDR**

**Average number of detected true DEFs**

**Figure S63. The curves of the true positives *vs.* the target FDR using the SEQC/MAQC-III dataset (4 *vs.* 4)**. The ***x***- and ***y***- axes indicate the target FDR level and the average number of true positives, respectively. The solid curve with blue circle markers represents DC*^T+R+NB^*, and other curves represent non-DC methods. The result of a method at a particular target FDR is shown if (1) its true FDR does not exceed the target FDR by 10%; and (2) it detects on average ≥ 0.5 true DEFs (rounds up to 1).

**Test Results of *N* = 10**


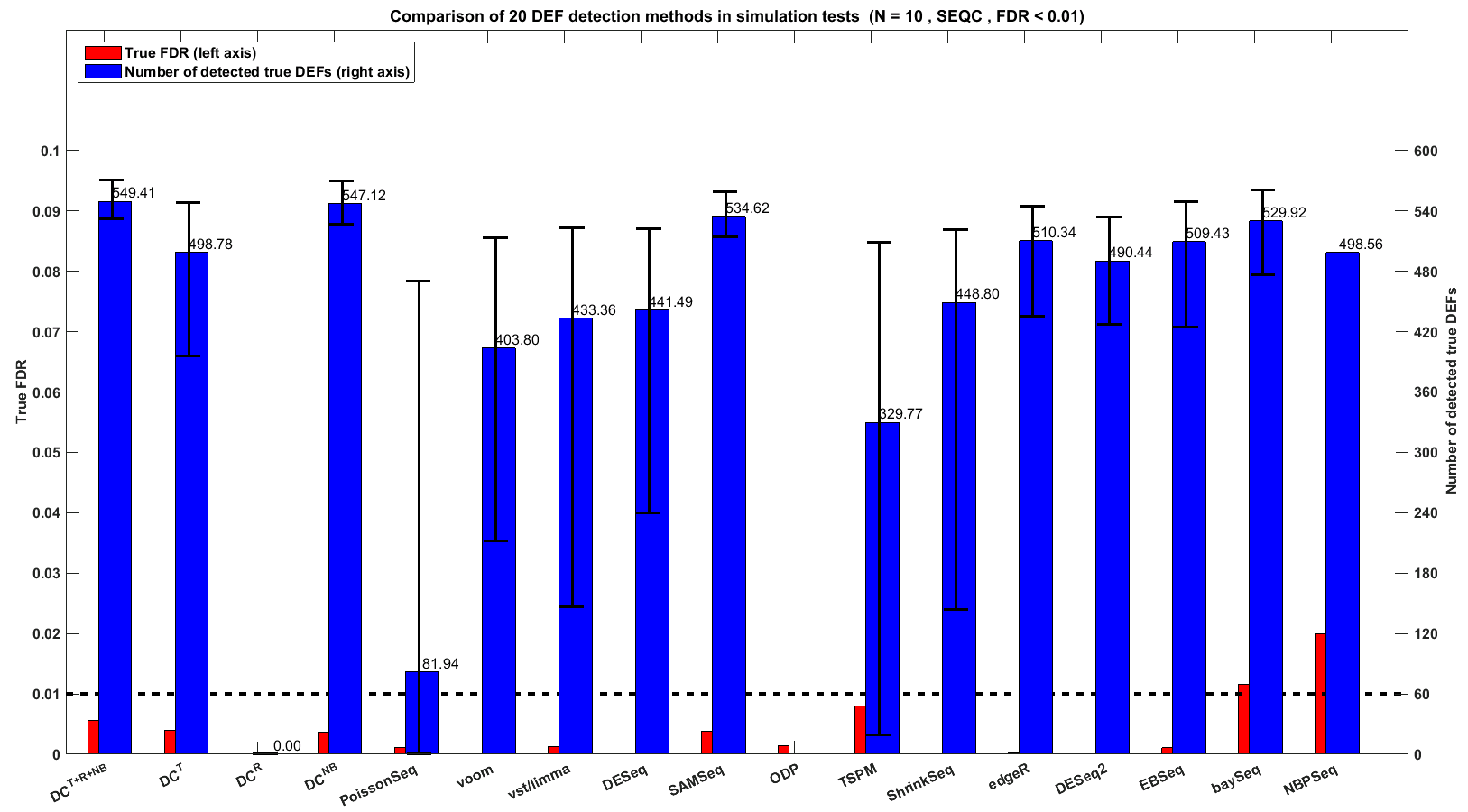

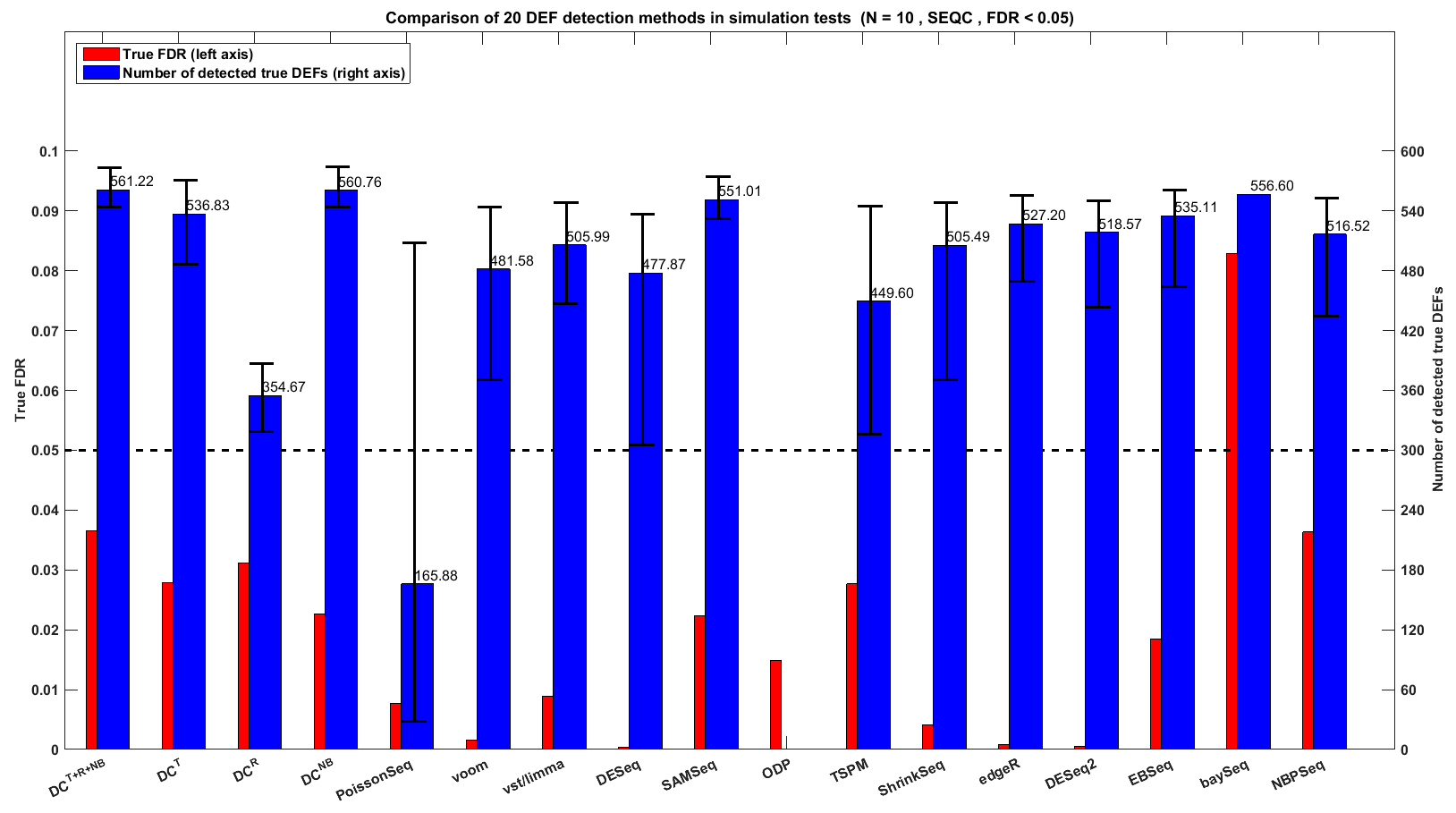


**(a) Comparison at target FDR < 0.01**

**(b) Comparison at target FDR < 0.05**

**True FDR**

**Number of detected true DEFs**

**Number of detected true DEFs**

**True FDR**

**Figure S64. Evaluates RNA-seq differential expression analysis methods using the SEQC/MAQC-III dataset (5 *vs.* 5).** The red bars indicate the average true FDRs (refer to the left ***y***-axis). The horizontal dashed line across the figure marks the target FDR. The blue bars indicate the average number of the detected true DEFs (refer to the right ***y***-axis). The 90% confidence intervals of the detected DEFs are marked except for those whose true FDRs exceed the target FDR by 10%. **(a)** target FDR < 0.01. **(b)** target FDR < 0.05.


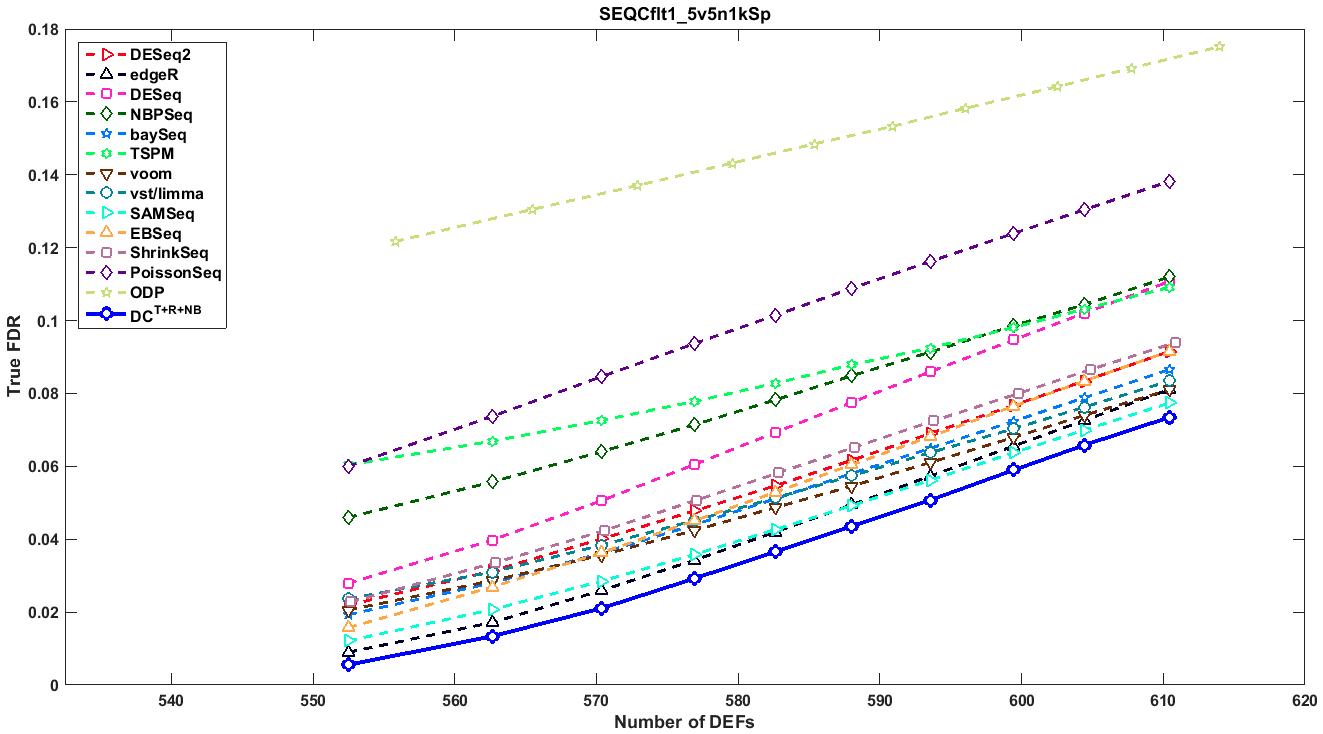


**Number of detected DEFs**

**True FDR**

**Figure S65. The curves of the true positives *vs.* the target FDR using the SEQC/MAQC-III dataset (5 *vs.* 5)**. The ***x***- and ***y***- axes indicate the target FDR level and the average number of true positives, respectively. The solid curve with blue circle markers represents DC*^T+R+NB^*, and other curves represent non-DC methods. The result of a method at a particular target FDR is shown if (1) its true FDR does not exceed the target FDR by 10%; and (2) it detects on average ≥ 0.5 true DEFs (rounds up to 1).


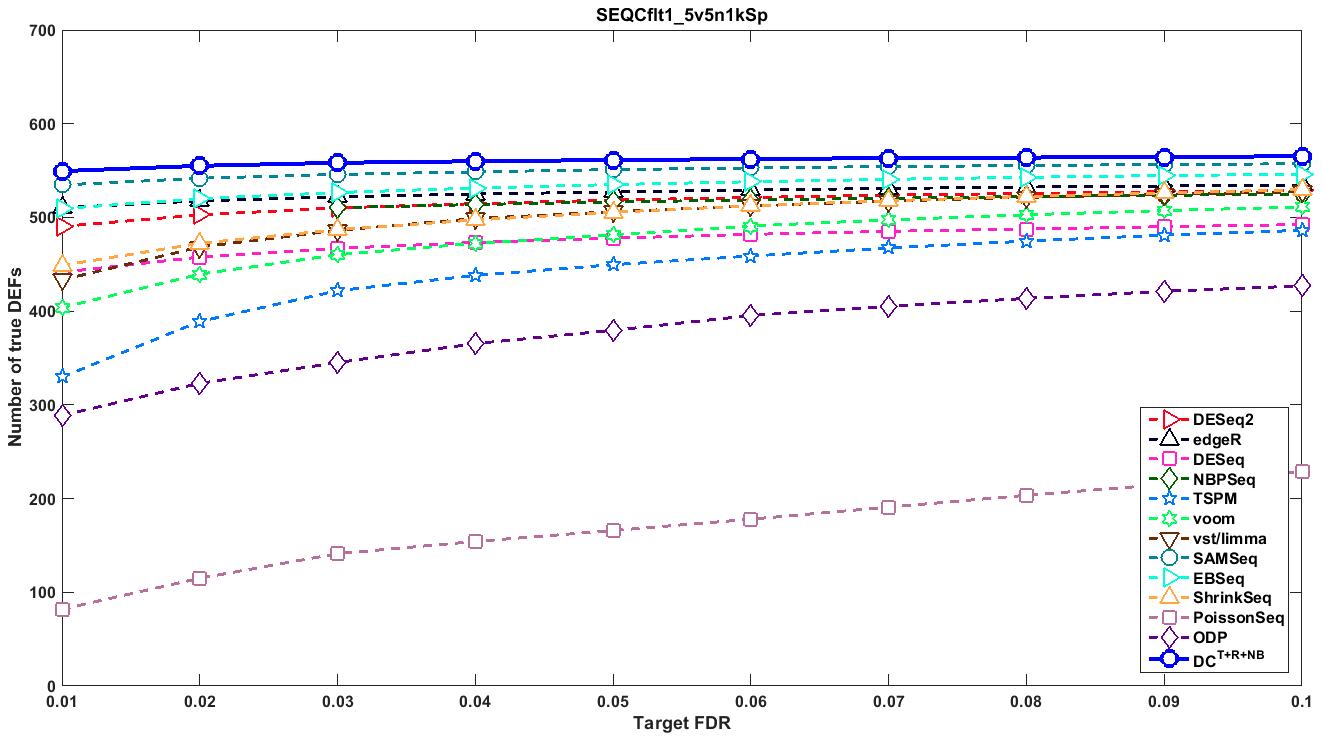


**Target FDR**

**Average number of detected true DEFs**

**Figure S66. The curves of the true positives *vs.* the target FDR using the SEQC/MAQC-III dataset (5 *vs.* 5)**. The ***x***- and ***y***- axes indicate the target FDR level and the average number of true positives, respectively. The solid curve with blue circle markers represents DC*^T+R+NB^*, and other curves represent non-DC methods. The result of a method at a particular target FDR is shown if (1) its true FDR does not exceed the target FDR by 10%; and (2) it detects on average ≥ 0.5 true DEFs (rounds up to 1).

**Test Results of *N* = 12**


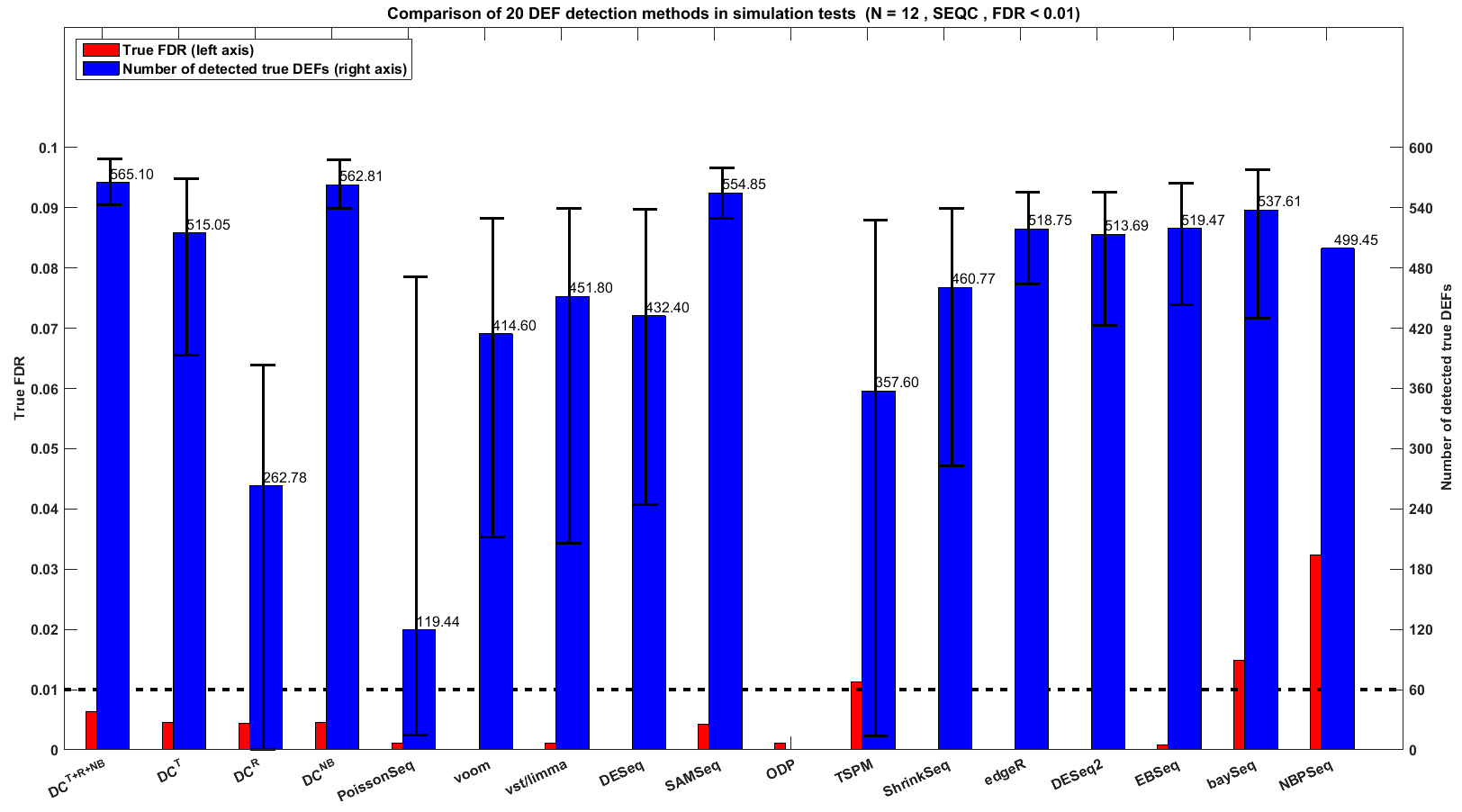

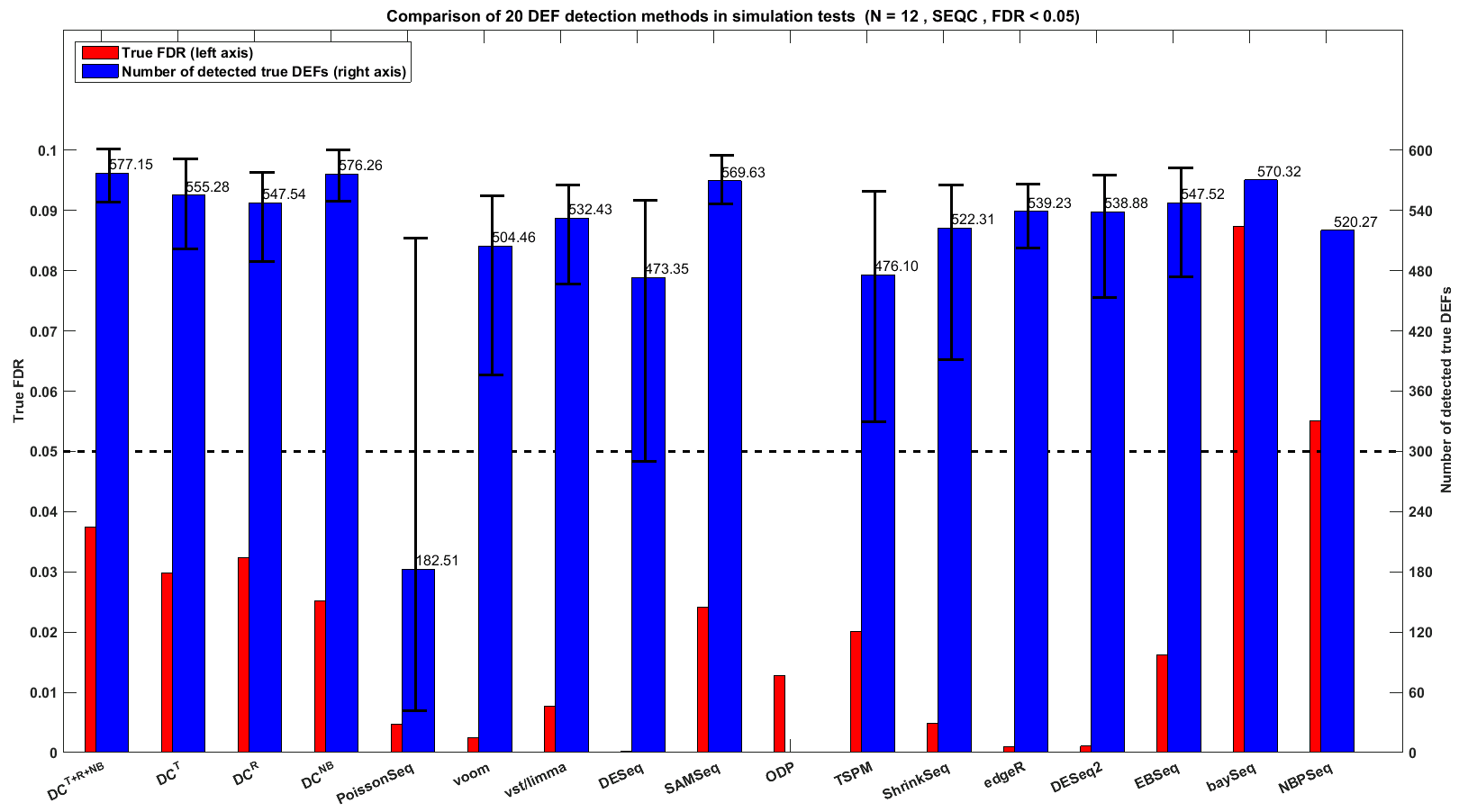


**(a) Comparison at target FDR < 0.01**

**(b) Comparison at target FDR < 0.05**

**True FDR**

**Number of detected true DEFs**

**Number of detected true DEFs**

**True FDR**

**Figure S67. Evaluates RNA-seq differential expression analysis methods using the SEQC/MAQC-III dataset (6 *vs.* 6).** The red bars indicate the average true FDRs (refer to the left ***y***-axis). The horizontal dashed line across the figure marks the target FDR. The blue bars indicate the average number of the detected true DEFs (refer to the right ***y***-axis). The 90% confidence intervals of the detected DEFs are marked except for those whose true FDRs exceed the target FDR by 10%. **(a)** target FDR < 0.01. **(b)** target FDR < 0.05.


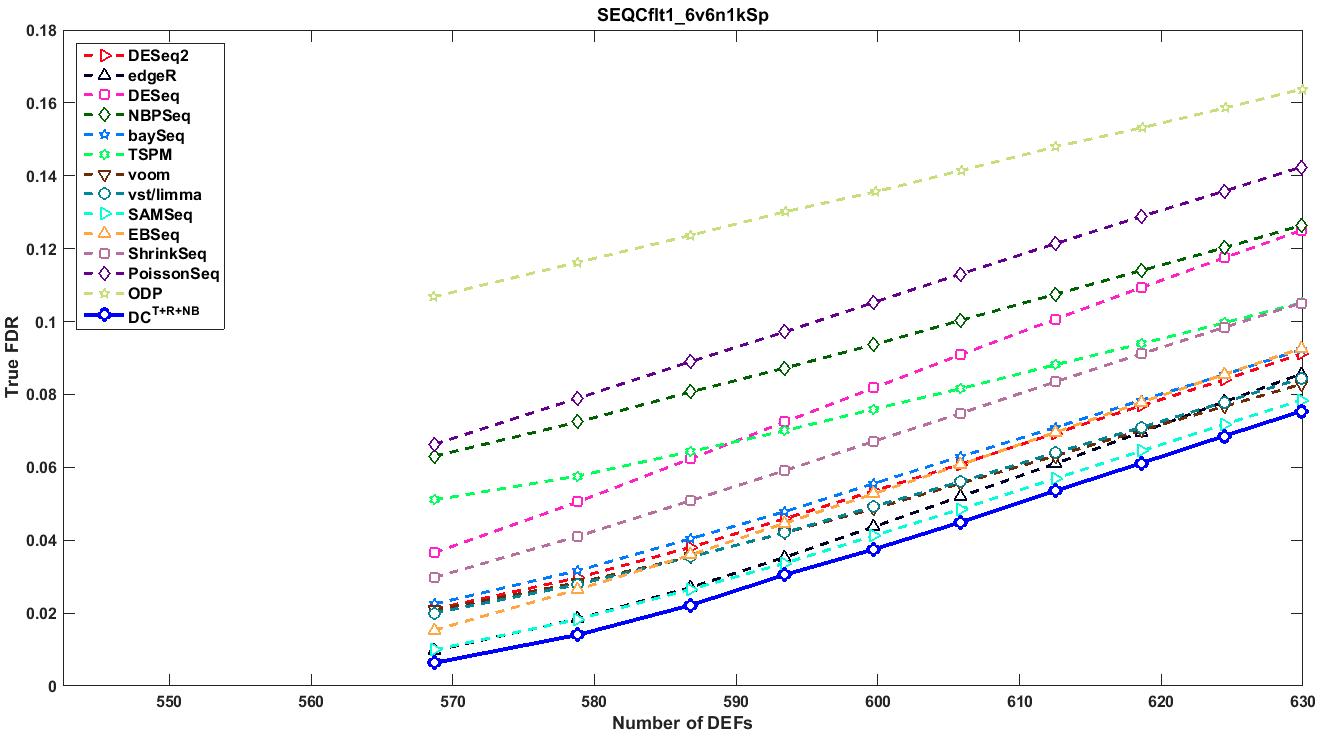


**Number of detected DEFs**

**True FDR**

**Figure S68. The curves of the true positives *vs.* the target FDR using the SEQC/MAQC-III dataset (6 *vs.* 6)**. The ***x***- and ***y***- axes indicate the target FDR level and the average number of true positives, respectively. The solid curve with blue circle markers represents DC*^T+R+NB^*, and other curves represent non-DC methods. The result of a method at a particular target FDR is shown if (1) its true FDR does not exceed the target FDR by 10%; and (2) it detects on average ≥ 0.5 true DEFs (rounds up to 1).


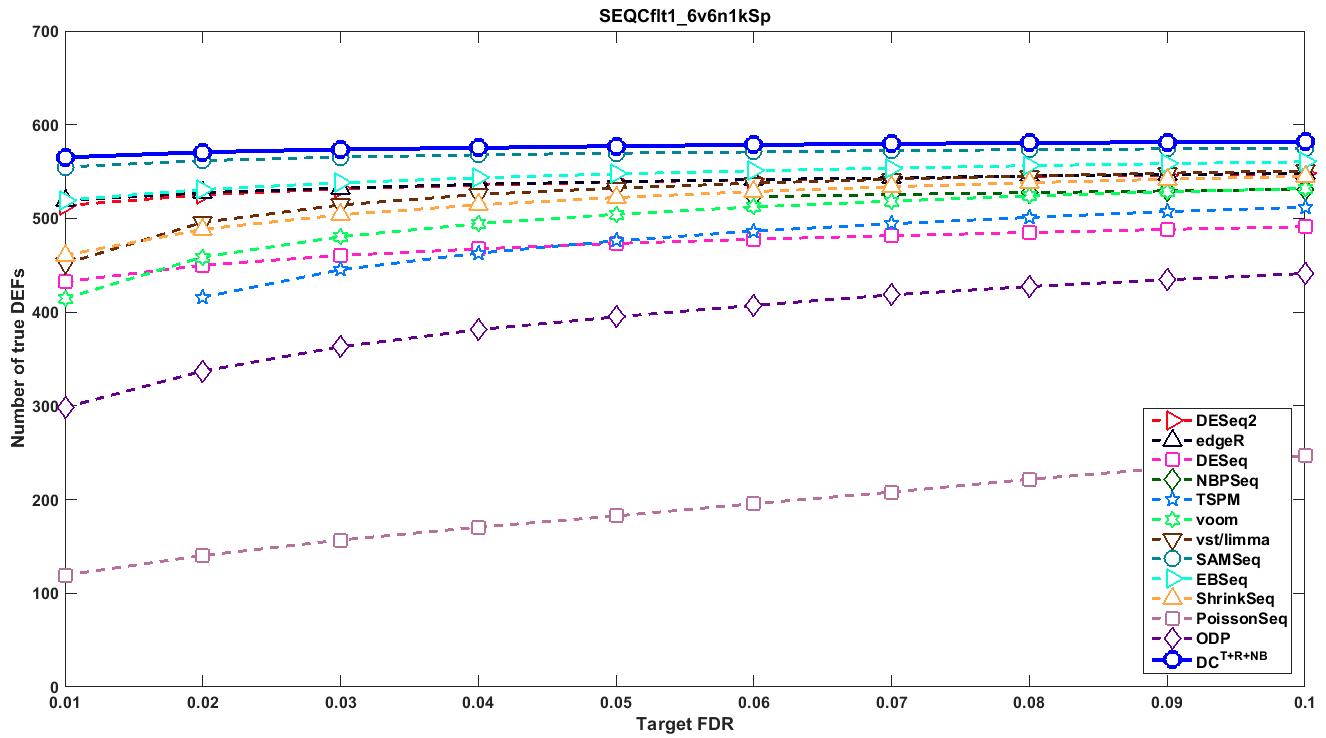


**Target FDR**

**Average number of detected true DEFs**

**Figure S69. The curves of the true positives *vs.* the target FDR using the SEQC/MAQC-III dataset (6 *vs.* 6)**. The ***x***- and ***y***- axes indicate the target FDR level and the average number of true positives, respectively. The solid curve with blue circle markers represents DC*^T+R+NB^*, and other curves represent non-DC methods. The result of a method at a particular target FDR is shown if (1) its true FDR does not exceed the target FDR by 10%; and (2) it detects on average ≥ 0.5 true DEFs (rounds up to 1).

**Test Results of *N* = 16**


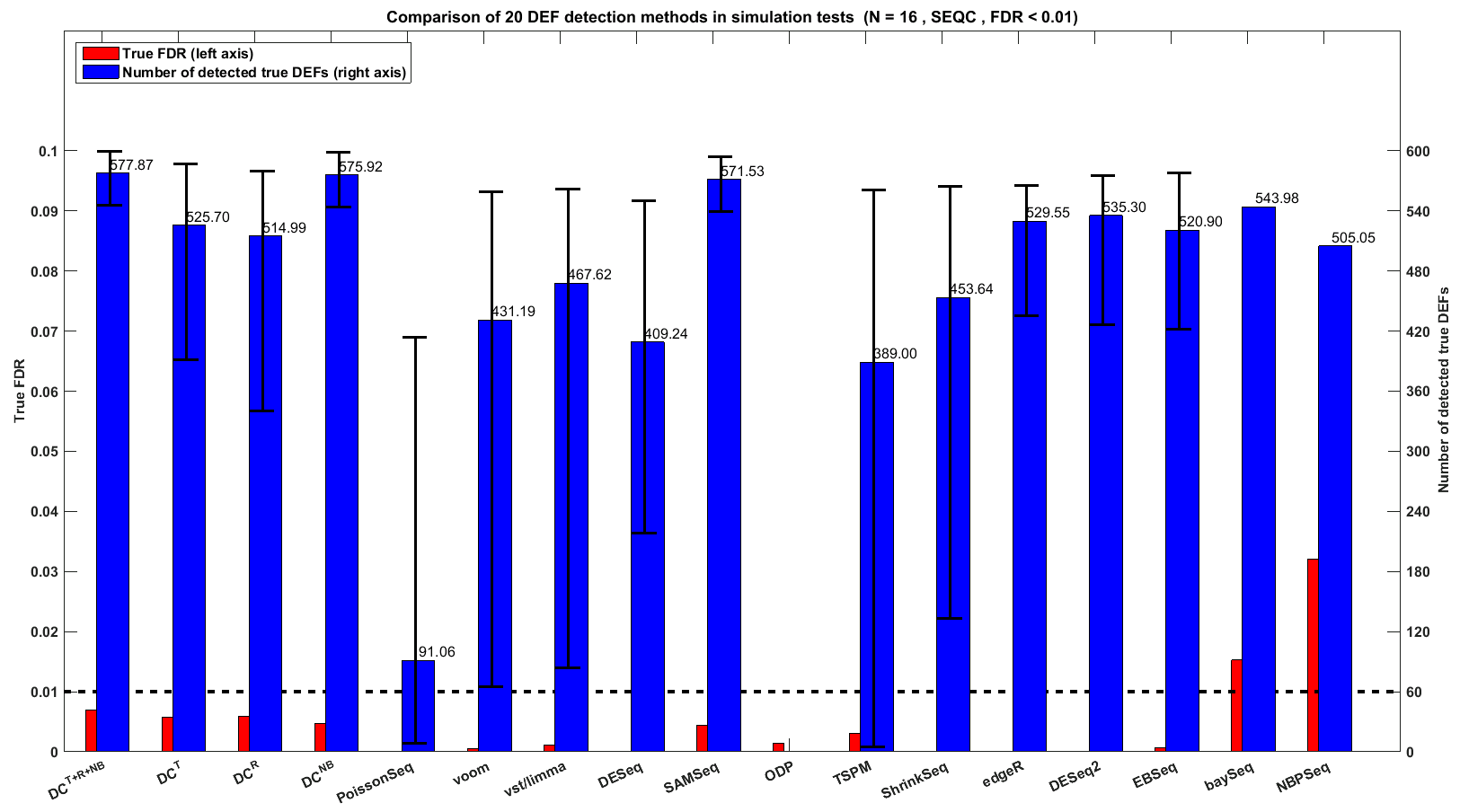

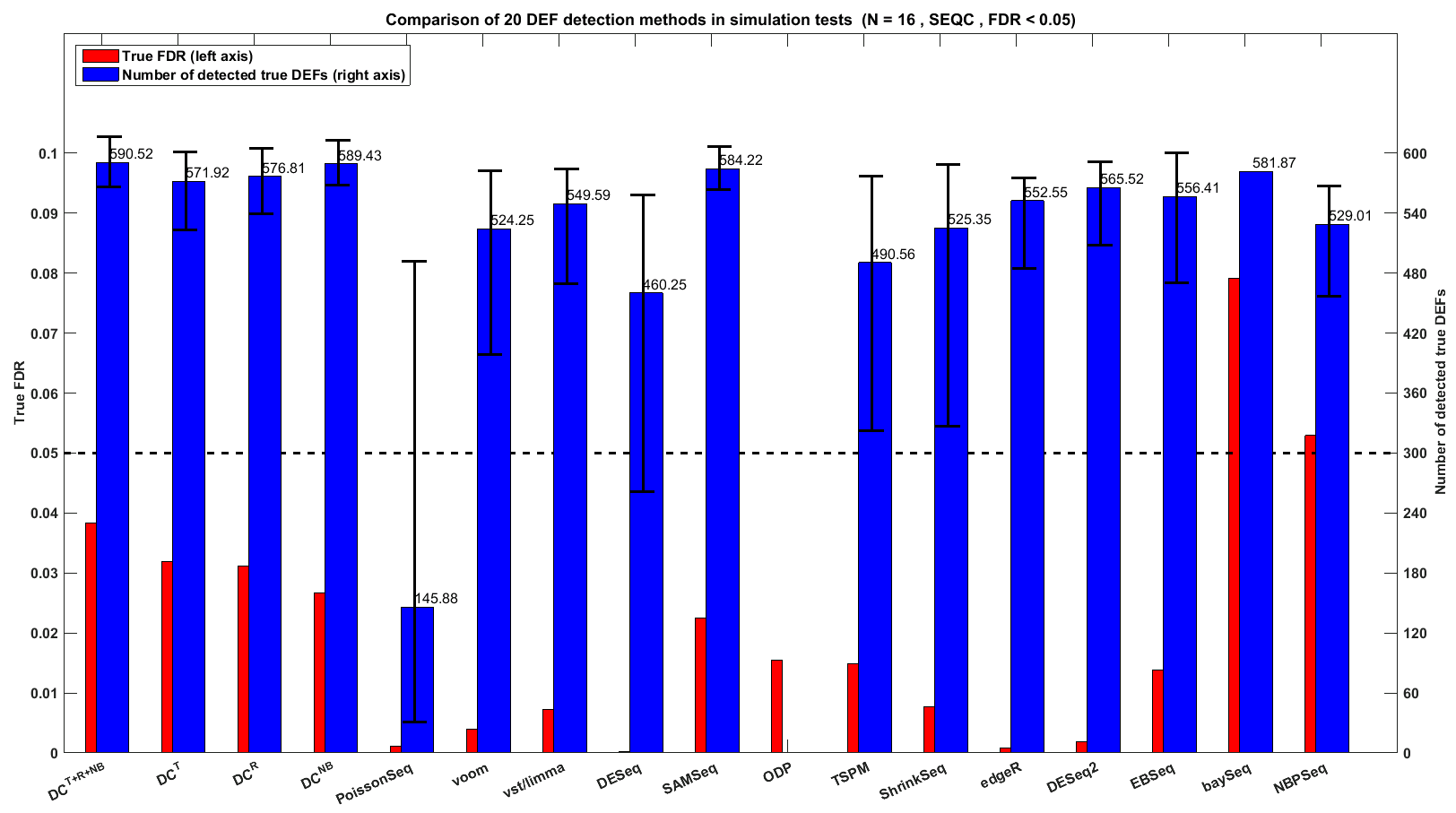


**(a) Comparison at target FDR < 0.01**

**(b) Comparison at target FDR < 0.05**

**True FDR**

**Number of detected true DEFs**

**Number of detected true DEFs**

**True FDR**

**Figure S70. Evaluates RNA-seq differential expression analysis methods using the SEQC/MAQC-III dataset (8 *vs.* 8).** The red bars indicate the average true FDRs (refer to the left ***y***-axis). The horizontal dashed line across the figure marks the target FDR. The blue bars indicate the average number of the detected true DEFs (refer to the right ***y***-axis). The 90% confidence intervals of the detected DEFs are marked except for those whose true FDRs exceed the target FDR by 10%. **(a)** target FDR < 0.01. **(b)** target FDR < 0.05.


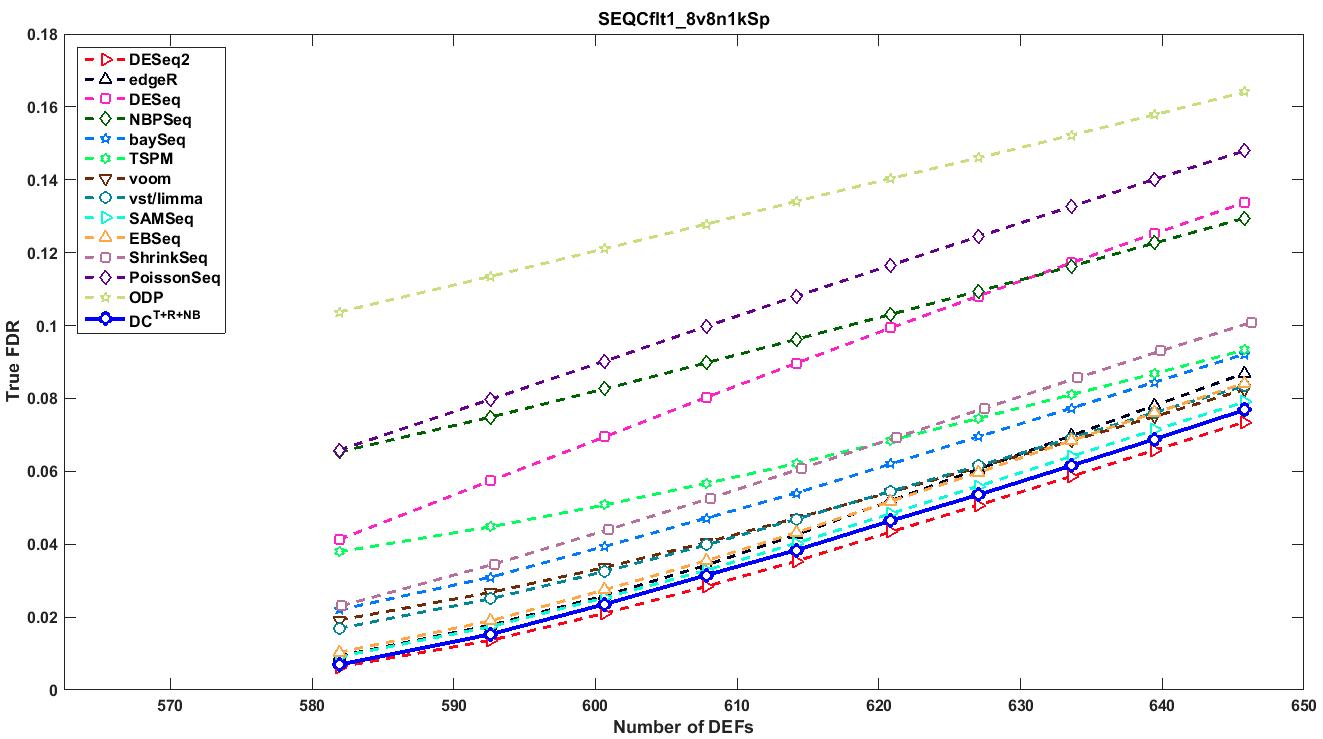


**Number of detected DEFs**

**True FDR**

**Figure S71. The curves of the true positives *vs.* the target FDR using the SEQC/MAQC-III dataset (8 *vs.* 8)**. The ***x***- and ***y***- axes indicate the target FDR level and the average number of true positives, respectively. The solid curve with blue circle markers represents DC*^T+R+NB^*, and other curves represent non-DC methods. The result of a method at a particular target FDR is shown if (1) its true FDR does not exceed the target FDR by 10%; and (2) it detects on average ≥ 0.5 true DEFs (rounds up to 1).


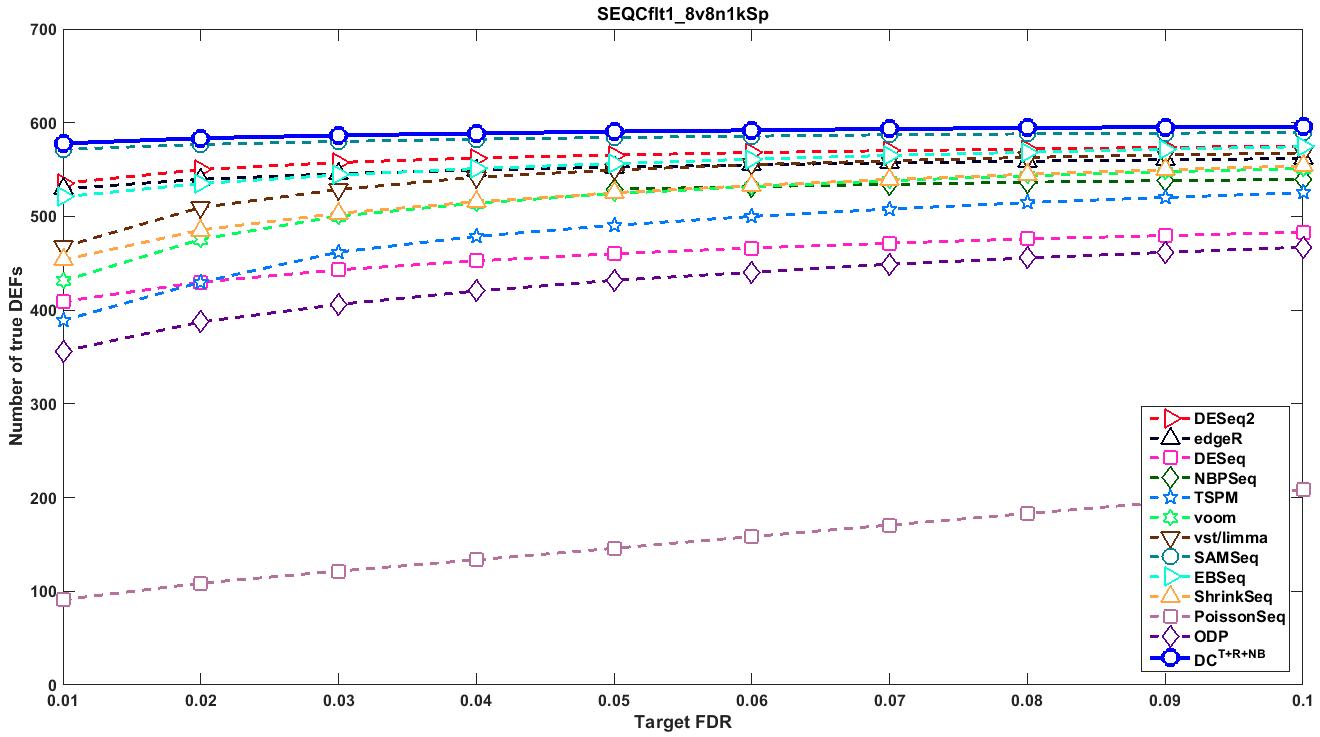


**Target FDR**

**Average number of detected true DEFs**

**Figure S72. The curves of the true positives *vs.* the target FDR using the SEQC/MAQC-III dataset (8 *vs.* 8)**. The ***x***- and ***y***- axes indicate the target FDR level and the average number of true positives, respectively. The solid curve with blue circle markers represents DC*^T+R+NB^*, and other curves represent non-DC methods. The result of a method at a particular target FDR is shown if (1) its true FDR does not exceed the target FDR by 10%; and (2) it detects on average ≥ 0.5 true DEFs (rounds up to 1).

# R-codes for the RNA-seq DEF Detection Methods Used in Comparisons

All DEF detection methods were called using the default parameters unless indicated otherwise. The codes for calling DEF detection methods (except for DESeq2 and ODP) were obtained from [[1](#_ENREF_1)].

## DESeq2

# The DESeq2 package (v1.6.3) was installed from Bioconductor.

library(DESeq2)

DESeq2.dds = DESeqDataSetFromMatrix(countData = count.matrix,

colData = data.frame(grp = class), design = ~ grp)

DESeq2.dds = DESeq(DESeq2.dds)

DESeq2.res = results(DESeq2.dds)

DESeq2.stat = DESeq2.res$stat

DESeq2.pvalues = DESeq2.res$pvalue

DESeq2.adjpvalues = DESeq2.res$padj

## DESeq

# The DESeq package (v1.18.0) can be installed from Bioconductor.

library(DESeq)

DESeq.cds = newCountDataSet(countData = count.matrix, conditions = factor(class))

DESeq.cds = estimateSizeFactors(DESeq.cds)

DESeq.cds = estimateDispersions(DESeq.cds, sharingMode = "maximum",

method = "pooled", fitType = "local")

DESeq.test = nbinomTest(DESeq.cds, "1", "2")

DESeq.pvalues = DESeq.test$pval

DESeq.adjpvalues = p.adjust(DESeq.pvalues, method = "BH")

## voom/limma

# The limma package (v3.22.6) can be installed from Bioconductor.

library(limma)

library(edgeR)

nf = calcNormFactors(count.matrix, method = "TMM")

voom.data = voom(count.matrix, design = model.matrix(~factor(class)),

lib.size = colSums(count.matrix) * nf)

voom.data$genes = rownames(count.matrix)

voom.fitlimma = lmFit(voom.data, design = model.matrix(~factor(class)))

voom.fitbayes = eBayes(voom.fitlimma)

voom.pvalues = voom.fitbayes$p.value[, 2]

voom.adjpvalues = p.adjust(voom.pvalues, method = "BH")

## vst/limma

# The limma package (v3.22.6) was installed from Bioconductor.

# The DESeq package (v1.18.0) was installed from Bioconductor.

library(DESeq)

library(limma)

DESeq.cds = newCountDataSet(countData = count.matrix,

conditions = factor(class))

DESeq.cds = estimateSizeFactors(DESeq.cds)

DESeq.cds = estimateDispersions(DESeq.cds, method = "blind",

fitType = "local")

DESeq.vst = getVarianceStabilizedData(DESeq.cds)

DESeq.vst.fitlimma = lmFit(DESeq.vst, design = model.matrix(~factor(class)))

DESeq.vst.fitbayes = eBayes(DESeq.vst.fitlimma)

DESeq.vst.pvalues = DESeq.vst.fitbayes$p.value[, 2]

DESeq.vst.adjpvalues = p.adjust(DESeq.vst.pvalues, method = "BH")

## edgeR

# The edgeR package (v3.8.5) was installed from Bioconductor.

library(edgeR)

edgeR.dgelist = DGEList(counts = count.matrix, group = factor(class))

edgeR.dgelist = calcNormFactors(edgeR.dgelist, method = "TMM")

edgeR.dgelist = estimateCommonDisp(edgeR.dgelist)

edgeR.dgelist = estimateTagwiseDisp(edgeR.dgelist, trend = "movingave")

edgeR.test = exactTest(edgeR.dgelist)

edgeR.pvalues = edgeR.test$table$PValue

edgeR.adjpvalues = p.adjust(edgeR.pvalues, method = "BH")

## NBPSeq

# The NBPSeq package (v0.3.0) was installed from Bioconductor.

library(edgeR)

library(NBPSeq)

NBPSeq.dgelist = DGEList(counts = count.matrix, group = factor(class))

NBPSeq.dgelist = calcNormFactors(NBPSeq.dgelist, method = "TMM")

NBPSeq.norm.factors = as.vector(NBPSeq.dgelist$samples$norm.factors)

NBPSeq.test = nbp.test(counts = count.matrix, grp.ids = class,

grp1 = 1, grp2 = 2, norm.factors = NBPSeq.norm.factors,

model.disp = "NBP")

NBPSeq.pvalues = NBPSeq.test$p.values

NBPSeq.adjpvalues = NBPSeq.test$q.values

## baySeq

# The baySeq package (v2.0.50) was installed from Bioconductor.

library(baySeq)

baySeq.cd = new("countData", data = count.matrix, replicates = class,

groups = list(NDE = rep(1, length(class)), DE = class))

libsizes(baySeq.cd) <- getLibsizes(baySeq.cd, estimationType = "edgeR")

baySeq.cd = getPriors.NB(baySeq.cd, equalDispersions = TRUE, estimation = "QL", cl = NULL)

baySeq.cd = getLikelihoods.NB(baySeq.cd, prs = c(0.5, 0.5), pET = "BIC", cl = NULL)

baySeq.posteriors.DE = exp(baySeq.cd@posteriors)[, 2]

baySeq.table = topCounts(baySeq.cd, group = "DE", FDR = 1)

baySeq.FDR = baySeq.table$FDR.DE[match(1:nrow(count.matrix),baySeq.table$rowID)]

baySeq.Likelihood = baySeq.table$Likelihood[match(1:nrow(count.matrix),baySeq.table$rowID)]

## EBSeq

# The EBSeq package (v1.6.0) was installed from Bioconductor.

# It is suggested that the “maxround” argument of EBSeq should be at least 5. We set it as 10.

library(EBSeq)

sizes = MedianNorm(count.matrix)

rownames(count.matrix) <- 1:nrow(count.matrix)

EBSeq.test = EBTest(Data = count.matrix, Conditions = factor(class), sizeFactors = sizes, maxround = 10)

EBSeq.ppmat = GetPPMat(EBSeq.test)

EBSeq.probabilities.DE = EBSeq.ppmat[, "PPDE"]

EBSeq.lFDR = 1 - EBSeq.ppmat[, "PPDE"]

EBSeq.FDR = rep(NA, length(EBSeq.lFDR))

for (i in 1:length(EBSeq.lFDR)) {

EBSeq.FDR[i] = mean(EBSeq.lFDR[which(EBSeq.lFDR <=

EBSeq.lFDR[i])])

}

## TSPM

# The TSPM R script was downloaded from <http://www.stat.purdue.edu/~doerge/software/TSPM.R> (as of Feb. 17^th^, 2015).

library(edgeR)

source("TSPM.R")

TSPM.dgelist = DGEList(counts = count.matrix, group = factor(class))

TSPM.dgelist = calcNormFactors(TSPM.dgelist, method = "TMM")

norm.lib.sizes = as.vector(TSPM.dgelist$samples$norm.factors) *

as.vector(TSPM.dgelist$samples$lib.size)

TSPM.test = TSPM(counts = count.matrix, x1 = factor(class),

x0 = rep(1, length(class)), lib.size = norm.lib.sizes)

TSPM.pvalues = TSPM.test$pvalues

TSPM.adjpvalues = TSPM.test$padj

## SAMSeq

# The samr package (v2.0) was installed from CRAN.

library(samr)

SAMseq.test = SAMseq(count.matrix, class, resp.type = "Two class unpaired",

geneid = rownames(count.matrix), genenames = rownames(count.matrix), fdr.output = 1)

SAMseq.result.table = rbind(SAMseq.test$siggenes.table$genes.up,

SAMseq.test$siggenes.table$genes.lo)

SAMseq.score = rep(0, nrow(count.matrix))

SAMseq.score[match(SAMseq.result.table[,1], rownames(count.matrix))]

= as.numeric(SAMseq.result.table[,3])

SAMseq.FDR = rep(1, nrow(count.matrix))

SAMseq.FDR[match(SAMseq.result.table[,1], rownames(count.matrix))]

= as.numeric(SAMseq.result.table[,5])/100

## ShrinkBayes

# The ShrinkBayes package (v2.12) was downloaded from <http://www.few.vu.nl/~mavdwiel/ShrinkBayes.html>

# It is suggested that the “maxiter” argument of ShrinkBayes should be at least 10. We set it as 15.

library(ShrinkBayes)

library(edgeR)

nf = calcNormFactors(count.matrix, method = "TMM") *

colSums(count.matrix)/exp(mean(log(colSums(count.matrix))))

count.matrix = round(sweep(count.matrix, 2, nf, "/"))

group = factor(class)

form = y ~ 1 + group

ShrinkSeq.shrinkres = ShrinkSeq(form = form, dat = count.matrix,

shrinkfixed = "group", mixtdisp = FALSE, shrinkdisp = TRUE,

fams = "zinb", ncpus = 1)

ShrinkSeq.fitzinb = FitAllShrink(forms = form, dat = count.matrix,

fams = "zinb", shrinksimul = ShrinkSeq.shrinkres, ncpus = 1)

ShrinkSeq.npprior = NonParaUpdatePrior(fitall = ShrinkSeq.fitzinb,

modus = "fixed", shrinkpara = "group", maxiter = 15, ncpus = 1)

ShrinkSeq.nppostshr = NonParaUpdatePosterior(ShrinkSeq.fitzinb, ShrinkSeq.npprior, ncpus = 1)

ShrinkSeq.lfdrless = SummaryWrap(ShrinkSeq.nppostshr, thr = 0, direction = "lesser")

ShrinkSeq.lfdrgreat = SummaryWrap(ShrinkSeq.nppostshr, thr = 0, direction = "greater")

ShrinkSeq.FDR = BFDR(ShrinkSeq.lfdrless, ShrinkSeq.lfdrgreat)

## PoissonSeq

# The PoissonSeq package (v1.1.2) was installed from CRAN.

library(PoissonSeq)

PoissonSeq .dat = list(n = count.matrix, y = class, type = "twoclass", pair = F)

PoissonSeq.res = PS.Main(PoissonSeq.dat)

PoissonSeq.pvalues = rep(1, nrow(count.matrix))

PoissonSeq.fdr = rep(1, nrow(count.matrix))

PoissonSeq.pvalues[PoissonSeq.res$gname] = PoissonSeq.res$pval

PoissonSeq.fdr[PoissonSeq.res$gname] = PoissonSeq.res$fdr

## ODP

# The edge package (v2.0.0) was installed from Bioconductor.

library(edge)

library(splines)

de_obj = build_study(data = count.matrix, grp = factor(class), sampling = "static")

de_fit = fit_models(de_obj, stat.type = "odp")

de_clust = kl_clust(de_obj)

de_odp = odp(de_obj, de.fit = de_fit, odp.parms = de_clust)

edge.pvalues = de_odp@qvalueObj$pvalues

edge.fdr = de_odp@qvalueObj$qvalues

# References

1. Soneson C, Delorenzi M. A comparison of methods for differential expression analysis of RNA-seq data. BMC bioinformatics. 2013;14:91. doi:10.1186/1471-2105-14-91.
